# Isolation, Identification, and Total Synthesis of Pyranoquinolinone Alkaloids from *Conchocarpus mastigophorus* Kallunki (Rutaceae)

**DOI:** 10.1021/acs.jnatprod.5c01326

**Published:** 2026-01-15

**Authors:** Anderson R. Santos, Vanderlúcia F. de Paula, Amanda S. de Miranda, Júnio G. Silva, Luiz C. A. Barbosa

**Affiliations:** † Department of Chemistry, Universidade Federal de Minas Gerais, Av. Pres. Antonio Carlos, 6627, Campus Pampulha, CEP, 31270-901 Belo Horizonte, Minas Gerais, Brazil; ‡ Department of Science and Technology, 67658 Universidade Estadual do Sudoeste da Bahia, Av. José Moreira Sobrinho, s/n, CEP, 45208-091 Jequié, Bahia, Brazil

## Abstract

In this study, the phytochemical profile of *Conchocarpus mastigophorus* was investigated, leading to the isolation of four pyranoquinolinone alkaloids, including huajiaosimuline (**1**) and three new compounds: 3′-acetoxy-4′-hydroxyzanthosimuline (**2**), epoxyzanthosimuline (**3**), and mastigophorine (**4**). All new compounds possess two chiral centers and exist as a 1:1 mixture of epimers, differing in the configuration of a chiral center at the pyran ring. The absolute configurations of the epimers **2** and **3** were determined by data comparison with identical synthetic compounds, revealing mixtures of (2*S*,3'*R*)*-* and (2*R*,3'*R*)-configured epimers in both cases. By employing Sharpless asymmetric dihydroxylation and Shi epoxidation as key stereoselective steps, stereocontrol at C3 was achieved in the synthesis of compounds **2** and **3**, respectively. Our synthesis approach provided 3*'R*- and 3*'S*-configured **2** in three steps (41–46% overall yield, 95% dr) and 3*'R*-configured **3** in 2 steps (61% overall yield, 90% dr) from 4-hydroxy-1-methyl-2­(1*H*)-quinolinone and citral.

## Graphical abstract

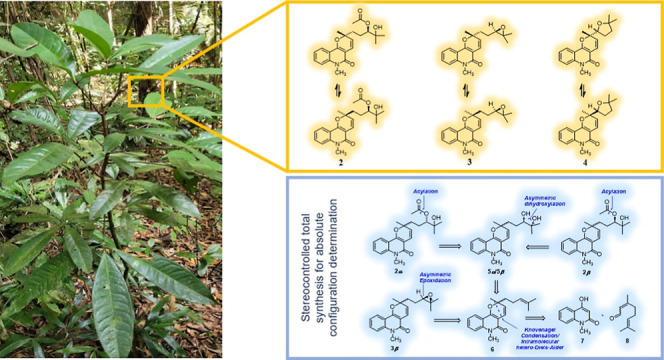

Natural products (NPs) have played a pivotal role in the discovery and development of pharmaceuticals and agrochemicals^1–3^ and account directly or indirectly for nearly 35% of marketed drugs and up to 80% of all antibiotics and anticancer agents.^2^ Common structural features encountered in NPs such as high degree of sp^3^-carbons and chirality have been associated with a higher probability of clinical approval.^4,5^ Because of their often complex structures, unequivocal structural elucidation of NPs may require a combination of methods such as NMR, density functional theory (DFT), X-ray crystallography^6^ and organic synthesis,^7,8^ the latter not only confirming structural identity but also enabling access to sufficient material for pharmacological studies and preparation of derivatives for drug discovery and development purposes.

Brazil is one of the world’s most biodiverse countries and is estimated to hold 40,000 to 50,000 species of higher plants,^9^ being a strategic source of new bioactive compounds. *Conchocarpus* J. C. Mikan, the most diverse genus of the Rutaceae family, comprises about 50 species distributed mainly in tropical and subtropical regions of the Americas,^10^ of which 92% species are found in Brazil, where 66% are endemic.^10,11^ In addition to its taxonomic diversity, this genus exhibits a rich phytochemical profile, including specialized metabolites such as alkaloids, coumarins, flavonoids, among others.^12,13^ In particular, its quinoline and acridone alkaloids exhibit a range of bioactivities, including leishmanicidal, antitumor, and antimicrobial,^14–16^ thereby attracting considerable interest due to their high pharmacological potential.

Previous studies by our group on the phytochemical profile of *Conchocarpus mastigophorus* led to the isolation and identification of 11 compounds,^12^ including two new NPs: the acridone alkaloid 1,3,6-trihydroxy-2,4,5-trimethoxy-10-methylacridin-9­(10*H*)-one methylacridin and the lactam 3-hydroxy-1-methylpiperidin-2-one, whose structures were elucidated by NMR spectroscopy supported by DFT calculations. The other isolated compounds were identified as the quinoline 4-methoxy-1-methylquinolin-2­(1*H*)-one,^17^ the acridone alkaloids citramine,^18^ citrusinine I,^19^ glyfoline,^20^ and citbrasine,^19^ as well as marmesin,^21^ gamma-fagarine,^22^ haplotusine,^23^ and 1-methyl-2-phenylquinolin-2­(1*H*)-one,^24^ the latter four being NPs that had not been previously isolated from the genus *Conchocarpus*.

Biological activities have been reported for several of these NPs. The furanocoumarin marmesin inhibits tyrosinase, and exhibits anti-inflammatory and antiplasmodial activity;^25–28^ γ-fagarine shows antiviral activity^29,30^ and also displays antimicrobial^31–34^ and anticancer effects;^35^ 1-methyl-2-phenylquinolin-4­(1*H*)-one exhibits cytotoxic,^36^ trypanocidal,^37^ and antiplatelet activities;^38^ haplotusine shows moderate trypanocidal activity;^37^ 4-methoxy-1-methylquinolin-2­(1*H*)-one exhibits anti-inflammatory activity,^39^ moderate cytotoxicity,^40,41^ and butyrylcholinesterase inhibition;^42^ citrusine I displays antiviral,^43^ antiallergic,^44^ antioxidant,^45^ and cytotoxic activities^46^ and inhibits acetylcholinesterase^47^ and cathepsin V;^48^ glyfoline shows potent antitumor activity against solid tumors;^49–51^ and citbrasine inhibits cathepsin V^48^ and exhibits antiplasmodial^52^ and algicidal activities.^53^

Herein, we report our most recent advances in the phytochemical studies of *C. mastigophorus*, which resulted in the isolation of huajiaosimuline (**1**), a previously reported pyranoquinolinone alkaloid,^54^ along with three new pyranoquinolinones: (2*R*,3′*R*)-3′-acetoxy-4′-hydroxyzanthosimuline/(2*S*,3′*R*)-3′-acetoxy-4′-hydroxyzanthosimuline (**2**), (2*R,*3′*R*)-epoxyzanthosimuline/(2*S,* 3′*R*)-epoxyzanthosimuline (**3**), and mastigophorine (**4)**. We also describe the complete structural elucidation of the newly discovered compounds (**2**–**4**), which were identified as mixtures of epimers differing in the configuration of a stereolabile chiral center at the pyran ring. To confirm their structures, the NPs were synthesized, and the absolute configurations of the stereostable chiral centers in compounds **2** and **3** were established by high-performance liquid chromatography (HPLC) analysis of stereoisomers obtained after a stereocontrolled total synthesis.
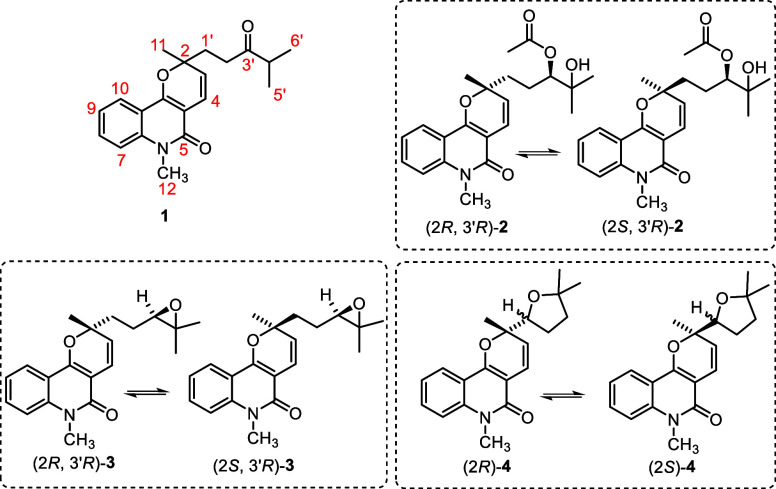


## Results and Discussion

Hexane and EtOAc extracts from the leaves of *C. mastigophorus* were subjected to successive purifications steps through silica-gel column chromatography (CC), followed by semipreparative HPLC using a C18 stationary phase, leading to the isolation of the pyranoquinolinones **1**–**4**. Compound **1** (7.2 mg) was obtained from the hexane extract, whereas the EtOAc extract yielded the new compounds **2** (360 mg), **3** (51 mg) and **4** (2 mg), each obtained as an equimolar mixture of two epimers.

Comparison of the NMR data obtained for compound **1** (Figures S1–S4) with literature data^54^ led to its identification as huajiaosimuline, a previously reported pyranoquinolinone-type alkaloid first isolated in 1993 from roots of *Zanthoxylum simulans* (Rutaceae).^54^

Compound **2** was isolated as a brownish oil. Its molecular formula was suggested to be C_22_H_27_NO_5_ based on HR-ESI-MS data (*m*/*z* 408.1428 [M + Na]^+^; calculated for C_22_H_27_NO_5_Na, *m*/*z* 408.1787). ^1^H and ^13^C NMR data (Table 1) suggested **2** to present the same pyranoquinoline nucleus featured by **1**, differing from it only in the side chain on C2. The occurrence of a common pyranoquinoline-type scaffold in **1** and **2** was further supported by MS data, which showed a similar fragmentation pattern in both compounds, including an *m*/*z* 226 ion, being observed as a base peak, that was assigned to a fragment resulting from C2–C1′ bond cleavage. The structure of the side chain at C2 was established based on ^1^H and ^13^C NMR data (Table 1), which indicated the presence of two carbon atoms bonded to heteroatoms (δ_C_ 79.9, C3′; δ_C_ 72.6, C4′), a carbonyl group (δ_C_ 171.4, C7′), and three methyl groups (δ_H_ 2.12, s, 3H, H8’; δ_H_ 1.17, s, 3H, H5′ and δ_H_ 1.16, s, 3H, H6′). HSQC correlations between Ha1’ (δ_H_ 1.92–1.78, m, 1H) and Hb1’ (δ_H_ 1.77–1.66, m, 1H) with C1′, as well as between Ha2’ (δ_H_ 1.92–1.78, m, 1H) and Hb2’ (δ_H_ 1.77–1.66, m, 1H) with C2’ (Figure 1)­ revealed two sets of diastereotopic methylene groups. A –CH_2_(1′)–CH_2_(2′)–CH­(3′)- spin system was identified based on COSY correlations between H1′ and H2′, and between H2′ and H3′ (Figure 1). The IR spectrum showed absorption bands at 1732 cm^–1^ and 3422 cm^–1^, indicating an ester and a hydroxyl group, respectively. These data, along with the HMBC correlation between methyl H8′ and carbonyl C7’ (Figure 1), provided evidence for an acetoxy moiety. The position of this ester group was assigned to C3′ based on a key HMBC correlation between H3′ and C7′ (Figure 1), while the hydroxyl group was assigned to the remaining heteroatom-bound carbon C4′.

**Table 1:** ^1^H (400 MHz) and ^13^C (100 MHz) NMR Data (CDCl_3_) of Compound **2** ^a^

position	**2a**		**2b**	
	δ_C_, type	δ_H_, mult (*J* in Hz)	δ_C_, type	δ_H_, mult (*J* in Hz)
2	81.3, C		81.1, C	
3	119.1, CH	5.47, d (10.0)	119.0, CH	5.44, d (10.0)
4	125.1, CH	6.80, d (10.0)	124.9, CH	6.80, d (10.0)
4a	105.7, C		105.7, C	
5	161.1, C		161.1, C	
6a	139.6, C		139.5, C	
7	114.2, CH	7.32, dbr (8.5)	114.2, CH	7.32, dbr (8.5)
8	131.1, CH	7.55 ddd (8.5, 6.5, 1.6)	131.1, CH	7.55 ddd (8.5, 6.5, 1.6)
9	121.9, CH	7.23, ddd (8.1)	121.9, CH	7.23, ddd (8.1)
10	123.1, CH	7.94, dd (8.1, 1.6)	123.1, CH	7.92, dd (8.1, 1.6)
10a	116.0, C		116.0, C	
10b	155.4, C		155.3, C	
11	27.3, CH_3_	1.46, s	27.1, CH_3_	1.45, s
12	29.4, CH_3_	3.69, s	29.4, CH_3_	3.69, s
1′-Ha/Hb	38.1, CH_2_	1.92–1.78, m/1.77–1.66, m	38.1, CH_2_	1.92–1.78,m/1.77–1.66, m
2′-Ha/Hb	24.3, CH_2_	1.92–1.78, m/1.77–1.66, m	24.0, CH_2_	1.92–1.78,m/1.77–1.66, m
3′	79.9, CH	4.86–4.77, m	79.9, CH	4.86–4.77, m
4′	72.6, C		72.6, C	
5′	25.3, CH_3_	1.17, s	25.2, CH_3_	1.17, s
6′	26.8, CH_3_	1.16, s	26.8, CH_3_	1.15, s
7′	171.4, C		171.3, C	
8′	21.2, CH_3_	2.12, s	21.2, CH_3_	2.09, s

a Compound **2** corresponds to a mixture of epimers **2a** and **2b**.

**Figure 1:**
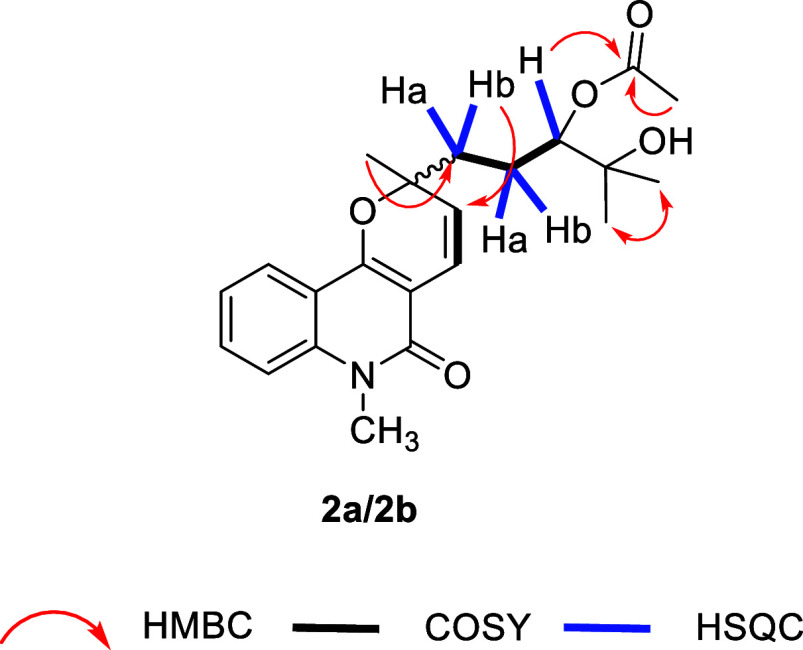
Key HMBC, COSY, and HSQC correlations for compound **2**.

Compound **2** was identified as a 1:1 mixture of epimers, based on the observation of duplicated signals assigned to H-3, H-11, H5′, H6′ and H8′ presenting similar intensity on ^1^H NMR spectrum. The corresponding carbon signals also showed duplication in the ^13^C NMR spectrum. The presence of an epimeric mixture was further supported by HR-MS data, which indicated a single molecular formula and is consistent with the proposed structure containing two stereogenic centers (C2 and C3′). This hypothesis was corroborated by HPLC analysis using both C18 and a chiral stationary phase (Figures S93 and S96), which revealed two chromatographic peaks with similar relative areas in both cases. Attempts to separate the epimers by semipreparative HPLC suggested that epimerization occurs spontaneously. An HPLC analysis of each freshly isolated stereoisomer performed 1 h after chromatography separation showed a small amount of the minor epimer. HPLC chromatogram and ^1^H and ^13^C NMR spectra obtained from one of the samples several days after separation, however, revealed that the original 1:1 diastereomeric mixture was regenerated, thus indicating that the single stereomer obtained by semipreparative HPLC underwent epimerization. A plausible explanation for this stereolability relies on the reversible opening and closing of the pyran ring at C2 via an oxa-Diels–Alder-type reaction.^55–60^ The reversibility of this reaction and a consequent dynamic equilibrium between 2*H*-pyrans and their open form oxatriene (a dienone or dienal) has been reported,^57^ including studies on 2*H*-pyran quinones,^59^ benzopyrans,^61^ and pyranopyridones.^62^ Interestingly, Porco and co-workers^59^ synthesized the fungal NP (+)-torreyanic acid through [2 + 4] dimerization of a 1:1 epymeric mixture of 2*H*-pyran quinone epoxides. Investigations on thermolysis of the dimer, together with computational studies, indicated the equilibration of the 2*H*-pyran epimers via “oxa-Diels–Alder-type” reaction. The oxidation of a hydroxyl precursor to give an oxatriene, followed by electrocyclization to the epymeric 2*H*-pyran quinone epoxides and their dimerization through cycloaddition, has been proposed as a plausible biosynthetic route to (+)-torreyanic acid^59,63^ and other fungal metabolites.^57^

The 2*H*-pyran (closed form) has been reported to be favored in equilibrium over the oxatriene (open form) by steric features destabilizing the oxatriene and the presence of electron-withdrawing substituents at the α-carbon of the dienone or dienal (oxatriene), which corresponds to the C-5 in a 2*H*-pyran ring.^60^

The intramolecular equilibrium between the open and closed forms of compound **2** was investigated by DFT calculations. The calculated Gibbs free energy difference (Δ*G*° = +9.5 kcal/mol) at 25 °C (298 K) and the corresponding equilibrium constant (*K* = 1.1 × 10^7^) indicate a strong thermodynamic preference for the closed form (Figure 2), showing that it predominates almost exclusively in solution at room temperature. This finding is consistent with experimental observations in which only the closed isomer was detected and the open form was not observed in ^1^H and ^13^C NMR experiments.

**Figure 2:**
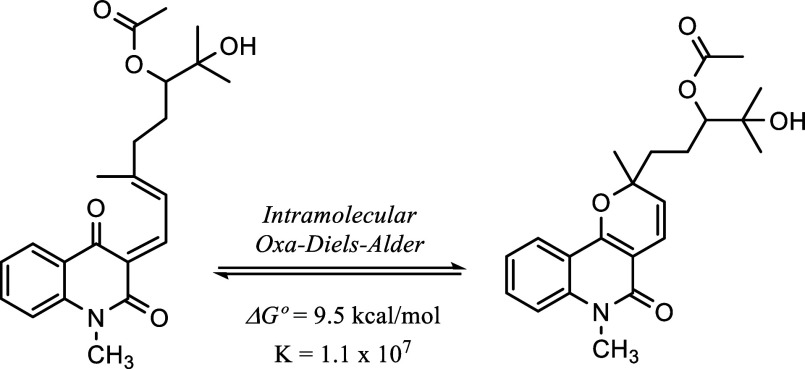
Intramolecular equilibrium between open and closed forms of compound **2** via a reversible oxa-Diels–Alder reaction.

Despite its thermodynamic instability, the open form may play an important role in the dynamic behavior of the pyranoquinolinone scaffold studied herein. Particularly, the observed epimerization of the closed isomer suggests the occurrence of a reversible ring-opening process, possibly mediated by the transient population of a flexible open intermediate.

The data outlined above led to the identification of compound **2** as a mixture of epimers (herein referred to as epimers **2a** and **2b**), corresponding to the structures shown in Figure 1, and named 3′-acetoxy-4′-hydroxyzanthosimuline. The absolute configuration at C3′ was further determined by comparison with the chromatographic data of (3′*R*)**-2** and (3′*S*)-**2**, obtained via asymmetric synthesis, as described in the following section.

Compound **3** was obtained as a light-yellow oil, and its molecular formula was established as C_20_H_23_NO_3_ by HR-ESI-MS data (*m*/*z* 326.1756 [M + H]^+^; calculated *m*/*z* 326.1751 for C_20_H_24_NO_3_). The ^1^H e ^13^C NMR data of **3** (Table 2) were similar to those of **2**, except for differences in the chemical shifts of signals associated with H3′(δ_H_ 4.86–4.77 in **2**; δ_H_ 2.71 in **3**), C3′ (δ_C_ 58.6), and C4’ (δ_C_ 64.2), as well as for the absence of signals related to an acetoxy group (δ_H_ 2.12, s, 3H – H8’/δ_C_ 21.2 – C8’; δ_C_ 171.4 – C7′). These findings suggest that **3** shares the same pyranoquinolinone core featured by **2** but contains a substituent different from that at C2. COSY correlations (Figure 2) revealed a –CH_2_(1′)–CH_2_(2′)–CH­(3′)- spin system in the C-2 side chain. Additionally, the chemical shifts for C3′ (δ_C_ 64.2) and C4’ (δ_C_ 58.6), combined with the molecular formula, suggested a C3′–O–C4′bond, leading to the identification of a terminal 2,2-dimethyloxirane moiety. These structural assignments were supported by HMBC correlations (Figure 3) between the methyl hydrogens H5′ (δ_H_ 1.23) and H6’ (δ_H_ 1.19) and the oxygenated carbons C4′ and C3′; between H3′ and C2′ and C1’; and between H1′ and C2 e C3. As previously discussed for **2**, duplicated signals were also observed in the ^1^H and ^13^C NMR spectra of **3**, and a 1:1 epimeric mixture was revealed by HPLC analysis, consistent with a diastereomeric mixture presumably resulting from the epimerization of C2. These data led to the identification of compound **3** as an epimeric mixture (here referred to as epimers **3a** and **3b**), which was named as epoxyzanthosimuline.

**Table 2:** ^1^H (400 MHz) and ^13^C (100 MHz) NMR Data (CDCl_3_) for Compound **3** ^a^

position	**3a**		**3b**	
	δ_C,_ type	δ_H_, mult (*J* in Hz)	δ_C,_ type	δ_H_, mult (*J* in Hz)
2	80.9, C		81.2, C	
3	124.8, CH	5.46, d (10.0)	125.1, CH	5.47, d (10.0)
4	118.9, CH	6.79, d (10.0)	119.0, CH	6.79, d (10.0)
4a	105.5, C		105.6, C	
5	161.1, C		161.1, C	
6a	139.5, C		139.5, C	
7	114.2, CH	7.30, dbr (8.5)	114.2, CH	7.30, dbr (8.5)
8	131.1, CH	7.53, ddd (8.5, 7.1, 1.6)	131.1, CH	7.53, ddd (8.6, 7.1, 1.6)
9	121.9, CH	7.21, ddd (8.3, 7.1, 1.0)	121.9, CH	7.21, ddd (8.3, 7.1, 1.0)
10	123.1, CH	7.92, dd (8.3, 1.6)	123.1, CH	7.92, dd (8.3, 1.6)
10a	115.9, C		115.9, C	
10b	155.4, C		155.4, C	
11	27.0, CH_3_	1.48, s	27.3, CH_3_	1.48, s
12	29.4, CH_3_	3.67, s	29.4, CH_3_	3.67, s
1′-Ha/Hb	38.2, CH_2_	2.04–1.97, m/1.85–1.78, m	38.3, CH_2_	1.95–1.90, m
2′- Ha/Hb	23.6, CH_2_	1.74–1.65, m	23.9, CH_2_	1.74–1.65, m
3′	64.2, CH	2.71, t (6.5)	64.2, CH	2.72, t (6.5)
4′	58.6, C		58.7, C	
5′	24.9, CH_3_	1.23, s	24.9, CH_3_	1.26, s
6′	18.7, CH_3_	1.19, s	18.7, CH_3_	1.21, s

a Compound **3** corresponds to a mixture of epimers **3a** and **3b**.

**Figure 3:**
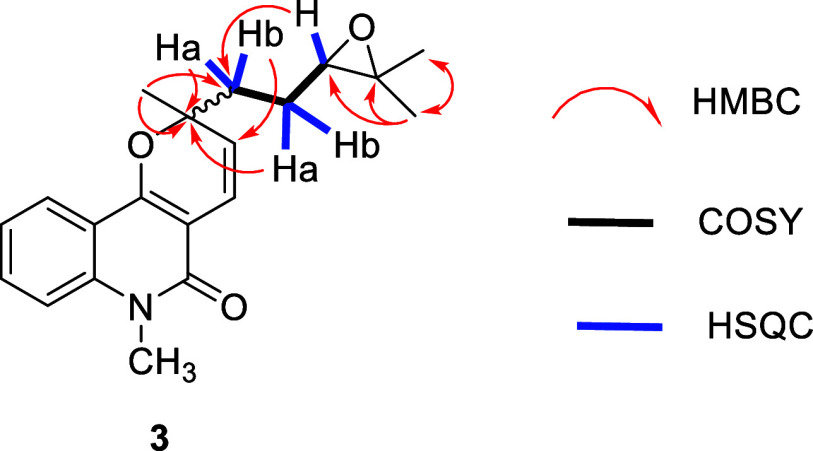
Key HMBC, COSY, and HSQC correlations for compound **3**.

Compound **4**, obtained as a brown oil, had its molecular formula established as C_20_H_23_NO_3_ by HR-ESI-MS data (*m*/*z* 348.1092 [M + Na]^+^; calculated *m*/*z* 348.1576 for C_20_H_23_NO_3_Na), thus being an isomer of compound **3**. The ^1^H and ^13^C NMR data for **4** (Table 3) were also similar to those of **3**, the key difference being a signal at 3.86–3.80 ppm ascribed to an oxygenated methine hydrogen [δ_H_ 3.86–3.80 (m, 1H; –CH(1′)-O−) in **4**]. Similarly to **3**, COSY and HSQC correlations (Figure 4) revealed a –CH–CH_2_–CH_2_– spin system with two sets of diastereotopic hydrogens (Ha2′/Hb2′, Ha3′/Hb3′) and suggested the presence of an oxygenated carbon (δ_C_ 74.4—C1′). The molecular formula, combined with HMBC correlations between H5′ and H6′ with C4′ only, and between methine hydrogen (H1′) and C2 (Figure 3), suggested **4** to be an analogue of **3** bearing a five-membered oxa-heterocycle in the place of the oxirane ring. This proposed structure was further supported by additional HMBC correlations between H5′ and H6′ with C3′ (Figure 4), as well as by the observed higher chemical shift of H1′in **4**, which is expected to be more deshielded than in **3** due to the lower ring strain in the five-membered ring compared to the three-membered ring present in **3**.^64^

**Table 3:** ^1^H (400 MHz) and ^13^C (100 MHz) NMR Data (CDCl_3_) of Compounds **4** ^a^

position	**4a**		**4b**	
	δ_C_, type	δ_H_, mult (*J* in Hz)	δ_C_, type	δ_H_, mult (*J* in Hz)
2	81.4, C		81.0, C	
3	125.2, CH	5.49, d (9.9)	124.8, CH	5.47, d (9.9)
4	119.3, CH	6.82, d (9.9)	119.0, CH	6.81, d (9.9)
4a	105.7, C		105.7, C	
5	161.1, C		161.1, C	
6a	136.6, C		139.6, C	
7	114.3, CH	7.32, dbr (8.7)	114.3, CH	7.32, dbr(8.7)
8	131.2, CH	7.55, ddd (8.7, 8.0, 1.6)	131.2, CH	7.55, ddd (8.7, 8.0, 1.6)
9	122.0, CH	7.23, tbr (8.0)	122.0, CH	7.23, tbr(8.0)
10	123.2, CH	7.94, dd (8.0, 1.6)	123.1, CH	7.93, dd (8.0, 1.6)
10a	116.0, C		116.0, C	
10b	155.3, C		155.3, C	
11	27.5, CH_3_	1.49, s	27.0, CH_3_	1.48, s
12	29.8, CH_3_	3.69, s	29.5, CH_3_	3.69, s
1’-	74.4, CH	3.86–3.80, m	74.3, CH	3.86–3.80, m
2′-Ha/Hb	39.7, CH_2_	2.20–2.23, m/1.88–1.69, m	39.6, CH_2_	2.20–2.23, m/1.88–1.69, m
3′- Ha/Hb	28.3, CH_2_	2.20–2.23, m/1.88–1.69, m	28.3, CH_2_	2.20–2.23, m/1.88–1.69, m
4′	73.0, C		73.0, C	
5′	25.5, CH_3_	1.25, s	25.4, CH_3_	1.25, s
6′	26.7, CH_3_	1.28, s	26.5, CH_3_	1.28, s

a Compound **4** corresponds to a mixture of epimers **4a** and **4b**.

**Figure 4:**
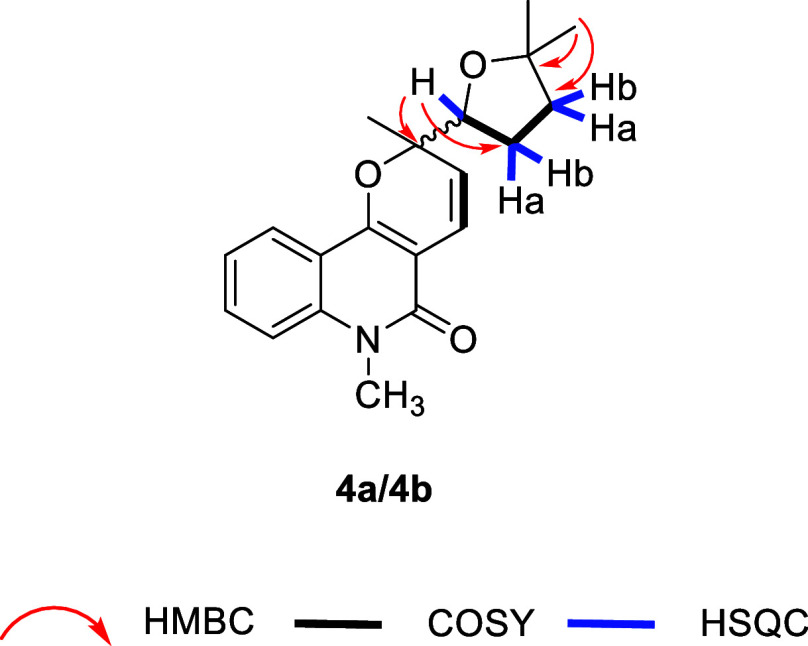
Key HMBC, COSY, and HSQC correlations of compound **4**.

Similar to the previously discussed compounds **2** and **3**, compound **4** was also found to occur as a 1:1 epimeric mixture, as indicated by duplicated ^1^H NMR signals assigned to H3, H4, H5′ and H6′, as well as by HPLC analysis. Determination of the absolute configuration of the stereostable chiral center C1′, however, was not achieved. Compound **4** could not be obtained diastereoselectively via asymmetric synthesis for data comparison, and the limited amount isolated from the natural source (2.0 mg) was insufficient for absolute configuration assignment by other methods. Nevertheless, the spectroscopic and chromatographic data presented herein allowed **4** to be identified as a mixture of epimers, for which we propose the name mastigophorine.

### Stereoselective Synthesis of Pyranoquinolinones and Determination of the Absolute Configuration of NPs **2** and **3**

Compounds **2**, **3**, and **4**, isolated from *C. mastigophorus*, were identified as pyroquinolinones bearing two chiral centers. All were found to occur as mixtures of epimers differing in the configuration of the chiral center at C2 in the pyran ring. This feature could make an unambiguous determination of the absolute configuration of the stereostable chiral center—at C3′ in **2** and **3,** and at C1′ in **4**—challenging using conventional methods such as electronic circular dichroism (ECD) and vibrational circular dichroism (VCD). In the case of ECD, the chirality center associated with the chromophore is racemic, which is expected to result in a null experimental spectrum. Although VCD has been reported as a tool for the determination of the absolute configuration of a mixture of chromane epimers,^65^ the analysis took into account experimental VCD data from similar compounds whose stereochemical configuration had been assigned. In addition, VCD may not be able to discriminate against diastereomers in some cases, so that a combination of complementary chiroptical methods,^66–68^ such as Raman optical activity and optical rotatory dispersion, may be required for unambiguous stereochemical assignment. Organic synthesis was then chosen for the determination of absolute configuration, since it is a powerful approach for structure elucidation^7,8^ and can also provide access to material for future pharmacological studies. Therefore, an asymmetric synthesis of **2** and **3** with stereocontrol at C3′ was undertaken to enable the determination of the absolute configuration of the naturally occurring epimers isolated from *C. mastigophorus*. In this study, each pair of synthesized epimers is referred to as α or β, according to the absolute configuration *S* or *R*, respectively, at C3′.

The retrosynthetic analysis depicted in Scheme 1 identified zanthosimuline (**6**) as a key precursor for pyranoquinolinones **2** and **3**. Zanthosimuline (**6**) is an NP originally isolated from the root bark of *Z. simulans*,^54^ whose synthesis has already been reported.^69–71^ Both compounds (3′*S*)-**2α** and (3′*R*)-**2β** were obtained via regioselective acylation of the corresponding diols **5α** and **5β**, which were prepared as the enantiomerically pure (3′*S*)-**5α** and (3′*R*)-**5β** intermediates through a C3′-stereocontrolled Sharpless asymmetric dihydroxylation^72^ of **6** as the key step, using AD-mix-α or AD-mix-β, respectively. Zanthosimuline (**6**), which can be synthesized from **7** and **8**, also provided access to compound (3′*R*)-**3α** via a stereoselective Shi asymmetric epoxidation.^73^

**Scheme 1:**
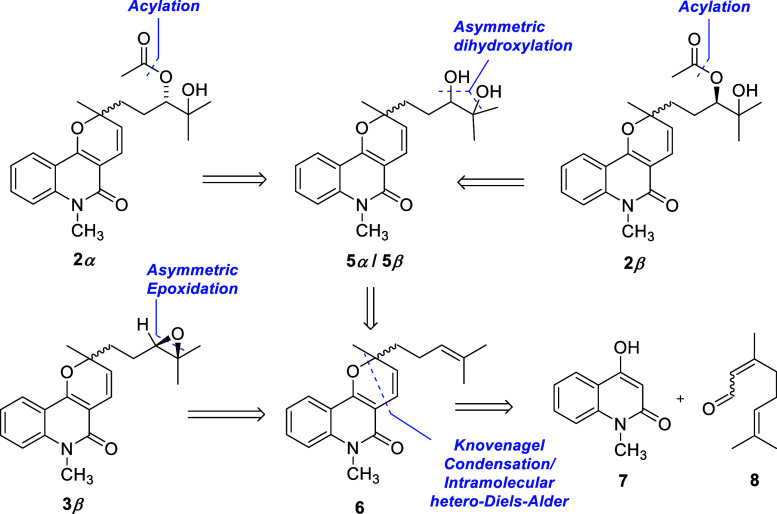
Retrosynthetic Analysis of Compounds **2**α, **2**β, and **3**β; Key Disconnections and Proposed Precursors Are Shown

Based on the retrosynthesis shown in Scheme 1, zanthosimuline (**6**) was obtained from 4-hydroxy-1-methyl-2­(1*H*)-quinolinone (**7**) and citral (**8**) via a Knoevenagel condensation followed by an intramolecular hetero-Diels–Alder reaction. Using the procedure reported by Neve and co-workers,^71^ zanthosimuline (**6**) was prepared from **7** and **8** in 85% yield in a single step (Scheme 2). The formation of **6** was confirmed by mass spectrometry, which showed a molecular ion consistent with the formula C_20_H_23_NO_2_ (*m*/*z* 309), and by NMR spectroscopy. The ^1^H NMR spectrum displayed signals at δ_H_ 1.48, δ_H_ 1.55 and δ_H_ 1.62, corresponding to the methyl hydrogens at C2 and C4′, and a signal at δ_H_ 3.68 assigned to hydrogens at *N*-methyl group. Signals attributed to aromatic hydrogens were observed between δ_H_ 7.17 and 8.00, along with vicinal hydrogens H3 and H4 (δ_H_ 5.48 and δ_H_ 6.80, respectively), corresponding to the pyrane ring. All spectral data were in agreement with those previously reported for zanthosimuline (**6**).^70^

**Scheme 2:**
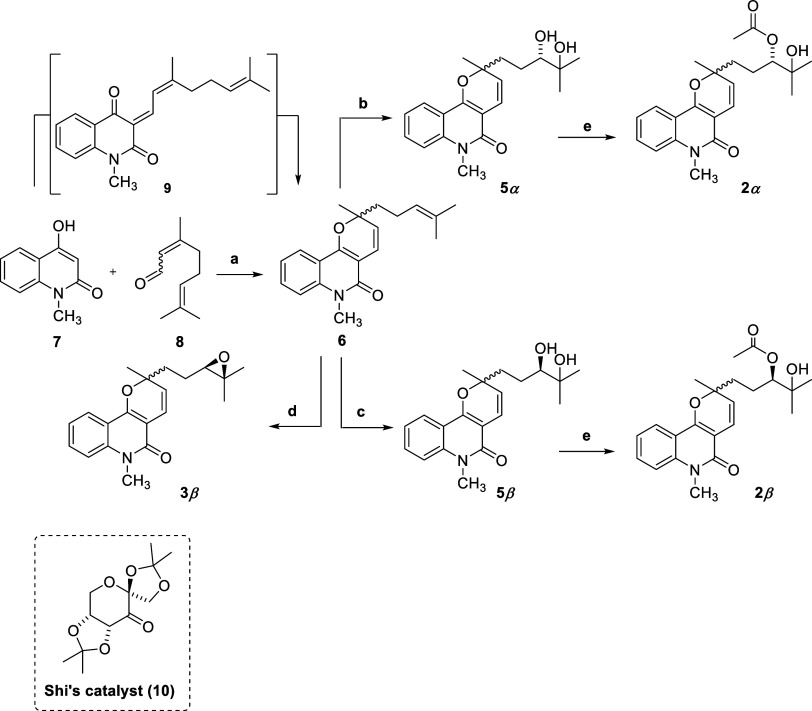
Stereoselective Synthesis of Natural Pyranoquinolinone Alkaloids

a Reaction conditions: (a) MgSO_4_, Py, Reflux, 18 h, 85%; (b) AD-mix-α (0.18 mol %), MeSO_2_NHCH_3_ (1 equiv), *t*-BuOH-H_2_O (1:1), 0 °C, 48 h, 68% (95% de); (c) AD-mix-β (0.18 mol %), MeSO_2_NHCH_3_ (1 equiv), *t*-BuOH-H_2_O (1:1), 0 °C, 48 h, 60% (96% de); (d) Oxone (1.6 equiv), Shi’s cat. (0.3 equiv), K_2_CO_3_ (5.8 equiv), Buffer, 0 °C, 1.5 h. 72% (92% de); (e) DMAP (0.15 equiv), Ac_2_O (15 equiv), Py, rt, 30 min, 80% for **2**α and **2**β

As a key step in the synthesis of compounds (3′*S*)-**2α** and (3′*R*)-**2β**, compound **6** was subjected to a Sharpless asymmetric dihydroxylation under the conditions described by Kanojia and co-workers.^74^ This reaction was performed using either AD-mix-α or AD-mix-β, affording (3′*S*)-**5α** in 68% yield with 95% diastereomeric excess, and (3′*R*)-**5β** in 60% yield with 96% diastereomeric excess, respectively. The absolute configuration at C3′ of each product was assigned according to the well-established predictive model for facial selectivity of AD-mix-α and AD-mix-β, which considers the relative size of the alkene substituents, resulting in predictable enantiopreference according to the chosen catalyst.^72^ Finally, treatment of (3′*S*)-**5α** and (3′*R*)-**5β** with acetic anhydride in the presence of DMAP furnished compounds (3′*S*)-**2α** and (3′*S*)-**2β**, both in 80% yield.

Evidence for the formation of the intermediate diols (3′*S*)-**5α** and (3′*R*)-**5β** was provided by IR absorptions at 3389 cm^–1^ and 1160 cm^–1^, consistent with the presence of a hydroxyl group and a C–O bond. In addition, the ^1^H and ^13^C NMR spectra showed signals at δ_H_ 3.37 and δ_C_ 73.2 and δ_C_ 78.7, which were assigned to H3′, and the oxygenated carbons C3′ and C4′, respectively. Furthermore, HSQC correlations between two hydrogens at δ_H_ 1.39–1.58 (m, 1H) and δ_H_ 1.60–1.74 (m, 1H) and a carbon at δ_C_ 26.3 (C2′) (Figure 5), as well as between the signals at δ_H_ 2.23–2.00 (m, 1H) and δ_H_ 1.93–1.69 (m, 1H) with a carbon at δ_C_ 38.9 (C1′), revealed the presence of two diastereotopic hydrogens at C1′ and at C2′, thus supporting the formation of the newly created chiral center at C3′. Similar to the other pyranoquinolinones discussed herein, duplicated signals in the ^1^H NMR spectrum for H3, H4′, and H7′ suggested that compounds (2*S*, 3′*S*)-**5α/**(2*R*, 3′*S*)-**5α**, and (2*S*, 3′*R*)-**5β/**(2*R*, 3′*R*)-**5β** exist as a 1:1 mixture of epimers, which was further confirmed by chiral phase HPLC analyses.

**Figure 5:**
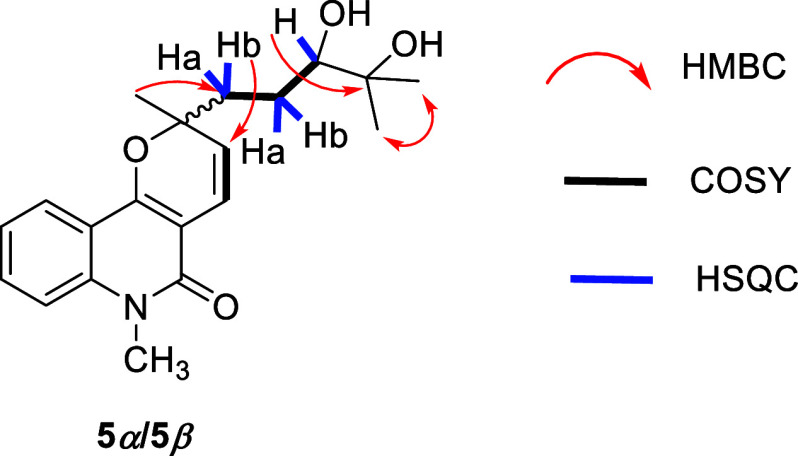
Key HMBC, COSY, and HSQC correlations for compounds **5α/5β**.

The proposed structures for the synthesized compounds (3′*S*)-**2α** and (3′*R*)-**2β** were supported by IR absorption bands at 3403 cm^–1^ and 1728 cm^–1^, which were assigned to hydroxyl and ester carbonyl groups, respectively. These findings, together with the presence of signals at δ_H_ 2.12 (H8′), δ_C_ 21.2 (C8′), and δ_C_ 171.4 (C7′) in the ^1^H and ^13^C NMR spectra, are consistent with monoacylation of the diols (3′*S*)-**5α** and (3′*R*)-**5β**. As expected, the spectroscopic data of the synthetic (3′*S*)-**2α** and (3′*R*)-**2β**, including HMBC correlations, were identical to those of **2** obtained from *C. mastigophorus*, thereby confirming the proposed identity of this NP.

Chiral phase HPLC analysis of compounds (3′*S*)**-5α**, (3′*R*)**-5β**, (3′*S*)**-2α**, and (3′*R*)**-2β** revealed that they were obtained with high stereoselectivity, exhibiting enantiomeric excess greater than 95%. In all cases, the chromatograms displayed two major peaks of equal intensity, accompanied by two minor peaks. The two major peaks were assigned to a 1:1 mixture of epimers sharing the same absolute configuration at C3′, but with opposite configurations at C2. Notably, the chromatogram of compound (3′*S*)-**2α** showed two major peaks (corresponding to 2*R*,3′*S*- and 2*S*,3′*S*-configured stereomers) with retention times distinct from those observed for the major peaks of (3′*R*)-**2β** (corresponding to 2*R*,3′*R*- and 2*S*,3′*R*-configured stereomers), as expected for enantiomeric compounds. The same pattern was observed for the corresponding diols (3′*S*)-**5α** and (3′*R*)-**5β**. Comparison of the chiral phase HPLC chromatogram of NP **2** obtained from *C. mastigophorus* with those of the synthetic (3′*S*)-**2α** (Figure S 94) and (3′*R*)-**2β**, including coelution experiments (Figures 6 and S94), enabled full stereochemical elucidation of the NP. These results, including coelution with (3′*R*)-**2β**, confirmed its identity as a 1:1 mixture of (2*R*,3′*R*)**-2** and (2*S*, 3′*R*)-**2**.

**Figure 6:**
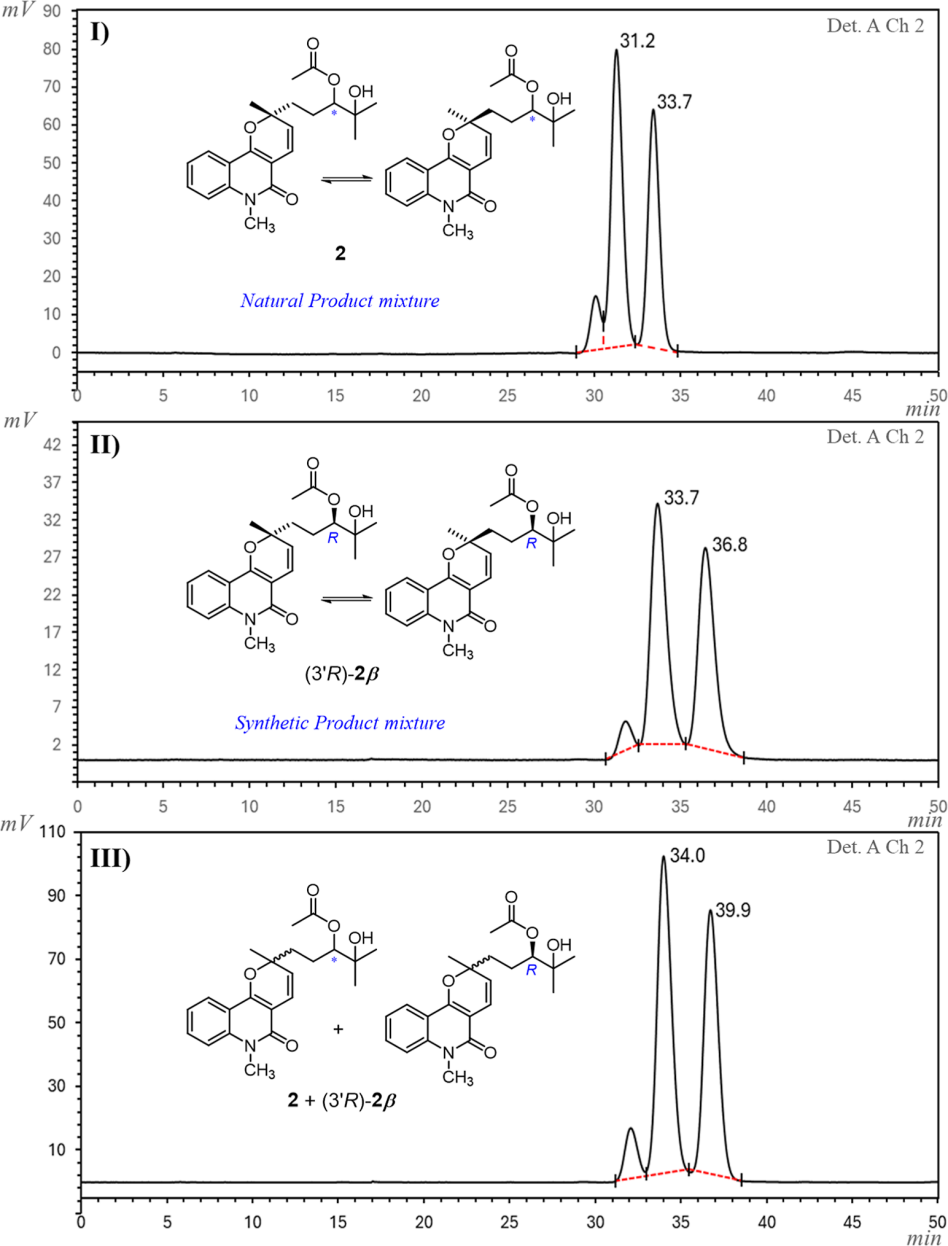
Chiral phase HPLC chromatograms of (I) NP **2**; (II) synthetic (3′*R*)-**2β**; and (III) coelution of NP **2** with synthetic (3′*R*)-**2β**.

To confirm the proposed structure of the NP **3**, the corresponding compound was first synthesized via nonstereoselective epoxidation of **6** with *m-*CPBA, affording a mixture of the four stereomers **3a**–**d** (Figure 7) in 80% yield. The mass spectrum of the synthesized compound showed a molecular ion peak at *m*/*z* 325, consistent with a molecular formula C_20_H_23_NO_3_. Furthermore, the ^1^H and ^13^C NMR data were identical to those of NP **3**, including duplicated ^1^H signals ascribed to the epimers. Chiral phase HPLC analysis confirmed that the synthesized **3a**–**d** consisted of four stereoisomers (Figure S79), as expected from the nonstereolective epoxidation of racemic **6**. To assign the absolute configuration at C3′ in the natural compound **3**, the (3′*R*)-**3β** stereoisomer was synthesized from **6** via a Shi asymmetric epoxidation^73^ employing chiral catalyst **10**.

**Figure 7:**
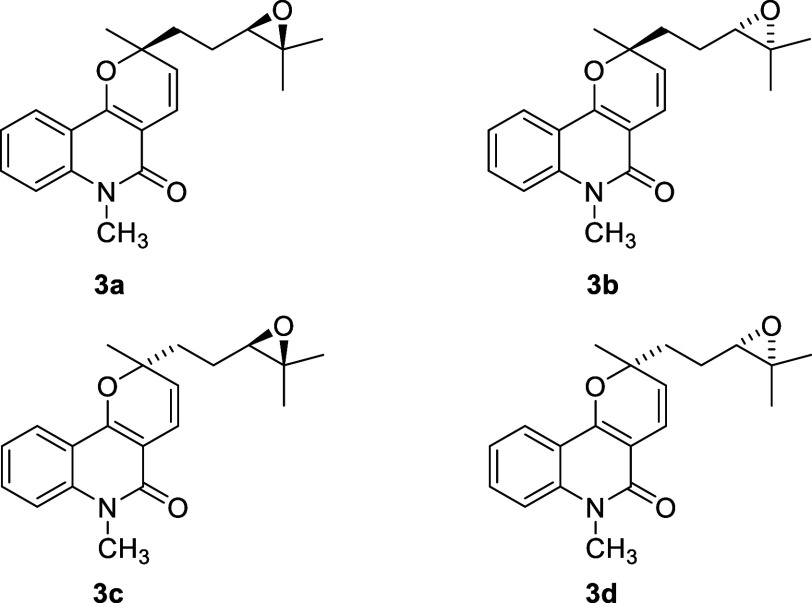
Mixture of stereoisomers obtained by nonstereoselective epoxidation of compound **6** with *m-*CPBA.

The absolute configuration of the resulting epoxide **3β** was deduced from models describing the enantiopreference observed in epoxidations of trisubstituted alkenes catalyzed by the Shi catalyst^73,75–77^ and from the previously observed enantiopreference displayed by this catalyst toward compounds with similar alkene moieties.^78–80^ The reaction proceeded stereoselectively to give compound **3β** in 72% yield and 92% enantiomeric excess (e.e.), as a 1:1 mixture of epimers, presumably bearing the *R* configuration at C3′. Mass spectrometry and NMR data from (3′*R*)-**3β** were identical to those obtained for NP **3**, as expected. The chiral phase HPLC chromatogram of (3′*R*)-**3β** showed two major peaks with similar intensity (Figure 8), thus confirming the stereoselective obtaining of a 1:1 mixture of (2*R*,3′*R*)-**3β** and (2*S*,3′*R*)-**3β** from the racemic mixture (2*R*/2*S*)-**6**. Comparison of the chiral phase HPLC chromatograms of the synthetic (3′*R*)-**3β** and the natural compound **3** (Figure 8) revealed them to be identical, thereby allowing the NP **3**, isolated from *C. mastigophorus*, to be identified as an epimeric mixture of (2*R*,3′*R)-* **3** and (2*S*,3′*R*)-**3**.

**Figure 8:**
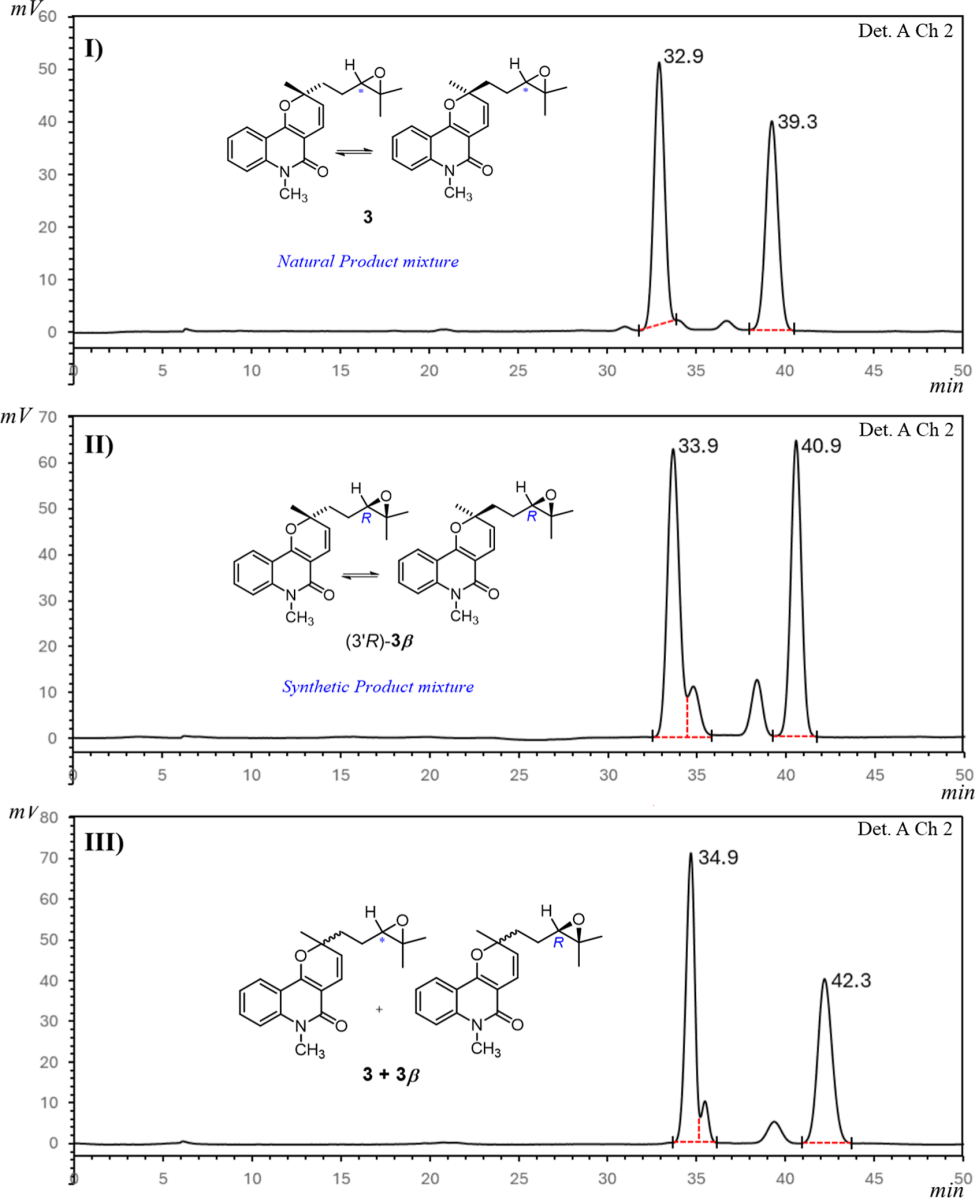
Chiral phase HPLC chromatograms of (I) NP **3**; (II) synthesized (3′*R*)-**3β**; and (III) coinjected NP **3** and synthesized (3′*R*)**-3β**.

## Conclusion

We reported herein, for the first time, the isolation and identification of three new pyranoquinolinone alkaloids from the leaves of *C. mastigophorus*, namely, 3′-acetoxy-4′-hydroxyzanthosimuline (**2**), epoxyzanthosimuline (**3**), and mastigophorine (**4**). Structural elucidation was achieved by analysis of HRMS, ^1^H and ^13^C NMR and chiral phase HPLC data, which fully matched data from identical compounds obtained by synthesis. The new NPs **2**, **3**, and **4** present a chiral center at C2 in the pyran ring and a chiral center at C3′ in the side chain. All of them were demonstrated to occur as a 1:1 mixture of epimers differing in configuration at C2.

The absolute configuration of the chiral center at C3 could be determined for NPs **2** and **3** by comparison of their retention times in chiral phase HPLC with those of the corresponding synthetic stereomers, revealing that **2** comprises a 1:1 mixture of (2*R*,3*'R*)-**2** and (2*S*,3*'R*)-**2,** and **3** is a 1:1 mixture of (2*R*,3*'R*)-**3** and (2*S*,3*'R*)-**3**. The total syntheses of the target compounds relied on the obtaining of the pyranoquinolinone via a Knoevenagel condensation between citral and 4-hydroxy-1-methylquinolin-2­(1*H*)-one as a key precursor, whereas stereocontrol at C3′ was achieved by employing Sharpless asymmetric dihydroxylation and Shi asymmetric epoxidation to provide, respectively, 3*'S*- or 3*'R*-configured **2** in three steps (41–46% overall yield), and 3*'R*-configured **3** in 2 steps (61% overall yield).

In summary, three novel pyranoquinoline alkaloids from *C. mastigophorus* were identified, all of which were shown to exist as a 1:1 mixture of two epimers differing in configuration at C2 in the pyran ring. The absolute configuration of the epimers was determined for two of the three novel alkaloids, revealing that both consist of a mixture of 2*S,*3*'R*- and 2*R,*3*'R*-stereomers. A stereoselective synthesis was developed to yield these two NPs as a 1:1 mixture of 3'*R*-configured epimers. The synthetic access to these compounds may not only facilitate studies on their pharmacological properties but also enable the preparation of analogues and derivatives for medicinal chemistry purposes, particularly considering that they feature reactive sites suitable for further functionalization.

## Experimental Section

### General Experimental Procedures

Optical rotations were determined in MeOH at 25 °C by using a Bellingham Stanley ADP220 polarimeter (Bellingham Stanley Ltd., Tunbridge Wells, United Kingdom). UV–vis spectra (200–500 nm) were recorded in CH_2_Cl_2_ at 25 °C using a Shimadzu UV-1900i spectrophotometer (Shimadzu Corporation, Kyoto, Japan). FTIR spectra were recorded on a Shimadzu IRSprit spectrometer equipped with a Gladi ATR accessory (4000–400 cm^–1^) (Shimadzu Corporation, Kyoto, Japan). NMR spectra were recorded at rt on a Bruker spectrometer (400 or 600 MHz for ^1^H, 100 or 150 MHz for ^13^C). Chemical shifts were reported in ppm, and the ^1^H NMR and ^13^C NMR spectra were referenced to the residual solvent signals of CDCl_3_ (δ_H_ 7.26, δ_C_ 77.01) as internal references. For diastereomeric mixtures, duplicated signals are reported separately, indicated by “/” followed by the corresponding nucleus [e.g., δ_C_ 125.7/125.4 (C3)]. HR-ESI-MS data were acquired on a Shimadzu ion trap-time-of-flight (Shimadzu Corporation, Kyoto, Japan) and a Bruker MicroToF (Bruker Daltonics, Billerica, MA, EUA) mass spectrometer by direct infusion. Thin-layer chromatography (TLC) was performed on glass-supported silica gel plates (0.50 mm, GF254) and aluminum-backed plates (0.20 mm, Polygram-UV254; Macherey-Nagel). TLC spots were visualized under UV irradiation (254 and 365 nm) or with Dragendorff’s reagent. CC was carried out using silica gel 60 (0.063–0.200 nm; Macherey-Nagel) and Sephadex LH-20 (Sigma-Aldrich). HPLC was performed on a Shimadzu instrument equipped with two LC-6AD pumps, a UV detector (SPD-20A) operating at two wavelengths (254 and 365 nm), and a fraction collector. Analyses were conducted using an Ascentis C_18_ column (25 cm × 21.2 mm, 5 μm) and chiral Astec Cellulose DMP column (25 cm × 4.6 mm, 5 μm - Supelco). Deuterated solvents and reagents were used as received from commercial suppliers.

### Plant Material

The plant material of *C. mastigophorus* was collected at the farm “Samaritana I” (S13°43′10″ W039°37′48″), Itamarí, Bahia (Brazil). A voucher (n° HUESB 14647) is deposited in the Herbarium of Universidade Estadual do Sudoeste da Bahia (HUESB). Authors obtained authorization of access to the Brazilian System for the Management of Genetic Heritage and Associated Traditional Knowledge, SISGEN AE66B71.

### Extraction and Isolation

The dried and powered leaves of *C. mastigophorus* (530 g) were sequentially extracted with hexane (4 L) and EtOAc (4 L). The resulting filtrates were concentrated to dryness in a rotary evaporator under reduced pressure, yielding the hexane (HL, 21.6 g) and EtOAc (EAL, 14 g) extracts from leaves. The hexane extract was fractionated by CC, on silica gel 60, employing a hexane-EtOAc solvent system with a gradient of increasing polarity (from 90:10 to 0:100, v/v) as the eluent. Fractions were monitored by TLC and combined into five groups (HL-1 to HL-5). Fraction HL-4 was further purified by CC on silica gel 60 using a hexane–dichloromethane (CH_2_Cl_2_)-EtOAc mixture (3:1:1 v/v) as the eluent, affording five subfractions (HL-4.1 to HL-4.5) based on TLC analysis. Subfraction HL-4.1 was filtered through Celite S, yielding compound **4** (2.0 mg). Subfraction HL-4.3 was purified by CC on silica gel 60, eluted with hexane-CH_2_Cl_2_ (1:1), affording compound **3** (51 mg).

The EAL extract was also fractionated by CC on silica gel 60 using a hexane-EtOAc solvent gradient (from 80:20 to 0:100, v/v). TLC-guided fractionation afforded four main fractions (EAL −1 to EAL −4). Fraction EAL-4 (1.0 g) was subjected to further purification by CC on silica gel 60, using hexane-EtOAc (1:3, v/v) as the eluent. TLC analysis of the eluates resulted in three pooled fractions (EAL-4.1 to EAL-4.3). Fraction EAL-4.1 (68.7 mg) was further chromatographed on silica gel 60 using a hexane-CH_2_Cl_2_–MeOH mixture (6:14:1, v/v), yielding four subfractions (EAL-4.1.1 to EAL-4.1.4). Subfraction EAL-4.1.2 was purified by silica gel using hexane- CH_2_Cl_2_–MeOH mixture (5:4:0.7), affording compound **1** (7.2 mg). Fraction EAL-4.2 (939.5 mg) was subjected to CC on Sephadex LH-20, using hexane-CH_2_Cl_2_ (1:4, v/v) as the mobile phase. Sixteen fractions were collected, analyzed by TLC, and regrouped into three pooled fractions (EAL-4.2.1 to EAL-4.2.3). Fraction EAL-4.2.2 (631 mg) was chromatographed again on Sephadex LH-20 with the same solvent system. The resulting subfraction (EAL-4.2.2.1, 523 mg) was purified by silica gel CC, eluted with a hexane- CH_2_Cl_2_-EtOAc mixture (1:1:1, v/v), affording compound **2** (360 mg).

### Separation of Epimers of **2** by Semipreparative HPLC and Analysis of Their Interconversion

The separation of epimers of **2** was performed by semipreparative HPLC-UV using an Ascentis C_18_ column (25 cm × 21.2 mm, 5 μm particle size). The mobile phase consisted of H_2_O (pump A) and MeCN-acetone (7:2 v/v, pump B), both containing 0.05% trifluoroacetic acid (TFA). Isocratic elution was performed with solvent ratios of 55% A and 45% B, resulting in a composition of H_2_O–MeCN-acetone (55:35:10, v/v/v). The flow rate was set at 10 mL min^–1^, and the injection volume was 500 μL (30 mg of sample). This procedure allowed the successful isolation of both diastereomers of **2** (**epimers 2a** and **2b**). The fractions containing compounds **2a** and **2b** were analyzed by analytical HPLC-UV using an Ascentiscolumn C_18_ (25 cm × 4.6 mm, 5 μm particle size) under the same mobile phase conditions employed in the semipreparative mode. The analytical method was performed with a flow rate of 1 mL min^–1^ and an injection volume of 20 μL (0.5 mg/mL). The analysis was carried out at 1 h after the semipreparative separation. The samples collected after separation by semipreparative HPLC were then extracted with CHCl_3_ and stored at 8 °C after solvent removal. ^1^H NMR and HPLC analyses (using the analytical method described above) of the samples were also performed after several days under storage at 8 °C.

### Chiral Phase HPLC Analyses

Both the plant-isolated and the synthetically obtained compounds were analyzed by HPLC-UV using a chiral Astec Cellulose DMP column (25 cm × 4.6 mm, 5 μm) and a mobile phase composed of hexane (pump A) and *i*-PrOH (pump B). Analyses were performed under an isocratic elution mode, with a ratio of 80% A and 20% B, at a flow rate of 0.8 mL/min. Detection was performed at 254 nm, and the injection volume was 10 μL (∼0.5 mg/mL).

### Computational Analysis

Molecular structures corresponding to the open and closed forms were built using Spartan’24 software (Wave function). For each form, a conformational search was performed using the Conformer Distribution module with the MMFF (Merck Molecular Force Field) method, allowing up to 10,000 automatically generated conformers. The lowest-energy conformer from each set was selected for further refinement. The geometries of the selected conformers were optimized using DFT with the B3LYP functional and the 6–31G­(d,p) basis set. Following optimization, vibrational frequency analyses were carried out at the same theoretical level to ensure that the structures correspond to true local minima (no imaginary frequencies) and to obtain the thermal, enthalpic, and entropic corrections required for the calculation of the Gibbs free energy (Δ*G*). The Δ*G* values were then used to compare the relative stability of the open and closed forms, as well as to calculate the equilibrium constant (*K*) using the thermodynamic relation Δ*G* = −*RT* ln *K*.^81^

#### Huajiaosimuline (**1**)

Brown oil; ^1^H NMR (600 MHz, CDCl_3_): δ 7.91 (dbr; *J* = 7.8 Hz; 1H; H10), 7.56 (tbr; *J* = 7.8 Hz, 1H, H8), 7.32 (dbr, *J* = 7.8 Hz, 1H, H7), 7.23 (t, *J* = 7.8 Hz, 1H, H9), 6.82 (d, *J* = 10.2 Hz, 1H, H4), 5.42 (d, *J* = 10.2 Hz, 1H, H3), 3.69 (s, 3H, H12), 2.65–2.60 (m, 2H, H2′), 2.55 (sept, *J* = 6.9 Hz, 1H, H4′), 2.18 (dt, *J* = 15.0, 9.0 Hz, 1H, Hb1′), 2.00 (dt, *J* = 15.0, 9.0 Hz, 1H, Ha1′), 1.48 (s, 3H, H11), 1.04 (d, *J* = 6.9 Hz, 3H, H6′), 1.01 (d, *J* = 6.9 Hz, 3H, H5′). ^13^C NMR (150 MHz, CDCl_3_): δ 214.1 (C3′), 161.1 (C5), 155.4 (C10b), 139.6 (C6a), 131.1 (C8), 124.7 (C4), 123.2 (C10), 122.0 (C9), 119.3 (C3), 115.9 (C4a), 114.3 (C7), 105.4 (C10a), 81.3 (C2), 41.2 (C4′), 35.4 (C1′), 35.3 (C2′), 29.5 (C12), 27.6 (C11), 18.5 (C6′), 18.3 (C5′); GC-EI-MS (70 eV): *m*/*z* 325 [M]^+^, 310, 227, 226 (100), 77, 43.

#### (−)-(3′*R*)-3′-Acetoxy-4′-hydroxyzanthosimuline (**2**)

Brown oil; [α]_D_^22^ = −6.6 (*c* 0.1, MeOH); UV (CH_2_Cl_2_) λ_max_ (log ε) 230 (4.3), 354 (3.8), 372 (3.7) nm; IR (ATR) ν_max_ 3422, 2974, 2936, 1732, 1648, 1609, 1324, 1244, 1020, 751 cm^–1^; ^1^H and ^13^C NMR data, see Table 1; HR-ESI-MS *m*/*z*: 408.1428 [M + H]^+^ (calcd for C_22_H_27_NO_5_Na, 408.1787).

#### (+)-(3′*R*)-Epoxyzanthosimuline (**3**)

Brown oil; [α]_D_^21^ = +3.3 (*c* 0.1; MeOH); UV (CH_2_Cl_2_) λ_max_ (log ε) 230 (4.4), 354 (3.8), 372 (3.7) nm; IR (ATR) ν_max_ 2976, 2926, 1644, 1630, 1586, 1324, 1121, 750 cm^–1^; ^1^H and ^13^C NMR data, see Table 2; HR-ESI-MS *m*/*z*: 326.1756 [M + H]^+^ (calcd C_20_H_24_NO_3_, 326.1751).

#### Mastigophorine (**4**)

Brown oil; UV (CH_2_Cl_2_) λ_max_ (log ε) 231 (4.5), 354 (3.9), 373 (3.8) nm; IR (ATR) ν_max_ 2969, 2929, 1636, 1615, 1579, 1162, 1092, 752 cm^–1^; ^1^H and ^13^C NMR data, see Table 3; HR-ESI-MS *m*/*z*: 348.1092 [M + Na]^+^ (calcd for C_20_H_23_NO_3_Na, 348.1576).

### Synthesis and Spectroscopic Data

#### Synthesis of Zanthosimuline (**6**)

A mixture of citral (geranial + neral) (0.96 g, 6.3 mmol, 1.1 equiv) and anhydrous MgSO_4_ (2.0 g) in pyridine (8.0 mL) was refluxed for 10 min. Then, 4-hydroxy-6-methylquinolin-2­(1*H*)-one (1.0 g, 5.7 mmol, 1.0 equiv) was slowly added. The reaction mixture was refluxed overnight. After cooling at room temperature, pyridine was removed under reduced pressure using a rotary evaporator. The resulting residue was resuspended in H_2_O and extracted with CH_2_Cl_2_. The organic layer was washed with 1 M HCl, aqueous NaHCO_3_, and brine, dried over anhydrous Na_2_SO_4_ and concentrated under reduced pressure using a rotary evaporator. The crude product was purified by CC on silica gel using hexane/EtOAc (3:1 v/v) as the eluent.

#### Zanthosimuline (**6**)

Brown oil (1.5 g, 3.87 mmol, 85% yield); IR (ATR) ν_max_ 1650, 1617, 1590, 1570, 1505 cm^–1^; ^1^H NMR (400 MHz, CDCl_3_): δ 7.95 (dd, *J* = 8.0, 1.6 Hz, 1H, H10), 7.52 (ddd, *J* = 8.6, 7.2, 1.6 Hz, 1H, H8), 7.29 (d, *J* = 8.6 Hz, 1H, H7), 7.21 (t, *J* = 8.0 Hz, 1H, H9), 6.80 (d, *J* = 10.0 Hz, 1H, H4), 5.48 (d, *J* = 10.0 Hz, 1H, H3), 5.09 (tt, *J* = 7.9, 1.4 Hz, 1H, H3′), 3.68 (s, 3H, H12), 2.14 (q, *J* = 7.9 Hz, 2H, H2′), 1.90–1.80 (m, 1H, Ha1′), 1.79–1.68 (m, 1H, Hb1′), 1.62 (s, 3H), 1.55 (s, 3H), 1.48 (s, 3H, H11); ^13^C NMR (100 MHz, CDCl_3_): δ 161.0 (C5), 155.4 (C10b), 139.4 (C6a), 131.9 (C4′), 130.9 (C8), 125.3 (C3), 123.8 (C3′), 123.1 (C9), 121.7 (C10), 118.4 (C7), 116.0 (C4a), 114.0 (C4), 105.5 (C10a), 81.3 (C2), 41.6 (C1′), 29.3 (C12), 27.1 (C11), 25.7 (C5′), 22.6 (C2′), 17.6 (C6′).

### Synthesis of Diols **5α** e **5β**

AD-mix α (0.91 g, 1.4 g/mmol of diol) was added stepwise to a cooled and stirred solution of **6** (200 mg, 0.65 mmol) and methanesulfonamide (61.8 mg, 0.65 mmol) in BuOH-H_2_O (1:1 v/v) (10.0 mL). The reaction mixture was stirred at 0 °C for 48 h. After this period, the reaction was quenched by the addition of a saturated aqueous Na_2_S_2_O_3_ (5.0 mL), and the mixture was extracted with EtOAc (3 × 10.0 mL). The combined organic layers were washed with brine (10 mL), dried over anhydrous Na_2_SO_4_, and concentrated under reduced pressure using a rotary evaporator. The crude product was purified by CC on silica gel using hexane-EtOAc (1:4, v/v) as an eluent, affording diol **5α**. The same procedure was repeated using AD-mix β, yielding diol **5β**.

### (−)-(2*R*, 3′*S*)-(3′,4′)-Dihydroxyzanthosimuline + (2*S*, 3′*S*)-(3′,4′)-Dihydroxyzanthosimuline (**5α**)

Brown oil (152.0 mg, 0.44 mmol, 68% yield, 95% *ee*); [α]_D_^22^ = −21.7 (_C_ 0.1, MeOH); UV (CH_2_Cl_2_) λ_max_ (log ε) 232 (4.6), 335 (3.8), 354 (3.8), 372 (3.6) nm; IR (ATR) ν_max_ 3389, 2970, 2931, 1643, 1610, 1579, 1363, 1160, 750, 727 cm^–1^; ^1^H NMR (400 MHz, CDCl_3_) δ: 7.92/7.91 (dd, *J* = 8.0, 1.6 Hz, 2H, H10), 7.51 (m, 2H, H8), 7.28 (dbr, *J* = 8.5 Hz, 2H, H7), 7.23–7.17 (m, 2H, H9), 6.75 (d, *J* = 10.1 Hz, 2H, H4), 5.47 (d, *J* = 10.1 Hz, 2H, H3), 3.64 (s, 6H, H12), 3.34/3.33 (dd, *J* = 10.5, 2.1 Hz, 2H, H3′), 2.23–2.00 (m, 2H, Ha1′), 1.93–1.69 (m, 2H, Hb1′), 1.74–1.60 (m, 2H, Ha2′), 1.56–1.36 (m, 2H, Hb2′), 1.46/1.45 (s, 6H, H11), 1.16/1.14 (s, 6H, H5′), 1.14/1.10 (s, 6H, H6′); ^13^C NMR (100 MHz, CDCl_3_) δ: 161.1 (C5), 155.5 (C10b), 139.3 (C6a), 131.0 (C9), 125.7/125.4 (C3), 123.1 (C10), 122.0 (C7), 118.7/118.5 (C4), 116.0 (C10a), 114.2 (C8), 105.6 (C4a), 81.7/81.4 (C2), 78.7/78.6 (C3′), 73.2 (C4′), 38.9/38.7 (C1′), 29.4 (C12), 27.3/26.8 (C11), 26.7/26.6 (C5′), 26.3/25.8 (C2′), 23.4 (C6′); HR-ESI-MS *m*/*z*: 366.1735 [M + Na]^+^ (calcd for C_20_H_25_NO_4_Na, 366.1681).

### (+)-(2*R*, 3′*R*)-(3′,4′)-Dihydroxyzanthosimuline + (2*S*, 3′*R*)-(3′,4′)-Dihydroxyzanthosimuline (**5β**)

Brown oil (135.0 mg, 0.39 mmol, 60% yield, 96% *ee*); [α]_D_^22^ = +24.2 (_C_ 0.1, MeOH); UV (CH_2_Cl_2_) λ_max_ (log ε) 232 (4.6), 335 (3.8), 354 (3.8), 372 (3.6) nm; IR (ATR) ν_max_ 3400, 2968, 2931, 1643, 1606, 1580, 1363, 1160, 750, 727 cm^–1^; ^1^H NMR (400 MHz, CDCl_3_) δ: 7.93/7.92 (dd, *J* = 8.0, 1.6 Hz, 2H, H10), 7.52 (m, 2H, H8), 7.28 (dbr, *J* = 8.5 Hz, 2H, H7), 7.23–7.17 (m, 2H, H9), 6.76 (d, *J* = 10.1 Hz, 2H, H4), 5.48 (d, *J* = 10.1 Hz, 2H, H3), 3.65 (s, 6H, H12), 3.35/3.34 (dd, *J* = 10.5, 2.1, 2H, H3′), 2.23–2.00 (m, 2H, Ha1′), 1.93–1.69 (m, 2H, Hb1′), 1.74–1.60 (m, 2H, Ha2′), 1.56–1.36 (m, 2H, Hb2′), 1.46/1.45 (s, 6H, H11), 1.16/1.14 (s, 6H, H5′), 1.14/1.10 (s, 6H, H6′); ^13^C NMR (100 MHz, CDCl_3_) δ: 161.1 (C5), 155.5 (C10b), 139.3 (C6a), 131.0 (C9), 125.7/125.4 (C3), 123.1 (C10), 122.0 (C7), 118.7/118.5 (C4), 116.0 (C10a), 114.2 (C8), 105.6 (C4a), 81.7/81.4 (C2), 78.7/78.6 (C3′), 73.2 (C4′), 38.9/38.7 (C1′), 29.4 (C12), 27.3/26.8 (C11), 26.7/26.6 (C5′), 26.3/25.8 (C2′), 23.4 (C6′); HR-ESI-MS *m*/*z*: 366.1697 [M + Na]^+^ (calcd for C_20_H_25_NO_4_Na, 366.1776).

### Synthesis of Acetoxyzanthosimuline (**2α** and **2β**)

Acetic anhydride (3.45 mmol) was added, dropwise, to a solution of respective diol (**5α** and **5β**, 80 mg, 0.23 mmol) and DMAP (5.0 mg, 0.046 mmol) in pyridine (3 mL). The reaction mixture was stirred at room temperature for 30 min and then quenched by the addition of 1 M aqueous HCl (5 mL). The product was extracted with EtOAc (3 × 10 mL), and the combined organic layers were washed with brine, dried over anhydrous Na_2_SO_4_, and concentrated under reduced pressure by using a rotary evaporator. The crude product was purified by CC on silica gel, using hexane-EtOAc (1:3, v/v) as the eluent to afford **2α** and **2β**.

### (+)-(2′*R*, 3′*S*)-3′-Acetoxy-4′-hydroyzanthosimuline + (2′*S*, 3′*S*)-3′-Acetyl-4′-hydroxyzanthosimuline (**2α**)

Brown oil (71 mg, 0.18 mmol, 80% yield); [α]_D_^22^ = +7.1 (_C_ 0.1, MeOH); UV (CH_2_Cl_2_) λ_max_ (log ε) 230 (4.4), 354 (3.8), 372 (3.7) nm; IR (ATR) ν_max_ 3403, 2974, 2936, 1728, 1643, 1612, 1582, 1364, 1238, 1026, 750 cm^–1^. ^1^H NMR (400 MHz, CDCl_3_) δ: 7.93/7.91 (dd, *J* = 8.2, 1.6 Hz, 2H, H10), 7.56–7.49 (m, 2H, H8), 7.32/7.27 (dbr, 2H, H7), 7.25/7.18 (m, 2H, H9), 6.79/6.78 (d, *J* = 10.0 Hz, 2H, H4), 5.46/5.43 (d, *J* = 10.0 Hz, 2H, H3), 4.85–4.76 (m, 2H, H3′), 3.66 (s, 6H, H12), 2.10/2.07 (s, 6H, H8′), 1.92–1.79 (m, 4H, Ha1’/Hb2′), 1.77–1.65 (m, 4H, Hb1’/Ha2′), 1.44 (s, 6H, H11), 1.15 (s, 6H, H5′), 1.14 (s, 6H, H6′) ^13^C NMR (100 MHz, CDCl_3_) δ: 171.3 (C7′), 161.0 (C5), 155.3 (C10b), 139.5 (C6a), 131.1 (C8), 125.1/124.9 (C3), 123.2/123.1 (C10), 121.9 (C9), 119.1/118.9 (C4), 116.0 (C10a), 114.2 (C7), 105.6 (C4a), 81.3/81.0 (C2), 79.9 (C3′), 72.4 (C4′), 38.1/38.0 (C1′), 29.4 (C12), 27.2/27.0 (C11), 26.7/26.7 (C5′), 25.3/25.2 (C6′), 24.3/23.9 (C2′), 21.2/21.1 (C8′). HR-ESI-MS *m*/*z* 386.1972 [M + H]^+^ (calcd for C_22_H_28_NO_5,_ 386.1962).

### (−)-(2′*R*, 3′*R*)-3′-Acetoxy-4′-hydroxyzanthosimuline + (2′*S*,3′*R*)-3′-Acetoxy-4′-hydroxyzanthosimuline (**2β**)

Brown oil (71 mg, 0.18 mmol, 80% yield); [α]_D_^22^ = −11.8 (_C_ 0.1, MeOH); UV (CH_2_Cl_2_) λ_max_ (log ε) 230 (4.4), 354 (3.8), 372 (3.7) nm; IR (ATR) ν_max_ 3411, 2979, 2928, 2363, 1730, 1644, 1606, 1241, 724 cm^–1^; ^1^H NMR (400 MHz, CDCl_3_) δ: 7.94/7.92 (dd, *J* = 8.2, 1.6 Hz, 2H, H10), 7.56–7.49 (m, 2H, H8), 7.30 (dbr, *J* = 8.7 Hz, 2H, H7), 7.25/7.18 (m, 2H, H9), 6.79/6.78 (d, *J* = 10.0 Hz, 2H, H4), 5.46/5.43 (d, *J* = 10.0 Hz, 2H, H3), 4.85–4.76 (m, 2H, H3′), 3.67 (s, 6H, H12), 2.10/2.07 (s, 6H, H8′), 1.92–1.79 (m, 4H, Ha1′/Hb2′), 1.77–1.65 (m, 4H, Hb1′/Ha2′), 1.45/1.44 (s, 6H, H11), 1.16 (s, 6H, H5′), 1.15/1.14 (s, 6H, H6′); ^13^C NMR (100 MHz, CDCl_3_) δ: 171.3 (C7′), 161.0 (C5), 155.3 (C10b), 139.5 (C6a), 131.1 (C8), 125.1/124.9 (C3), 123.2/123.1 (C10), 121.9 (C9), 119.1/119.0 (C4), 116.0 (C10a), 114.2 (C7), 105.6 (C4a), 81.3/81.0 (C2), 79.9 (C3′), 72.5 (C4′), 38.1/38.0 (C1′), 29.4 (C12), 27.2/27.0 (C11), 26.7/26.6 (C5′), 25.3/25.2 (C6′), 24.3/23.9 (C2′), 21.2/21.1 (C8′); HR-ESI-MS *m*/*z*: 408.1810 [M + Na]^+^ (calcd for C_22_H_27_NO_5_Na, 408.1781).

### Epoxidation of Zanthosimuline

Enantioselective epoxidation: zanthosimuline (200 mg, 0.65 mmol, 1.0 equiv) was dissolved in MeCN-dioxane (10 mL, 1:2, v/v). A buffer solution (8 mL) composed of 0.05 M of sodium tetraborate decahydrate (Na_2_B_4_O_7_·10H_2_O) in 4 × 10^–4^ M aqueous disodium EDTA was then added, followed by tetrabutylammonium hydrogen sulfate (0.05 equiv) and Shi’s ketone (0.3 equiv). The reaction mixture was cooled to 0 °C. A solution of Oxone (318 mg, 1.6 equiv) in aqueous disodium EDTA (4 × 10^–4^ M, 6 mL) and a solution of K_2_CO_3_ (518 mg, 5.8 equiv) in H_2_O (6 mL) were added dropwise, separately, and simultaneously, over 1.5 h, via syringe pumps. After this period, the reaction mixture was diluted with H_2_O and extracted with EtOAc (3 × 15 mL). The combined organic layers were washed with brine and dried over anhydrous Na_2_SO_4_, and the solvent was removed under reduced pressure using a rotary evaporator. The crude product was purified by CC on silica gel using hexane-EtOAc (3:2, v/v) as the eluent, affording **3β**.

Nonselective epoxidation: zanthosimuline (100 mg, 0.32 mmol) was dissolved in CH_2_Cl_2_ (3.0 mL) in a 10 mL round-bottom flask. The solution was cooled to 0 °C, and *m*-CPBA (77%, 116.2 mg, 0.39 mmol, 1.2 equiv) was added slowly. The reaction mixture was stirred at 0 °C for 4 h. The mixture was then washed with aqueous K_2_CO_3_ (5 mL), dried over anhydrous Na_2_SO_4_, and concentrated under reduced pressure. The crude product was purified according to a previously described procedure to afford compound **3β**.

### (+)-Epoxizanthosimuline (**3β**)

Brown oil (160 mg, 0.49 mmol, 76% yield, 92% *ee*); UV (CH_2_Cl_2_) λ_max_ (log ε) 230 (4.4), 354 (3.9), 372 (3.7) nm; IR (ATR) ν_max_ 2967, 2926, 1643, 1626, 1586, 1324, 1121, 750 cm^–1^; [α]_D_^22^ = +4.4 (_C_ 0.1, MeOH); ^1^H NMR (400 MHz, CDCl_3_) δ: 7.92 (dd, *J* = 8.0, 1.6 Hz, 2H, H10), 7.55–7.49 (m, 2H, H8), 7.29 (dbr, *J* = 8.5 Hz, 2H, H7), 7.22–7.17 (m, 2H, H9), 6.79 (d, *J* = 10.0 Hz, 2H, H4), 5.47/5.45 (d, *J* = 10.0 Hz, 2H, H3), 3.67 (s, 6H, H12), 2.75–2.67 (m, 2H, H3′), 2.05–1.89 (m, 2H, Ha1′), 1.87–1.77 (m, 2H, Hb1′), 1.74–1.62 (m, 4H, H2′), 1.48 (s, 6H, H11), 1.26/1.22 (s, 6H, H5′), 1.20/1.17 (s, 6H, H6′); ^13^C NMR (100 MHz, CDCl_3_) δ: 161.0 (C5), 155.3 (C10b), 139.5 (C6a), 131.1 (C8), 125.1/124.8 (C3), 123.1/123.0 (C10), 121.9 (C9), 119.0/118.9 (C4), 115.9 (C10a), 114.2 (C7), 105.6/105.5 (C4a), 81.2/80.9 (C2), 64.2/64.0 (C3′), 58.7/58.6 (C4′), 38.3/38.2 (C1′), 29.4 (C12), 27.3/27.0 (C11), 24.9 (C5′), 23.8/23.6 (C2′), 18.7 (C6′); HR-ESI-MS *m*/*z*: 326.1755 [M + H]^+^ (calcd for C_20_H_24_NO_3_, 326.1784).

### Epoxizanthosimuline Mixture (Nonselective Product—**3a–d**)

Brown oil (83.0 mg, 0.25 mol, 80% yield); IR (ATR, cm^–1^); [α]_D_^21^ = 0 (_C_ 0.1, MeOH); ν_max_ 2959, 2936, 1640, 1633, 1598, 1321, 1018, 752 cm^–1^; ^1^H NMR (400 MHz, CDCl_3_) δ: 7.93 (dd, *J* = 8.0, 1.6 Hz, 2H, H10), 7.55–7.49 (m, 2H, H8), 7.30 (dbr, *J* = 8.5 Hz, 2H, H7), 7.22 (t, *J* = 7.6 Hz, 2H, H9), 6.80 (d, *J* = 10.0 Hz, 2H, H4), 5.48/5.47 (d, *J* = 10.0 Hz, 2H, H3), 3.69 (s, 6H, H12), 2.77–2.69 (m, 2H, H3′), 2.07–1.91 (m, 2H, Ha1′), 1.89–1.79 (m, 2H, Hb1′), 1.76–1.64 (m, 4H, H2′), 1.49 (s, 6H, H11), 1.27/1.23 (s, 6H, H5′), 1.21/1.18 (s, 6H, H6′); ^13^C NMR (100 MHz, CDCl_3_) δ: 161.1 (C5), 155.4 (C10b), 139.5 (C6a), 131.1 (C8), 125.1/124.9 (C3), 123.1 (C10), 122.0 (C9), 119.1/119.0 (C4), 115.9 (C10a), 114.2 (C7), 105.6/105.5 (C4a), 81.2/80.9 (C2), 64.2/64.1 (C3′), 58.7/58.6 (C4′), 38.3/38.2 (C1′), 29.4 (C12), 27.3/27.0 (C11), 24.9 (C5′), 23.8/23.6 (C2′), 18.7 (C6′).

## Supplementary Material



## Data Availability

The original FIDs of the ^1^H, ^13^C NMR and 2D NMR data for the new isolated and synthetic compounds have been deposited in the nmrXvi database and are publicy available (DOI: 10.57992/nmrxiv.p145; https://nmrxiv.org/project/KIzR4CDtBxSl5TfoFobcQkrbzOozp3Mf68E0l10Y).

## Notes

Spectroscopic and spectrometric data of naturals compounds **1**, **2**, **3**, and **4**, and synthetic compounds **2α**, **2β**, **3β**, **3a**–d, **5α**, and **5β**; including the analytical (chiral and reversed-phase C_18_) and semipreparative HPLC chromatograms of the natural and synthetic compounds (PDF)
