# Supplementary material for: Isolation, Identification, and Total Synthesis of Pyranoquinolinone Alkaloids from Conchocarpus mastigophorus Kallunki (Rutaceae)
Source: J Nat Prod. 2026 Jan 15;89(2):492–505. doi: 10.1021/acs.jnatprod.5c01326 (PMC12954846; doi:10.1021/acs.jnatprod.5c01326)
Supplement: Supplementary file 1 [file np5c01326_si_001.pdf]

Isolation, identification and total synthesis of pyranoquinolinone alkaloids from *Conchocarpus mastigophorus* Kallunki (Rutaceae)

Anderson R. Santos[‡], Vanderlúcia F. de Paula[§], Amanda S. de Miranda[‡], Júnio G. Silva[‡], Luiz C. A. Barbosa[‡]*

[‡]Department of Chemistry, Universidade Federal de Minas Gerais, Av. Pres. Antonio Carlos, 6627, Campus Pampulha, CEP 31270-901, Belo Horizonte, MG, Brazil. **E-mail*: lcab@ufmg.br

[§]Department of Science and Technology, Universidade Estadual do Sudoeste da Bahia, Av. José Moreira Sobrinho, s/n, CEP 45208-091, Jequié, Bahia, Brazil.

Table of contents

Figure S 1. ^{1}H NMR spectrum of compound **1** (600 MHz, $CDCl_3$) 7

Figure S 2. Expansion 1 of the ^{1}H NMR spectrum of compound **1** (600 MHz, $CDCl_3$) 8

Figure S 3. Expansion 2 of the ^{1}H NMR spectrum of compound **1** (600 MHz, $CDCl_3$) 9

Figure S 4. ^{13}C NMR spectrum of compound **1** (150 MHz, $CDCl_3$) 10

Figure S 5. ^{1}H NMR spectrum of compound **2** (400 MHz, $CDCl_3$) 11

Figure S 6. Expansion 1 of the ^{1}H NMR spectrum of compound **2** (400 MHz, $CDCl_3$) 12

Figure S 7. Expansion 2 of the ^{1}H NMR spectrum of compound **2** (400 MHz, $CDCl_3$) 13

Figure S 8. Expansion 3 of the ^{1}H NMR spectrum of compound **2** (400 MHz, $CDCl_3$) 14

Figure S 9. ^{13}C NMR spectrum of compound **2** (150 MHz, $CDCl_3$) 15

Figure S 10. Expansion of the ^{13}C NMR spectrum of compound **2** (150 MHz, $CDCl_3$) 16

Figure S 11. HSQC spectrum of compound **2** (^{1}H: 600 MHz, ^{13}C: 150 MHz, $CDCl_3$) 17

Figure S 12. HMBC spectrum of compound **2** (^{1}H: 600 MHz, ^{13}C: 150 MHz, $CDCl_3$) 18

Figure S 13. (+)-HRESIMS spectrum of compound **2** 18

Figure S 14 - IR spectrum of compound **2** 19

Figure S 15. UV Spectrum (16 µg/mL, CH_2Cl_2) of compound **2** 19

Figure S 16. ^{1}H NMR spectrum of compound **3**(400 MHz, $CDCl_3$) 20

Figure S 17. Expansion 1 of the ^{1}H NMR spectrum of compound **3** (400 MHz, $CDCl_3$) 21

Figure S 18. Expansion 2 of ^{1}H NMR spectrum of compound **3** (400 MHz, $CDCl_3$) 22

Figure S 19. Expansion 3 of ^{1}H NMR spectrum of compound **3** (400 MHz, $CDCl_3$) 23

Figure S 20. ^{13}C NMR spectrum of compound **3** (100 MHz, $CDCl_3$) 24

Figure S 21. HSQC spectrum of compound **3** (^{1}H: 400 MHz, ^{13}C: 100 MHz, $CDCl_3$) 25

Figure S 22. HMBC spectrum of compound **3** (^{1}H: 400 MHz, ^{13}C: 100 MHz, $CDCl_3$) 26

Figure S 23. COSY spectrum of compound **3** (400 MHz, $CDCl_3$)....27

Figure S 24. (+)-HRESIMS spectrum of compound **3** 28

Figure S 25. IR spectrum of compound 3 28

Figure S 26. UV spectrum (15 μg/mL, CH_2Cl_2) of compound 3 29

Figure S 27. ^{1}H NMR spectrum of compound **4** (600 MHz, $CDCl_3$) 30

Figure S 28. Expansion 1 of the ^{1}H NMR spectrum of compound **4** (600 MHz, $CDCl_3$) 31

Figure S 29. Expansion 2 of the ^{1}H NMR spectrum of compound **4** (600 MHz, $CDCl_3$) 32

Figure S 30. Expansion 3 of the ^{1}H NMR spectrum of compound **4** (600 MHz, $CDCl_3$) 33

Figure S 31. ^{13}C NMR spectrum of compound **4** (150 MHz, $CDCl_3$) 34

Figure S 32. HSQC spectrum of compound **4** (^{1}H: 600 MHz, ^{13}C: 150 MHz, $CDCl_3$) 35

Figure S 33. HMBC spectrum of compound **4** (^{1}H: 600 MHz, ^{13}C: 150 MHz, $CDCl_3$) 36

Figure S 34. COSY spectrum of compound **4** (600 MHz, $CDCl_3$) 37

Figure S 35. (+)-HRESIMS spectrum of compound **4** 38

Figure S 36. IR spectrum of compound **4** 38

Figure S 37. UV spectrum (15 μg/mL, CH_2Cl_2) of compound **4** 39

Figure S 38. ^{1}H NMR spectrum of compound **6** (400 MHz, $CDCl_3$) 39

Figure S 39. Expansion 1 of the ^{1}H NMR spectrum of compound **6** (400 MHz, $CDCl_3$) 40

Figure S 40. Expansion 2 of the ^{1}H NMR spectrum of compound **6** (400 MHz, $CDCl_3$) 41

Figure S 41. Expansion 3 of the ^{1}H NMR spectrum of compound **6** (400 MHz, $CDCl_3$) 42

Figure S 42. ^{13}C NMR spectrum of compound **6** (100 MHz, $CDCl_3$) 43

Figure S 43. ^{1}H NMR spectrum of compound **5*α*** (400 MHz, $CDCl_3$) 44

Figure S 44. Expansion 1 of ^{1}H NMR spectrum of compound **5*α*** (400 MHz, $CDCl_3$) 45

Figure S 45. Expansion 2 of ^{1}H NMR spectrum of compound **5*α*** (400 MHz, $CDCl_3$) 46

Figure S 46. Expansion 3 of ^{1}H NMR spectrum of compound **5*α*** (400 MHz, $CDCl_3$) 47

Figure S 47. ^{13}C NMR spectrum of compound **5*α*** (100 MHz, $CDCl_3$) 48

Figure S 48. Expansion of the ^{13}C NMR spectrum of compound **5*α*** (100 MHz, $CDCl_3$) 49

Figure S 49. HSQC spectrum of compound **5α** (^{1}H: 400 MHz, ^{13}C: 100 MHz, $CDCl_3$) 50

Figure S 50. HMBC spectrum of compound **5α** (^{1}H: 400 MHz, ^{13}C: 100 MHz, $CDCl_3$) 51

Figure S 51. (+)-HRESIMS spectrum of compound **5α** 52

Figure S 52. IR spectrum of compound **5α** 52

Figure S 53. UV spectrum (17 μg/mL, CH_2Cl_2) of compound **5α** 53

Figure S 54. ^{1}H NMR spectrum of compound **5β** (400 MHz, $CDCl_3$) 53

Figure S 55. ^{13}C NMR spectrum of compound **5β** (100 MHz, $CDCl_3$) 54

Figure S 56. (+)-HRESIMS spectrum of compound **5β** 55

Figure S 57. IR spectrum of compound **5β** 55

Figure S 58. UV spectrum (15 μg/mL, CH_2Cl_2) of compound **5β** 56

Figure S 59. ^{1}H NMR spectrum of compound **2α** (400 MHz, $CDCl_3$) 57

Figure S 60. Expansion 1 of the ^{1}H NMR spectrum of compound **2α** (400 MHz, $CDCl_3$) 57

Figure S 61. Expansion 2 of the ^{1}H NMR spectrum of compound **2α** (400 MHz, $CDCl_3$) 58

Figure S 62. Expansion 3 of the ^{1}H NMR spectrum of compound **2α** (400 MHz, $CDCl_3$) 59

Figure S 63. ^{13}C NMR spectrum of compound **2α** (100 MHz, $CDCl_3$) 60

Figure S 64. Expansion of the ^{13}C NMR spectrum of compound **2α** (100 MHz, $CDCl_3$) 61

Figure S 65. HSQC spectrum of compound **2α** (1H: 400 MHz, ^{13}C: 100 MHz, $CDCl_3$) 62

Figure S 66. HMBC spectrum of compound **2α** (^{1}H: 400 MHz, ^{13}C: 100 MHz, $CDCl_3$) 63

Figure S 67. COSY spectrum of compound **2α** (400 MHz, $CDCl_3$) 64

Figure S 68. (+)-HRESIMS spectrum of compound **2α** 65

Figure S 69. IR spectrum of compound **2α** 66

Figure S 70. UV spectrum (15 µg/mL, CH_2Cl_2) of compound **2α**...66

Figure S 71. ^{1}H NMR spectrum of compound **2β** (400 MHz, $CDCl_3$)..67

Figure S 72. Expansion 1 of the ^{1}H NMR spectrum of compound **2β** (400 MHz, $CDCl_3$)..........................68

Figure S 73. Expansion 2 of the ^{1}H NMR spectrum of compound **2β** (400 MHz, $CDCl_3$)..........................69

Figure S 74. Expansion 3 of the ^{1}H NMR spectrum of compound **2β** (400 MHz, $CDCl_3$)..........................70

Figure S 75. ^{13}C NMR spectrum of compound **2β** (100 MHz, $CDCl_3$)..71

Figure S 76. Expansion of the ^{13}C NMR spectrum of compound **2β** (100 MHz, $CDCl_3$)............................72

Figure S 77. HSQC spectrum of compound **2β** (^{1}H: 400 MHz, ^{13}C: 100 MHz, $CDCl_3$)..............................73

Figure S 78. COSY spectrum of compound **2β** (400 MHz, $CDCl_3$) 74

Figure S 79. (+)-HRESIMS spectrum of compound **2β**...75

Figure S 80. IR spectrum of compound **2β**..75

Figure S 81. UV spectrum (17 µg/mL, CH_2Cl_2) of compound **2β**...76

Figure S 82. ^{1}H NMR spectrum of compound **3β** (400 MHz, $CDCl_3$)..77

Figure S 83. Expansion 1 of the ^{1}H NMR spectrum of compound **3β** (400 MHz, $CDCl_3$)..........................77

Figure S 84. Expansion 2 of the ^{1}H NMR spectrum of compound **3β** (400 MHz, $CDCl_3$)..........................78

Figure S 85. Expansion 3 of the ^{1}H NMR spectrum of compound **3β** (400 MHz, $CDCl_3$)..........................79

Figure S 86. ^{13}C NMR spectrum of compound **3β** (100 MHz, $CDCl_3$)..80

Figure S 87. HSQC spectrum of compound **3β** (^{1}H: 400 MHz, ^{13}C: 100 MHz, $CDCl_3$)..............................81

Figure S 88. HMBC spectrum of compound **3β** (^{1}H: 400 MHz, ^{13}C: 100 MHz, $CDCl_3$)..............................82

Figure S 89. COSY spectrum of compound **3β** (400 MHz, $CDCl_3$) 83

Figure S 90. (+)-HRESIMS spectrum of compound **3β**...84

Figure S 91. IR spectrum of compound **3β** 85

Figure S 92. UV spectrum (15 μg/mL, CH_2Cl_2) of compound **3β** 85

Figure S 93. Chiral phase HPLC Chromatogram of the synthetic compounds **5α** and **5β** 86

Figure S 94. Chiral phase HPLC chromatogram of natural product **2**, synthetic compound **2α**, and their mixture (**2** + **2α**) 87

Figure S 95. Chiral phase HPLC chromatogram of synthetic compound **3β** and racemic synthetic compound **3a-d** 88

Figure S 96. Semipreparative HPLC chromatogram of natural product **2** 89

Figure S 97. Analytical HPLC chromatogram obtained from samples of epimer obtained from separation through semipreparative HPLC. 90

Figure S 98. Overlap of HPLC chromatograms of compound **4**. I. Semipreparative column analysis. II. Analytical column analysis. III. and IV. Re-injection of the isolated diastereoisomers into the analytical column immediately after separation. 91

Figure S 99. Pictures of *Conchocarpus mastigophorus* Kallunki (Rutaceae) 89

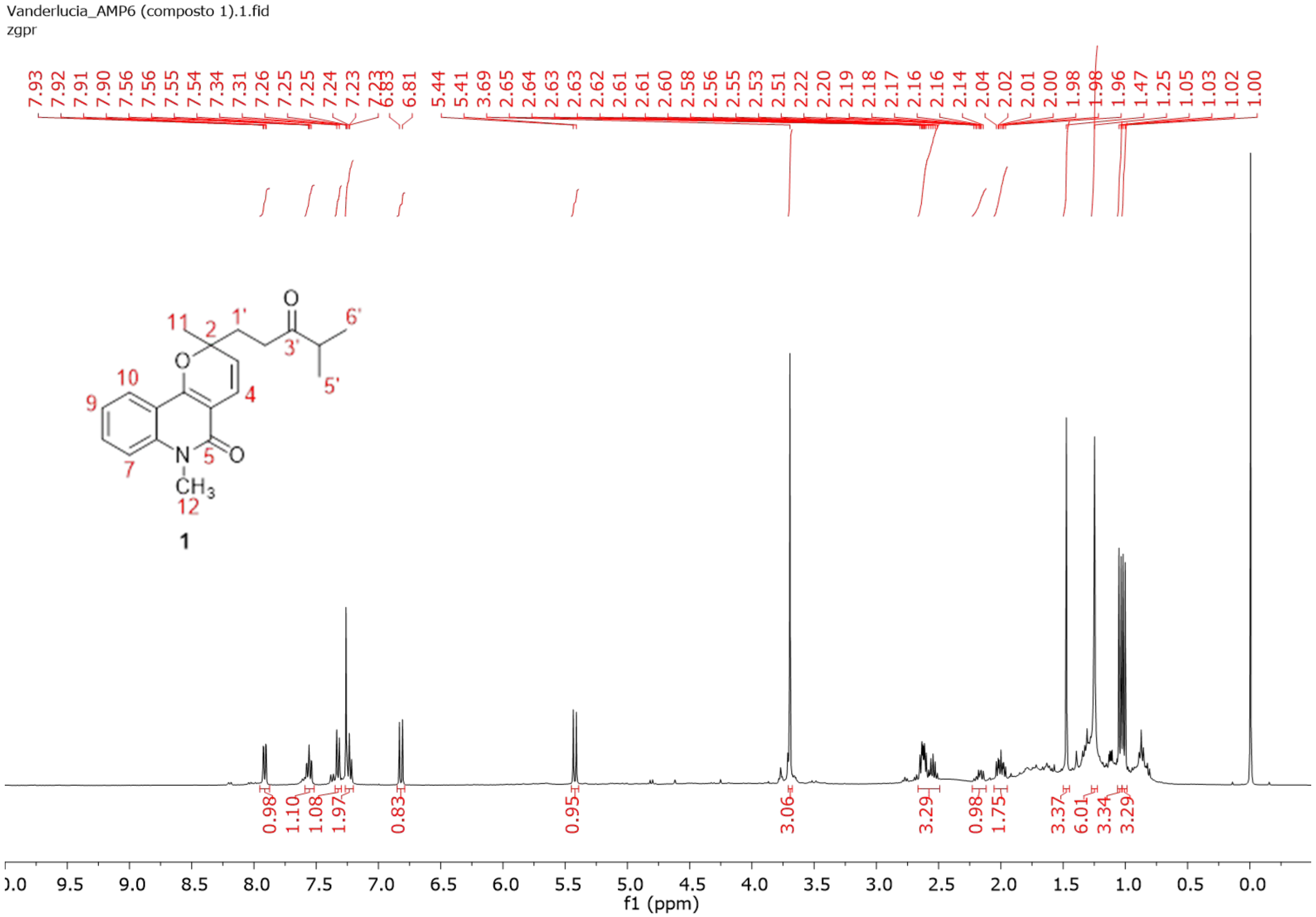

Figure S 1. ^{1}H NMR spectrum of compound **1** (600 MHz, $CDCl_3$)

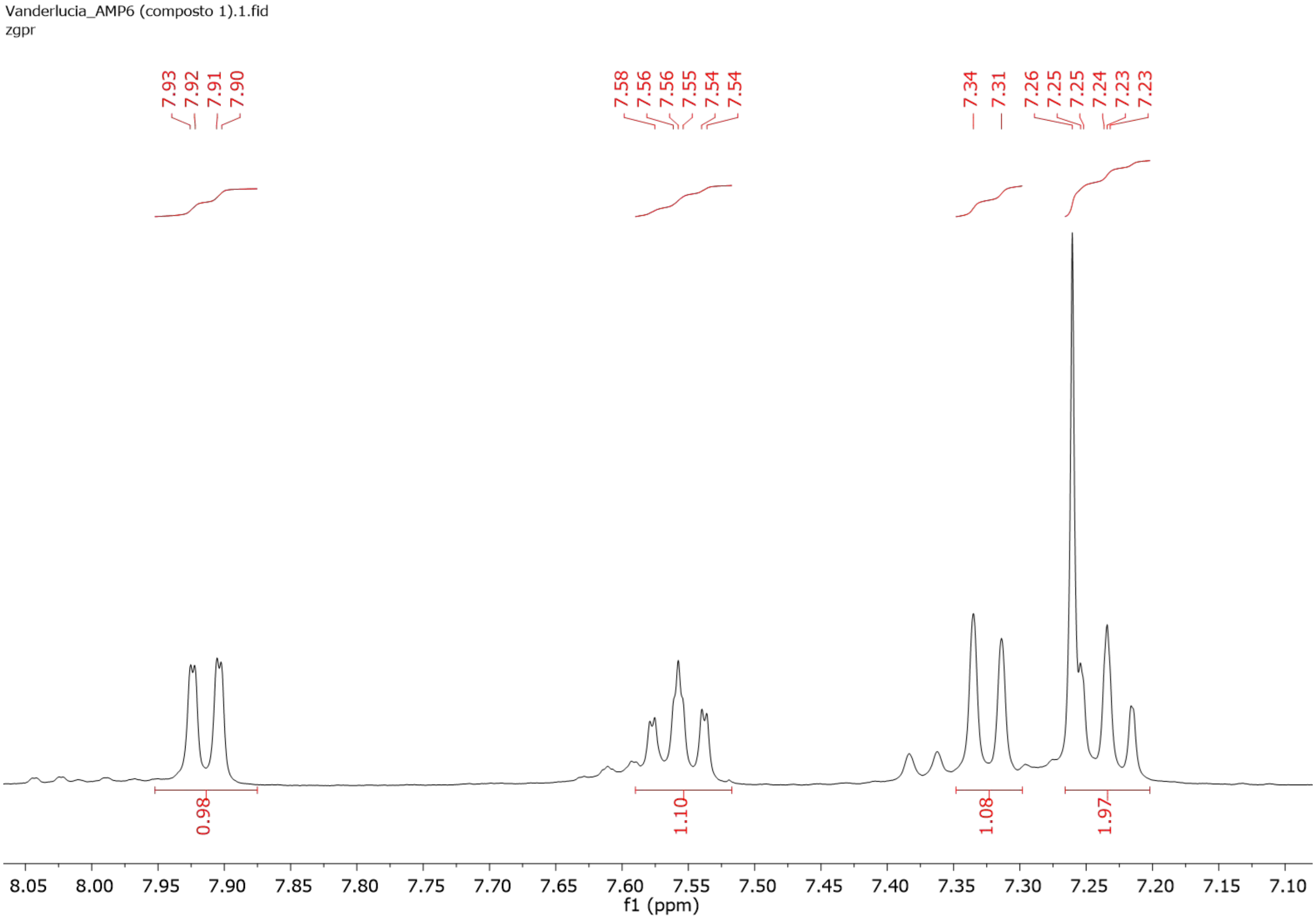

Figure S 2. Expansion 1 of the ^{1}H NMR spectrum of compound **1** (600 MHz, $CDCl_3$)

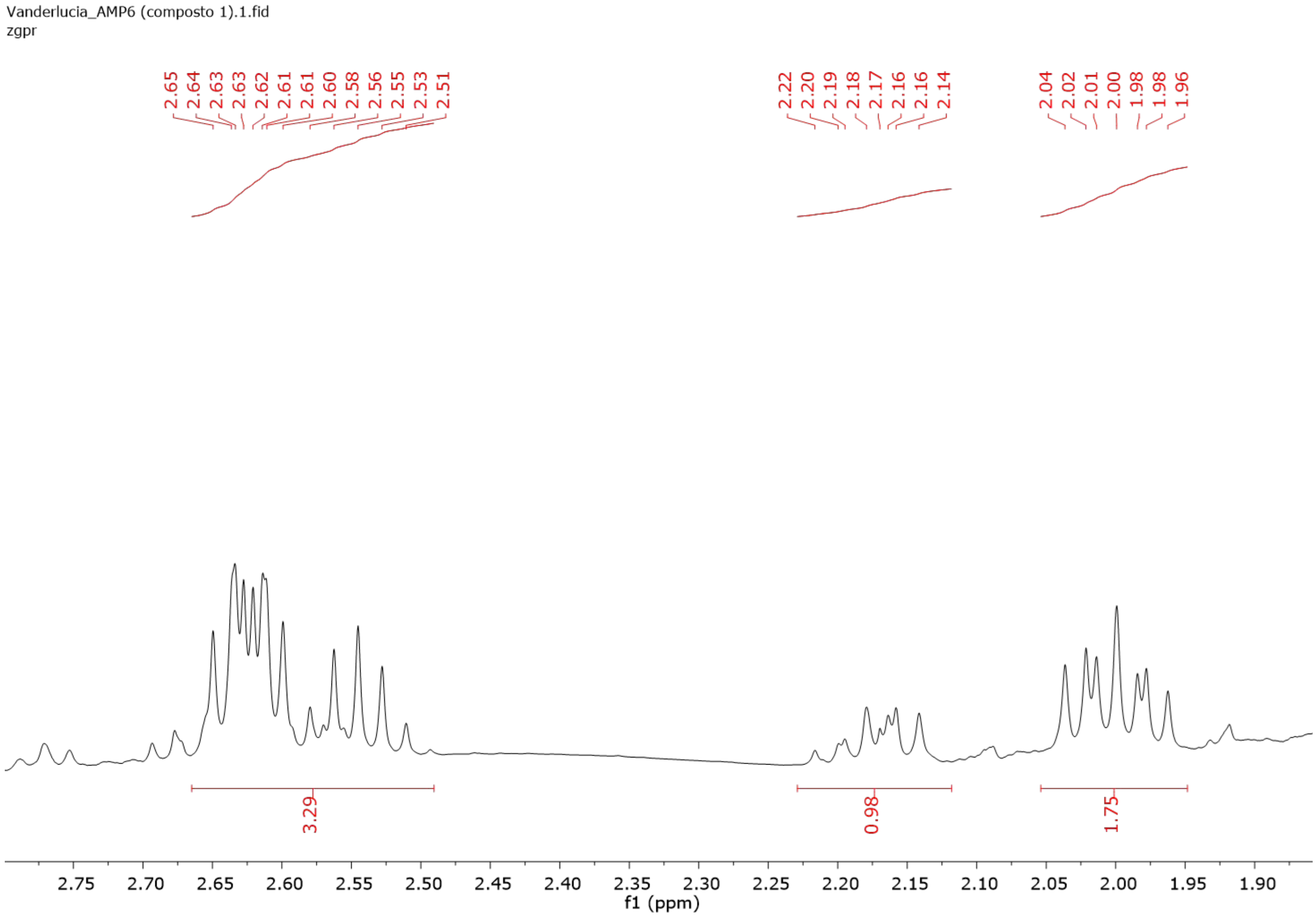

Figure S 3. Expansion 2 of the ^{1}H NMR spectrum of compound **1** (600 MHz, $CDCl_3$)

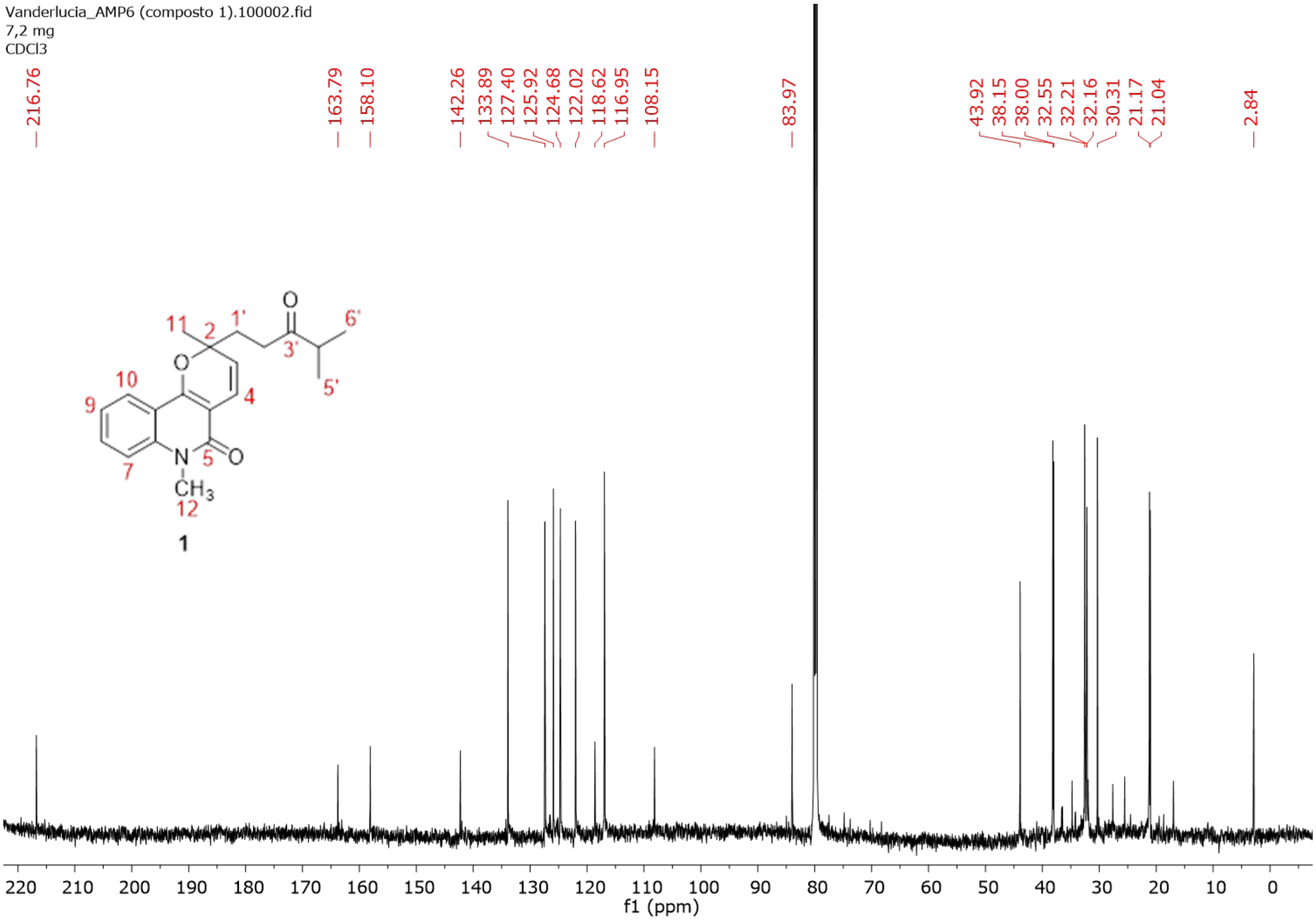

Figure S 4. ^{13}C NMR spectrum of compound **1** (150 MHz, $CDCl_3$)

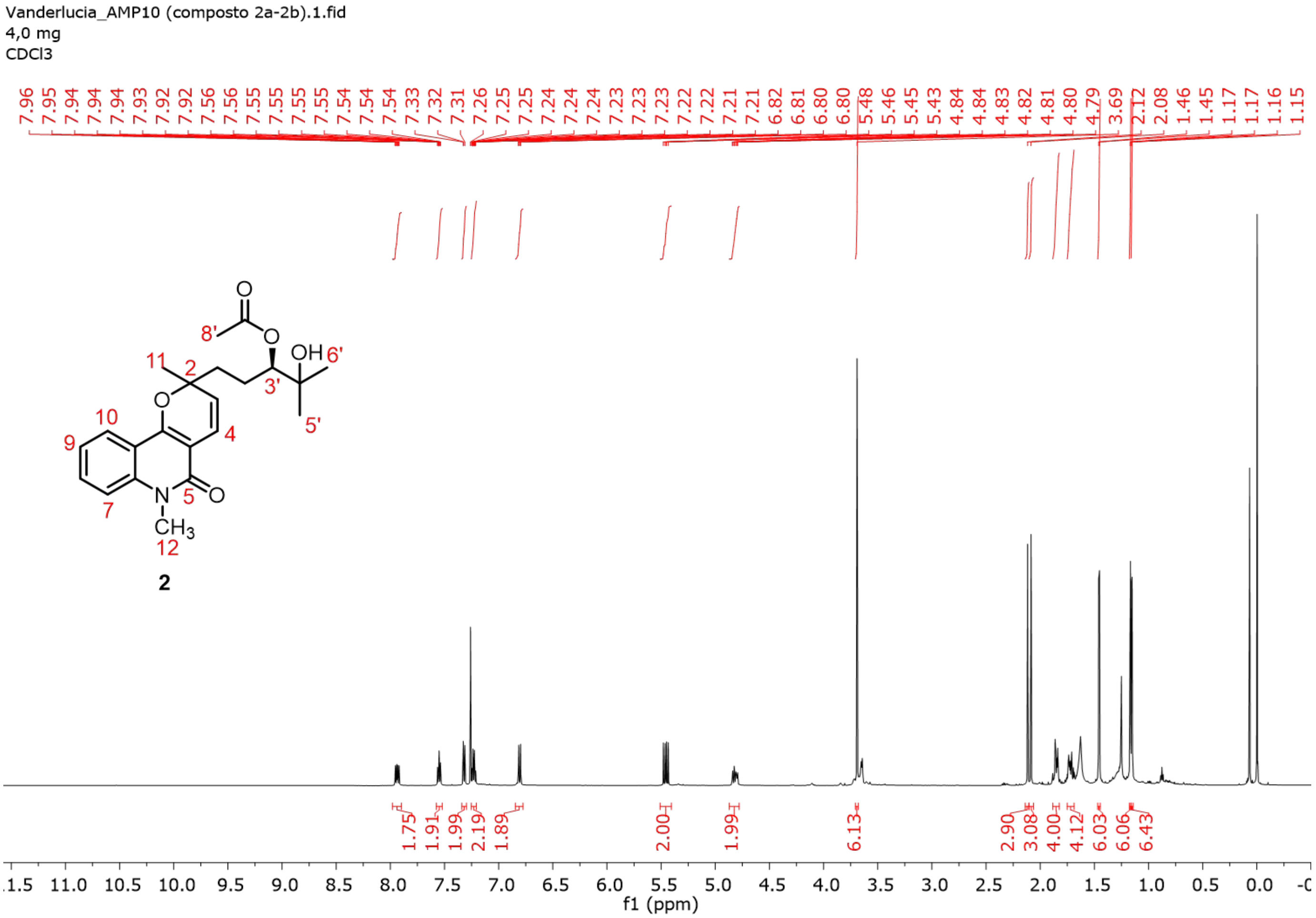

Figure S 5. ^{1}H NMR spectrum of compound **2** (400 MHz, $CDCl_3$)

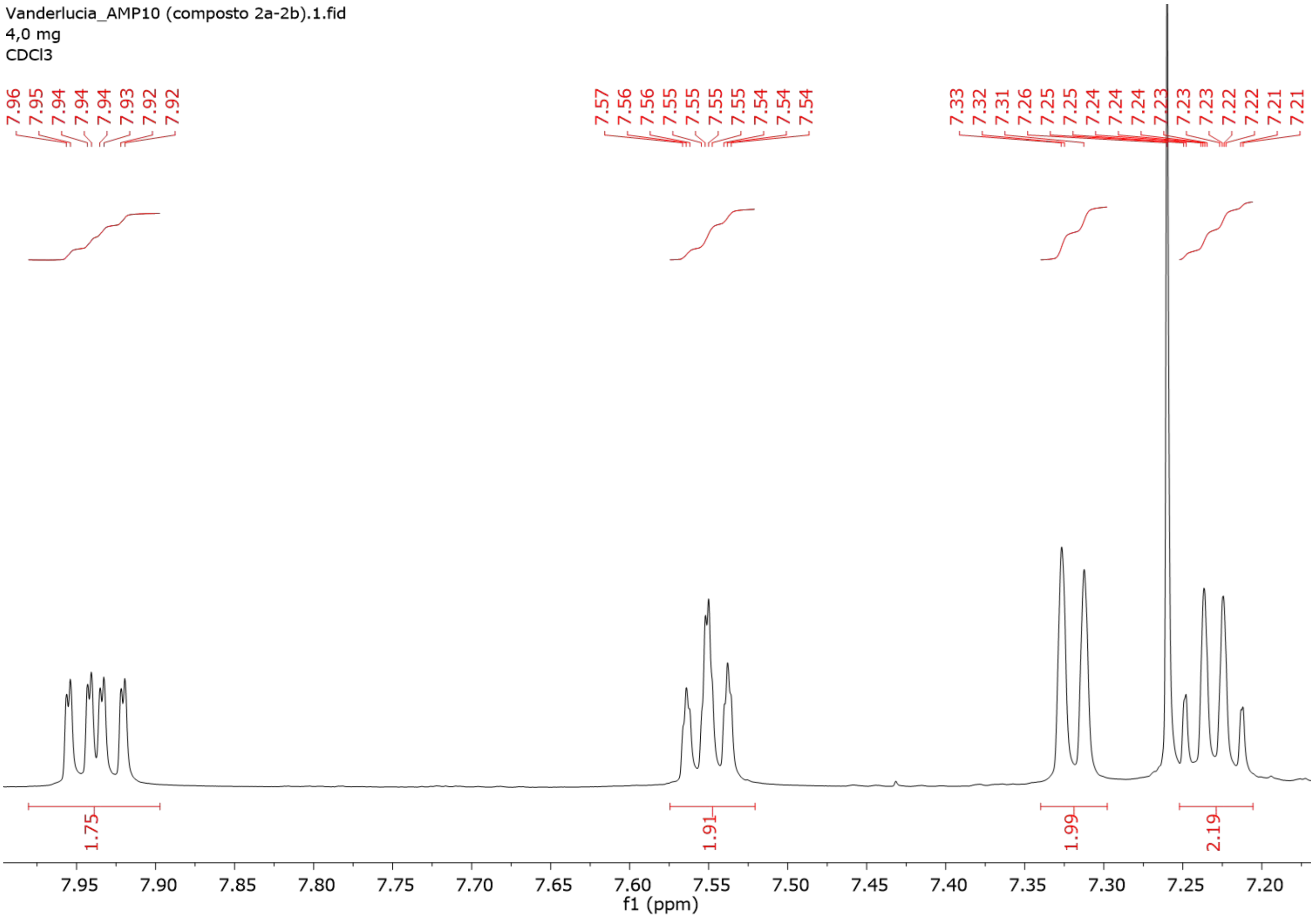

Figure S 6. Expansion 1 of the ^{1}H NMR spectrum of compound **2** (400 MHz, $CDCl_3$)

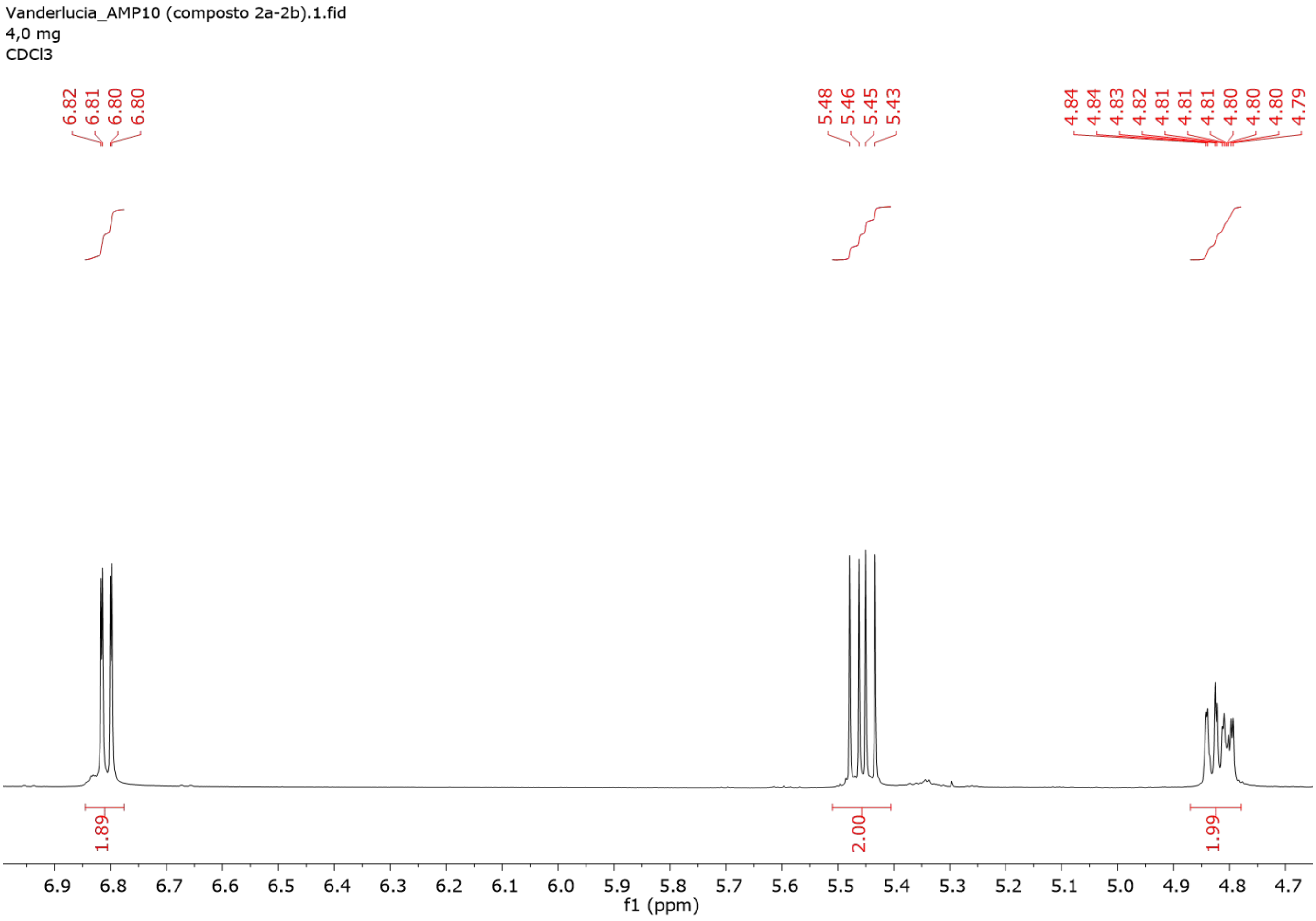

Figure S 7. Expansion 2 of the ^{1}H NMR spectrum of compound **2** (400 MHz, $CDCl_3$)

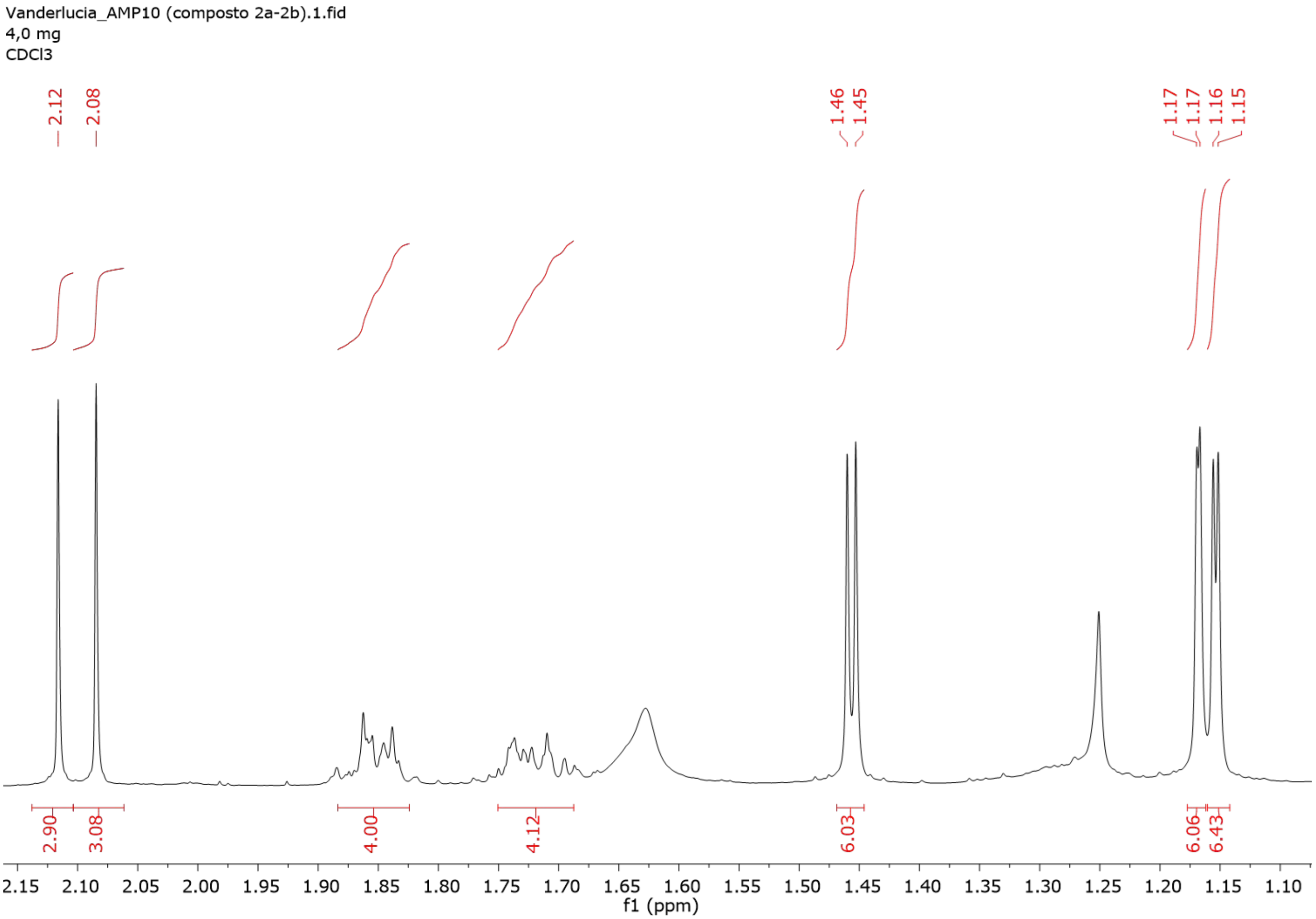

Figure S 8. Expansion 3 of the ^{1}H NMR spectrum of compound **2** (400 MHz, $CDCl_3$)

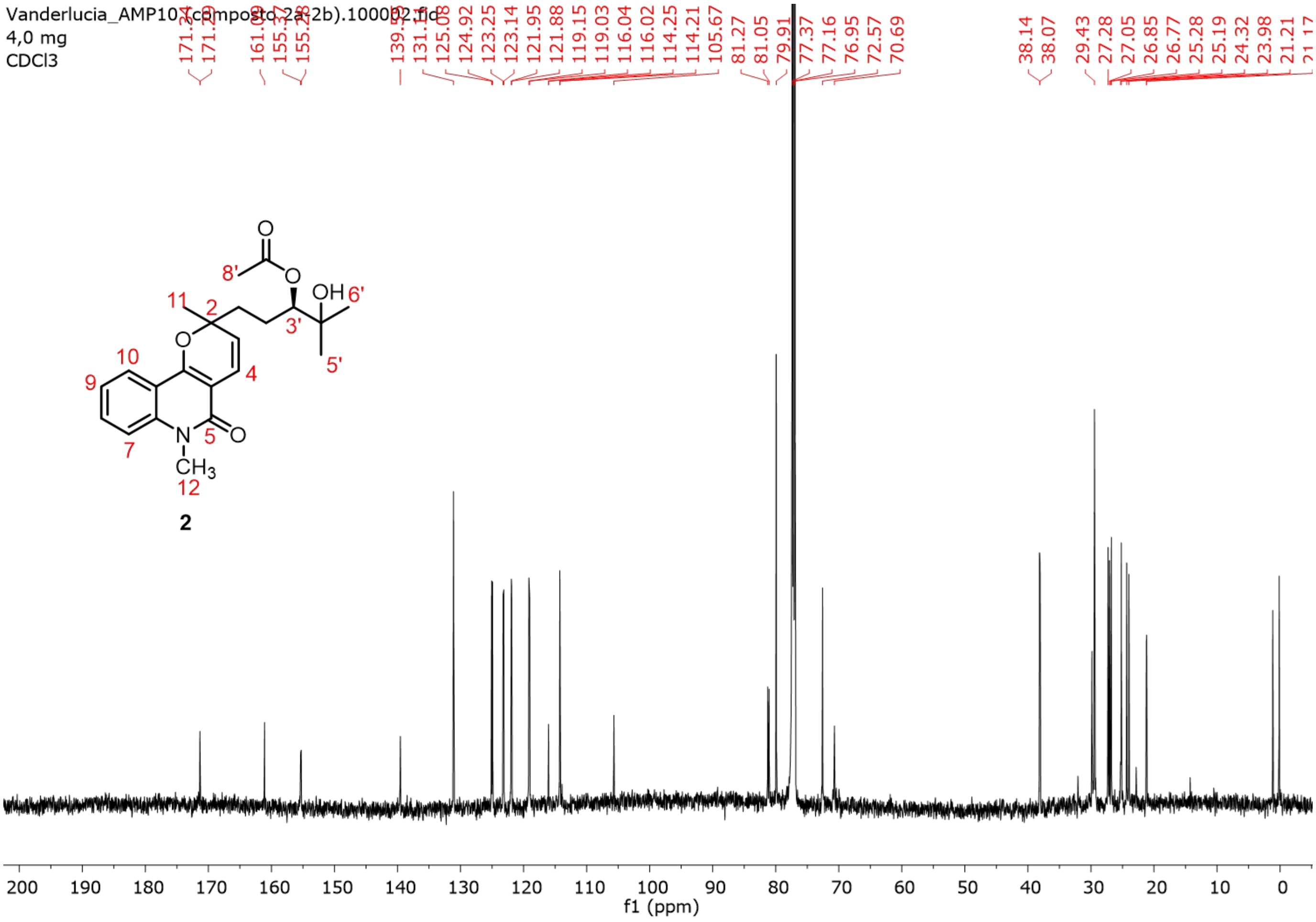

Figure S 9. ^{13}C NMR spectrum of compound **2** (150 MHz, $CDCl_3$)

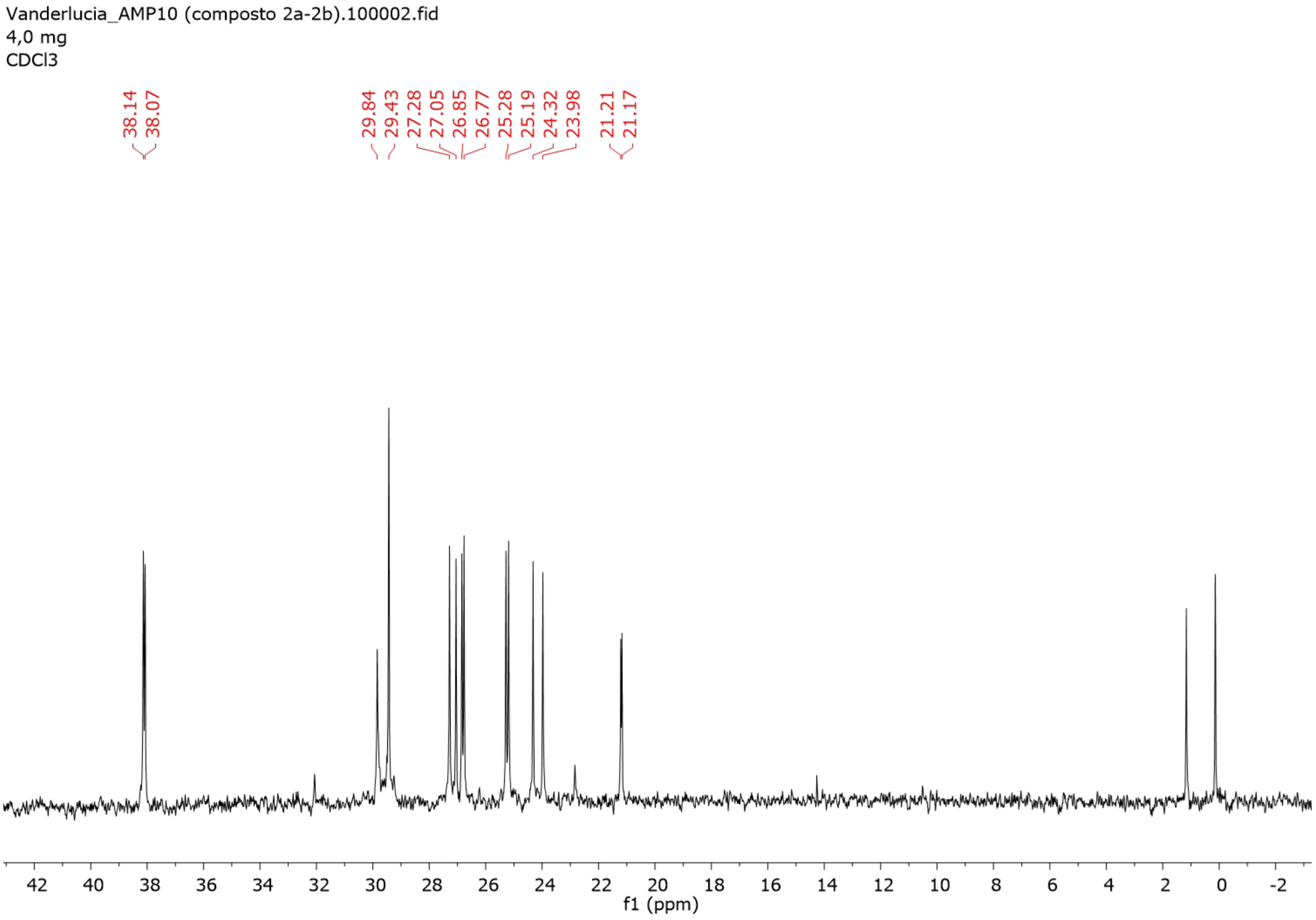

Figure S 10. Expansion of the ^{13}C NMR spectrum of compound **2** (150 MHz, $CDCl_3$)

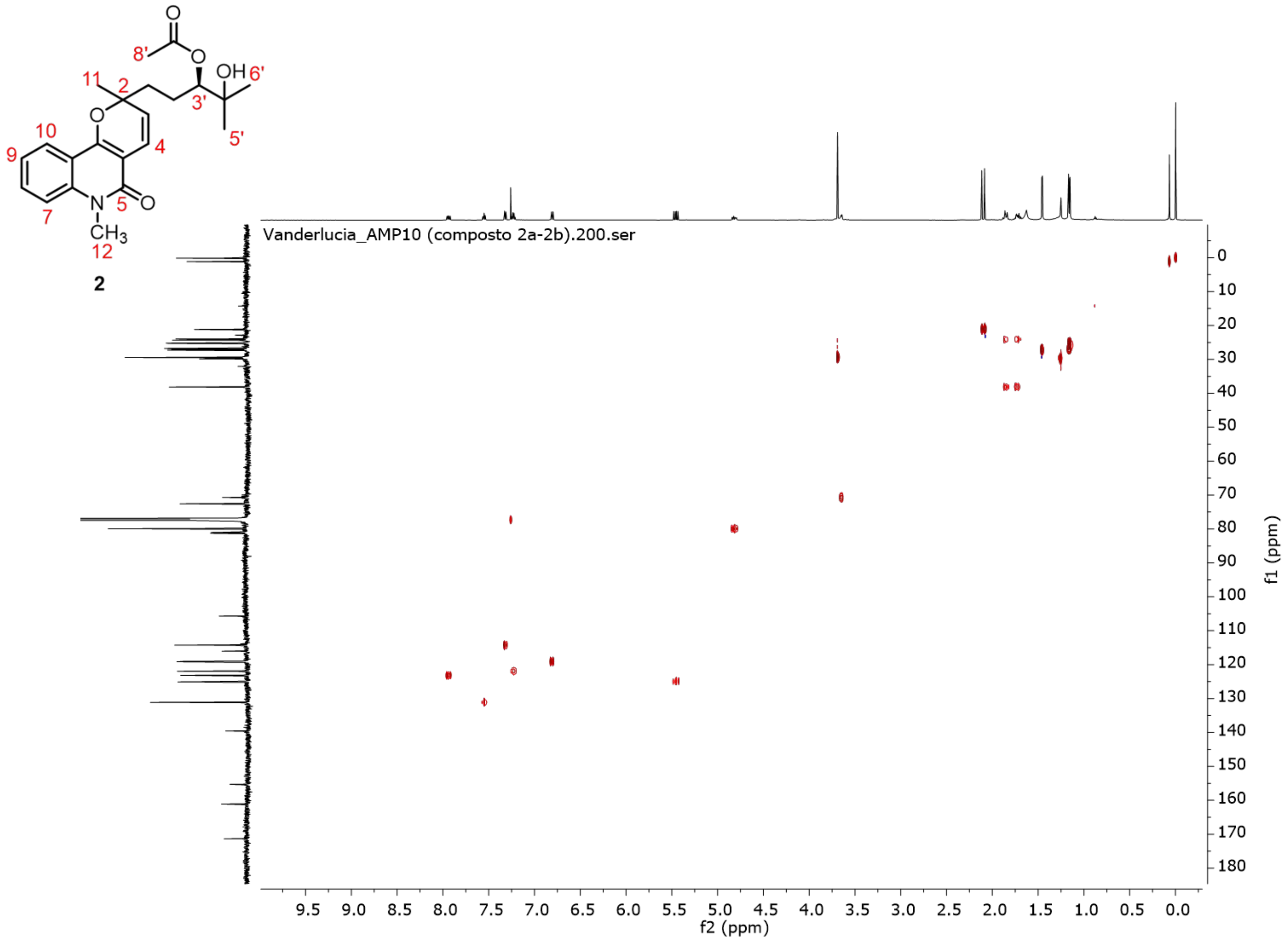

Figure S 11. HSQC spectrum of compound **2** (^{1}H: 600 MHz, ^{13}C: 150 MHz, $CDCl_3$)

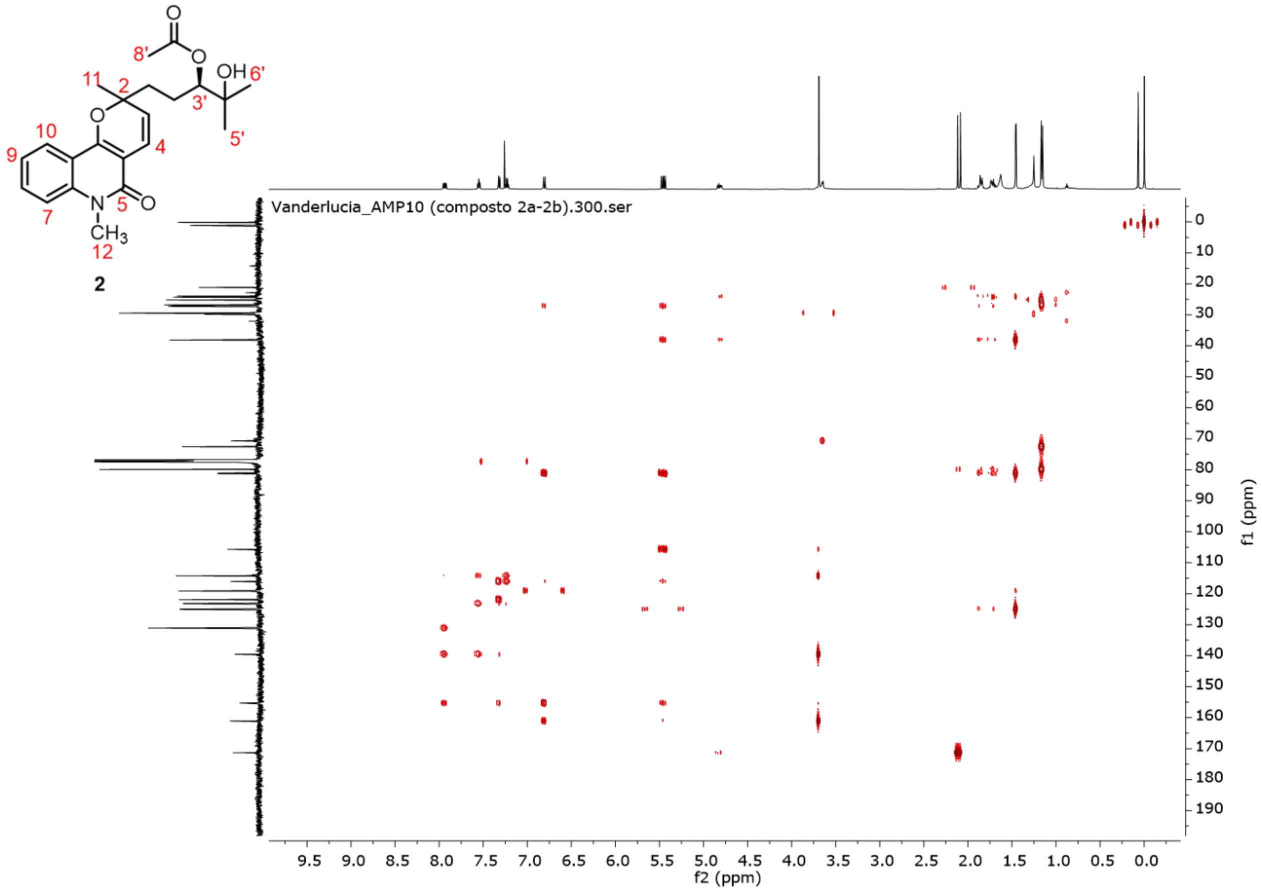

Figure S 12. HMBC spectrum of compound **2** (^{1}H: 600 MHz, ^{13}C: 150 MHz, $CDCl_3$)

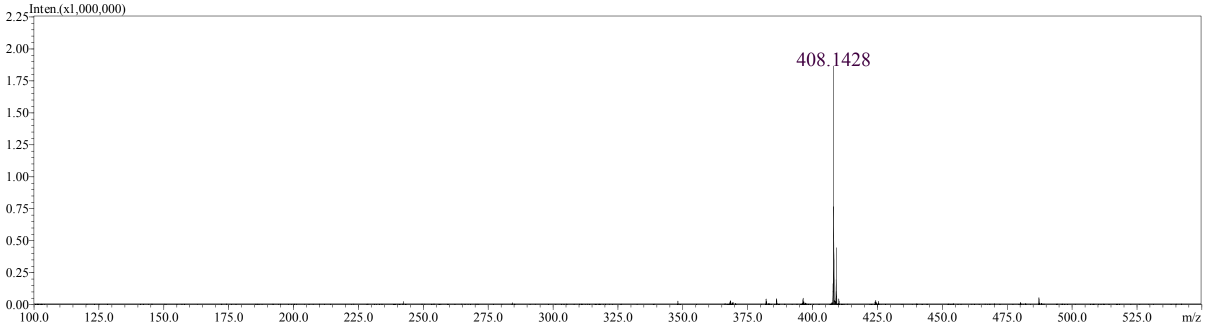

Figure S 13. (+)-HRESIMS spectrum of compound **2** (The discrepancy between the calculated and observed *m/z* values for $[C_{22}H_{27}NO_5Na]^+$ (difference = +0,0359 u) was attributed to poor calibration during spectra acquisition. The molecular formula of compound **2** was further confirmed by HRMS data obtained for the synthetic compounds **2*α*** and **2*β***, and also by spectroscopic data (^{1}H and ^{13}C NMR, IR and UV-vis), which revealed them to be identical to **2**).

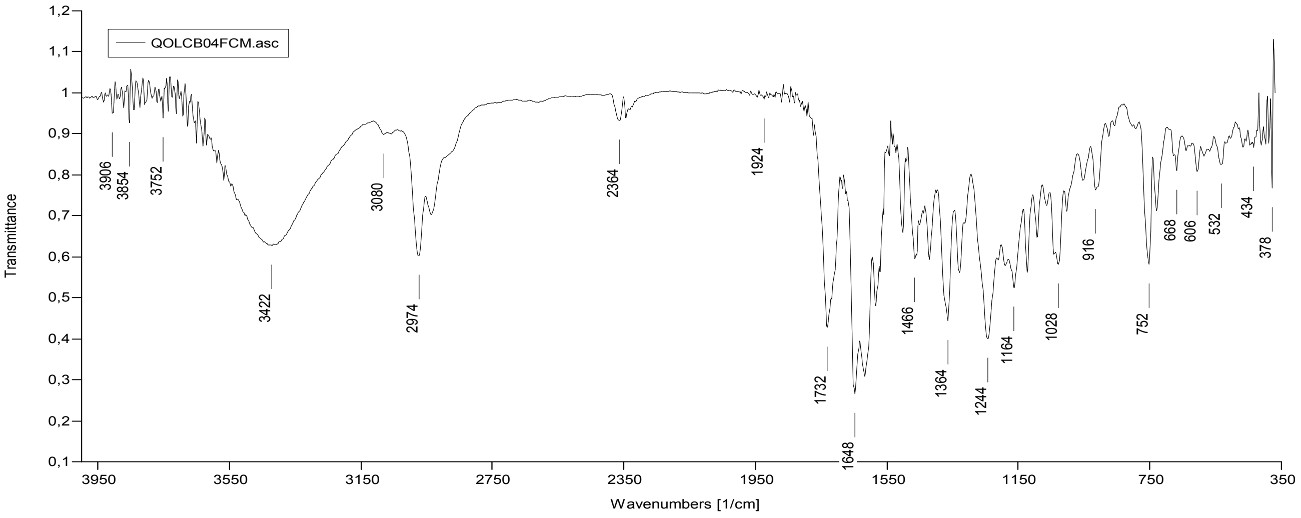

Figure S 14 - IR spectrum of compound **2**

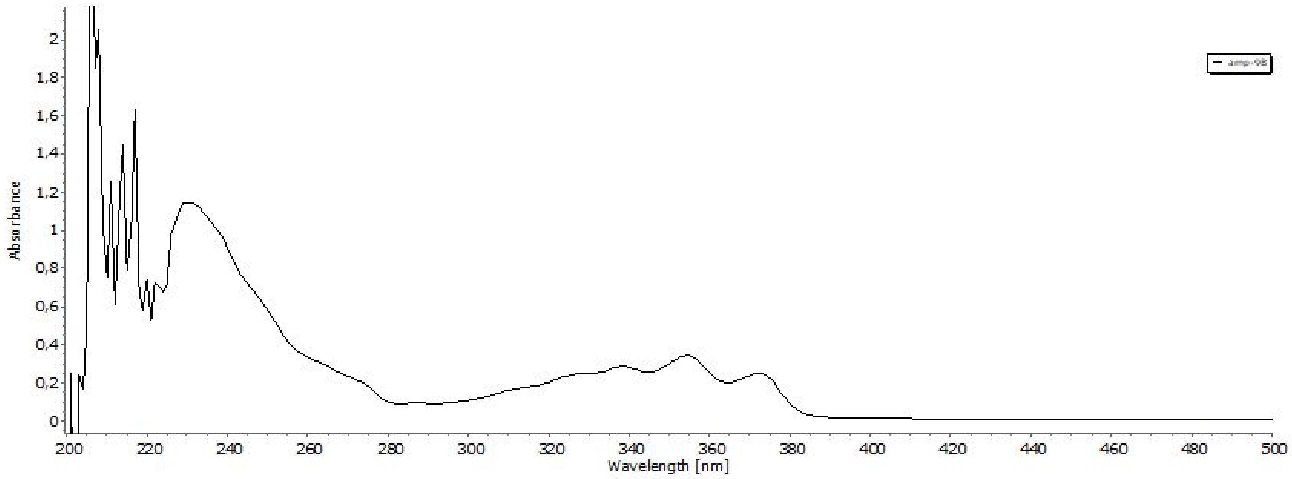

Figure S 15. UV Spectrum (16 µg/mL, CH_2Cl_2) of compound **2**

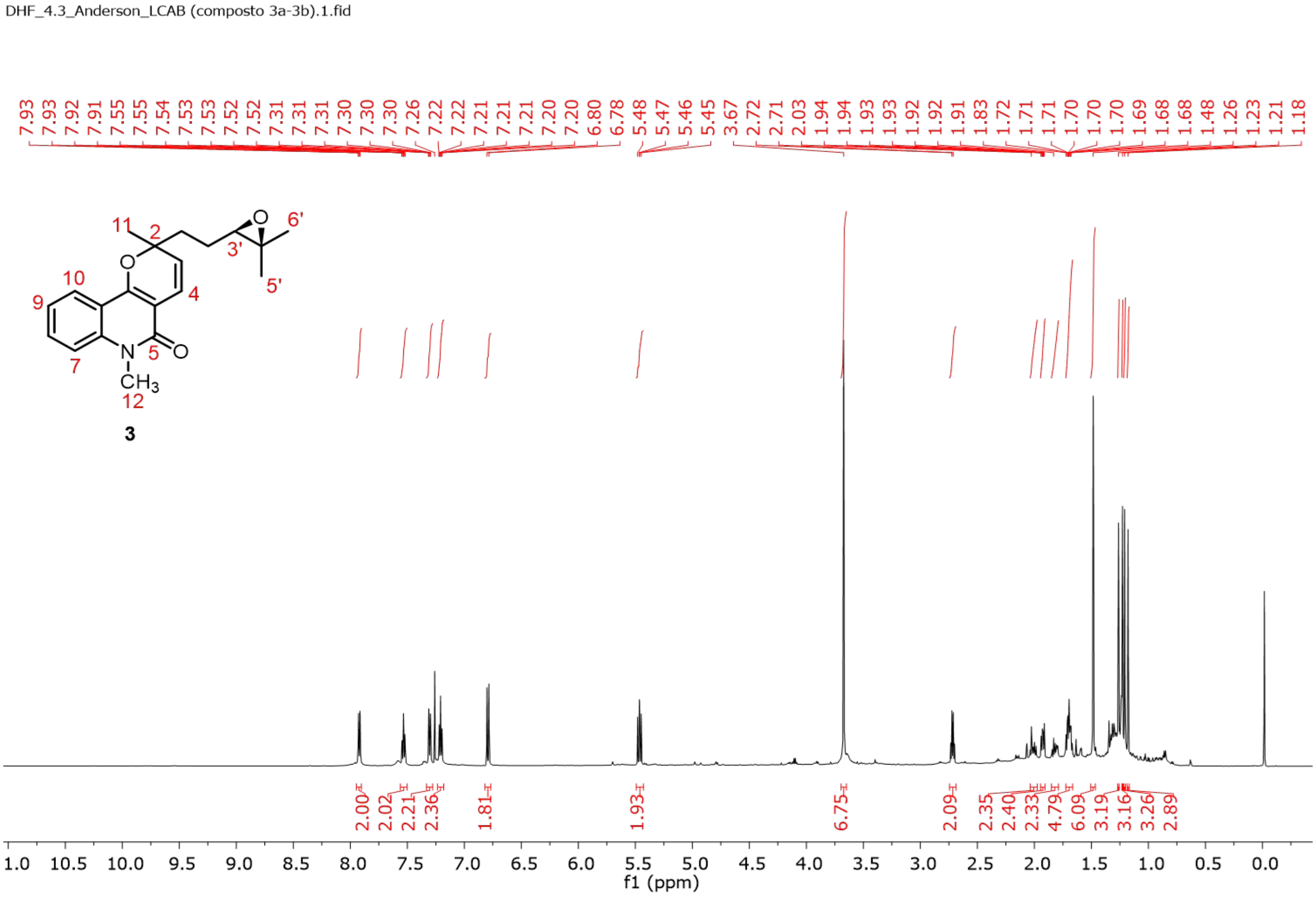

Figure S 16. ^{1}H NMR spectrum of compound **3** (400 MHz, $CDCl_3$)

DHF_4.3_Anderson_LCAB (composto 3a-3b).1.fid

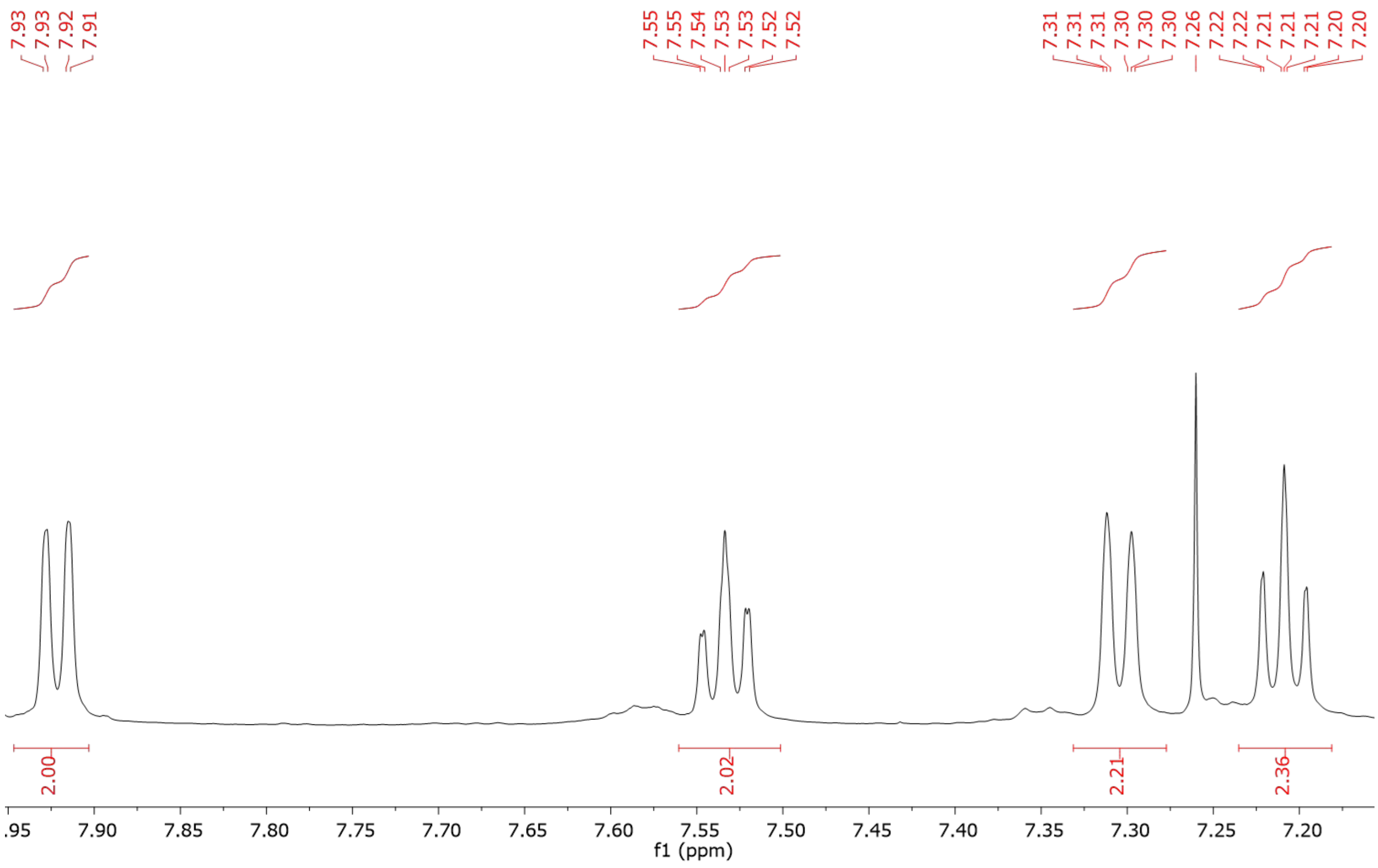

Figure S 17. Expansion 1 of the ^{1}H NMR spectrum of compound **3** (400 MHz, $CDCl_3$)

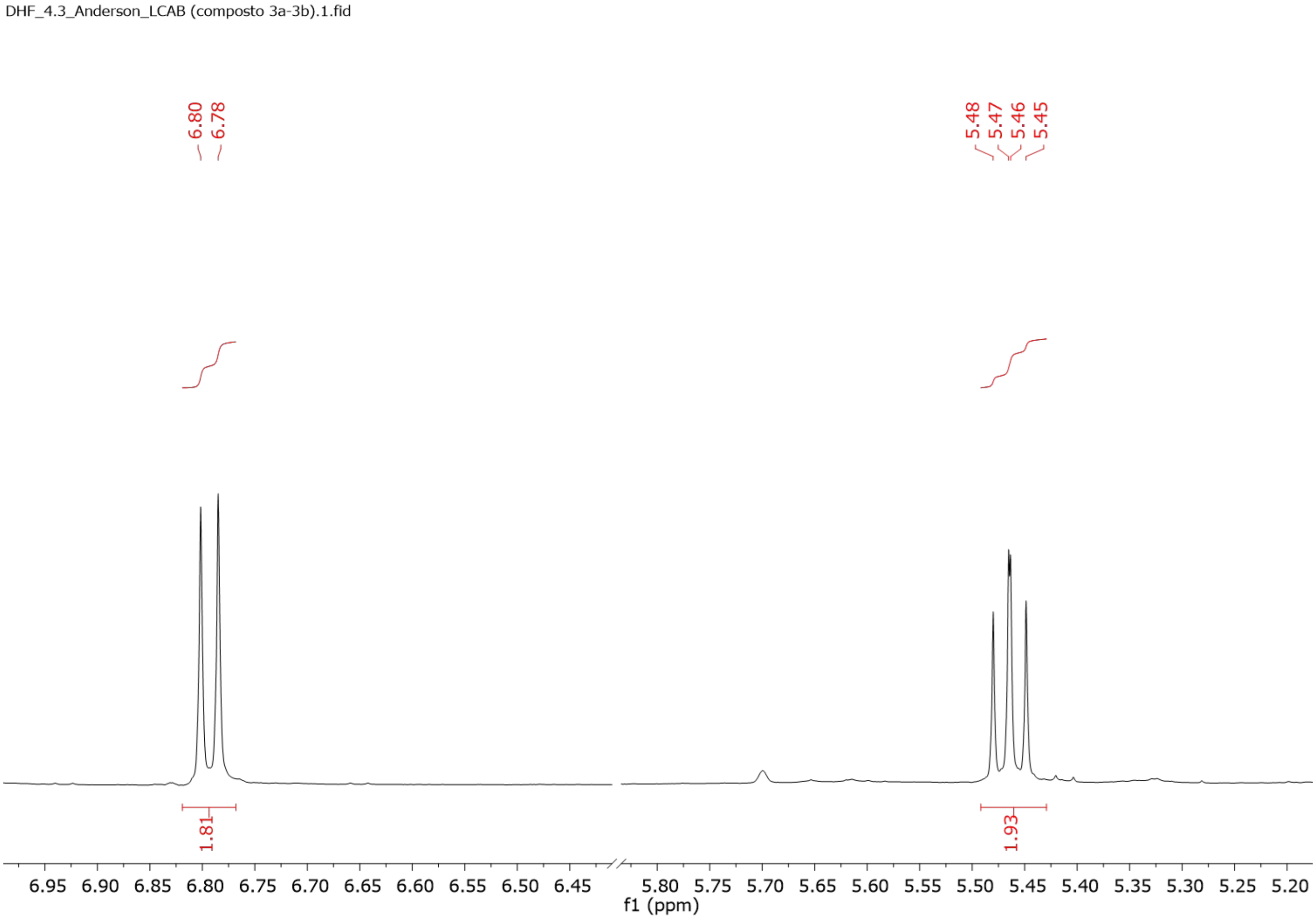

Figure S 18. Expansion 2 of ^{1}H NMR spectrum of compound **3** (400 MHz, $CDCl_3$)

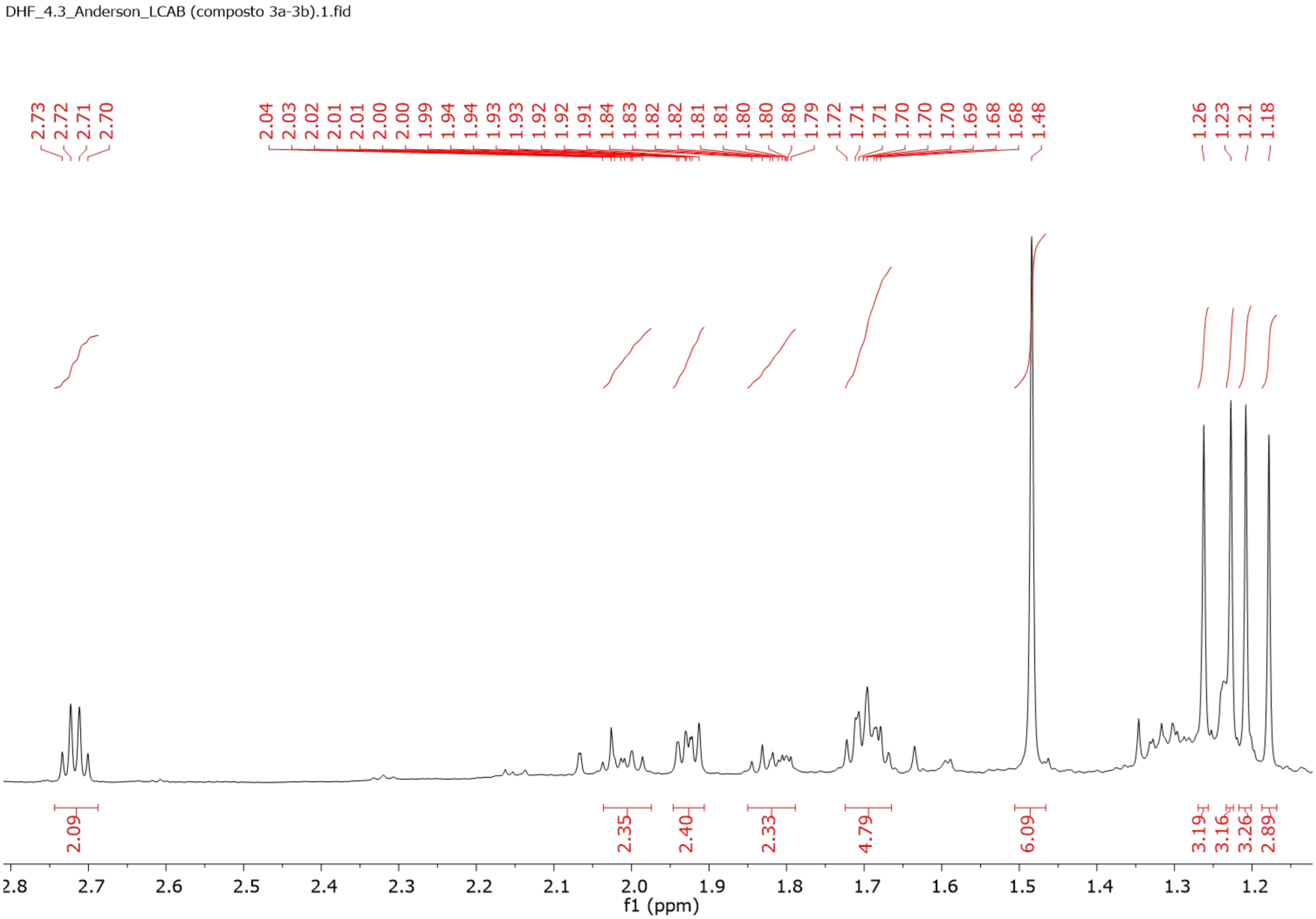

Figure S 19. Expansion 3 of ^{1}H NMR spectrum of compound **3** (400 MHz, $CDCl_3$)

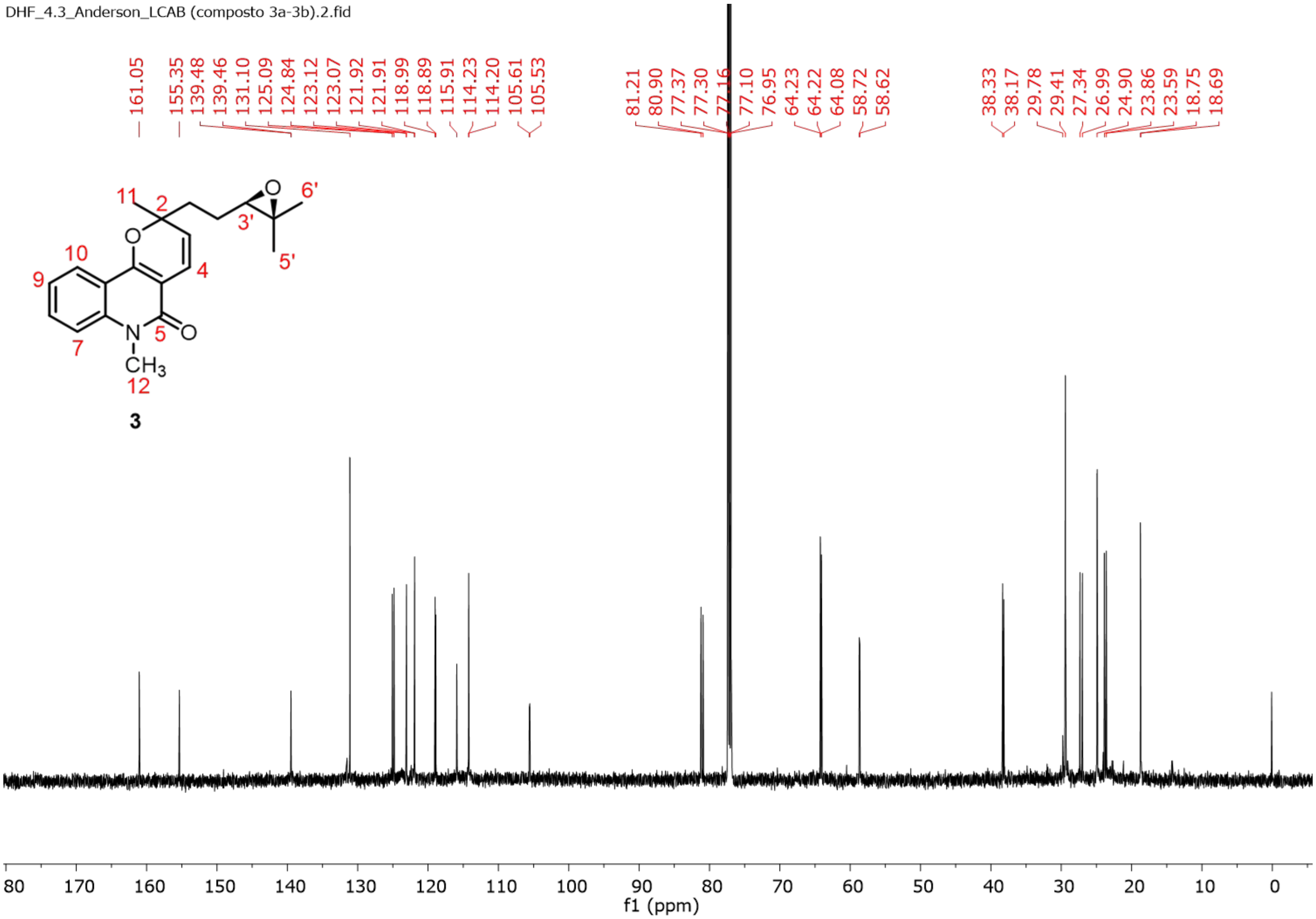

Figure S 20. ^{13}C NMR spectrum of compound **3** (100 MHz, $CDCl_3$)

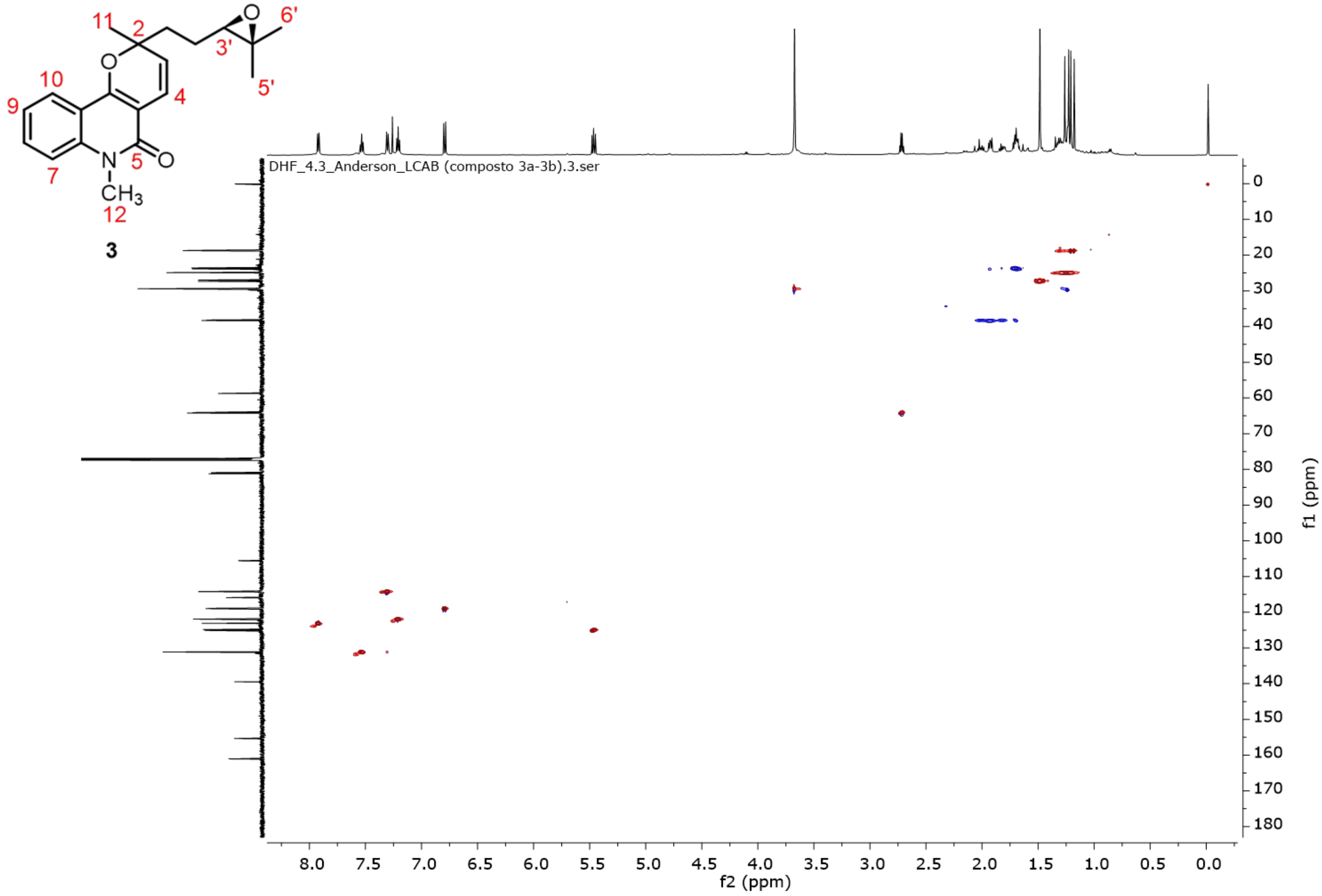

Figure S 21. HSQC spectrum of compound **3** (^{1}H: 400 MHz, ^{13}C: 100 MHz, $CDCl_3$)

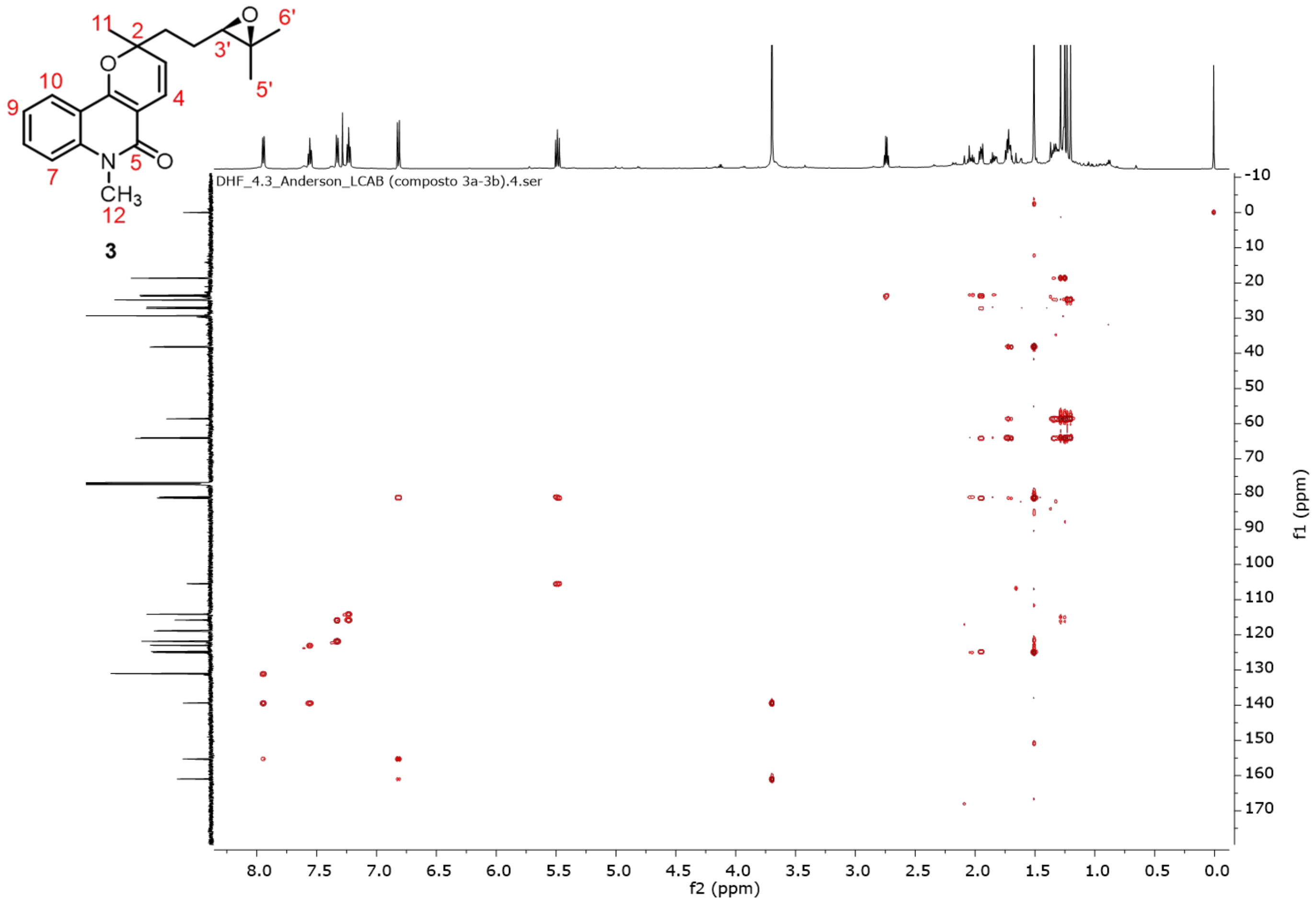

Figure S 22. HMBC spectrum of compound **3** (^{1}H: 400 MHz, ^{13}C: 100 MHz, $CDCl_3$)

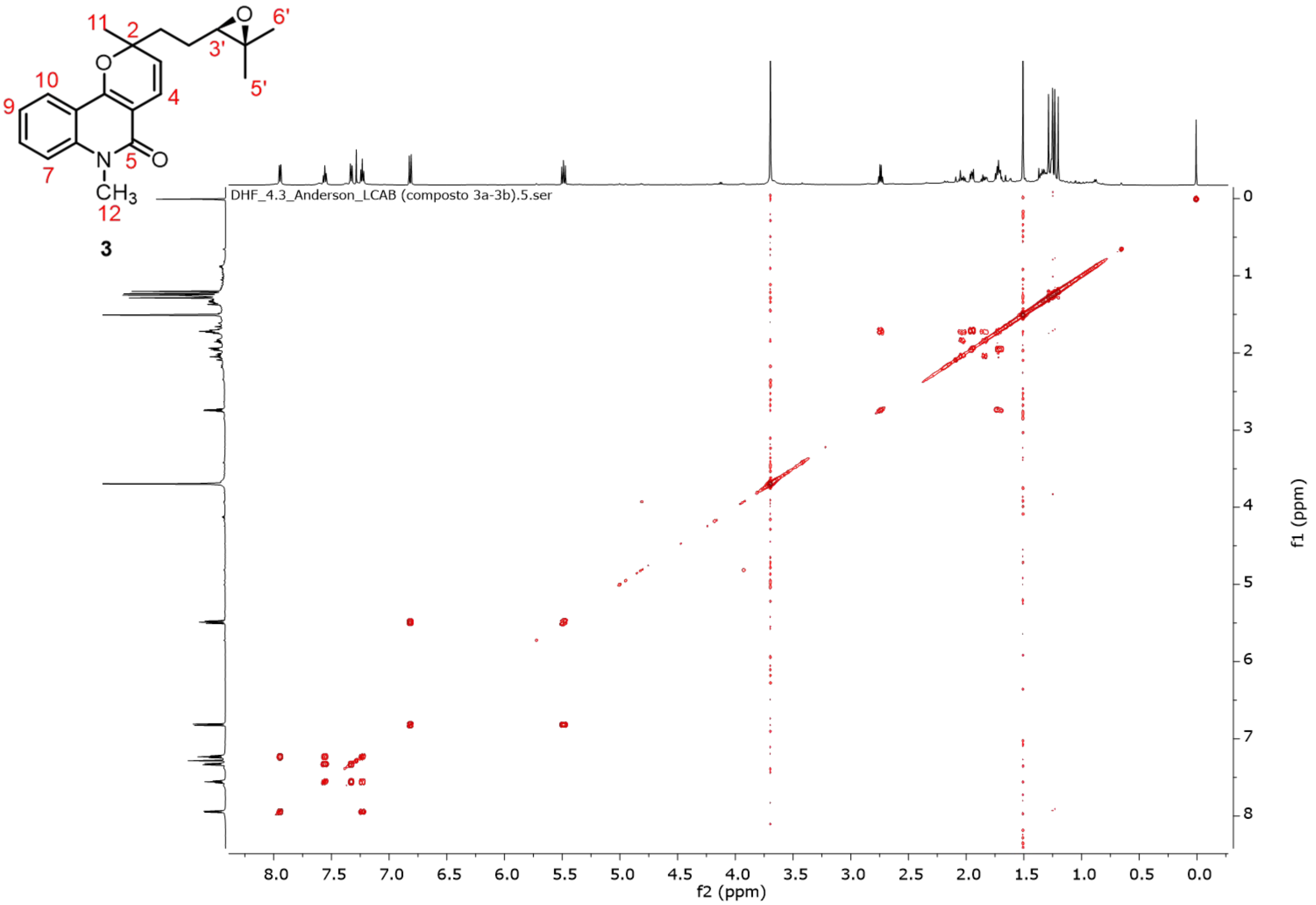

Figure S 23. COSY spectrum of compound **3** (400 MHz, $CDCl_3$)

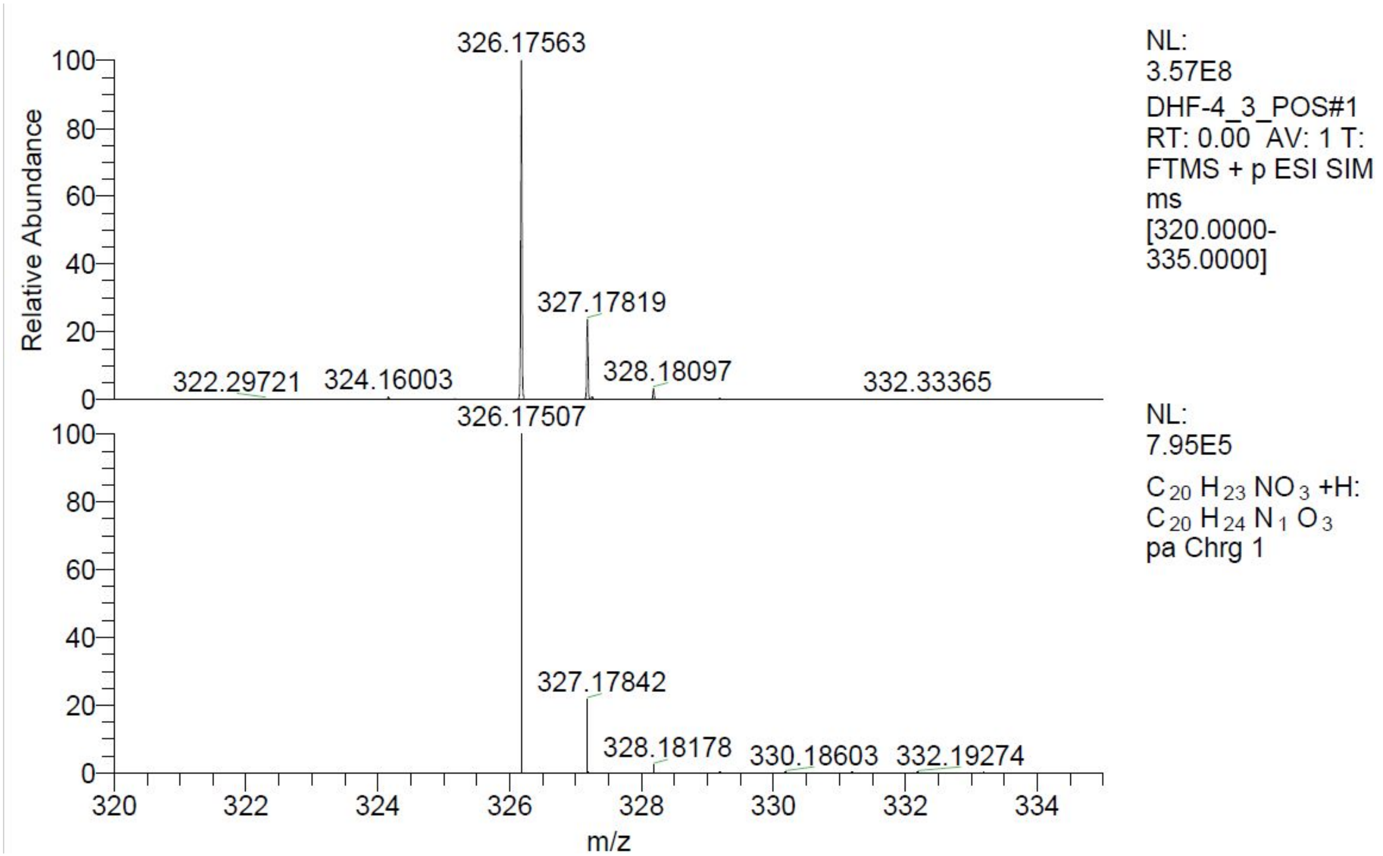

Figure S 24. (+)-HRESIMS spectrum of compound **3**

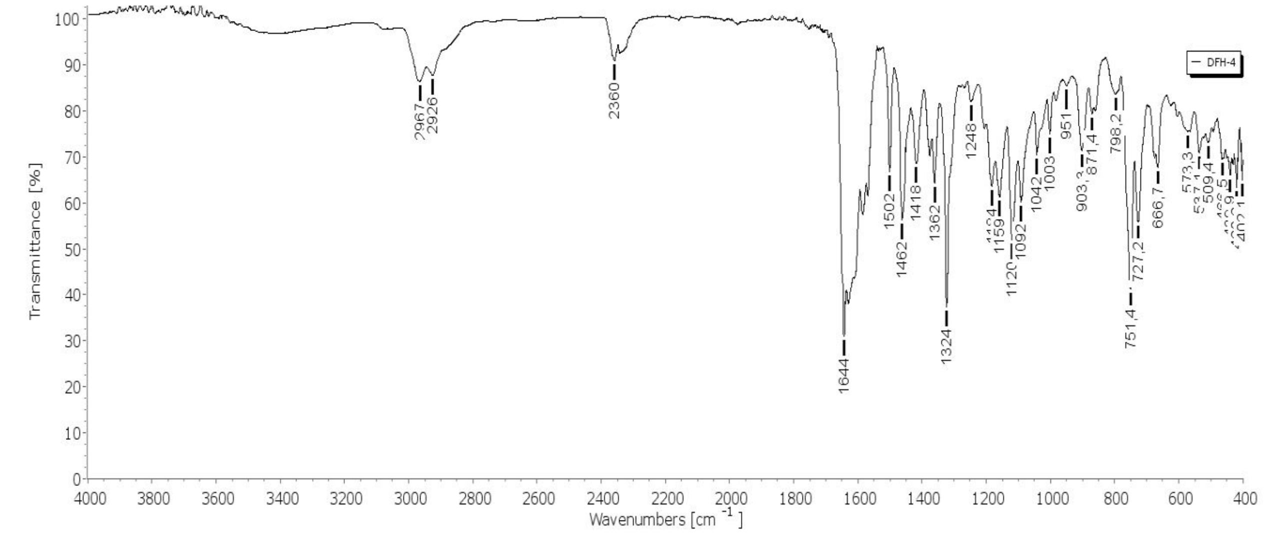

Figure S 25. IR spectrum of compound **3**

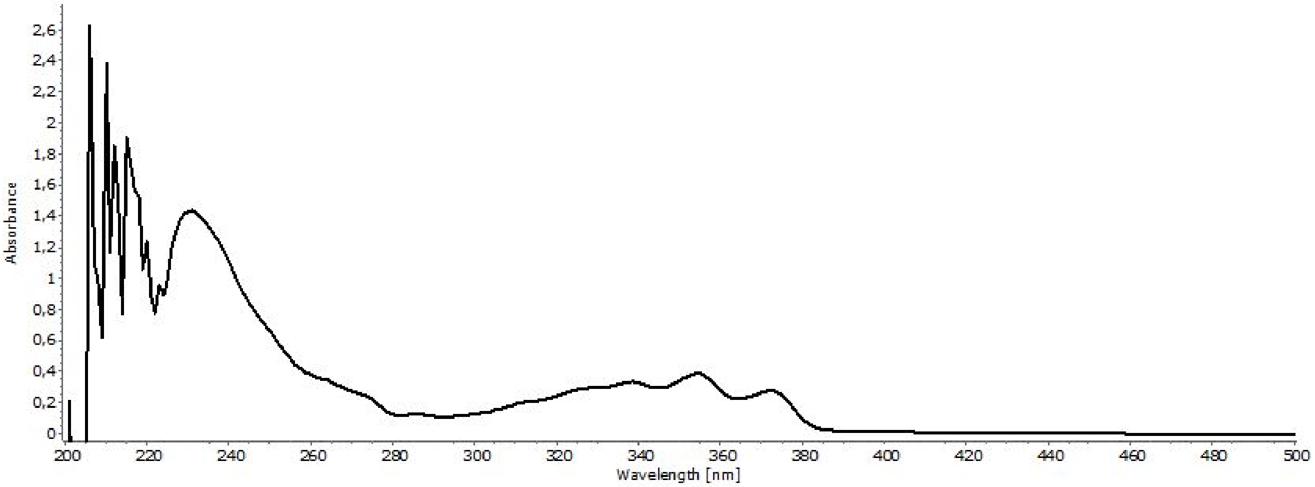

Figure S 26. UV spectrum (15 µg/mL, CH_2Cl_2) of compound **3**

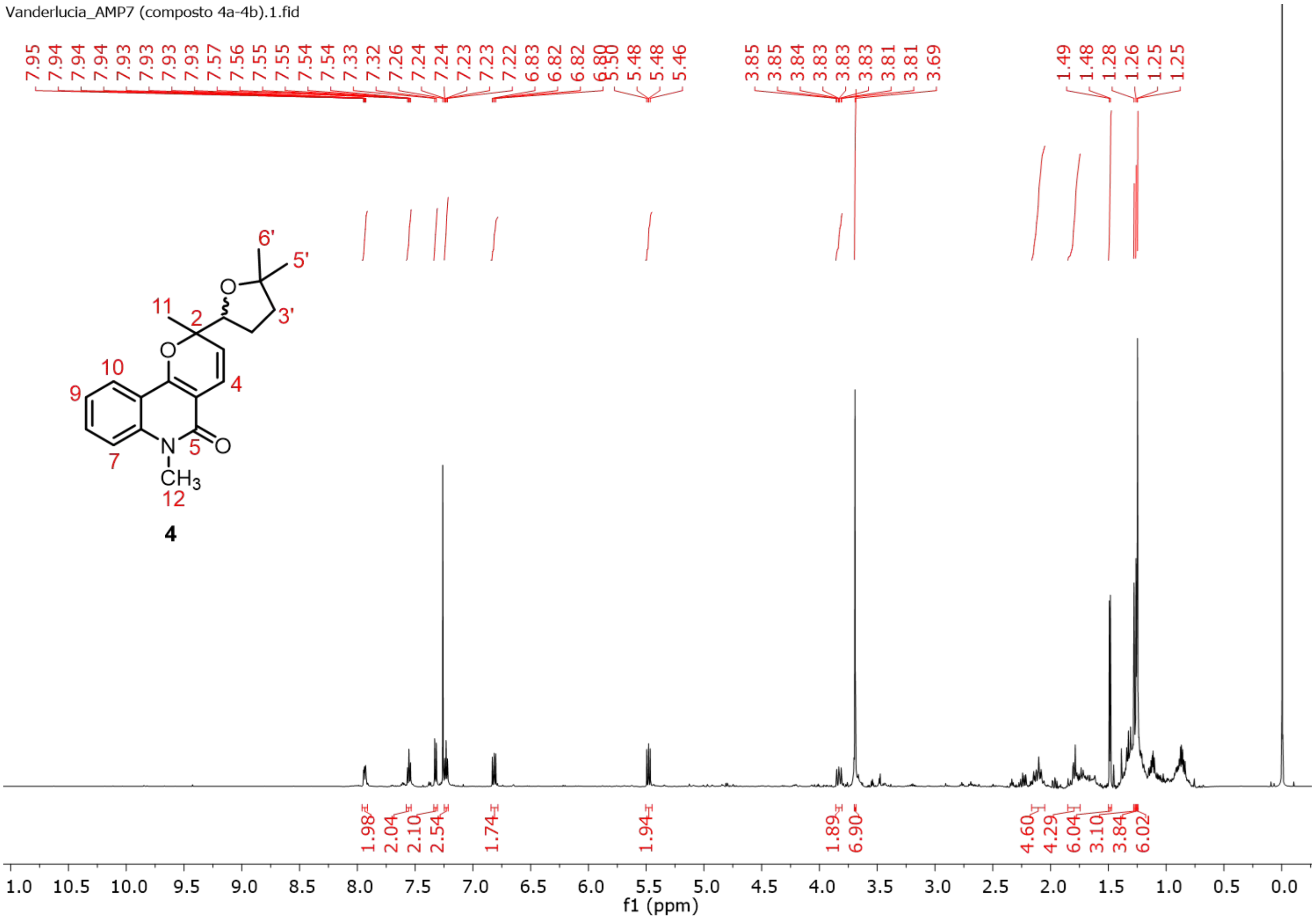

Figure S 27. ^{1}H NMR spectrum of compound **4** (600 MHz, $CDCl_3$)

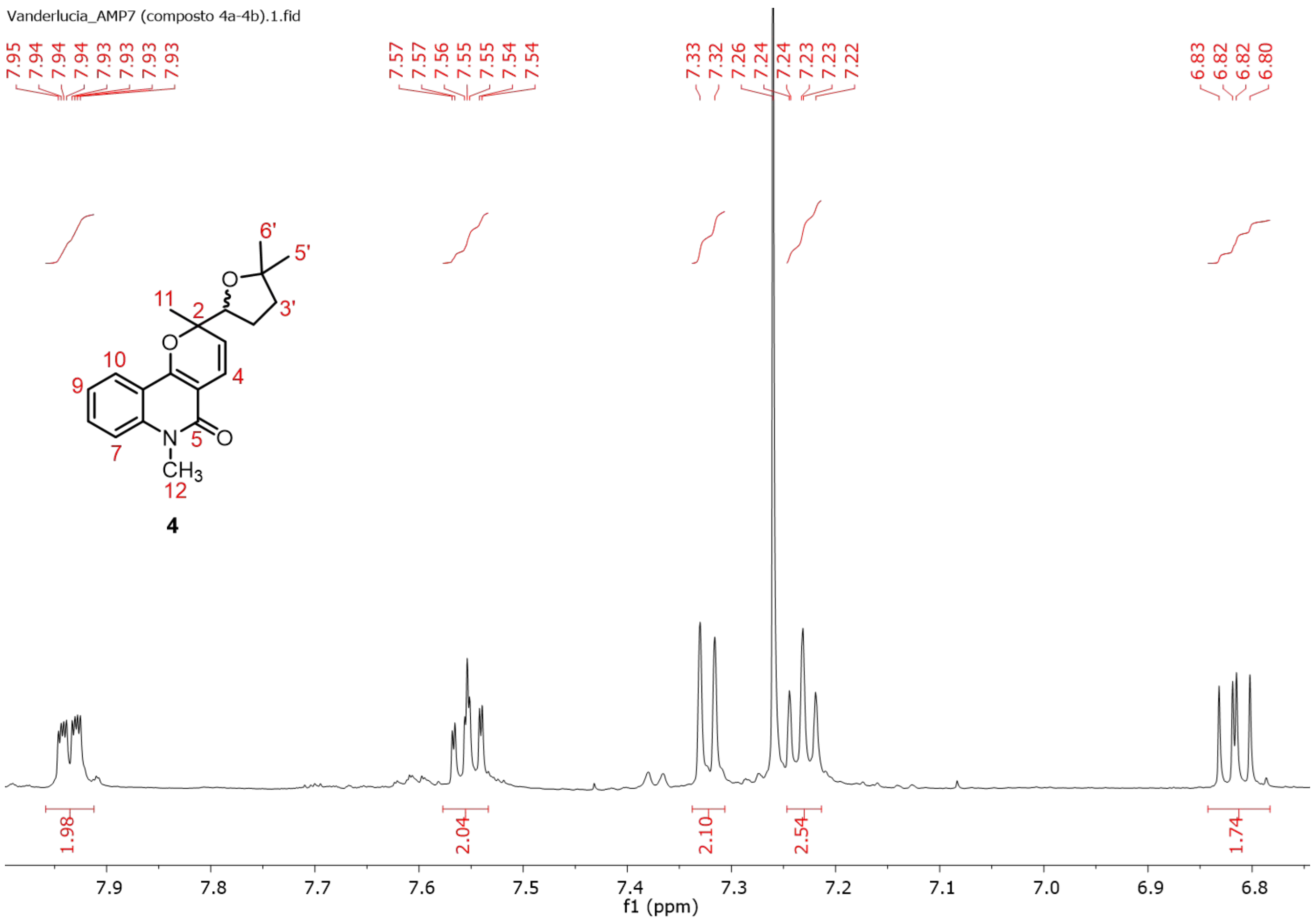

Figure S 28. Expansion 1 of the ^{1}H NMR spectrum of compound **4** (600 MHz, $CDCl_3$)

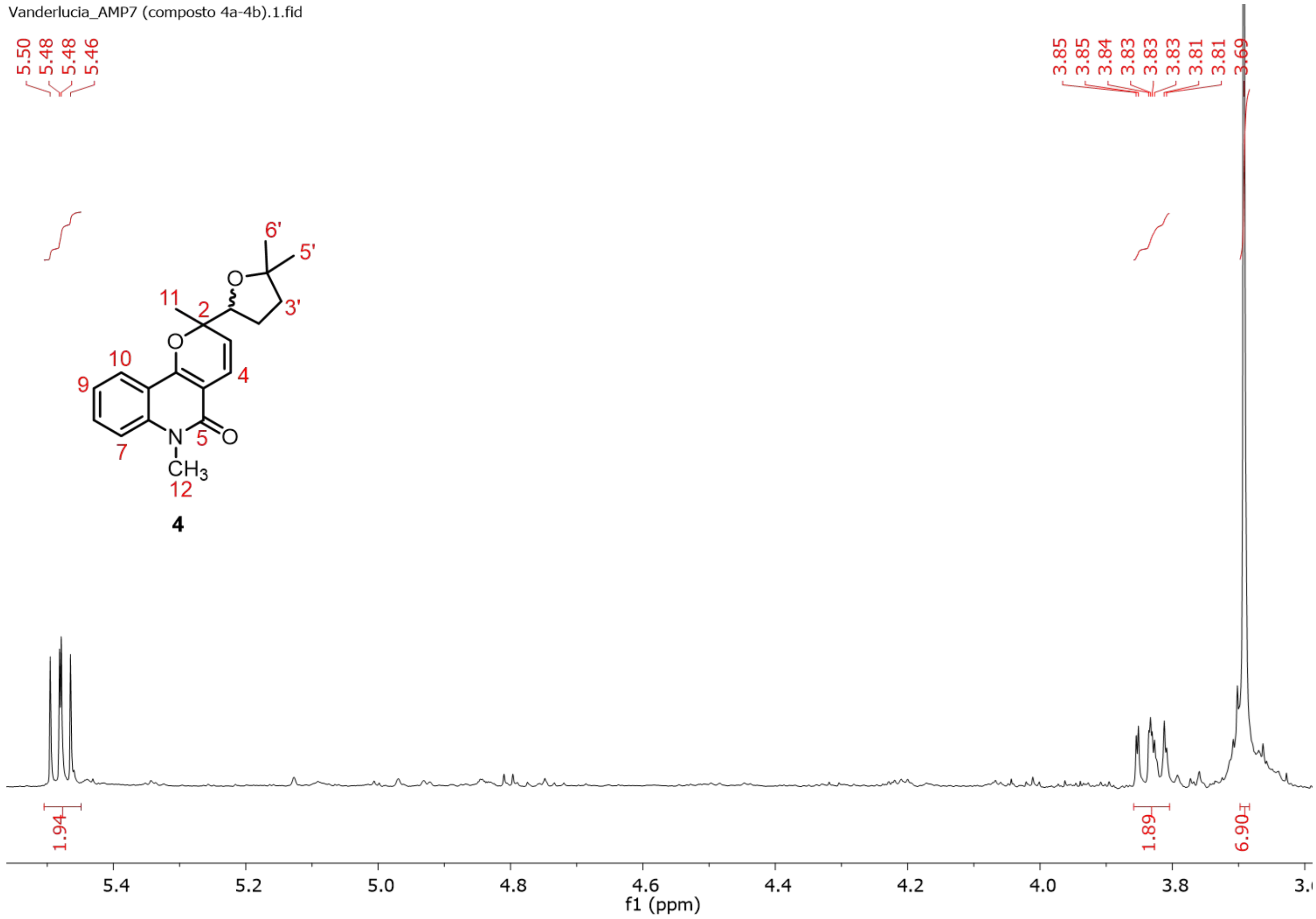

Figure S 29. Expansion 2 of the ^{1}H NMR spectrum of compound **4** (600 MHz, $CDCl_3$)

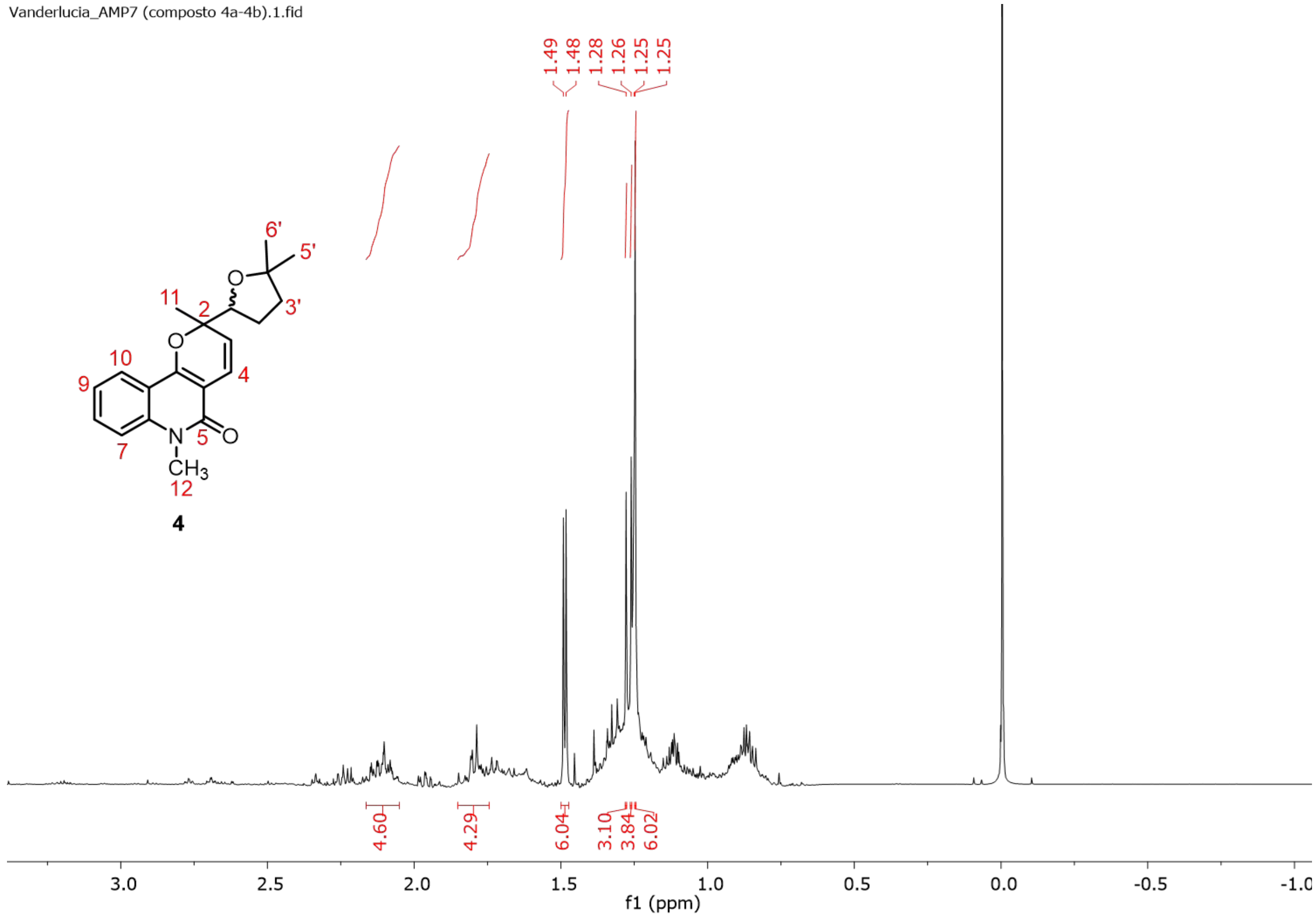

Figure S 30. Expansion 3 of the ^{1}H NMR spectrum of compound **4** (600 MHz, $CDCl_3$)

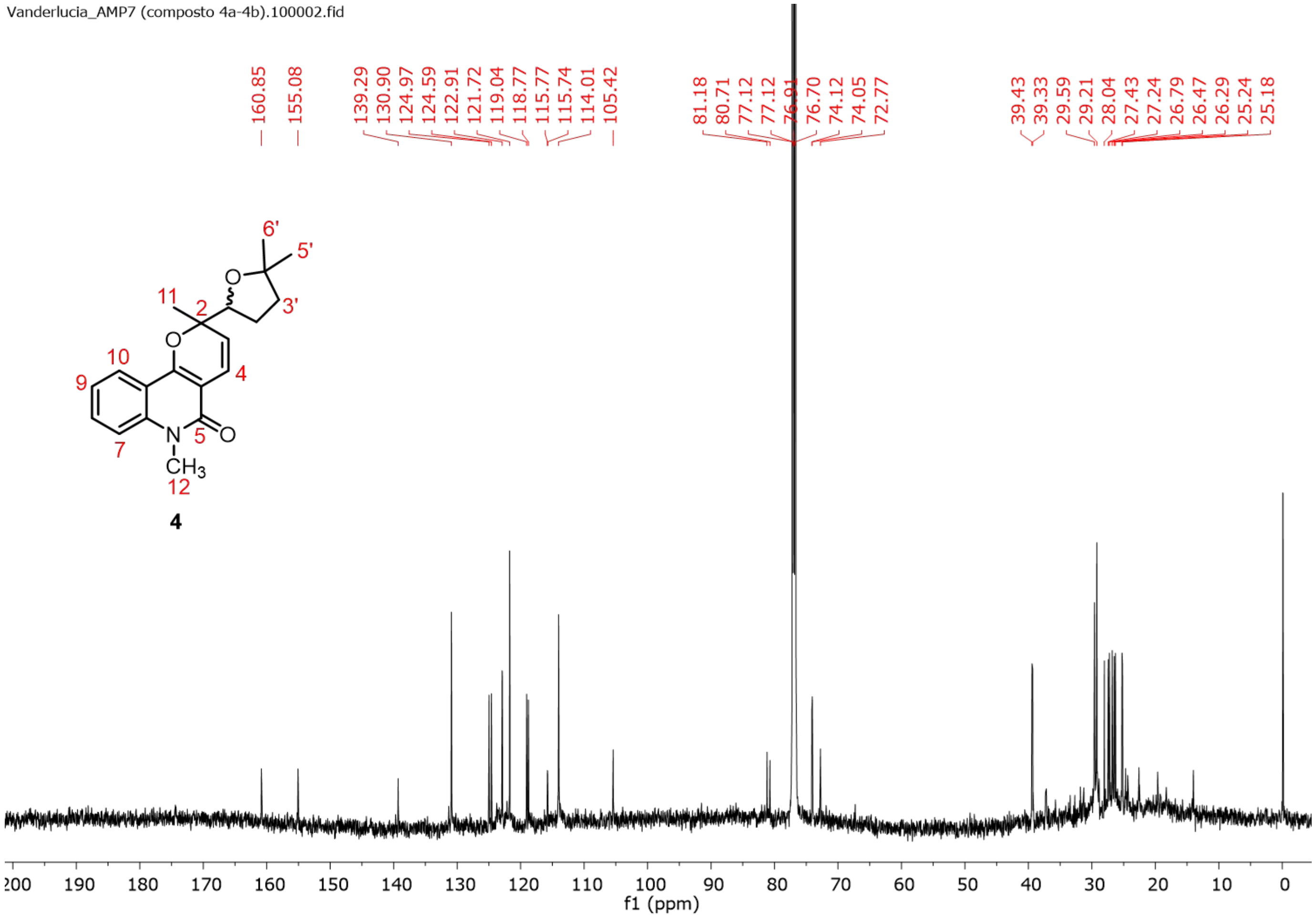

Figure S 31. ^{13}C NMR spectrum of compound **4** (150 MHz, $CDCl_3$)

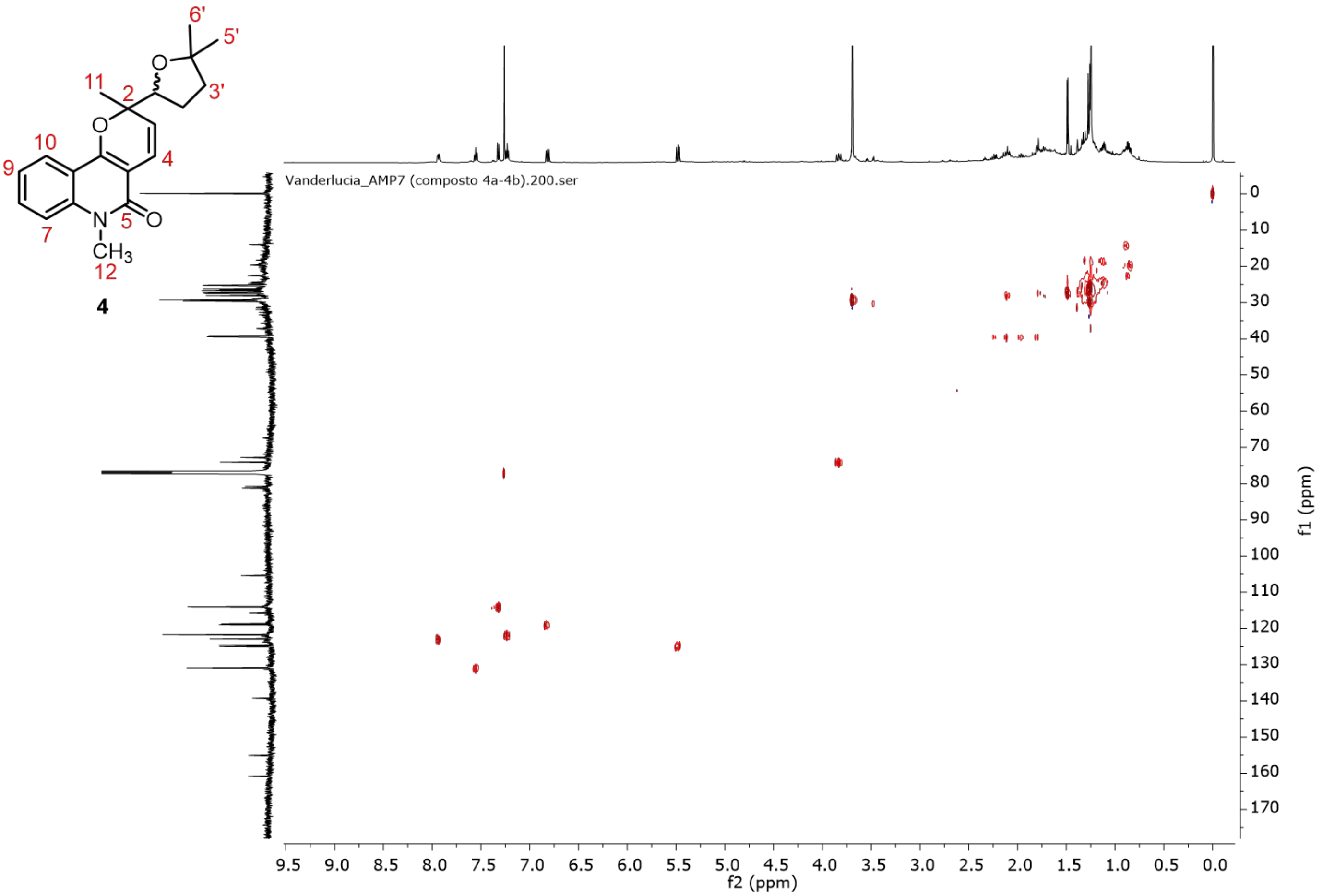

Figure S 32. HSQC spectrum of compound 4 (^{1}H: 600 MHz, ^{13}C: 150 MHz, $CDCl_3$)

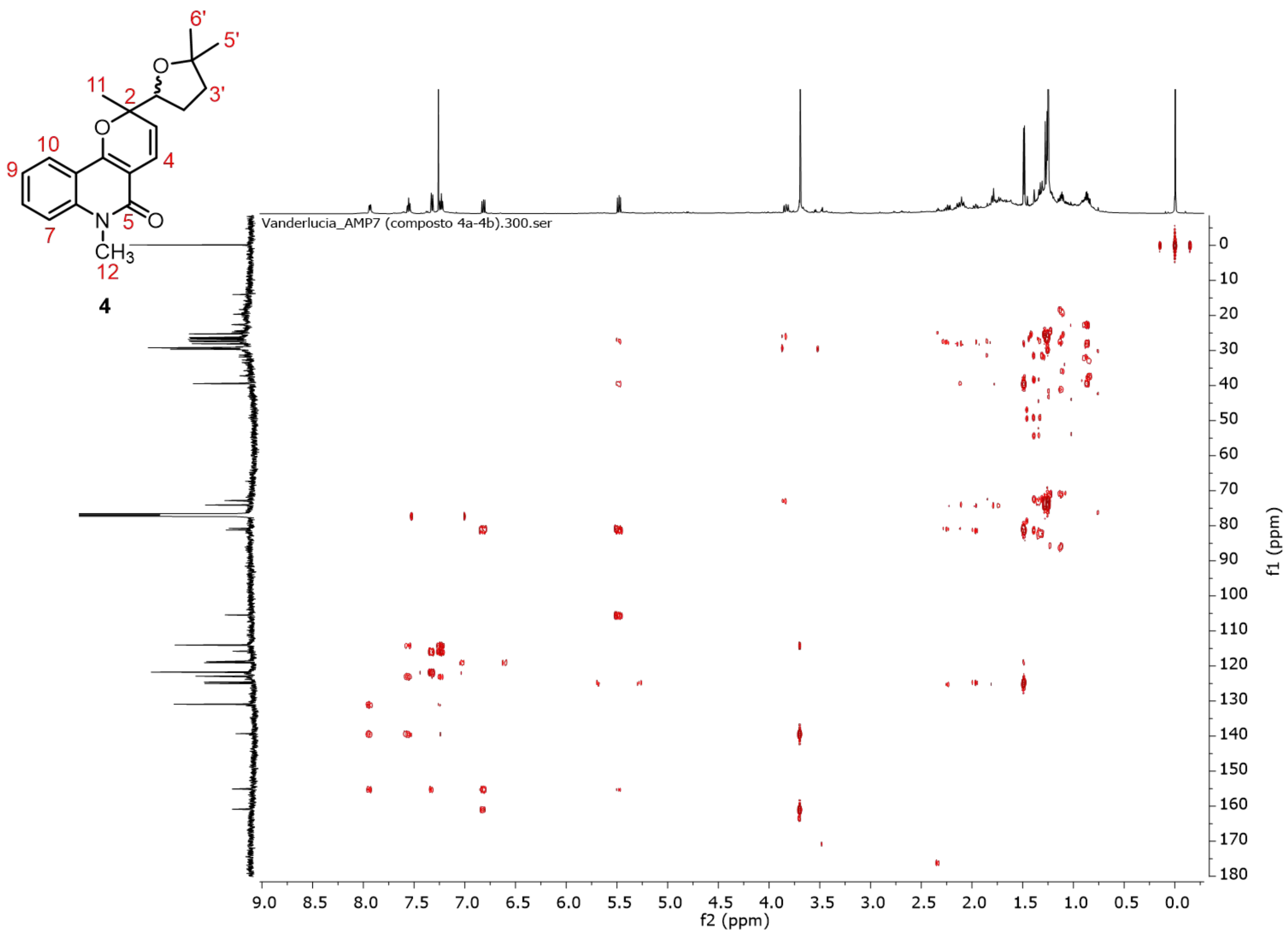

Figure S 33. HMBC spectrum of compound **4** (^{1}H: 600 MHz, ^{13}C: 150 MHz, $CDCl_3$)

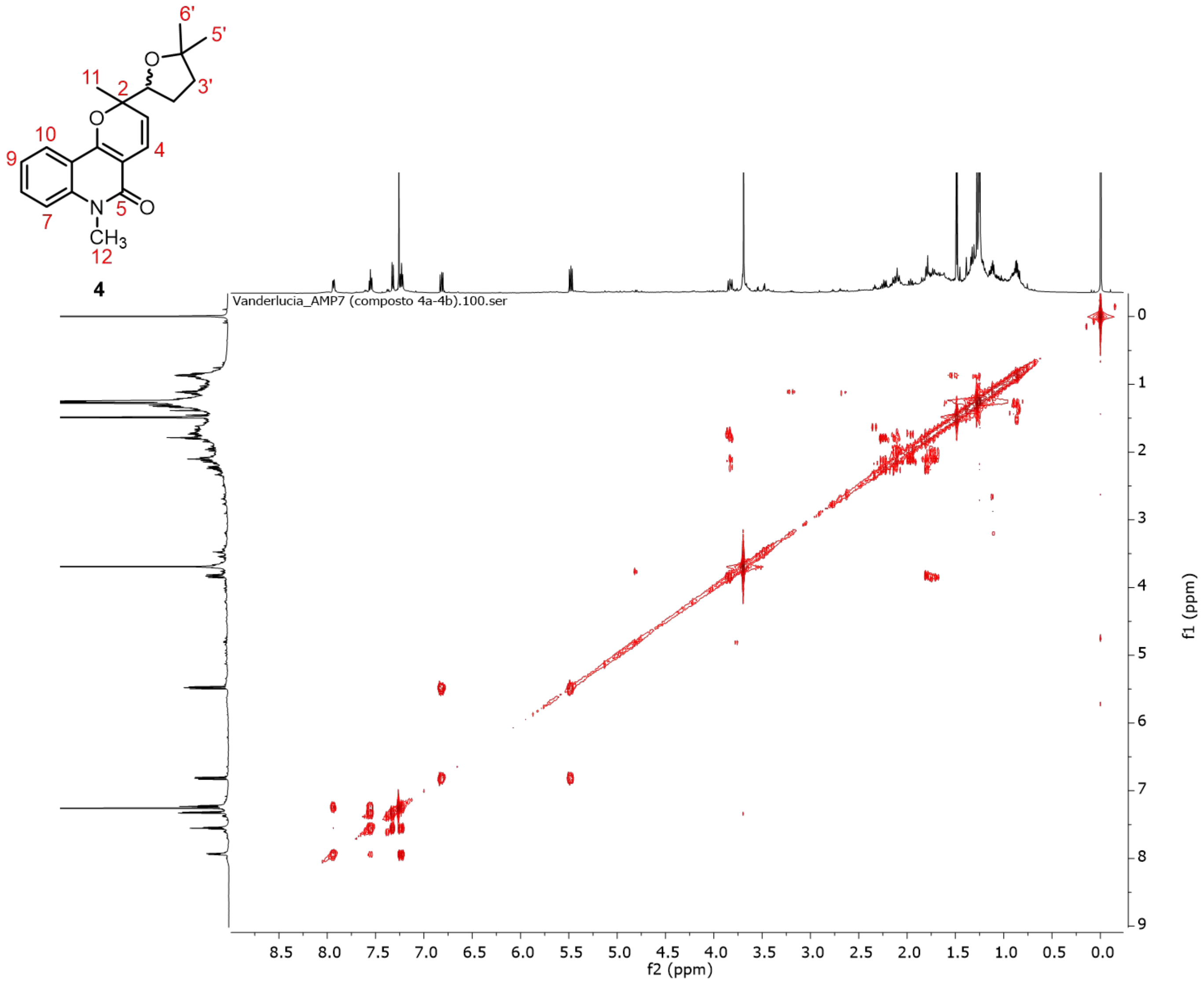

Figure S 34. COSY spectrum of compound **4** (600 MHz, $CDCl_3$)

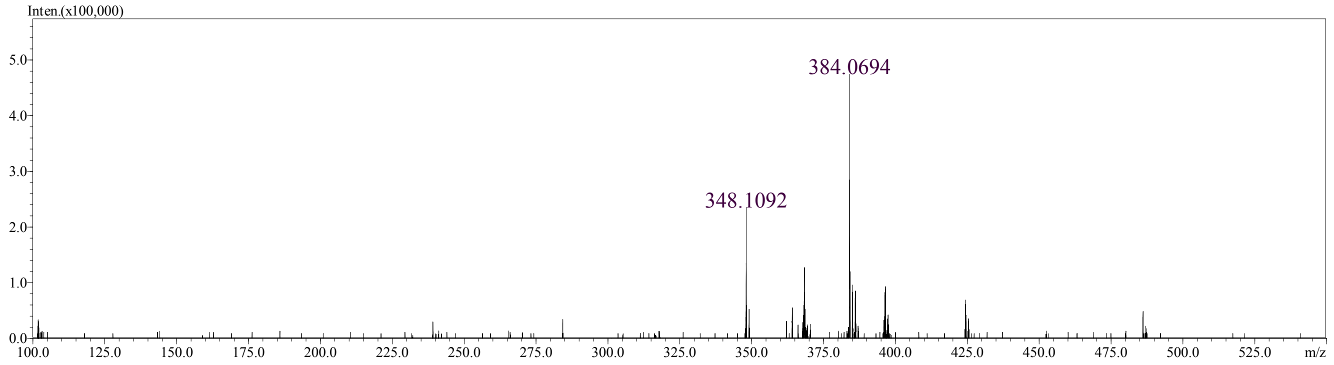

Figure S 35. (+)-HRESIMS spectrum of compound 4

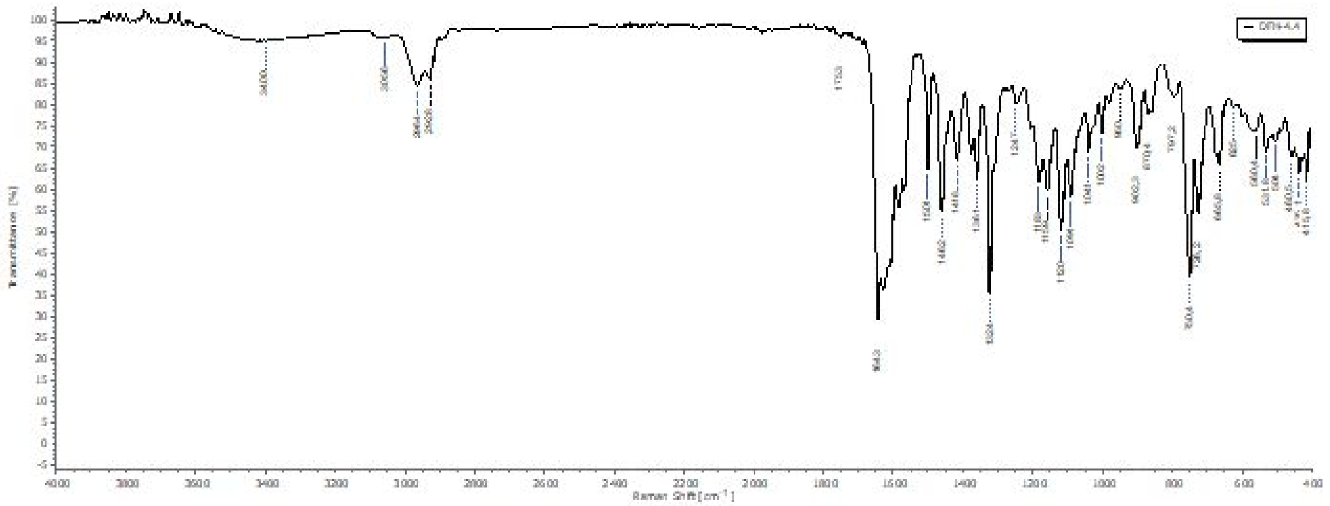

Figure S 36. IR spectrum of compound 4

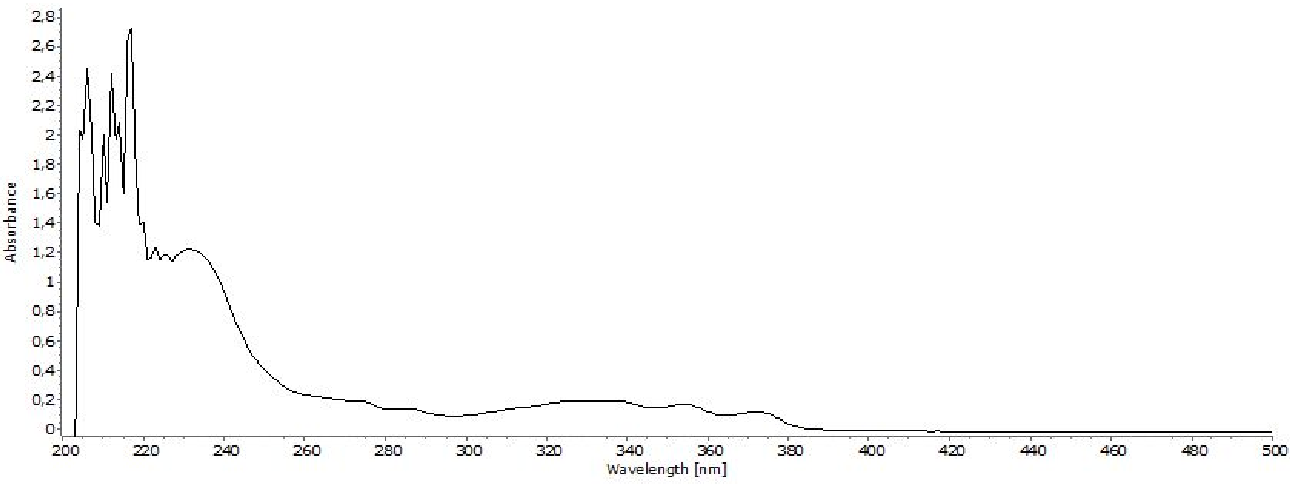

Figure S 37. UV spectrum (15 µg/mL, CH_2Cl_2) of compound **4**

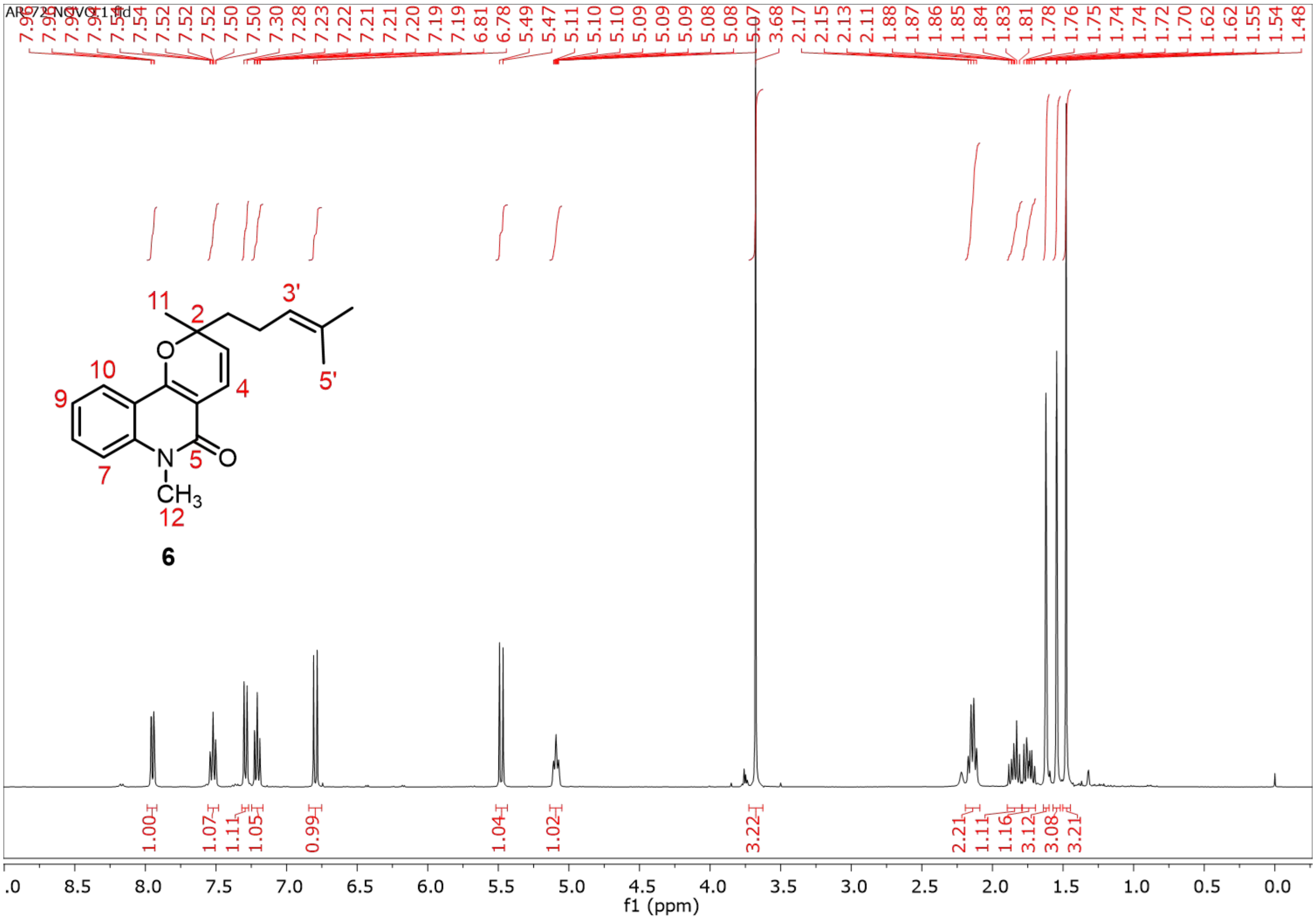

Figure S 38. ^1H NMR spectrum of compound **6** (400 MHz, $CDCl_3$)

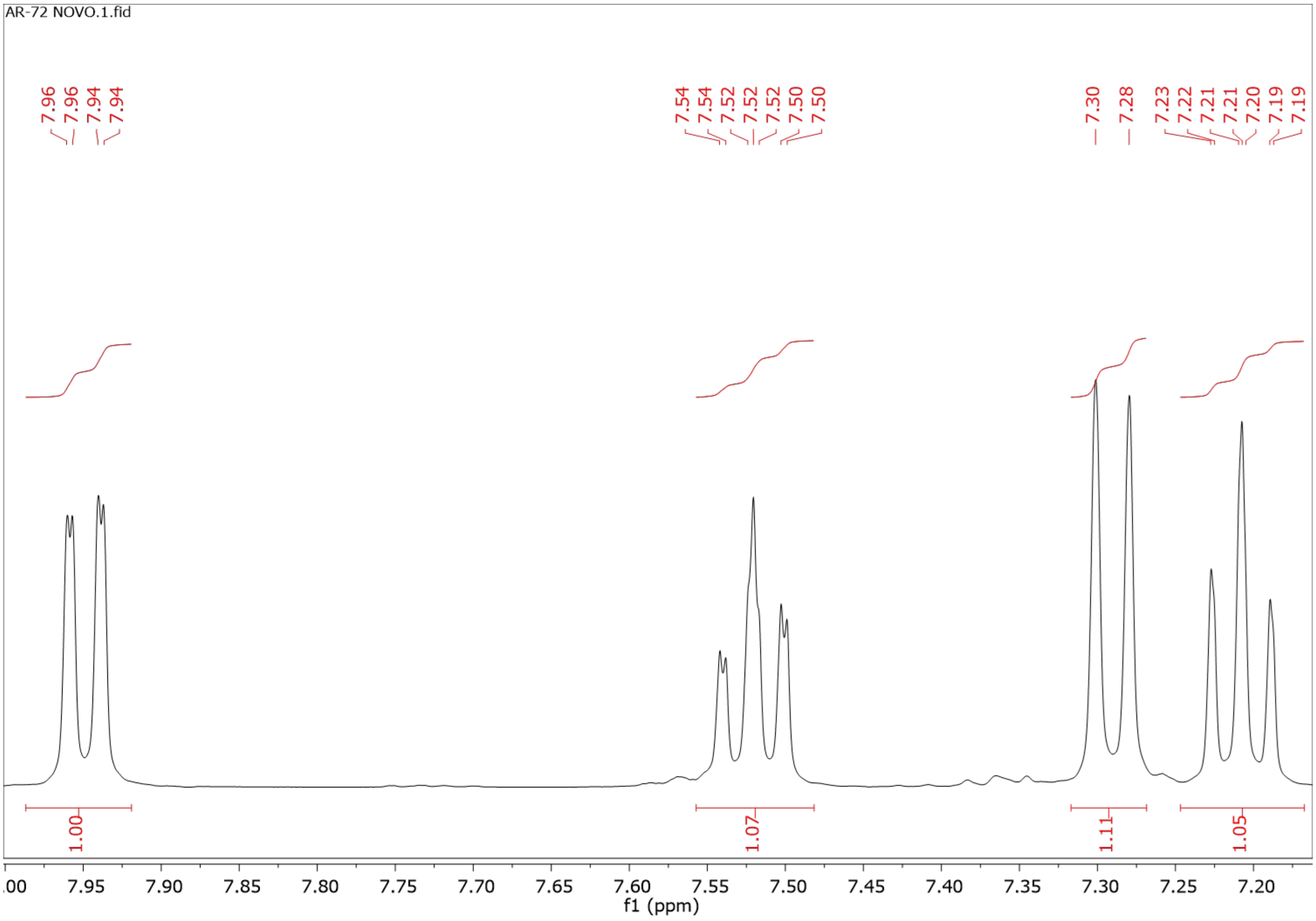

Figure S 39. Expansion 1 of the ^{1}H NMR spectrum of compound **6** (400 MHz, $CDCl_3$)

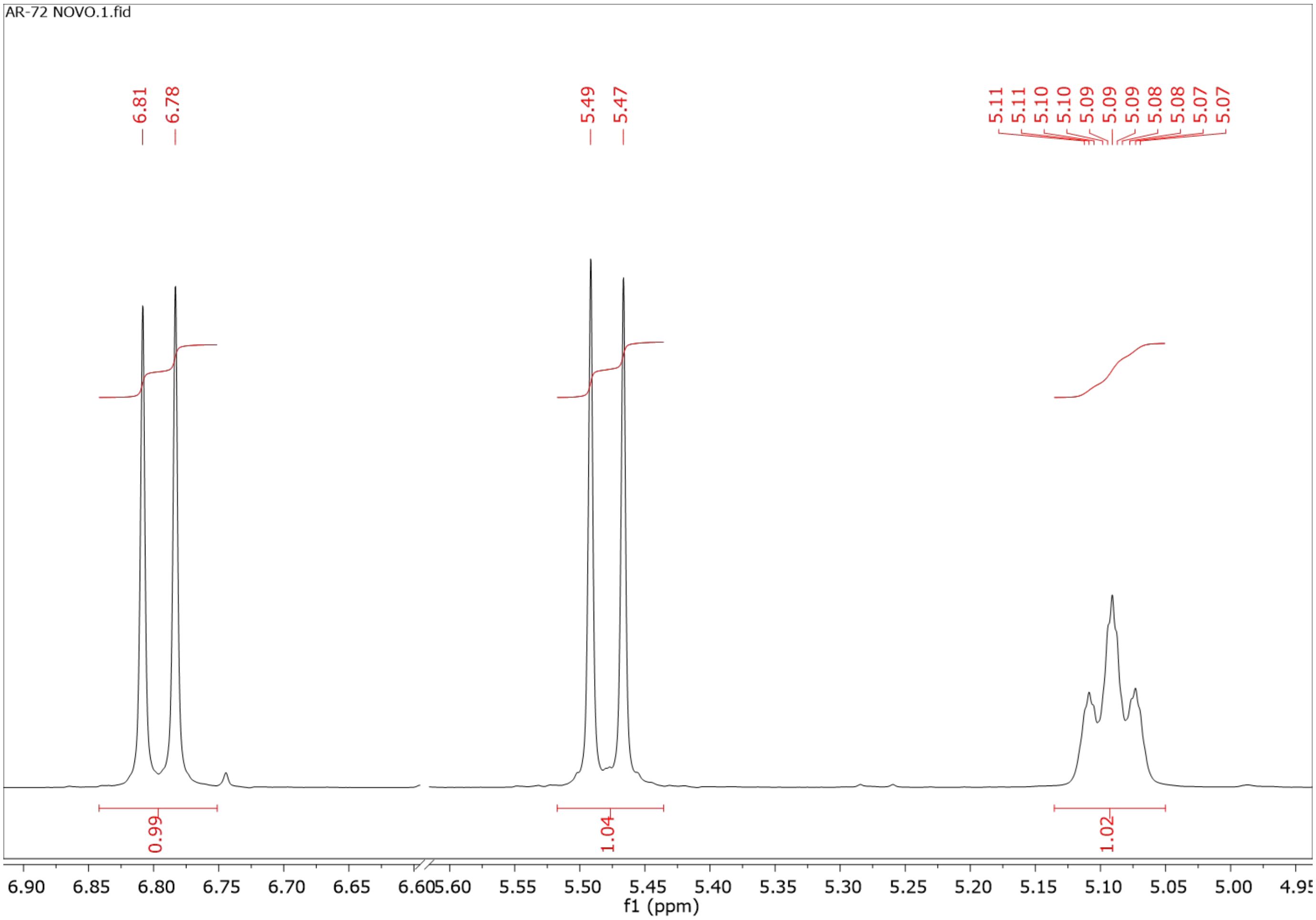

Figure S 40. Expansion 2 of the ^{1}H NMR spectrum of compound **6** (400 MHz, $CDCl_3$)

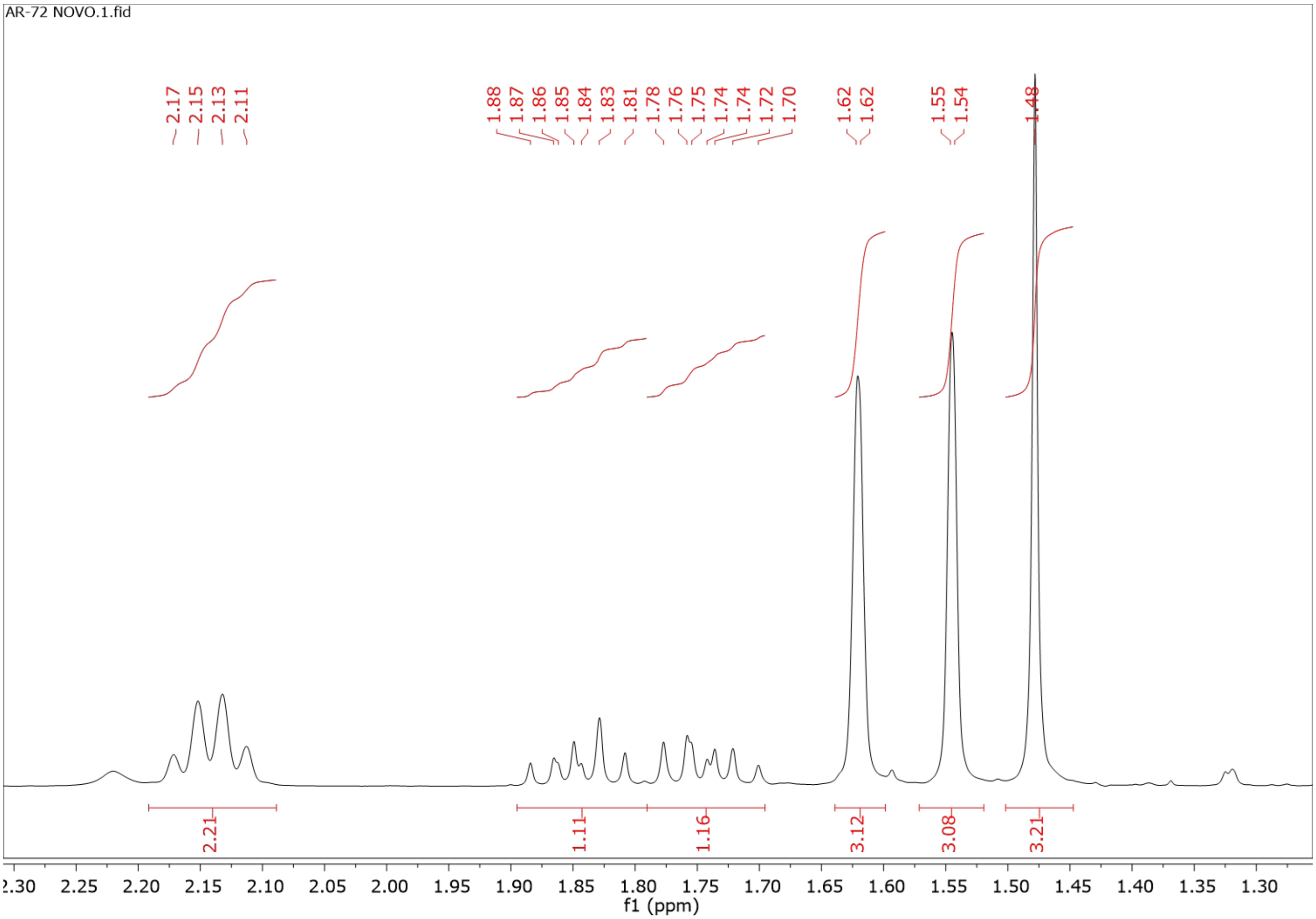

Figure S 41. Expansion 3 of the ^{1}H NMR spectrum of compound **6** (400 MHz, $CDCl_3$)

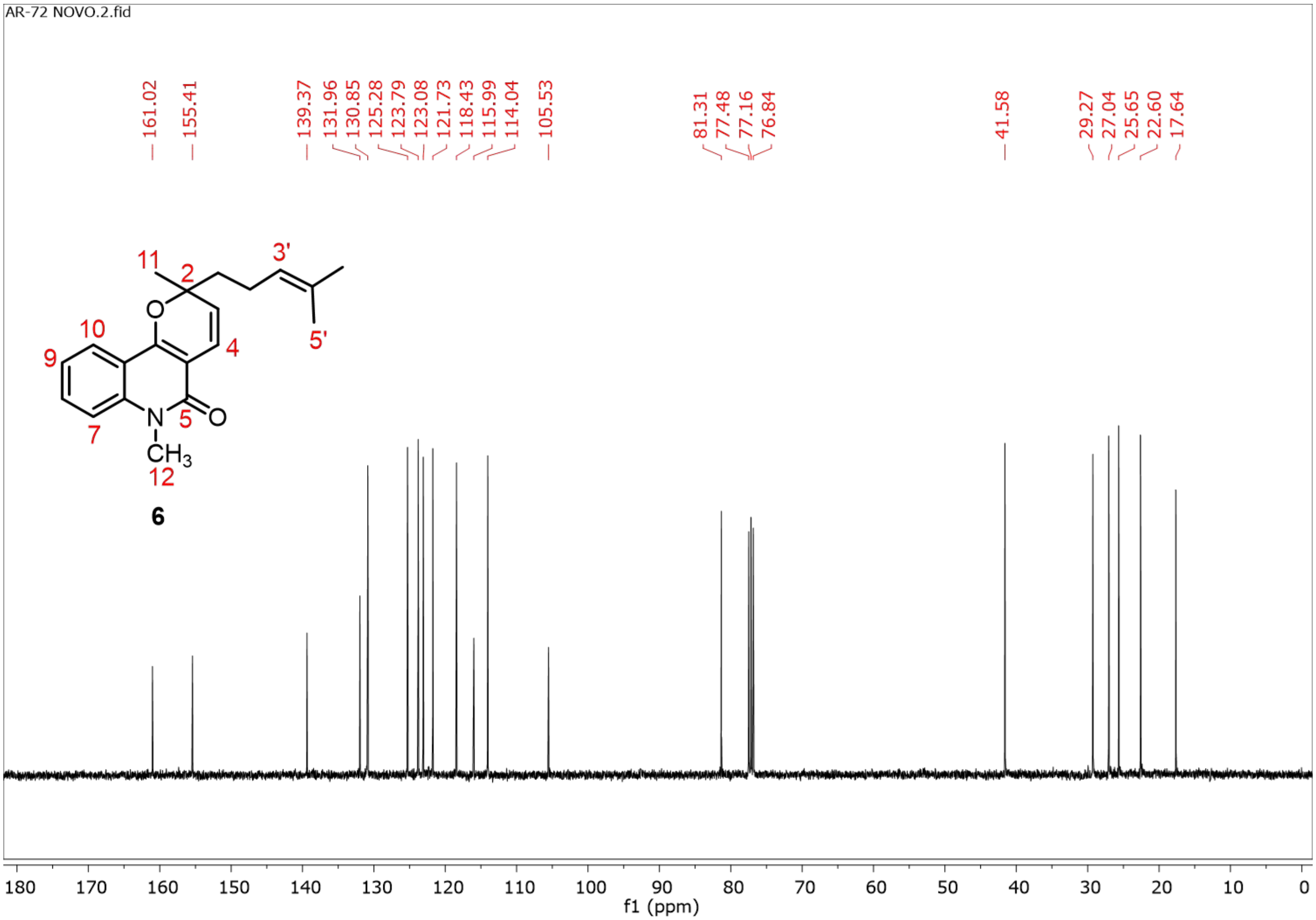

Figure S 42. ^{13}C NMR spectrum of compound **6** (100 MHz, $CDCl_3$)

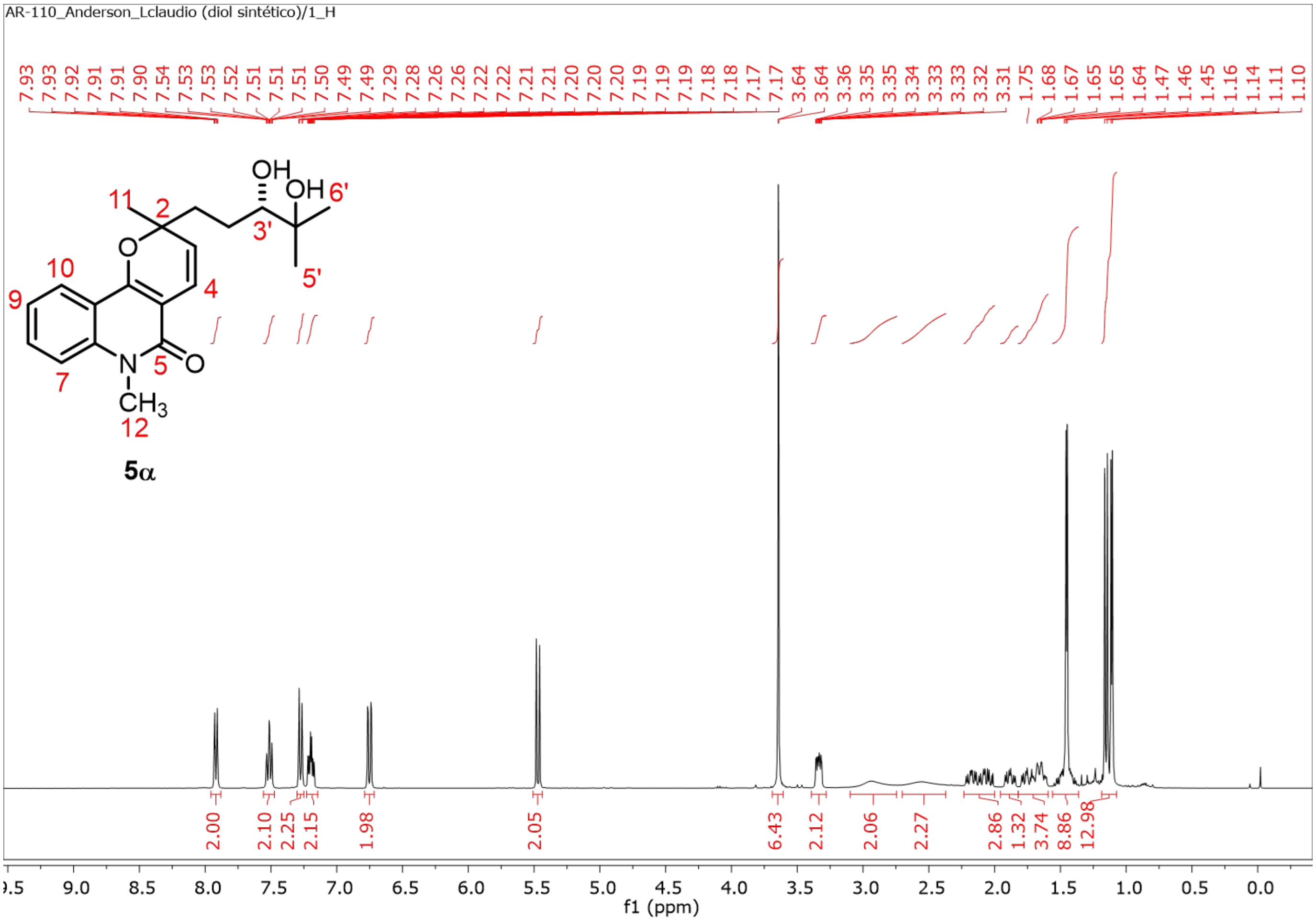

Figure S 43. ^{1}H NMR spectrum of compound **5α** (400 MHz, $CDCl_3$)

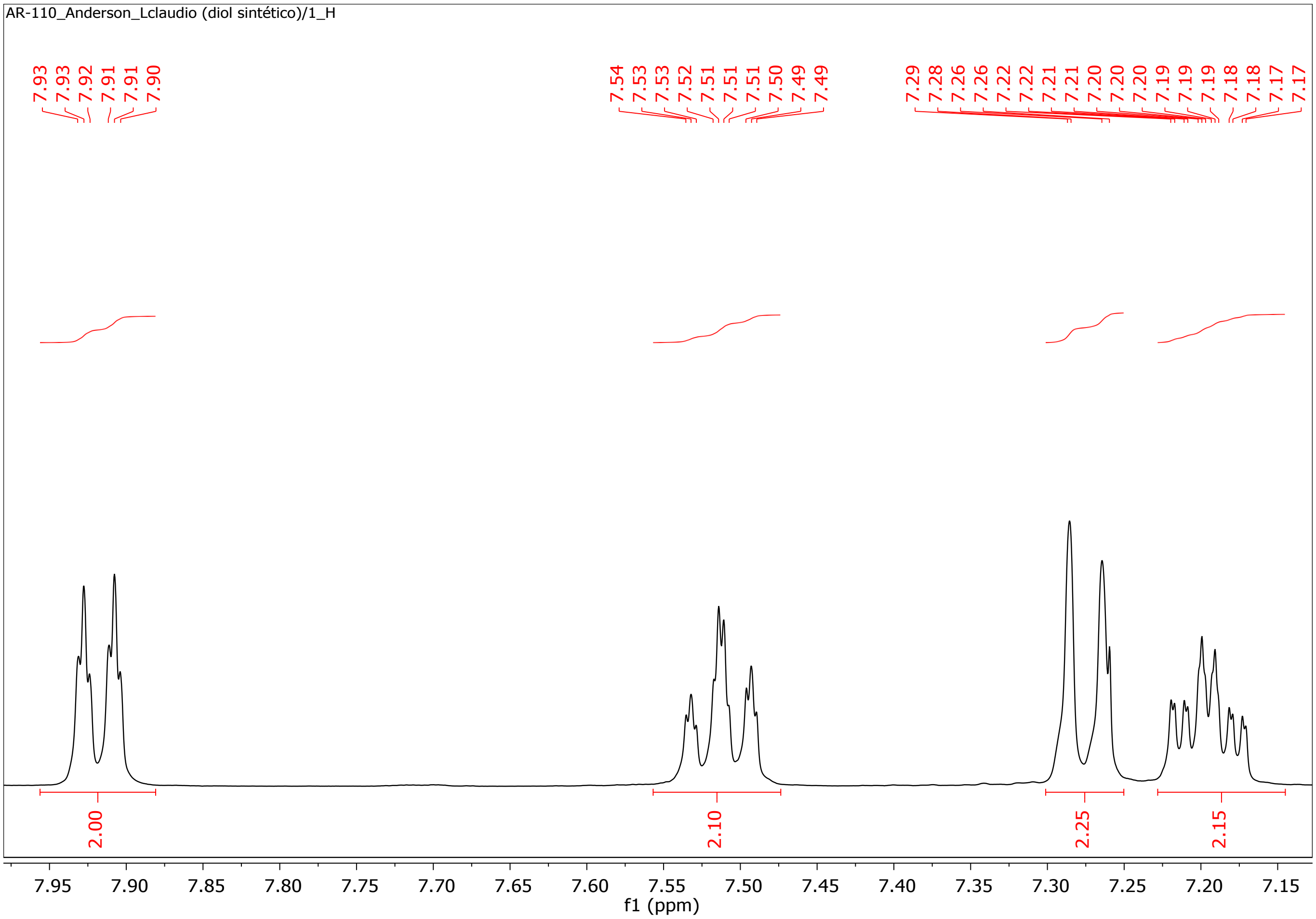

Figure S 44. Expansion 1 of ¹H NMR spectrum of compound **5α** (400 MHz, $CDCl_3$)

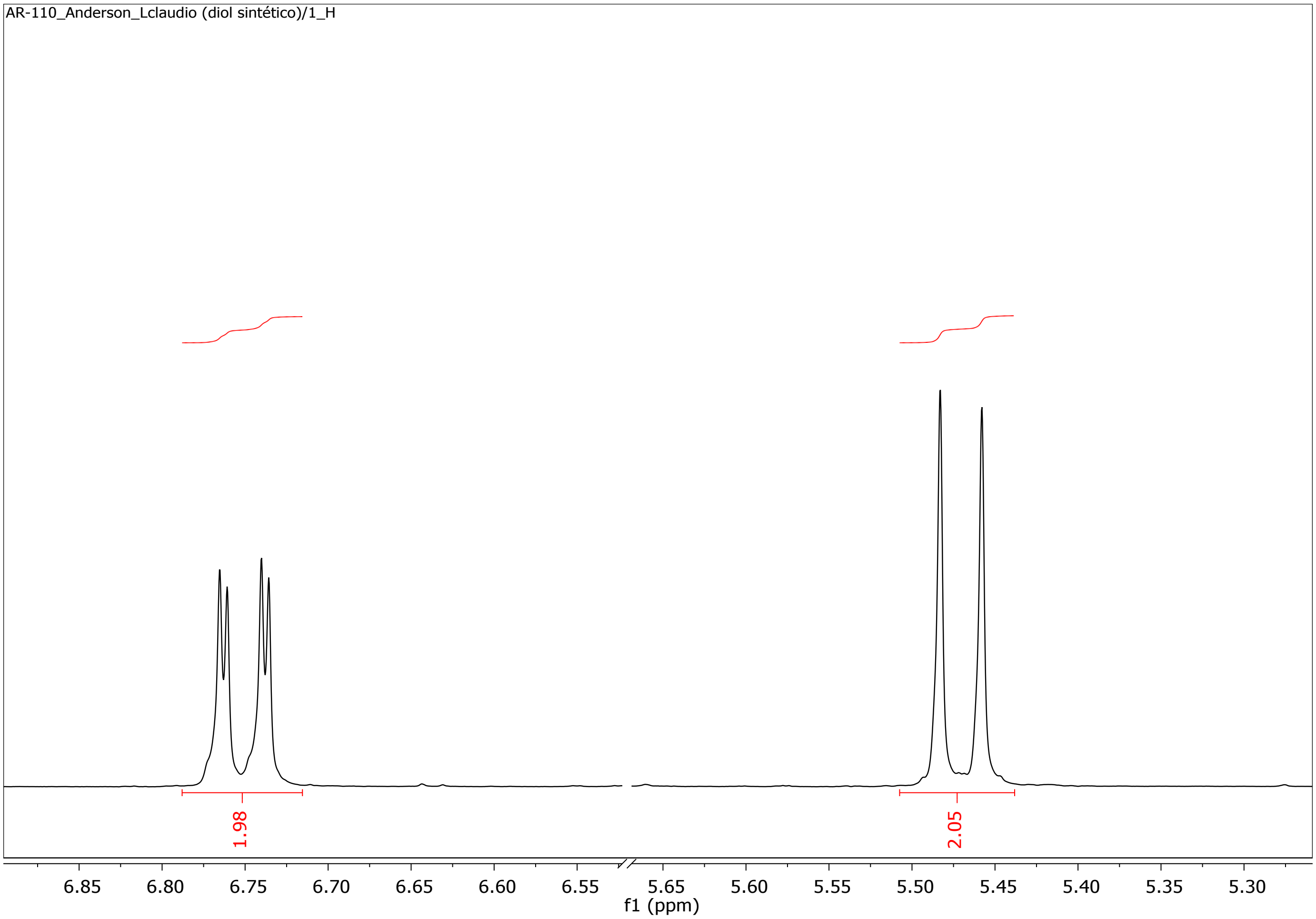

Figure S 45. Expansion 2 of ^{1}H NMR spectrum of compound **5*α*** (400 MHz, $CDCl_3$)

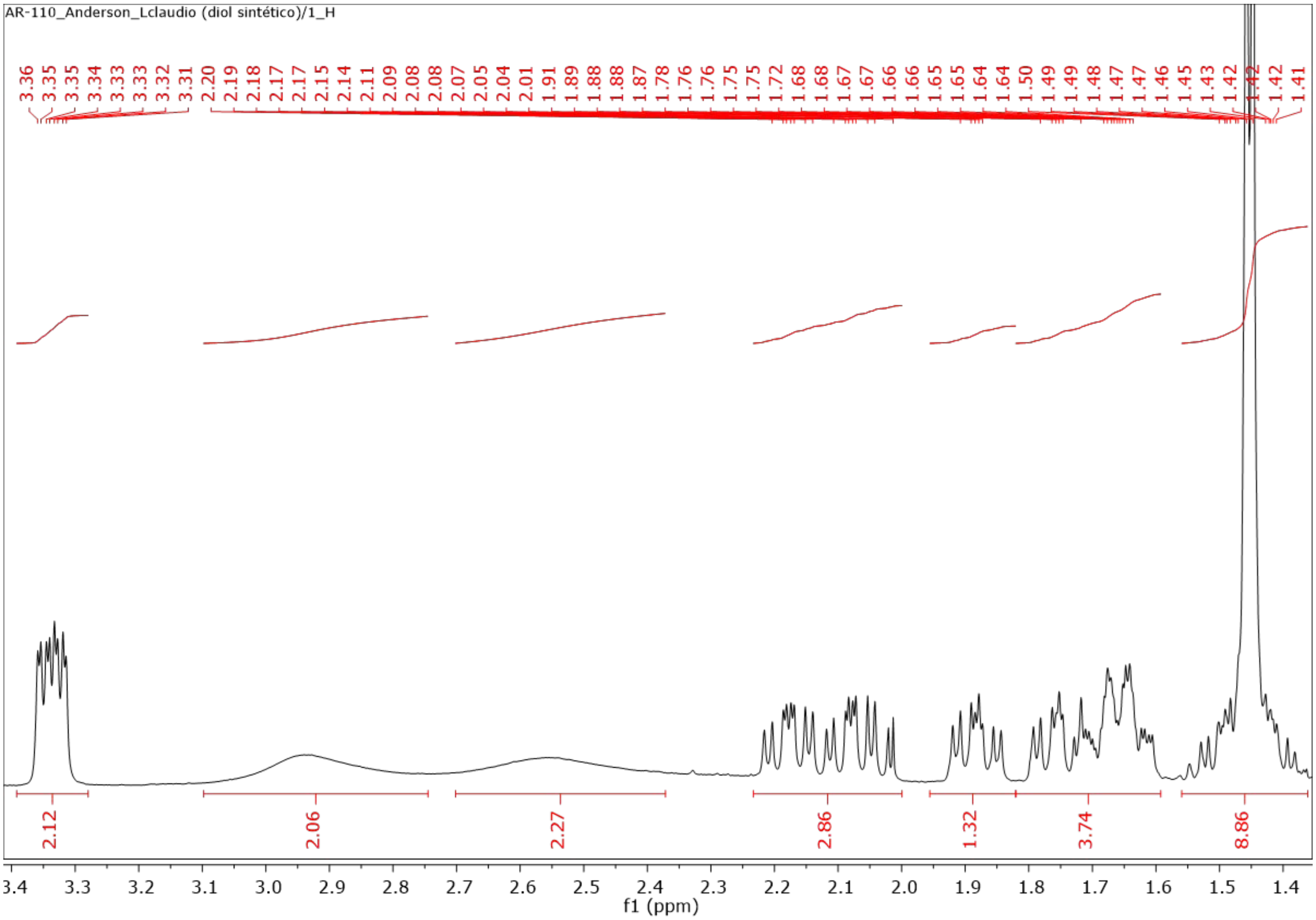

Figure S 46. Expansion 3 of ^{1}H NMR spectrum of compound **5α** (400 MHz, $CDCl_3$).

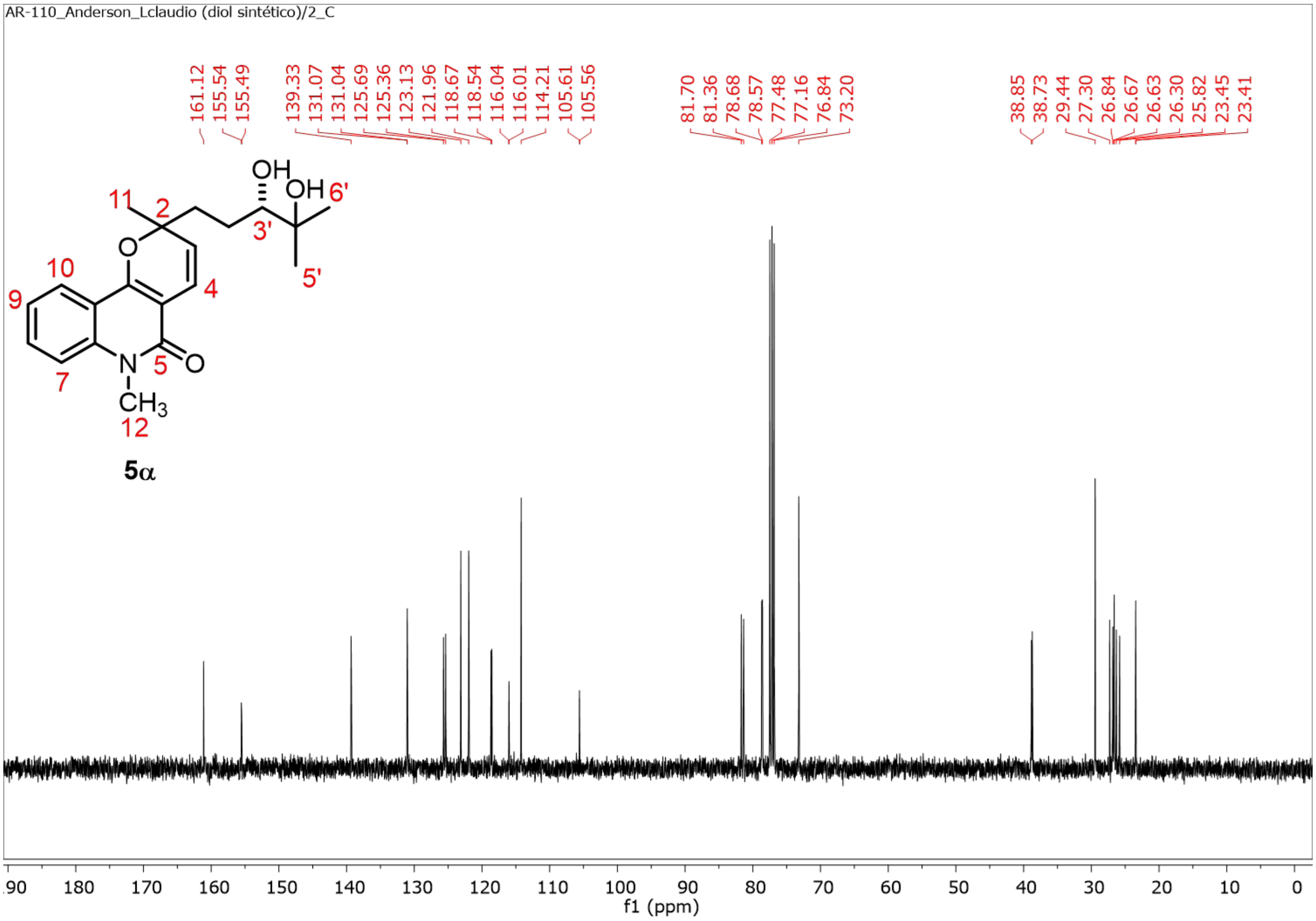

Figure S 47. ^{13}C NMR spectrum of compound **5α** (100 MHz, $CDCl_3$)

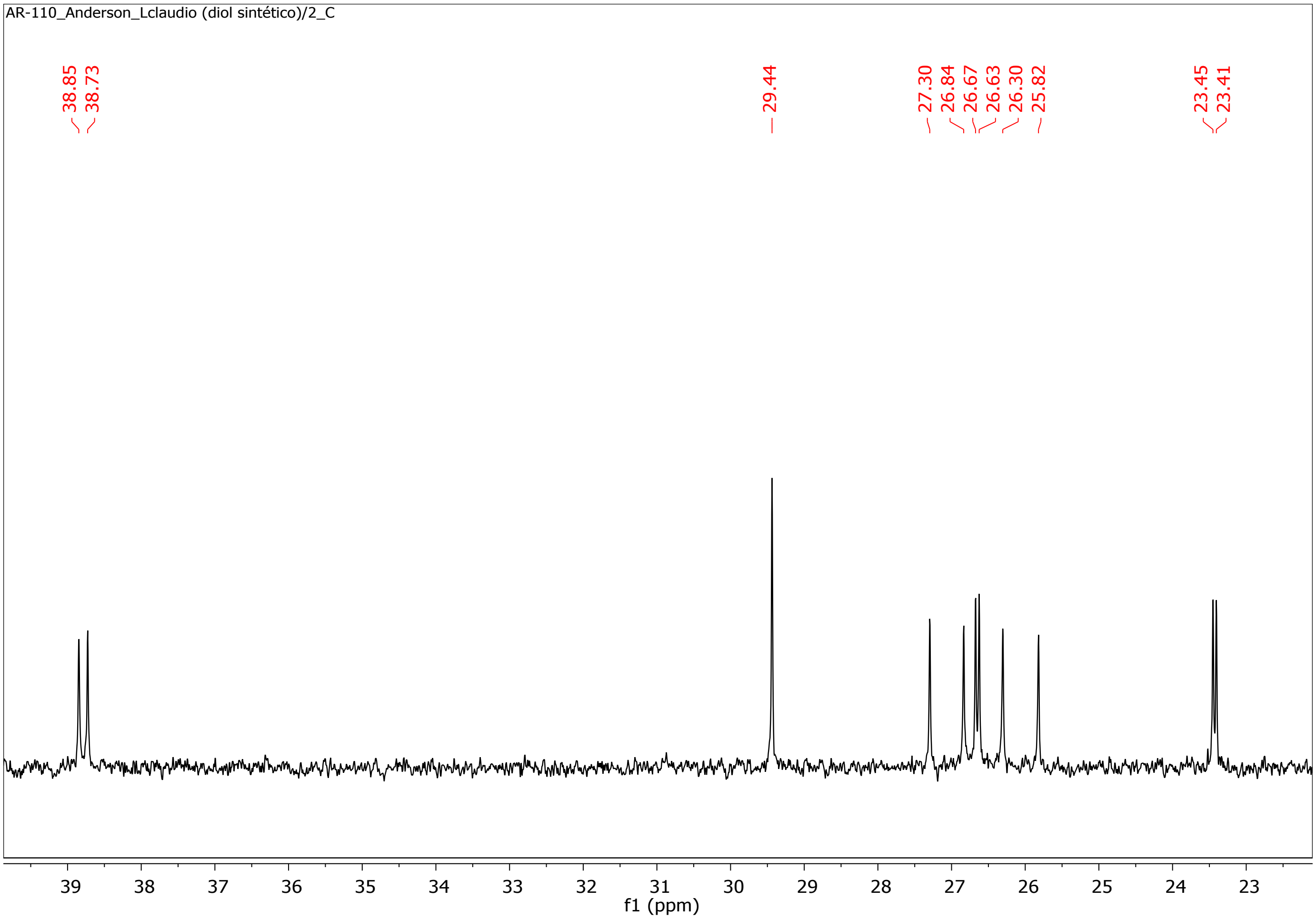

Figure S 48. Expansion of the ^{13}C NMR spectrum of compound **5α** (100 MHz, $CDCl_3$)

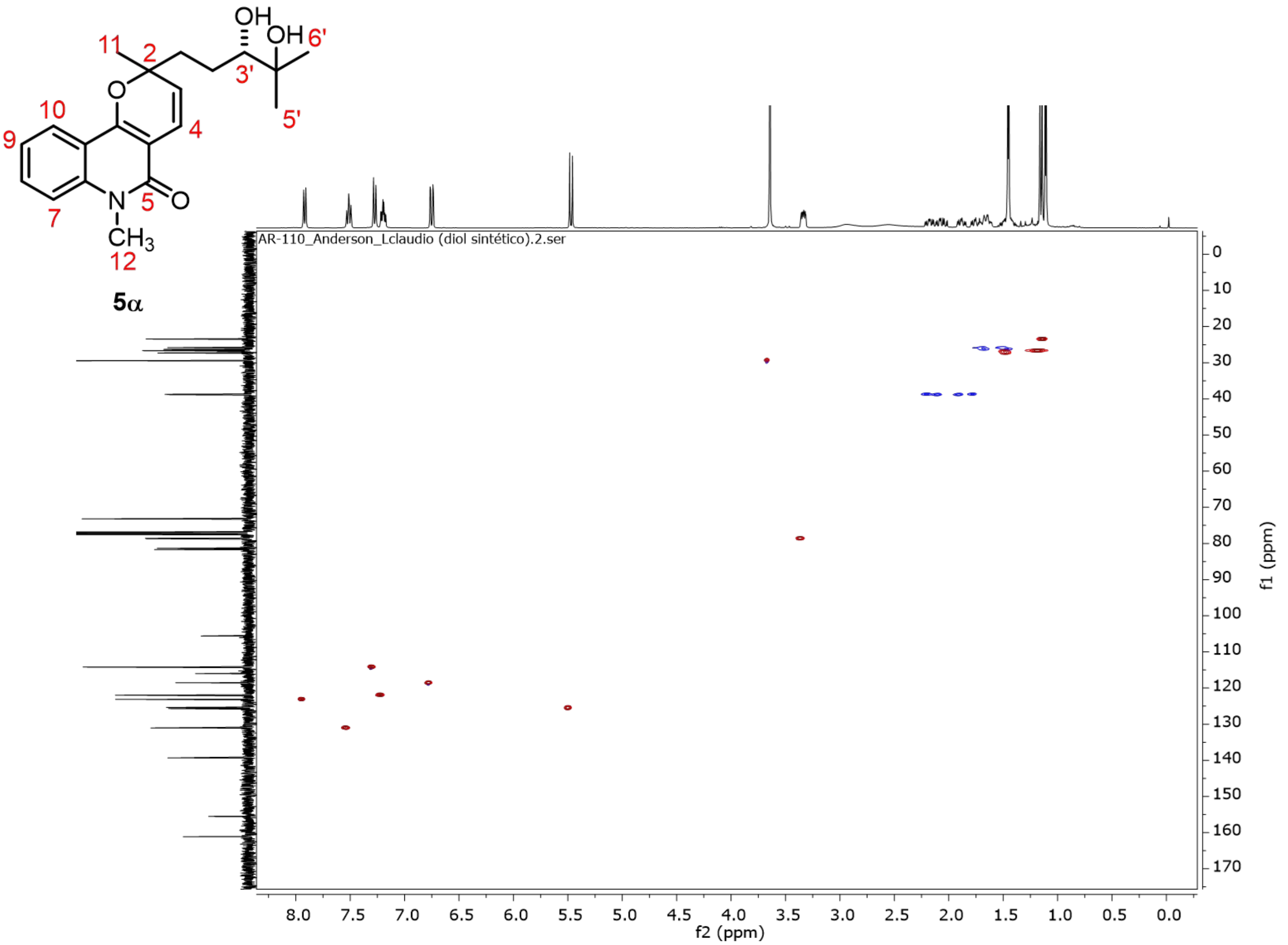

Figure S 49. HSQC spectrum of compound **5α** (^{1}H: 400 MHz, ^{13}C: 100 MHz, $CDCl_3$)

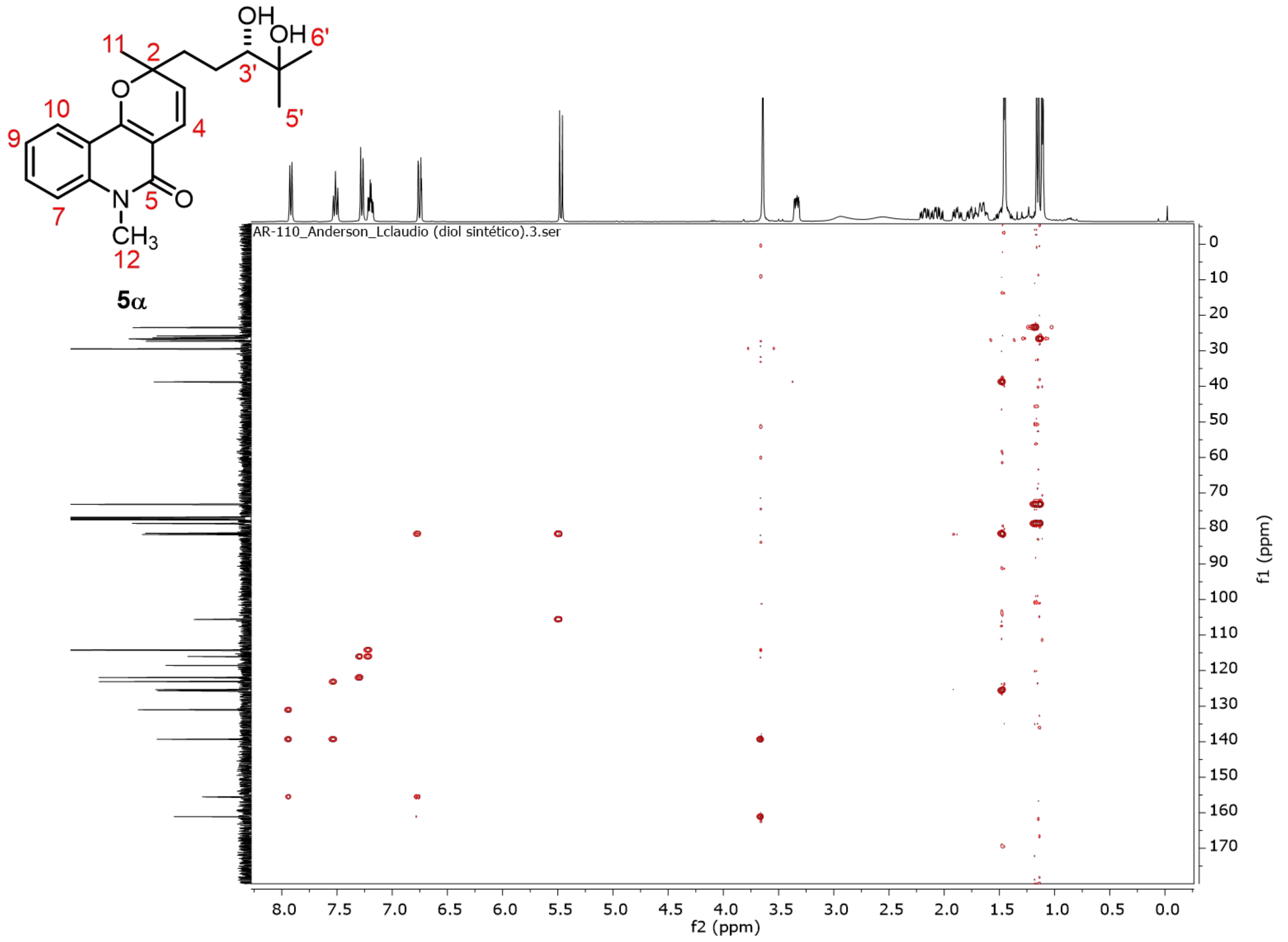

Figure S 50. HMBC spectrum of compound **5*α*** (^{1}H: 400 MHz, ^{13}C: 100 MHz, $CDCl_3$)

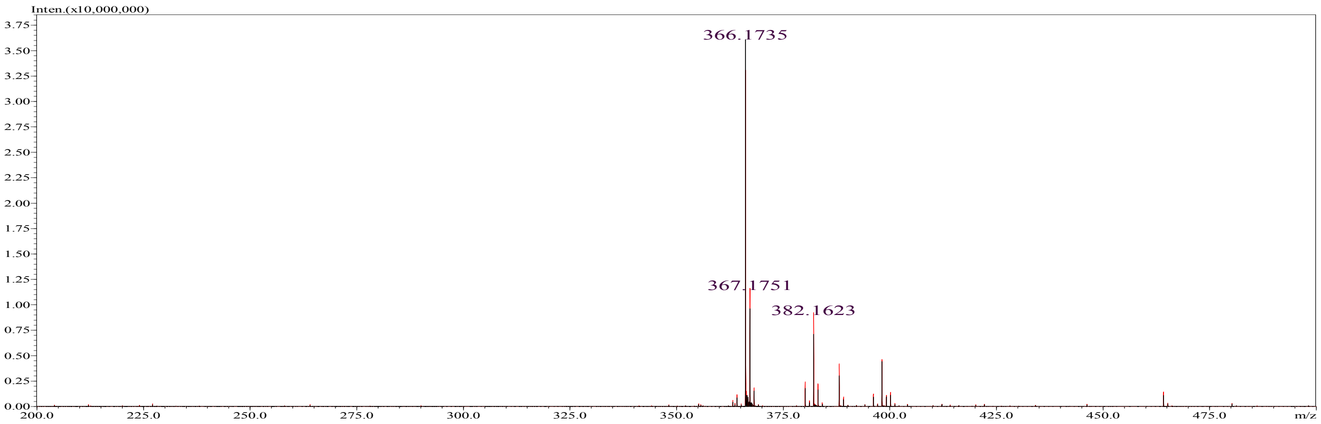

Figure S 51. (+)-HRESIMS spectrum of compound **5α**

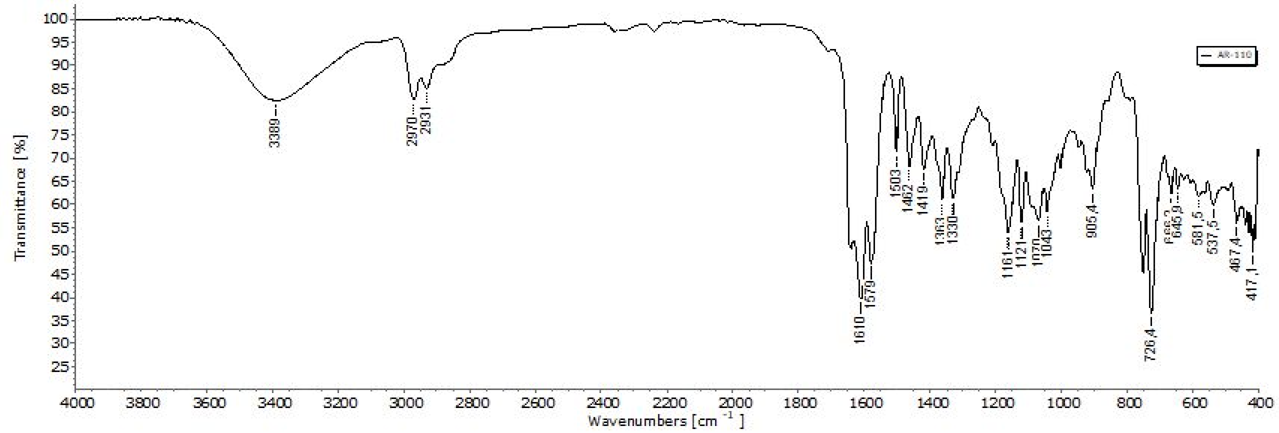

Figure S 52. IR spectrum of compound **5α**

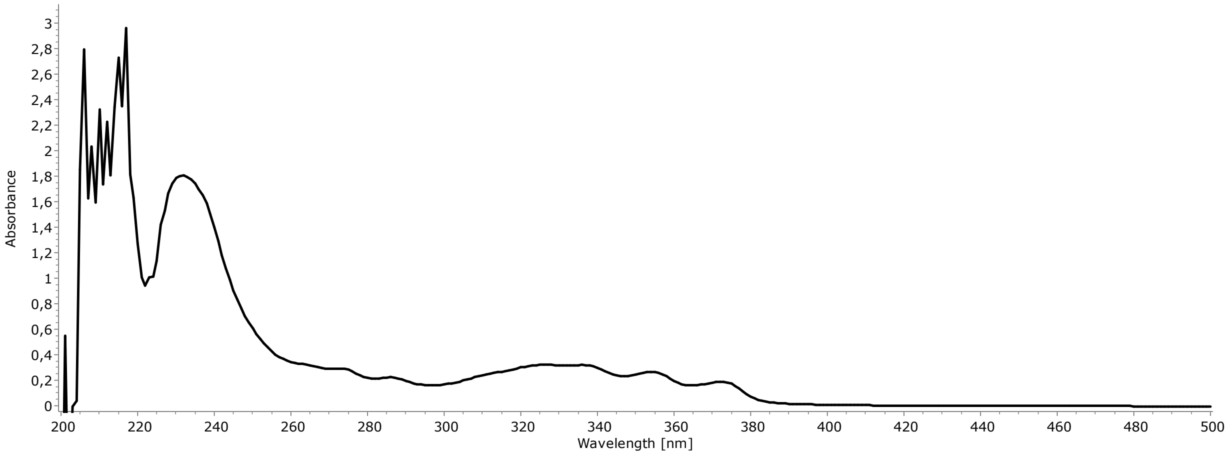

Figure S 53. UV spectrum (17 µg/mL, CH_2Cl_2) of compound ***5α***

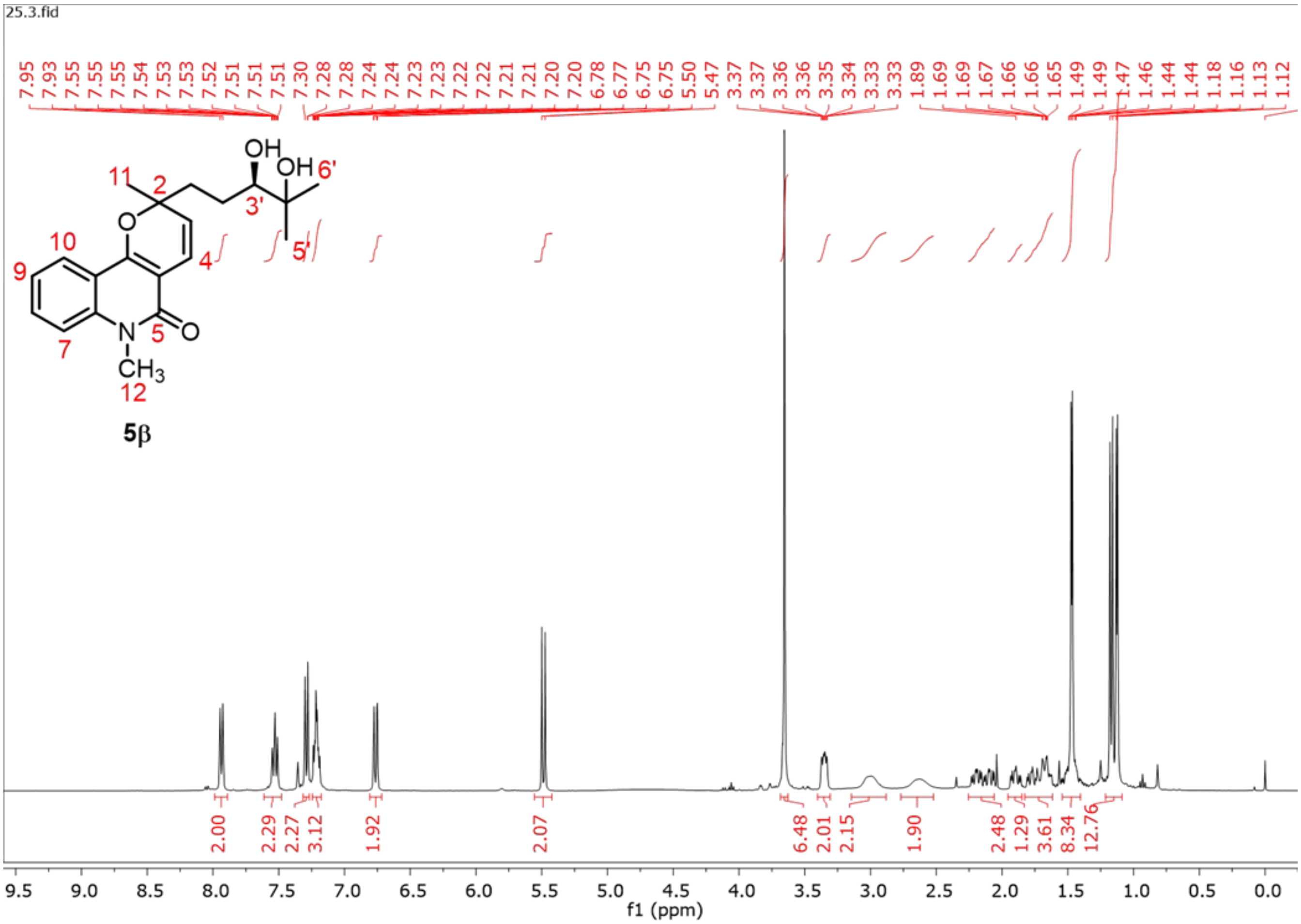

Figure S 54. ^{1}H NMR spectrum of compound ***5β*** (400 MHz, $CDCl_3$)

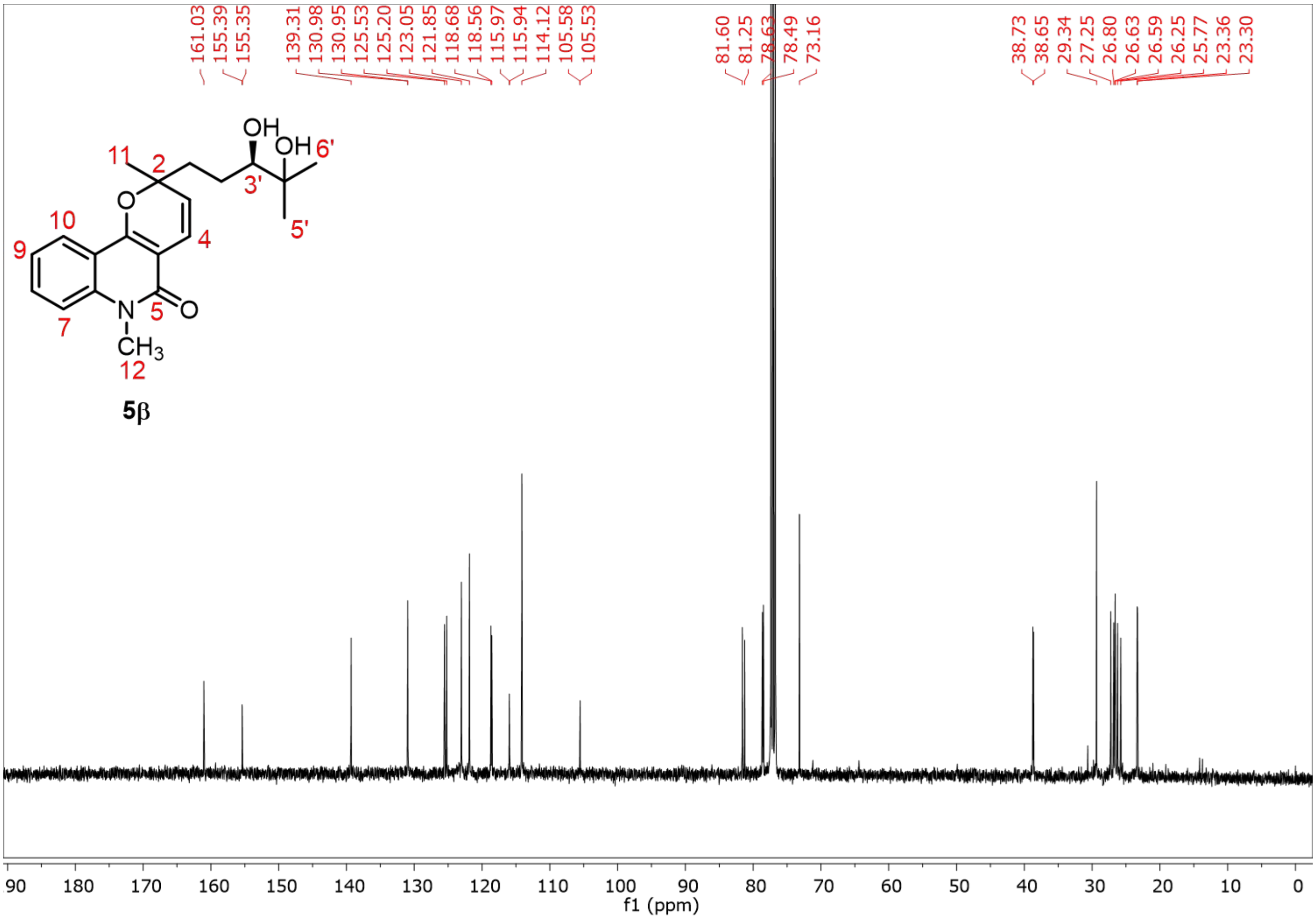

Figure S 55. ^{13}C NMR spectrum of compound **5*β*** (100 MHz, $CDCl_3$)

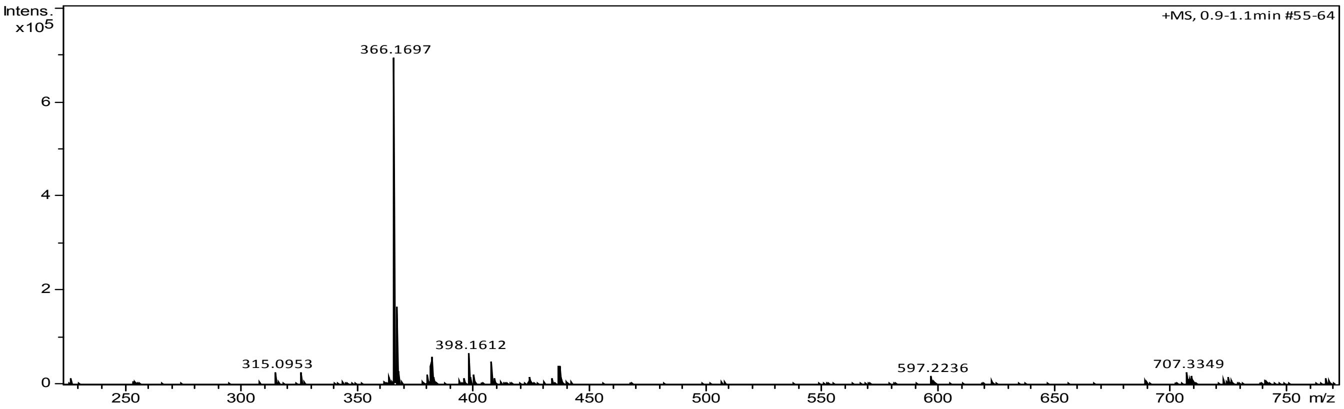

Figure S 56. (+)-HRESIMS spectrum of compound **5*β***

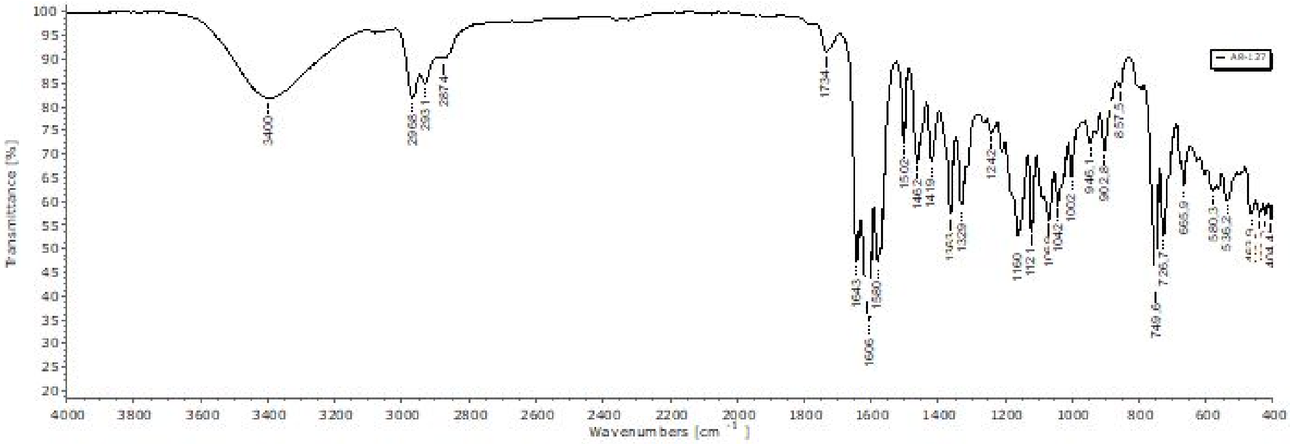

Figure S 57. IR spectrum of compound **5*β***

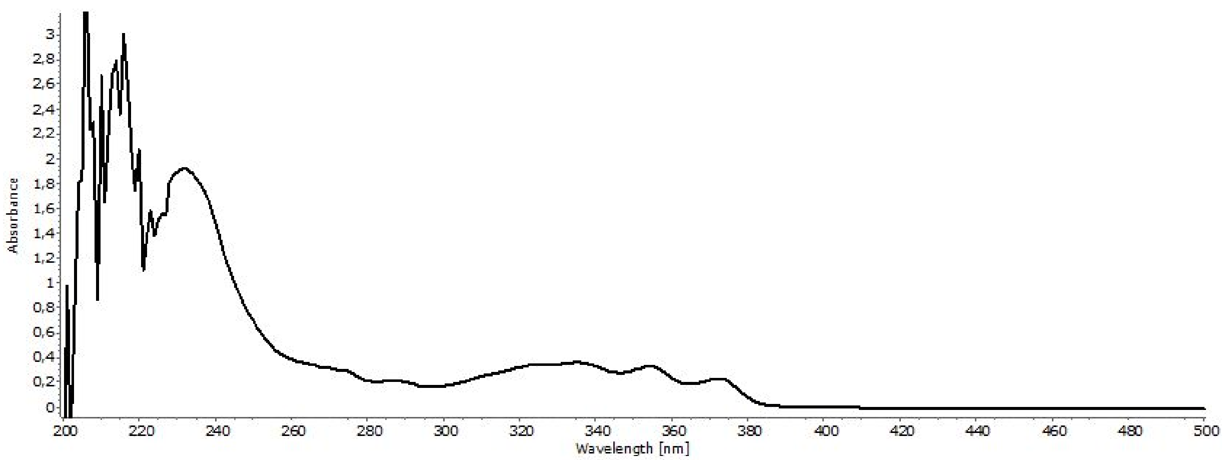

Figure S 58. UV spectrum (15 μg/mL, CH_2Cl_2) of compound **5*β***

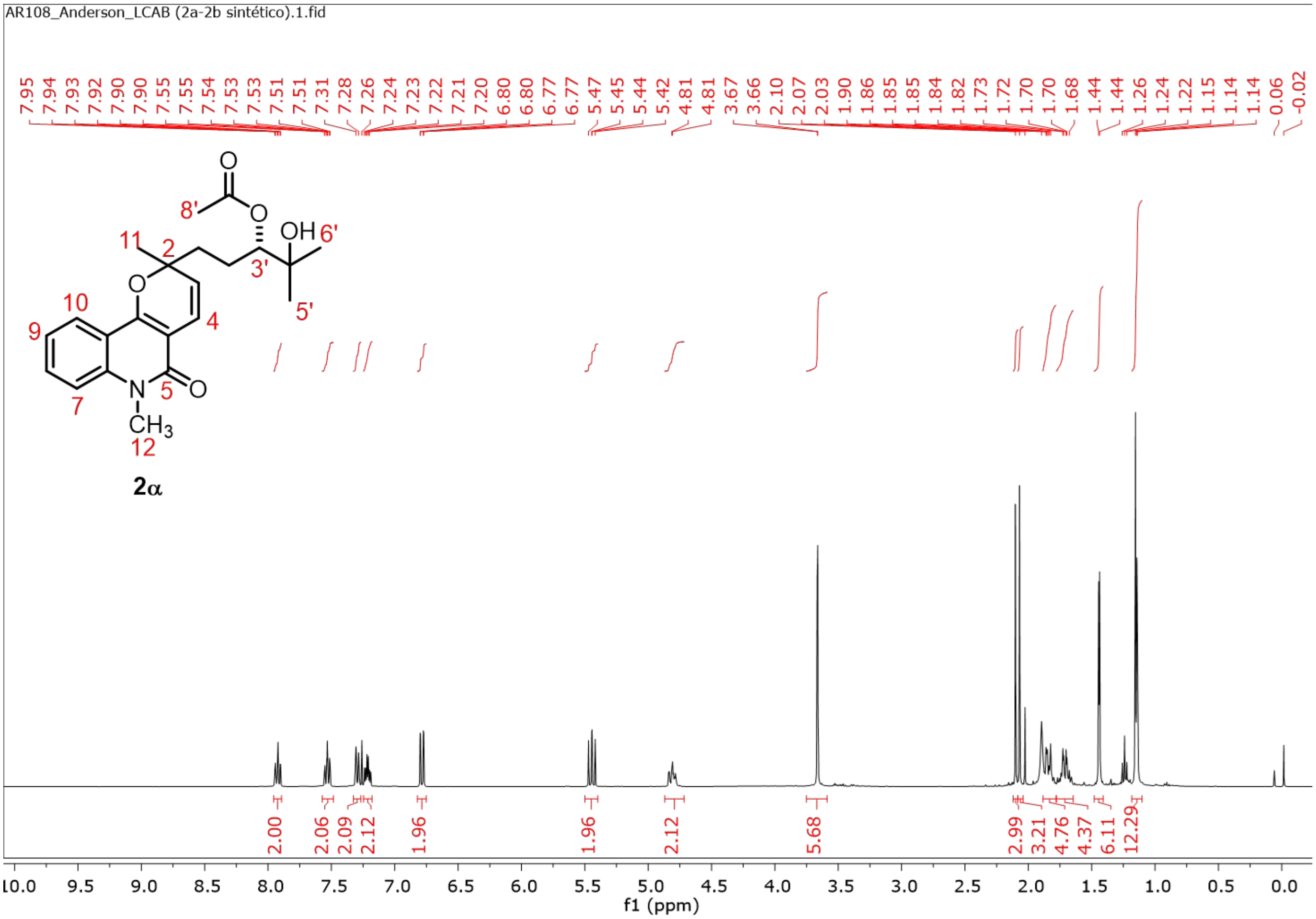

Figure S 59. ^{1}H NMR spectrum of compound **2α** (400 MHz, $CDCl_3$)

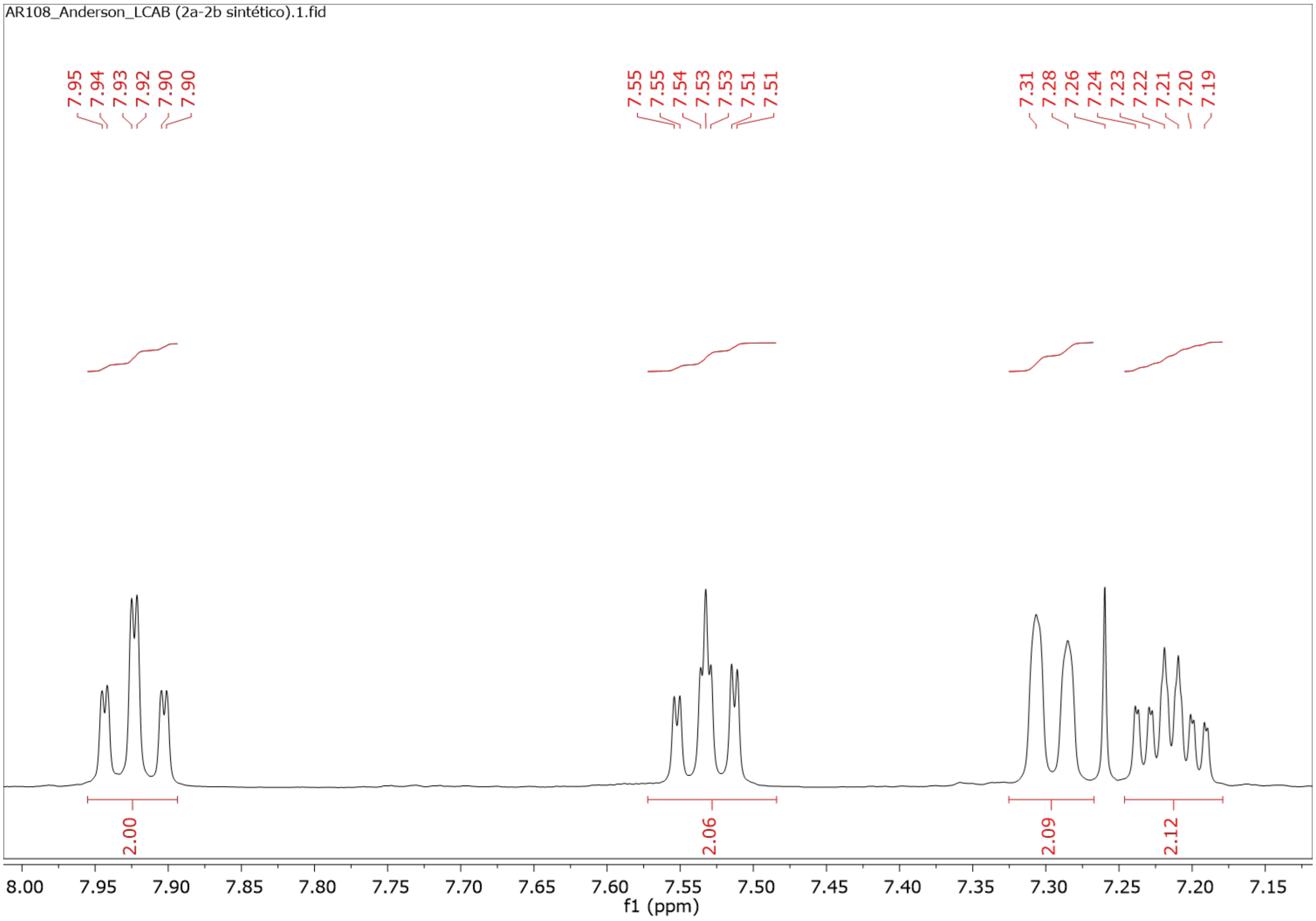

Figure S 60. Expansion 1 of the ^{1}H NMR spectrum of compound **2α** (400 MHz, $CDCl_3$)

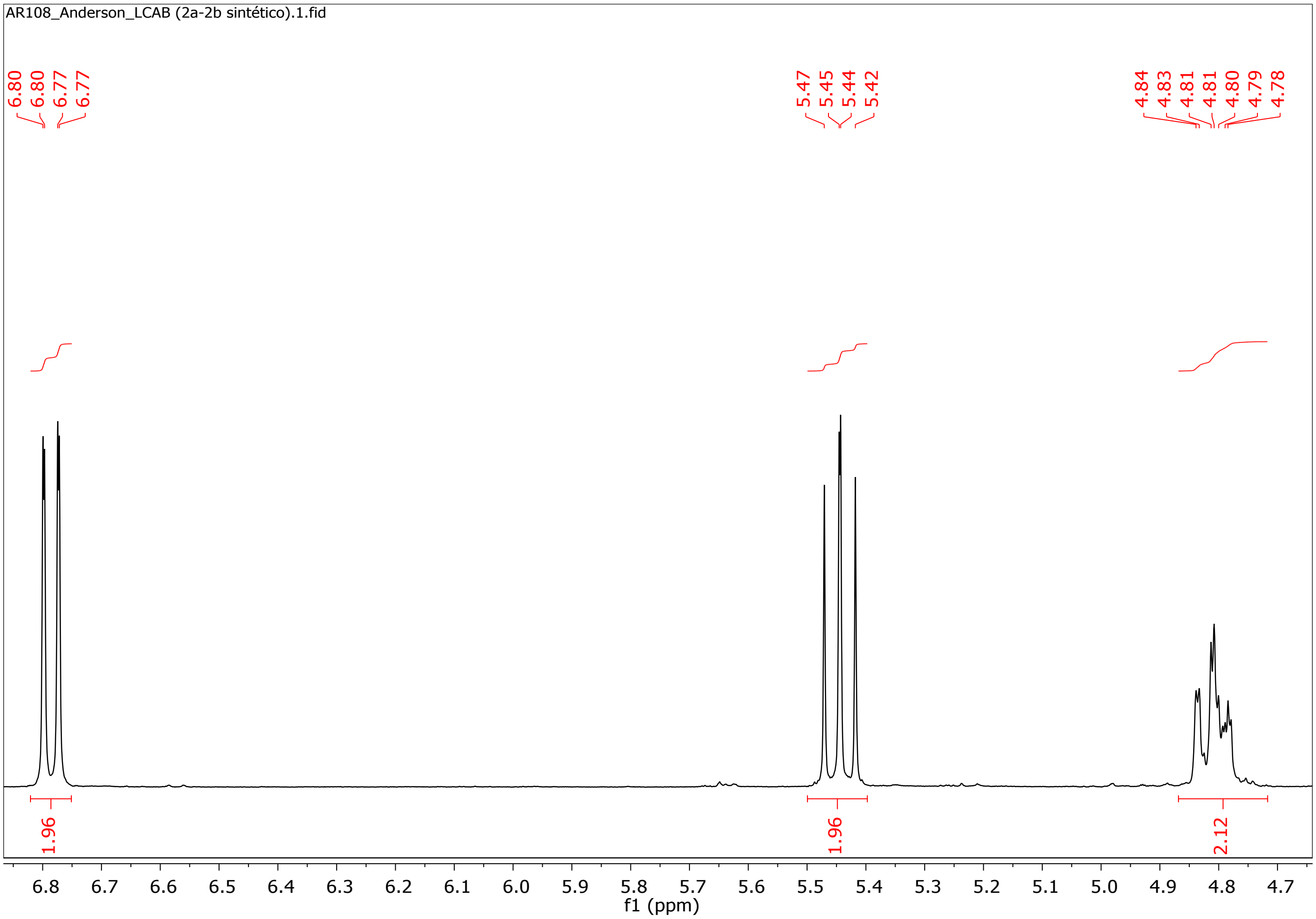

Figure S 61. Expansion 2 of the ^{1}H NMR spectrum of compound **2*α*** (400 MHz, $CDCl_3$)

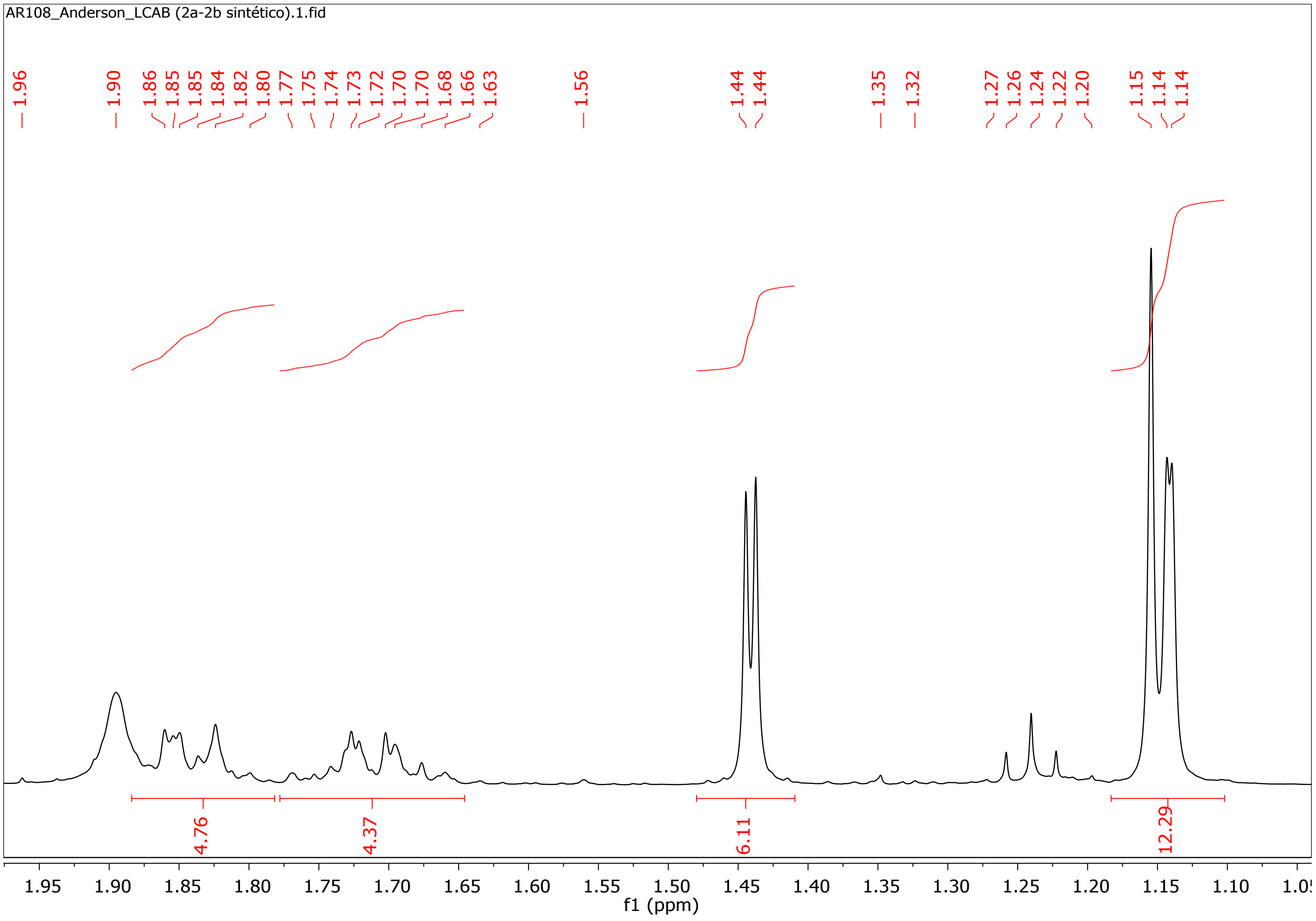

Figure S 62. Expansion 3 of the ^{1}H NMR spectrum of compound **2α** (400 MHz, $CDCl_3$)

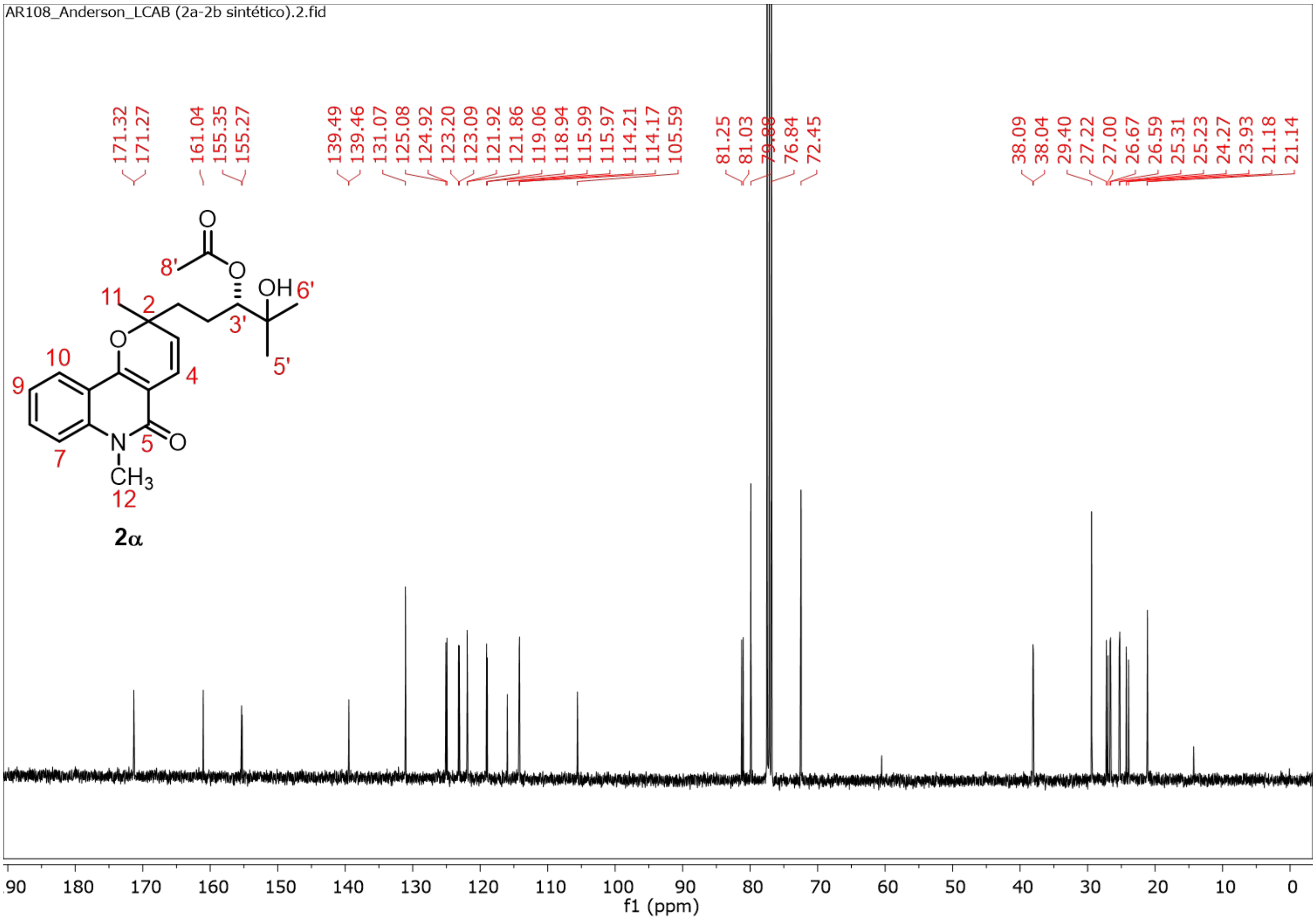

Figure S 63. ^{13}C NMR spectrum of compound **2α** (100 MHz, $CDCl_3$)

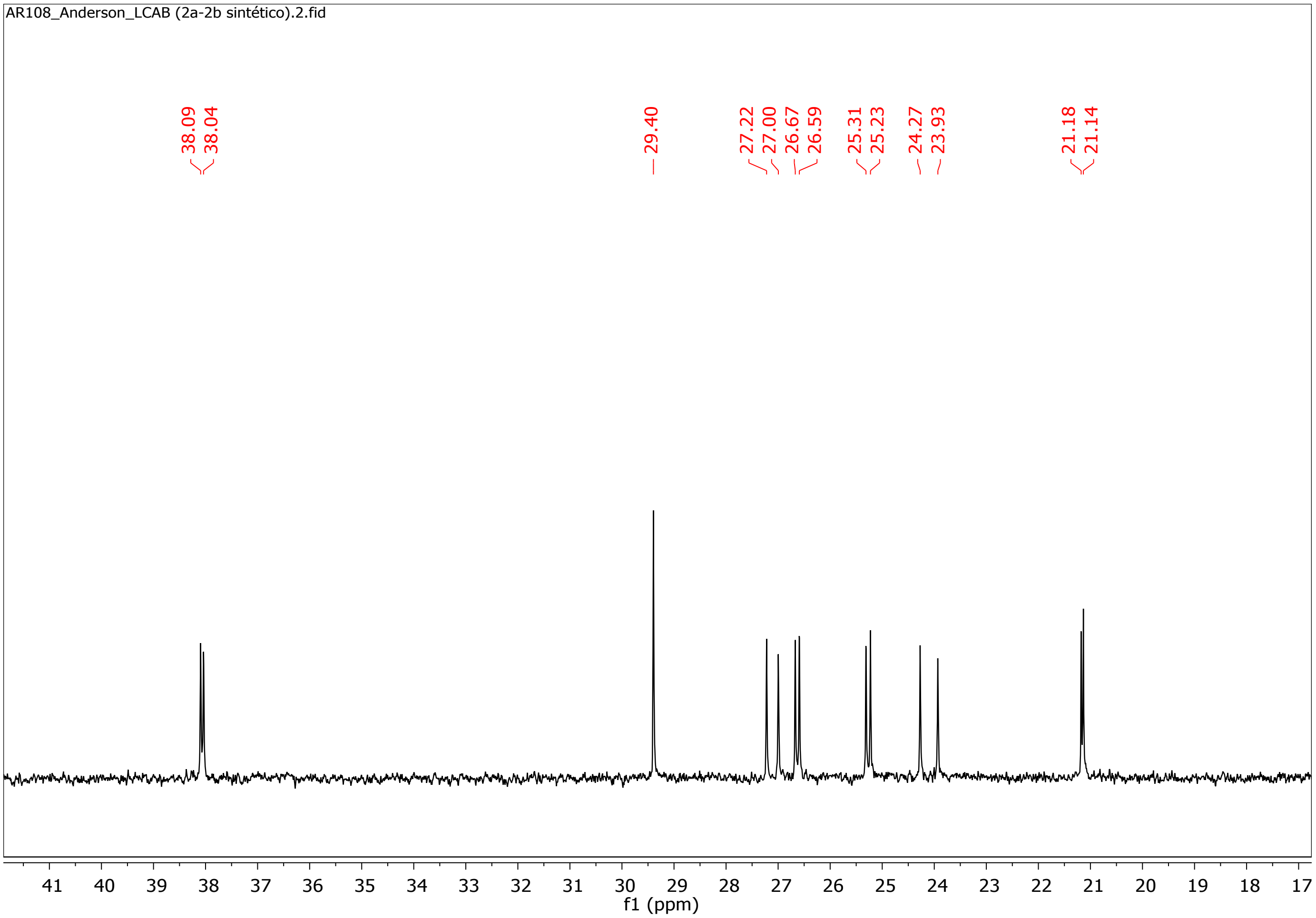

Figure S 64. Expansion of the ^{13}C NMR spectrum of compound **2*α*** (100 MHz, $CDCl_3$)

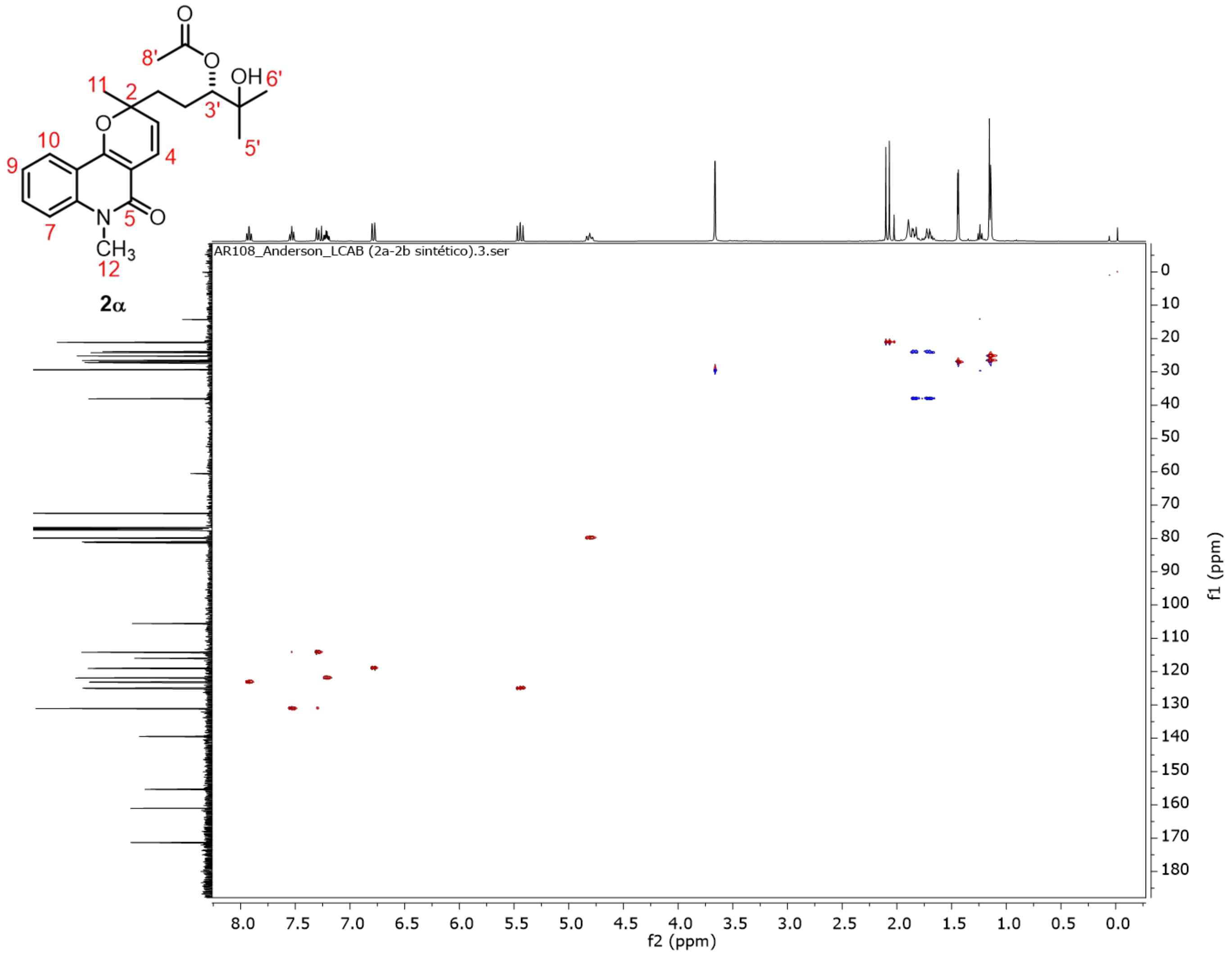

Figure S 65. HSQC spectrum of compound **2α** (1H: 400 MHz, ^{13}C: 100 MHz, $CDCl_3$)

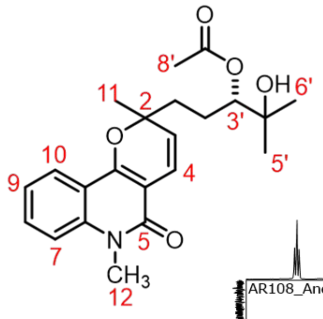

2α

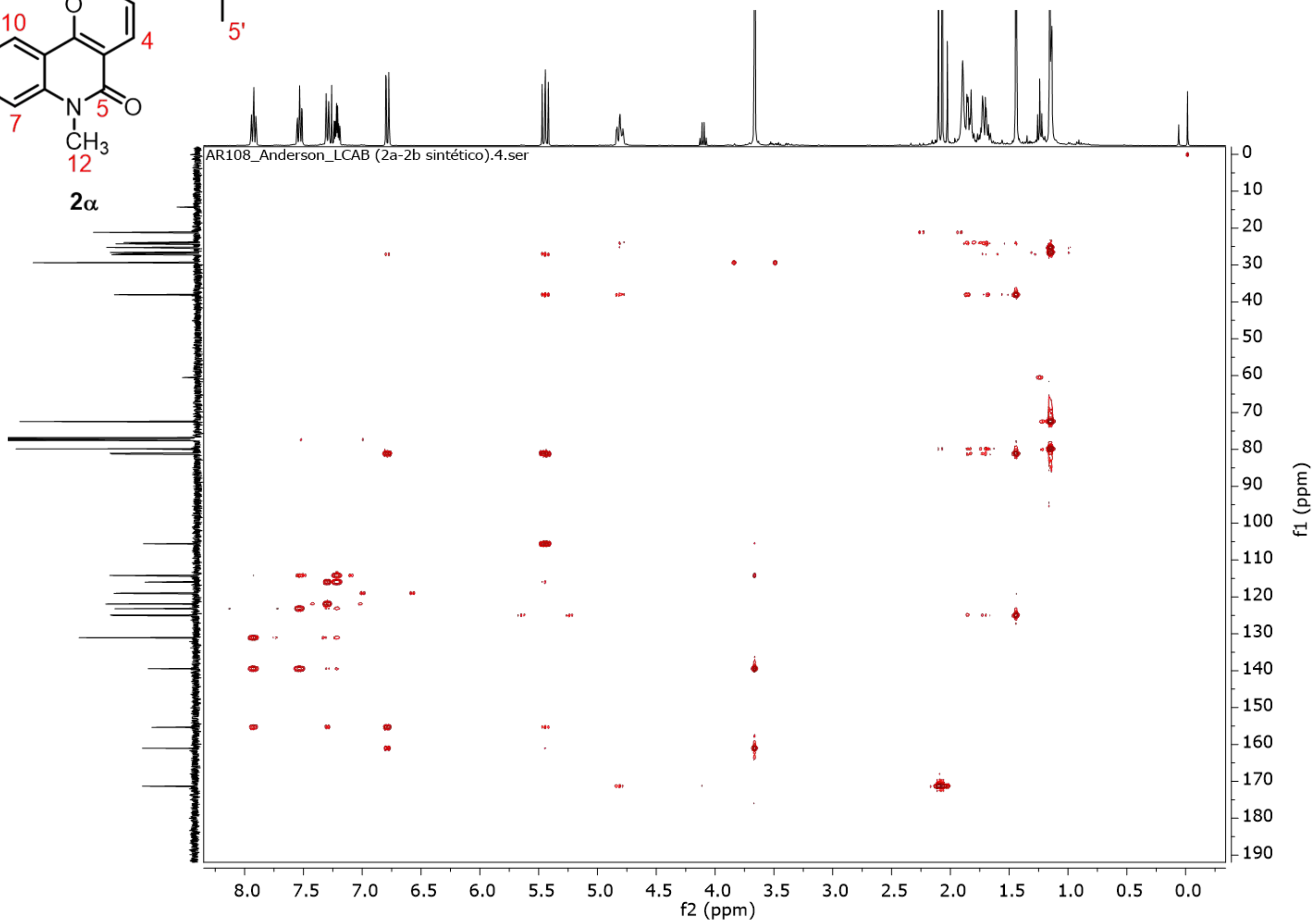

Figure S 66. HMBC spectrum of compound **2*α*** (^{1}H: 400 MHz, ^{13}C: 100 MHz, $CDCl_3$)

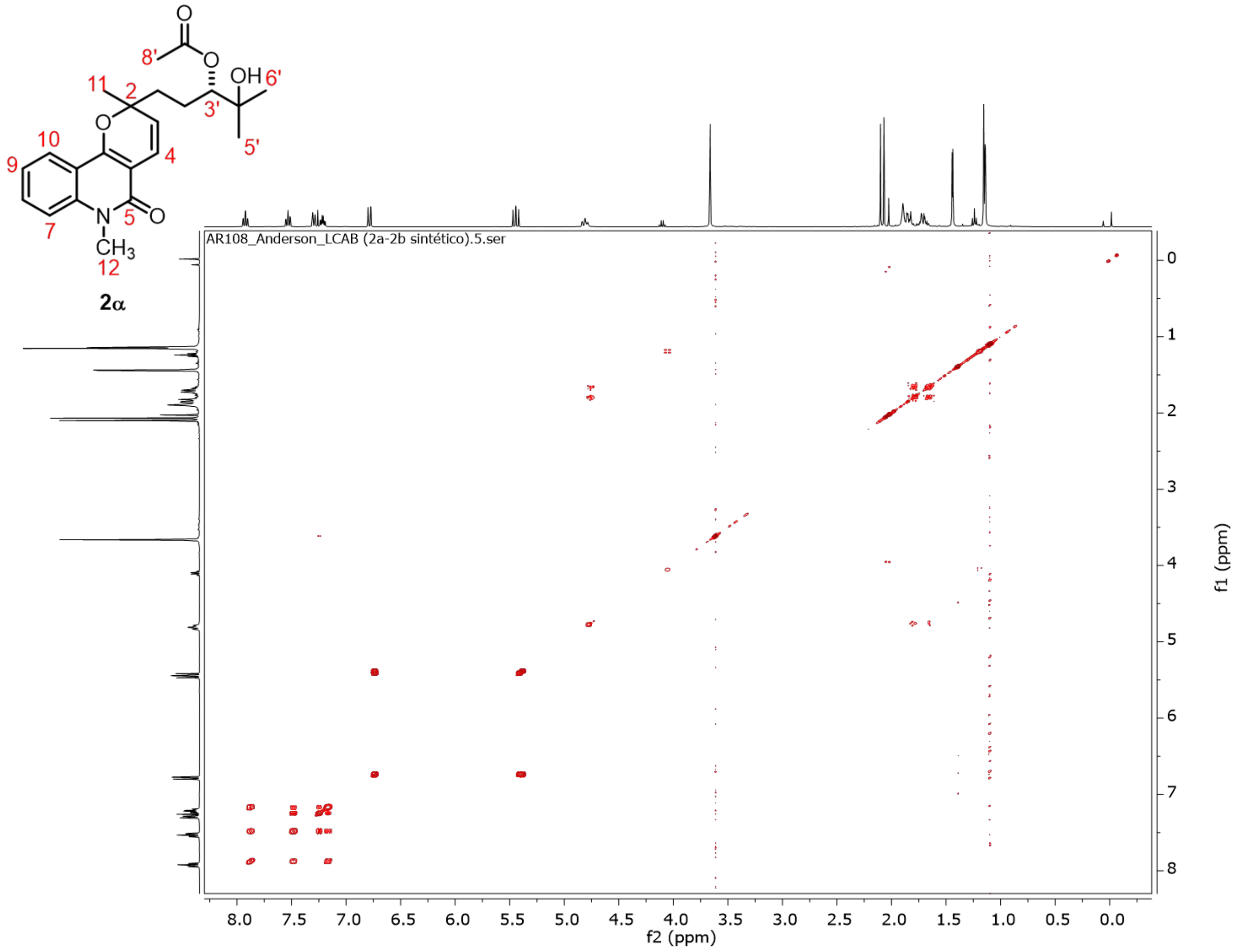

Figure S 67. COSY spectrum of compound **2α** (400 MHz, $CDCl_3$)

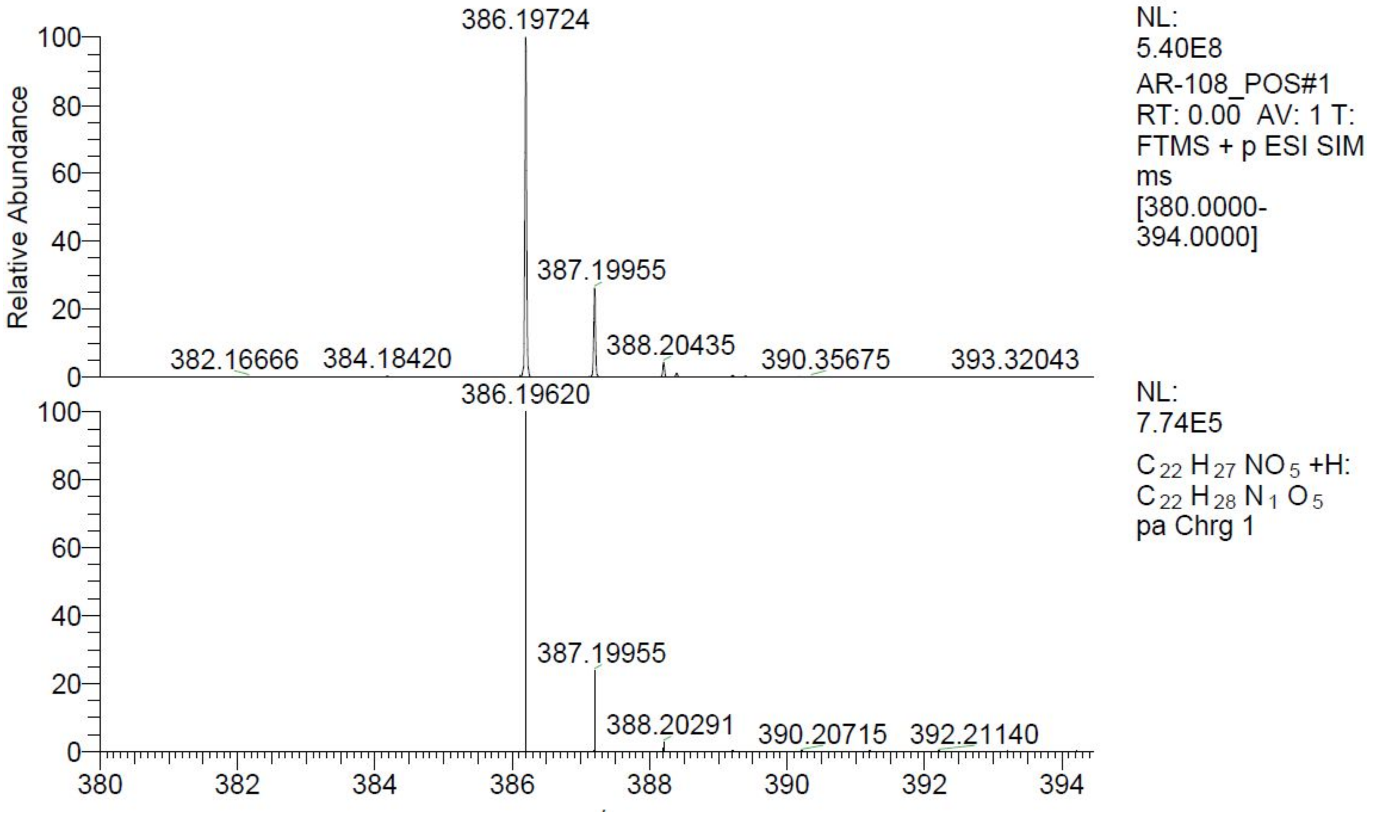

Figure S 68. (+)-HRESIMS spectrum of compound **2*α***

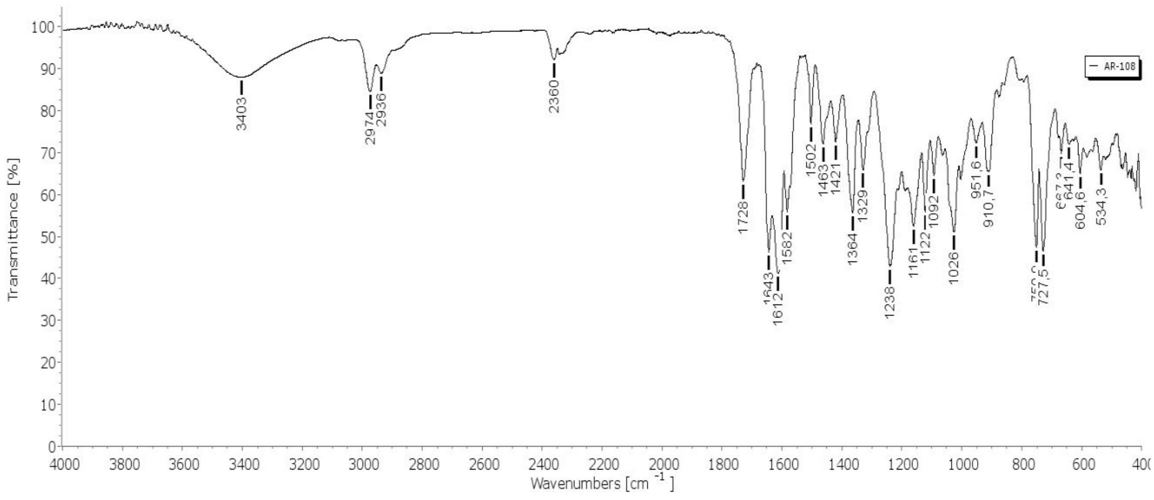

Figure S 69. IR spectrum of compound 2*α*

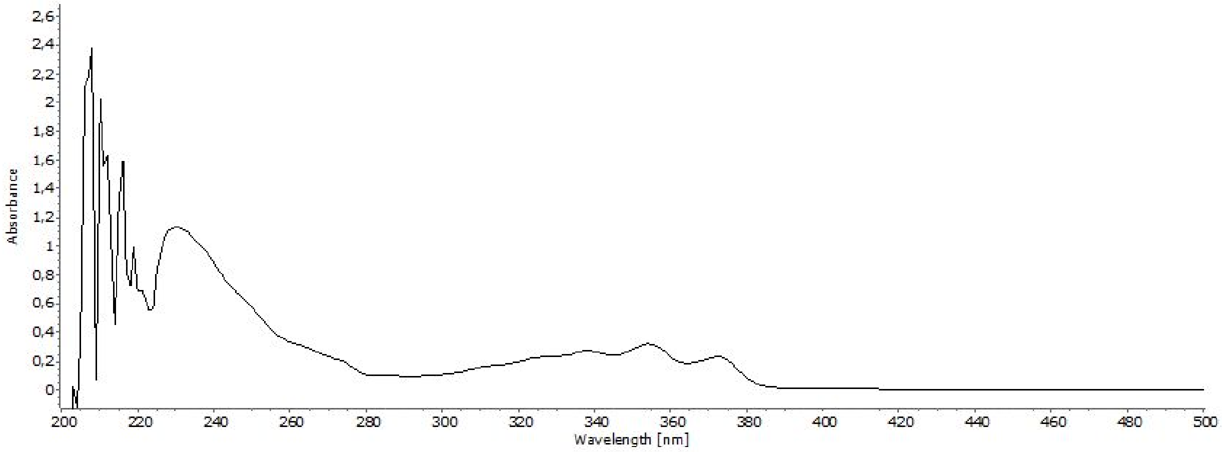

Figure S 70. spectrum (15 µg/mL, CH_2Cl_2) of compound 2*α*

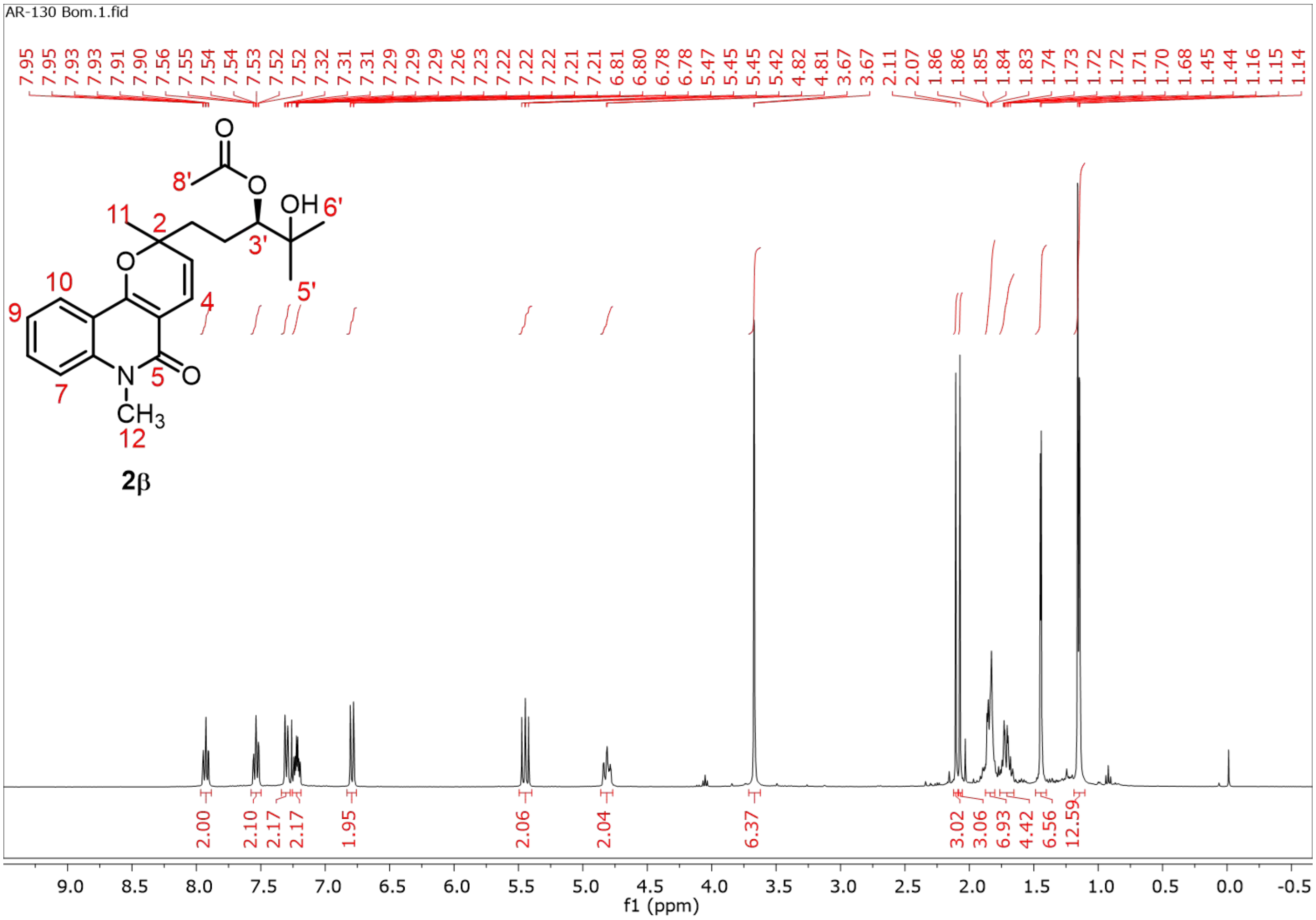

Figure S 71. ^{1}H NMR spectrum of compound **2*β*** (400 MHz, $CDCl_3$)

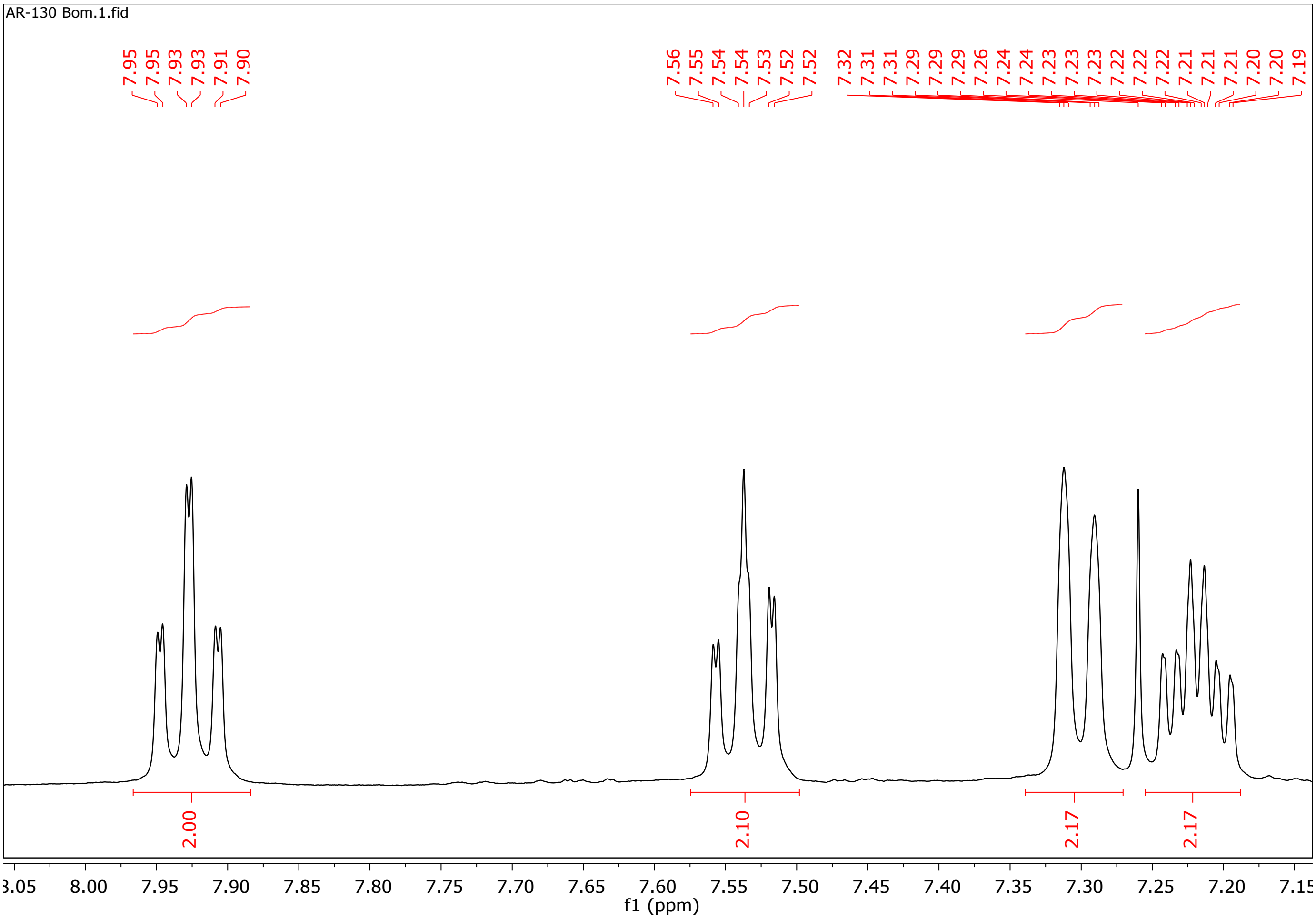

Figure S 72. Expansion 1 of the ^{1}H NMR spectrum of compound **2*β*** (400 MHz, $CDCl_3$)

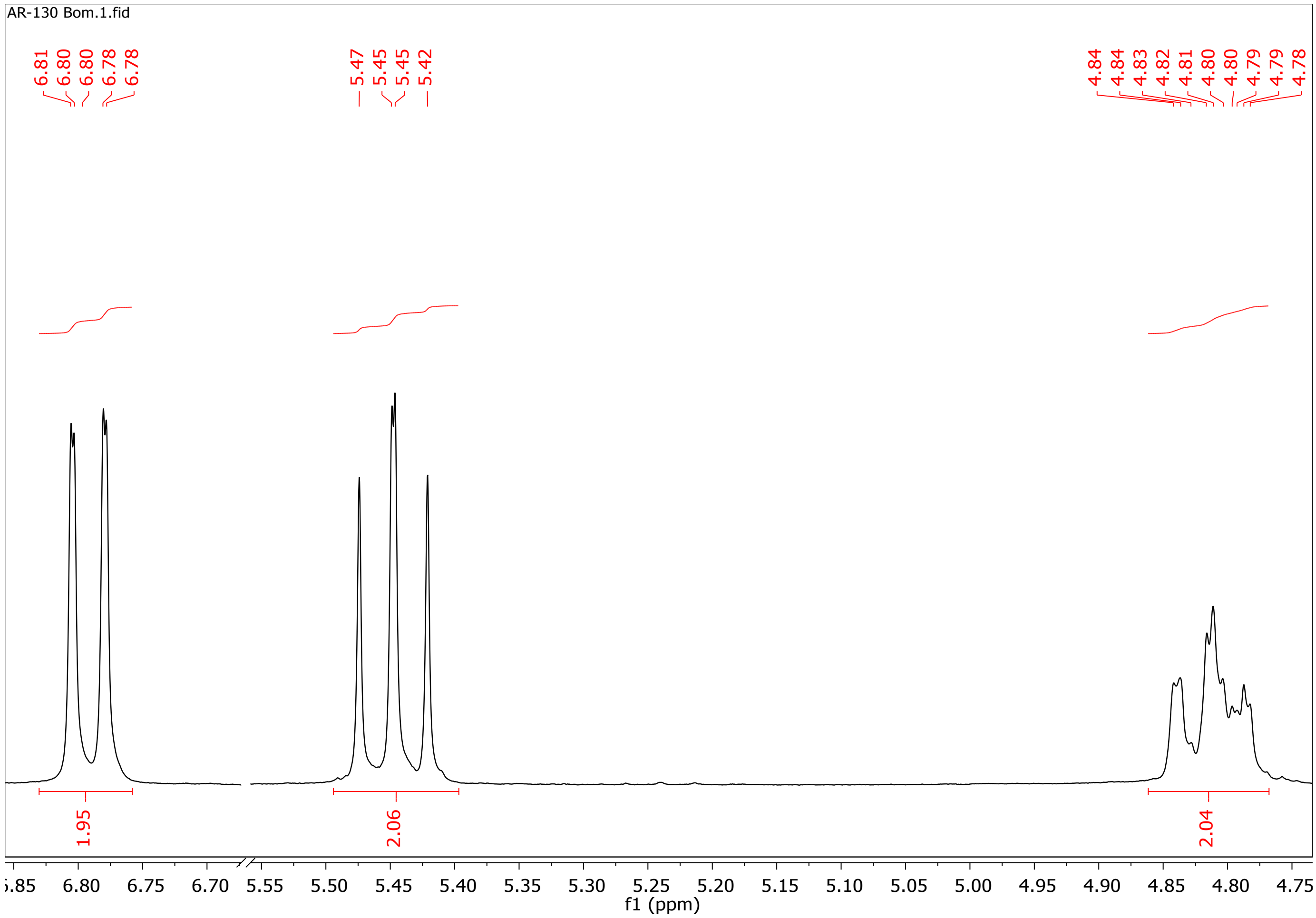

Figure S 73. Expansion 2 of the ^{1}H NMR spectrum of compound **2β** (400 MHz, $CDCl_3$)

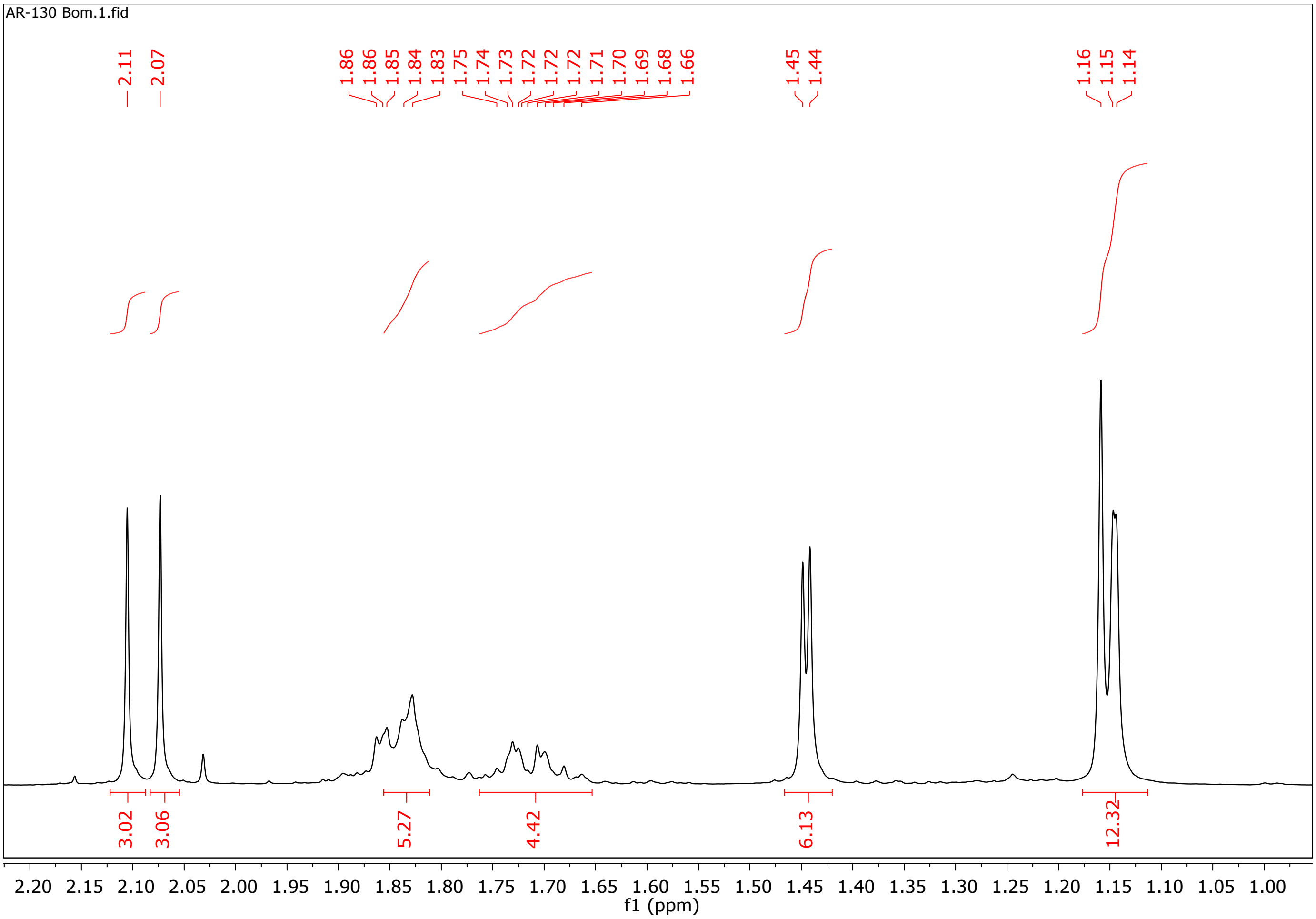

Figure S 74. Expansion 3 of the ^{1}H NMR spectrum of compound **2*β*** (400 MHz, $CDCl_3$)

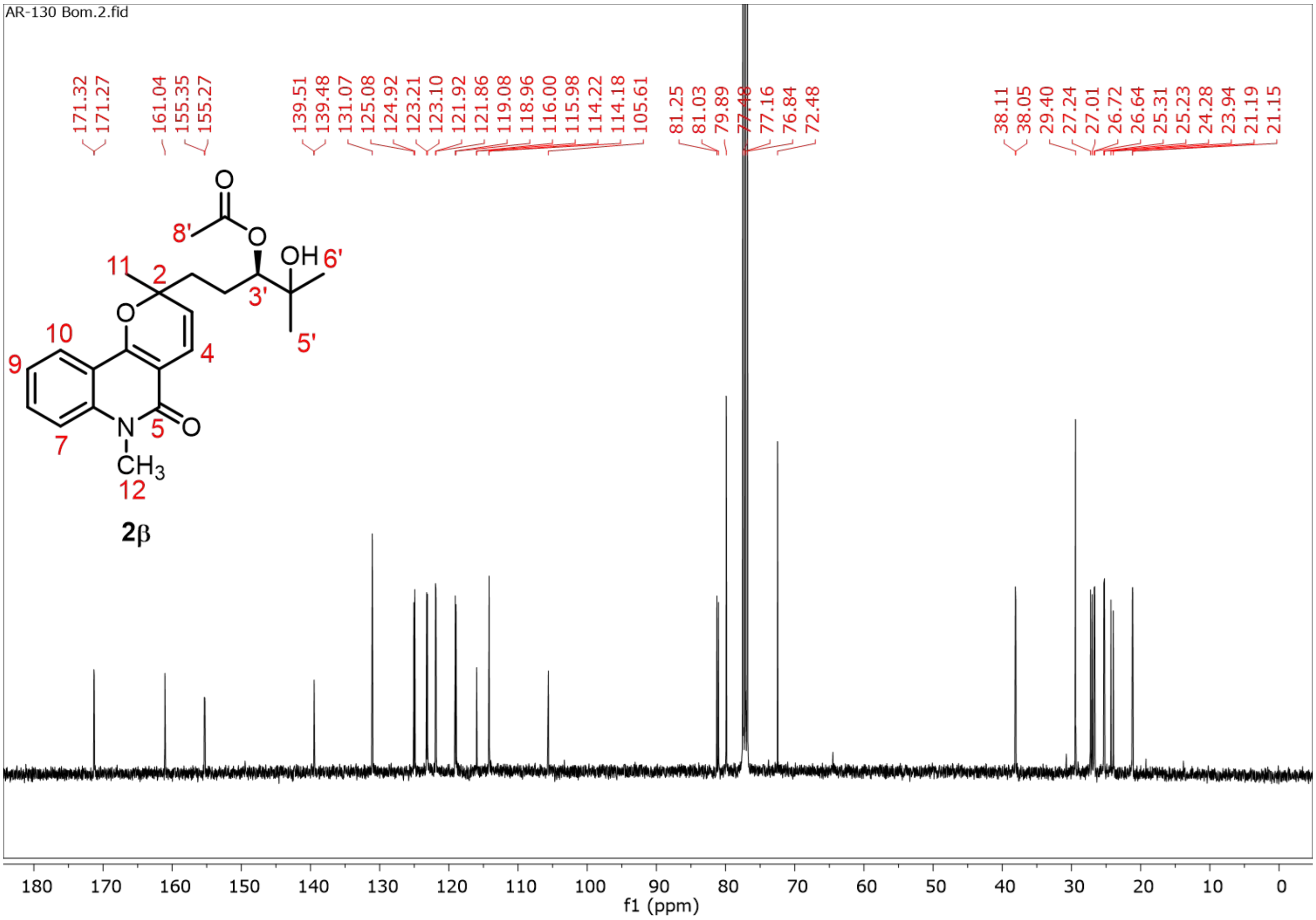

Figure S 75. ^{13}C NMR spectrum of compound **2*β*** (100 MHz, $CDCl_3$)

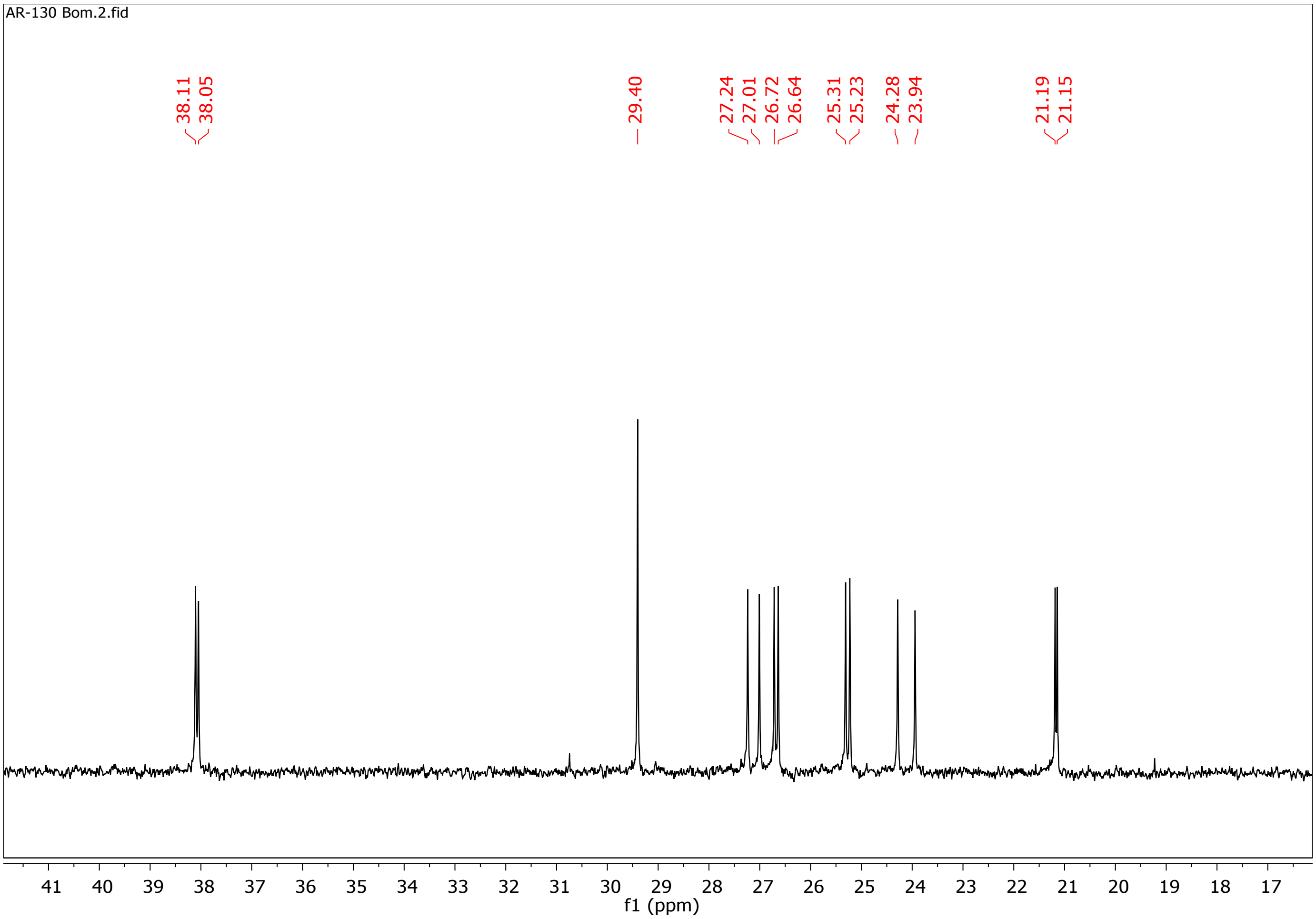

Figure S 76. Expansion of the ^{13}C NMR spectrum of compound **2*β*** (100 MHz, $CDCl_3$)

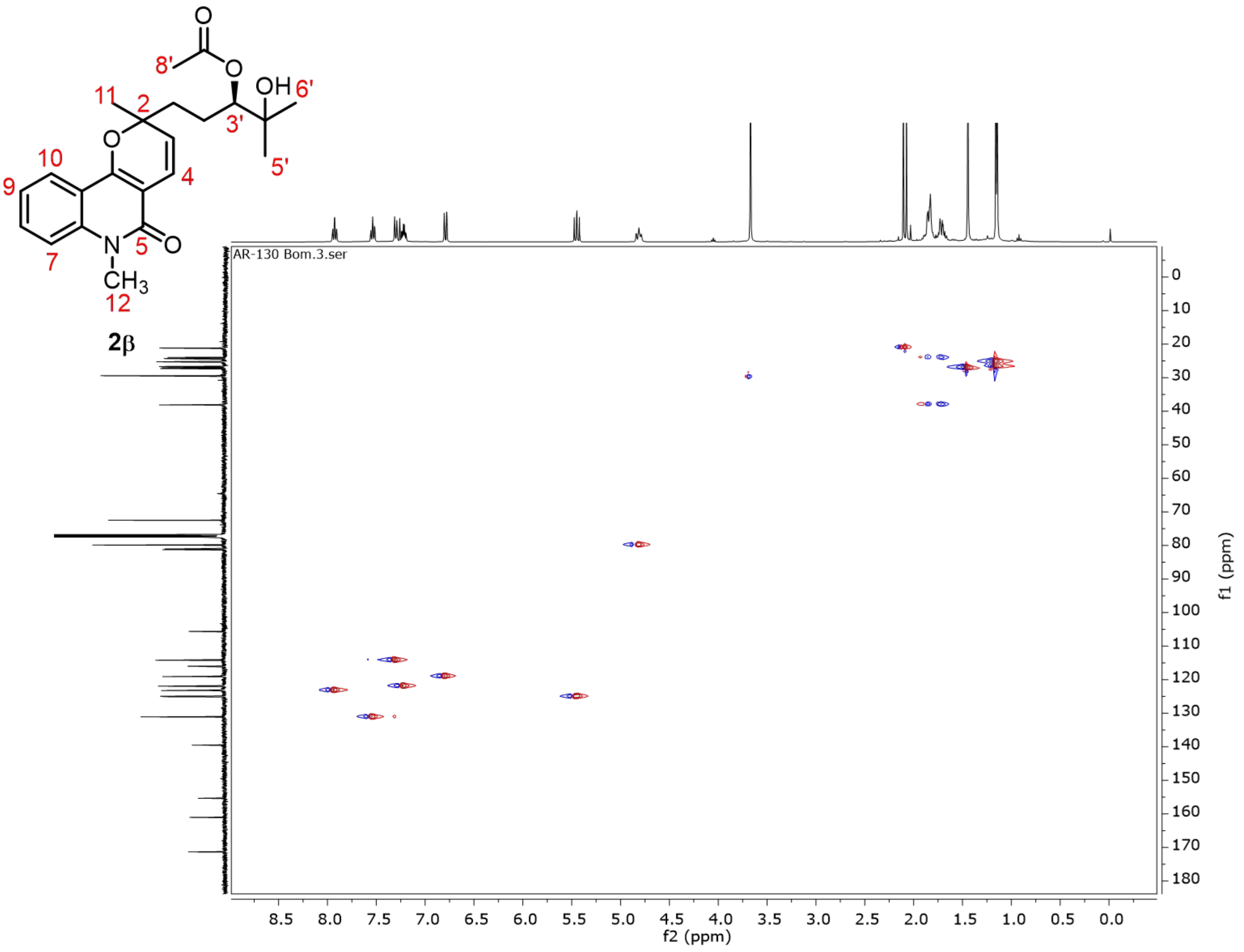

Figure S 77. HSQC spectrum of compound **2*β*** (^{1}H: 400 MHz, ^{13}C: 100 MHz, $CDCl_3$)

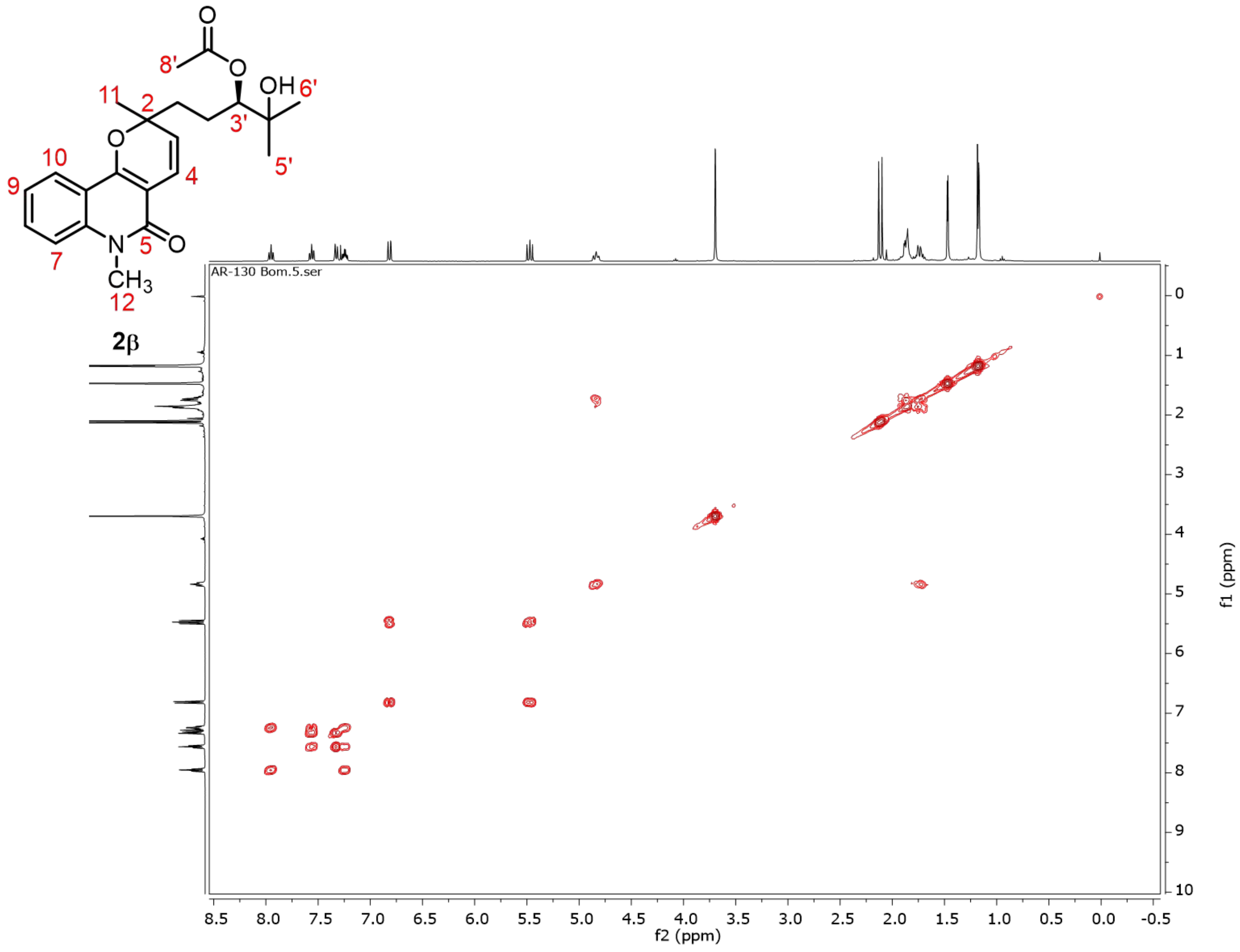

Figure S 78. COSY spectrum of compound **2*β*** (400 MHz, $CDCl_3$)

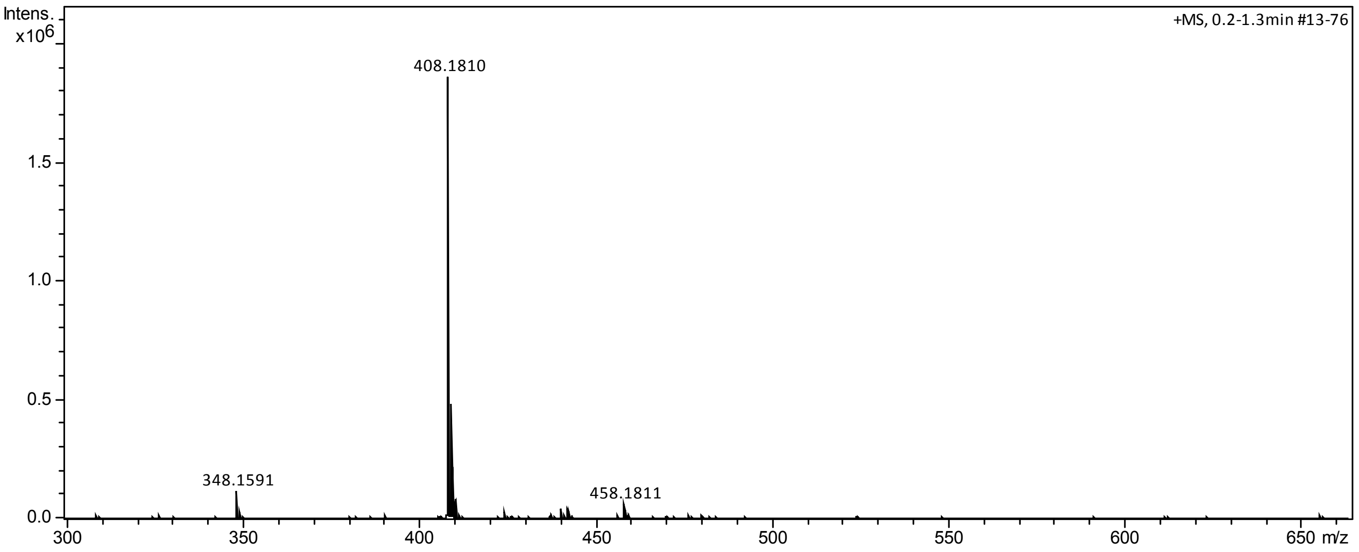

Figure S 79. (+)-HRESIMS spectrum of compound **2*β***

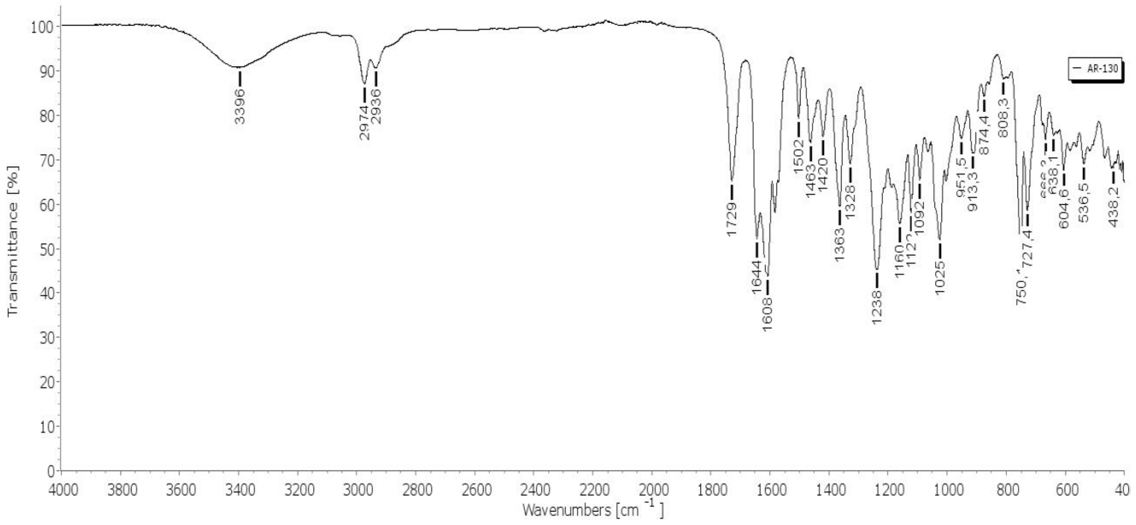

Figure S 80. IR spectrum of compound **2*β***

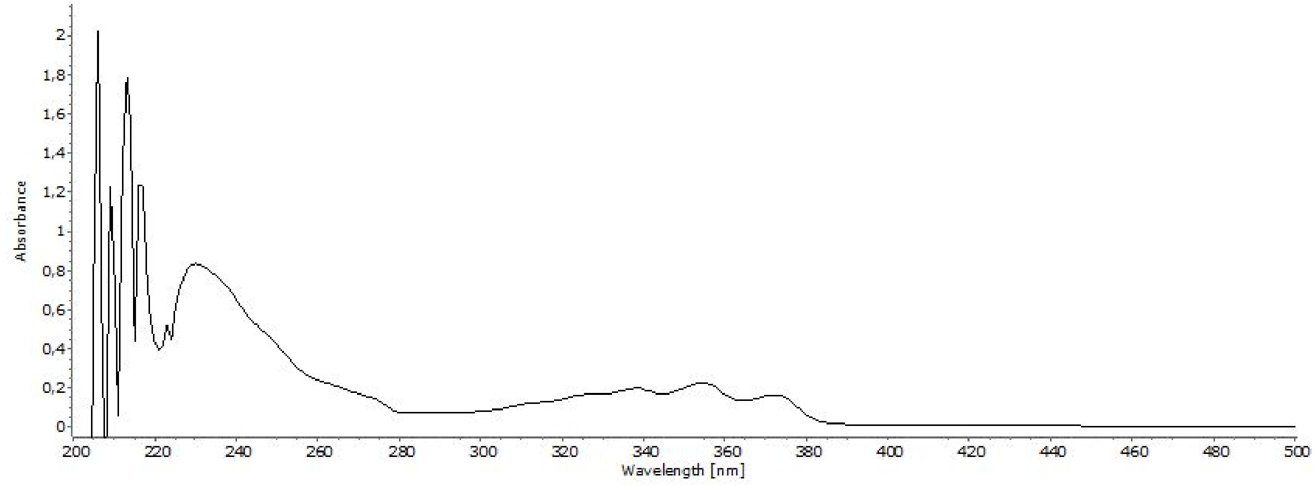

Figure S 81. UV spectrum (17 µg/mL, CH_2Cl_2) of compound **2*β***

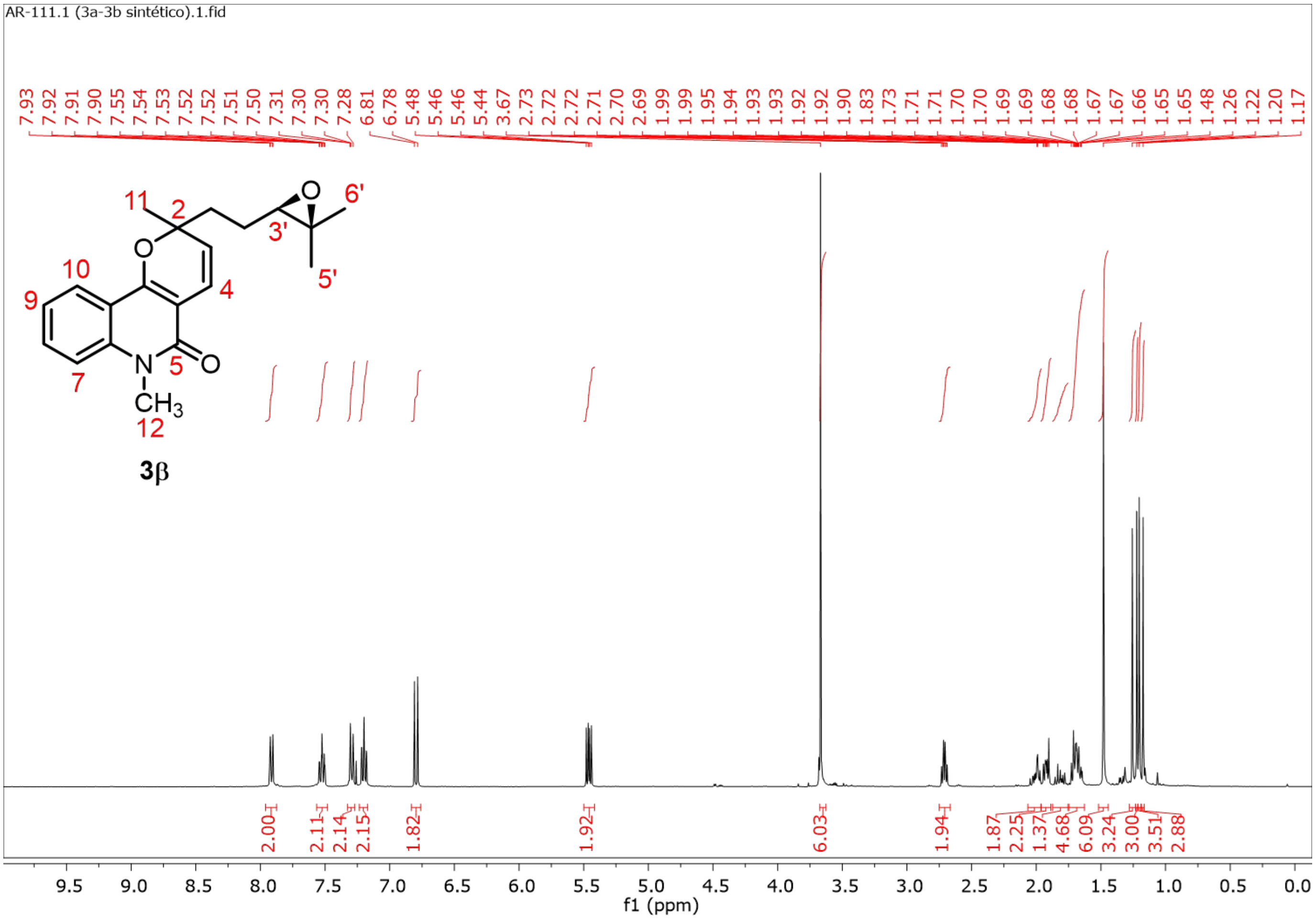

Figure S 82. ^{1}H NMR spectrum of compound **3β** (400 MHz, $CDCl_3$)

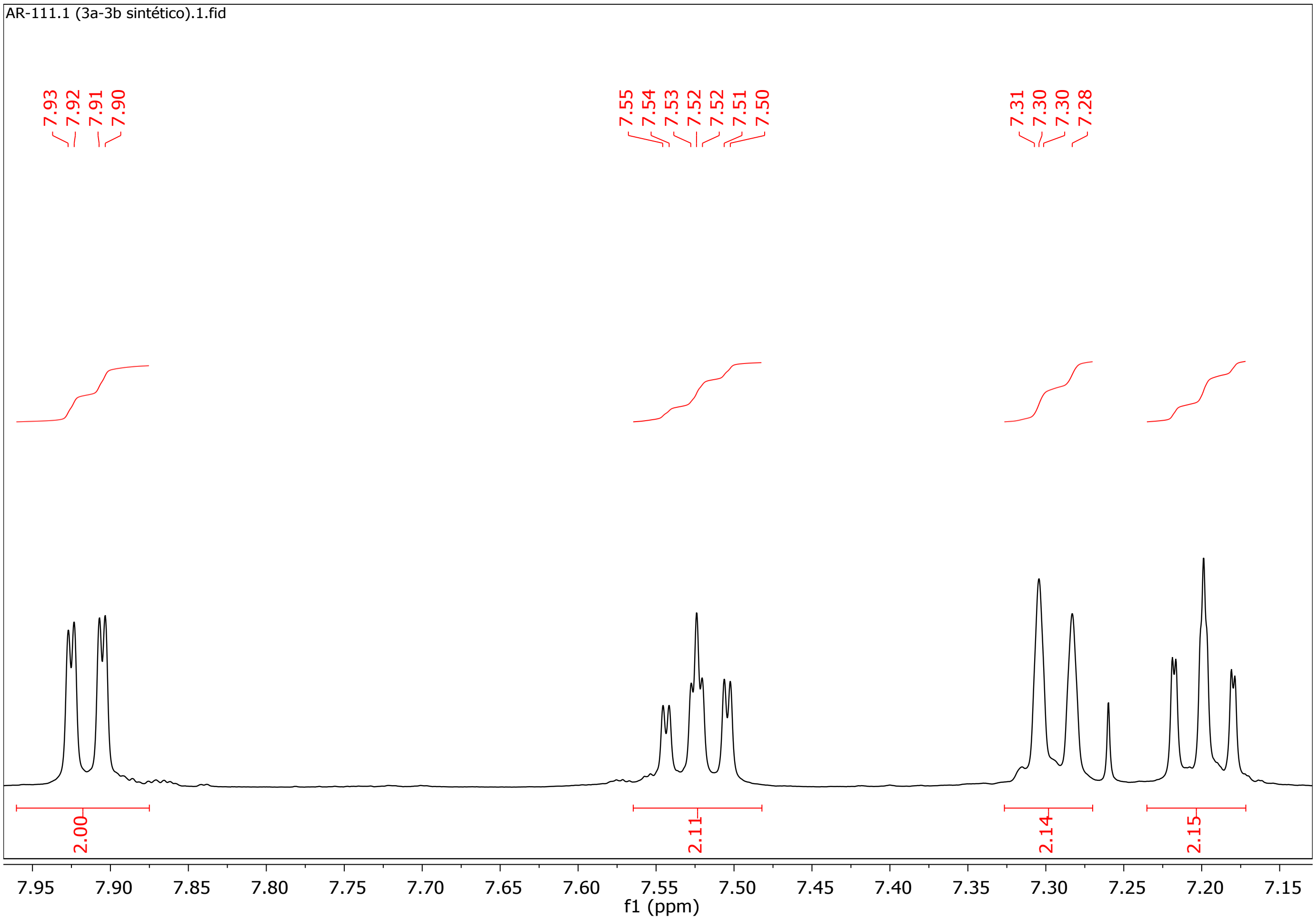

Figure S 83. Expansion 1 of the ^{1}H NMR spectrum of compound **3β** (400 MHz, $CDCl_3$)

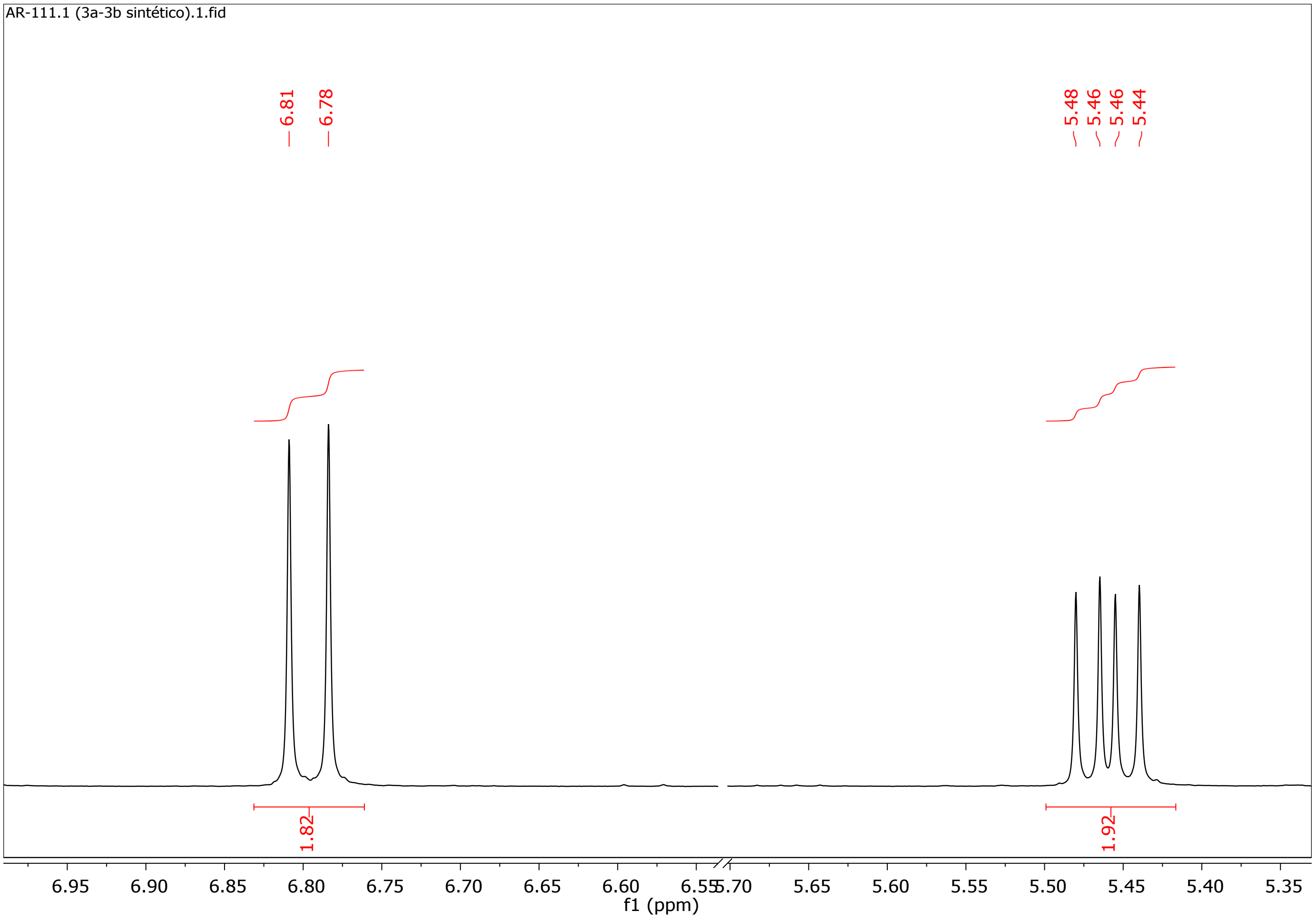

Figure S 84. Expansion 2 of the ^{1}H NMR spectrum of compound **3*β*** (400 MHz, $CDCl_3$)

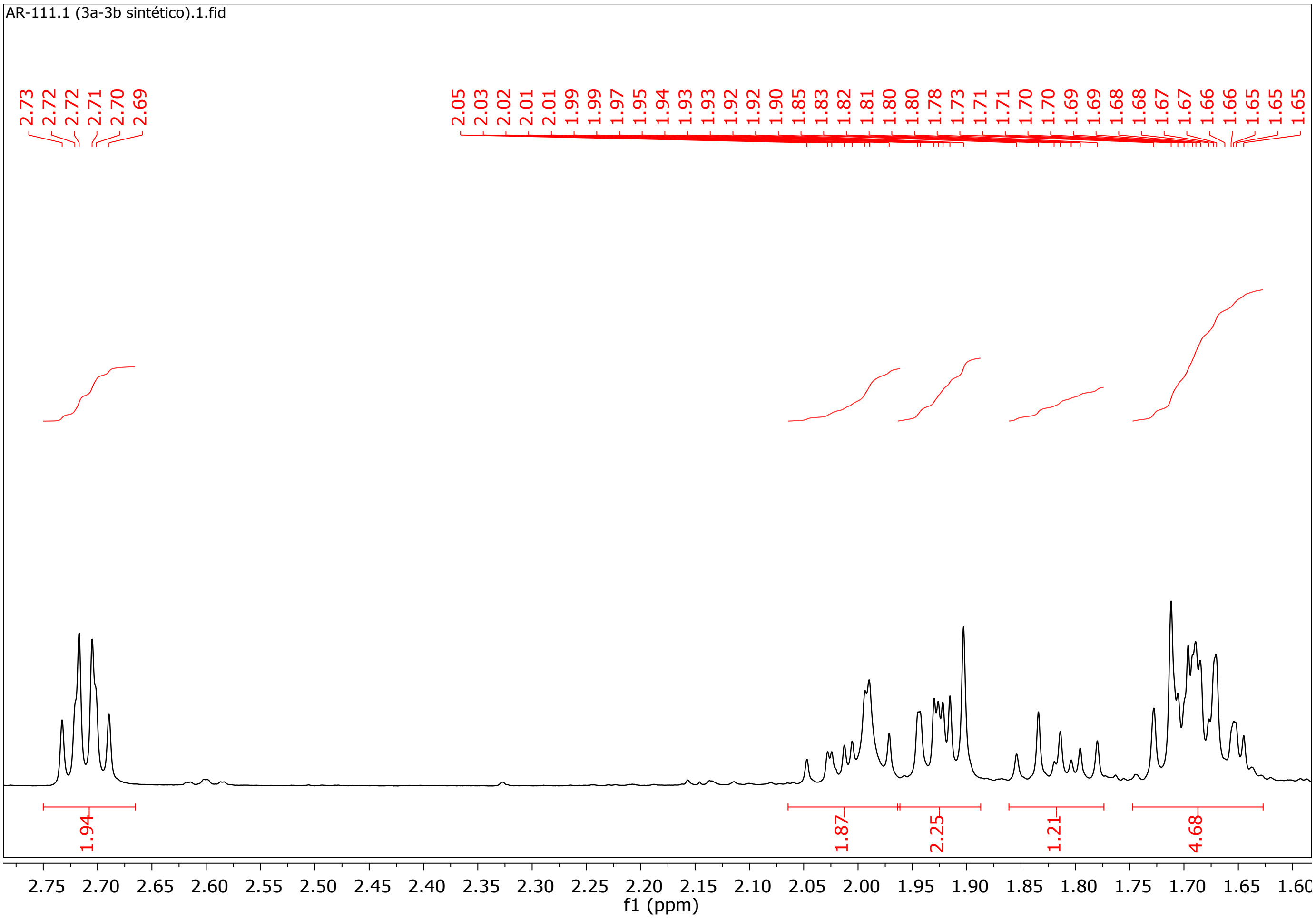

Figure S 85. Expansion 3 of the ^{1}H NMR spectrum of compound **3*β*** (400 MHz, $CDCl_3$)

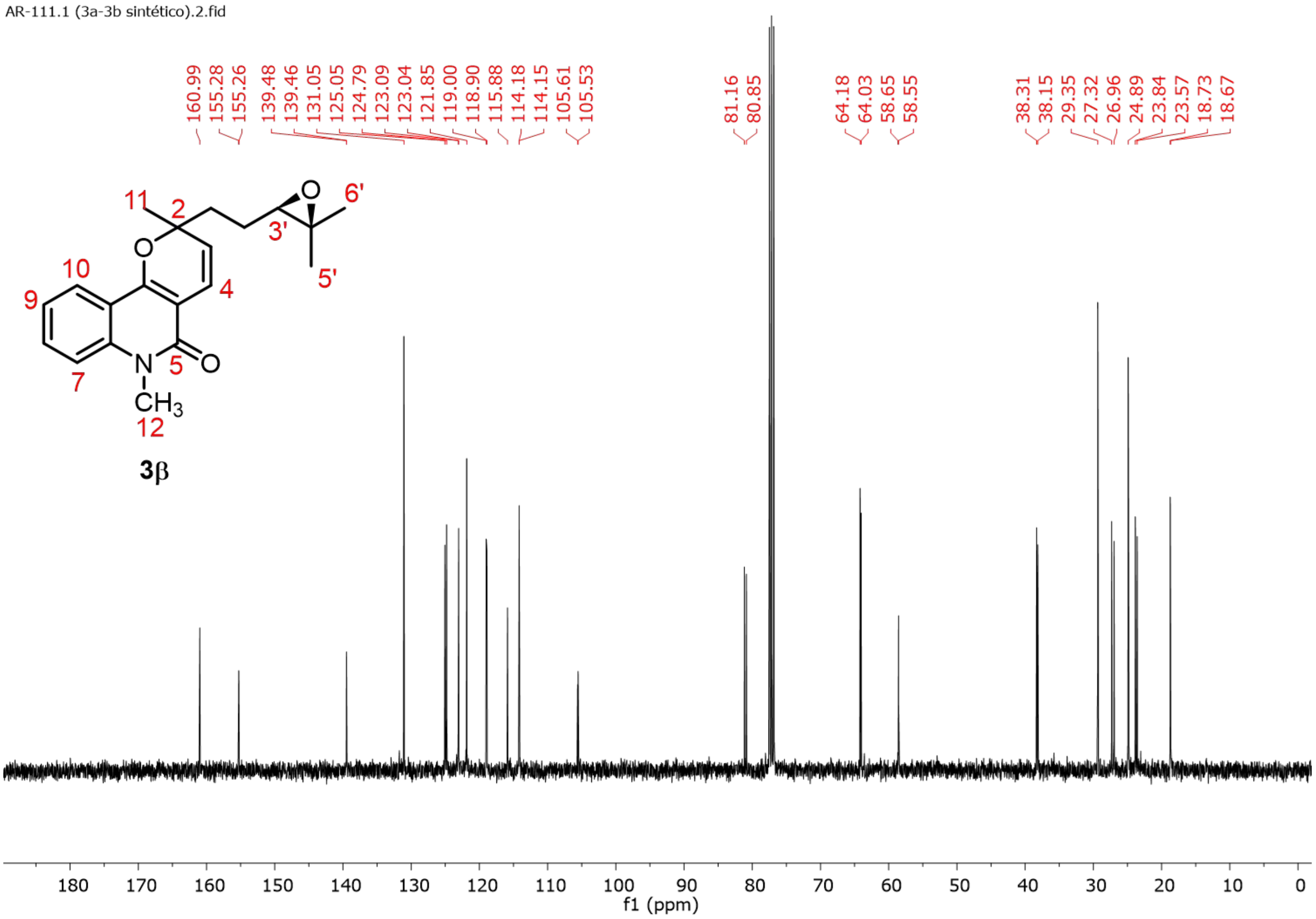

Figure S 86. ^{13}C NMR spectrum of compound **3β** (100 MHz, $CDCl_3$)

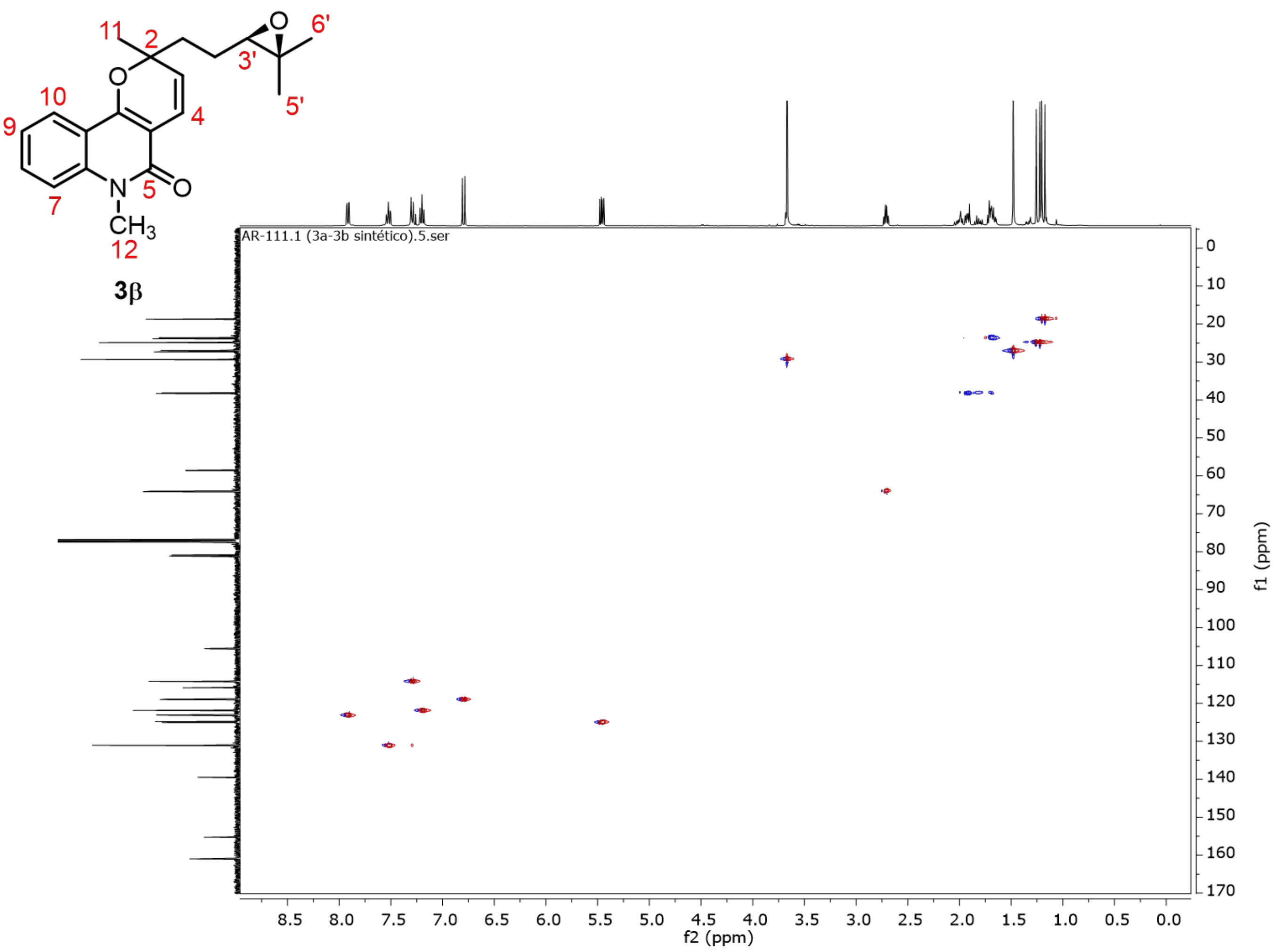

Figure S 87. HSQC spectrum of compound **3β** (^{1}H: 400 MHz, ^{13}C: 100 MHz, $CDCl_3$)

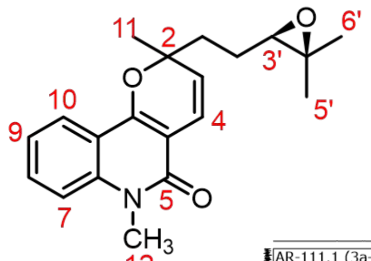

3β

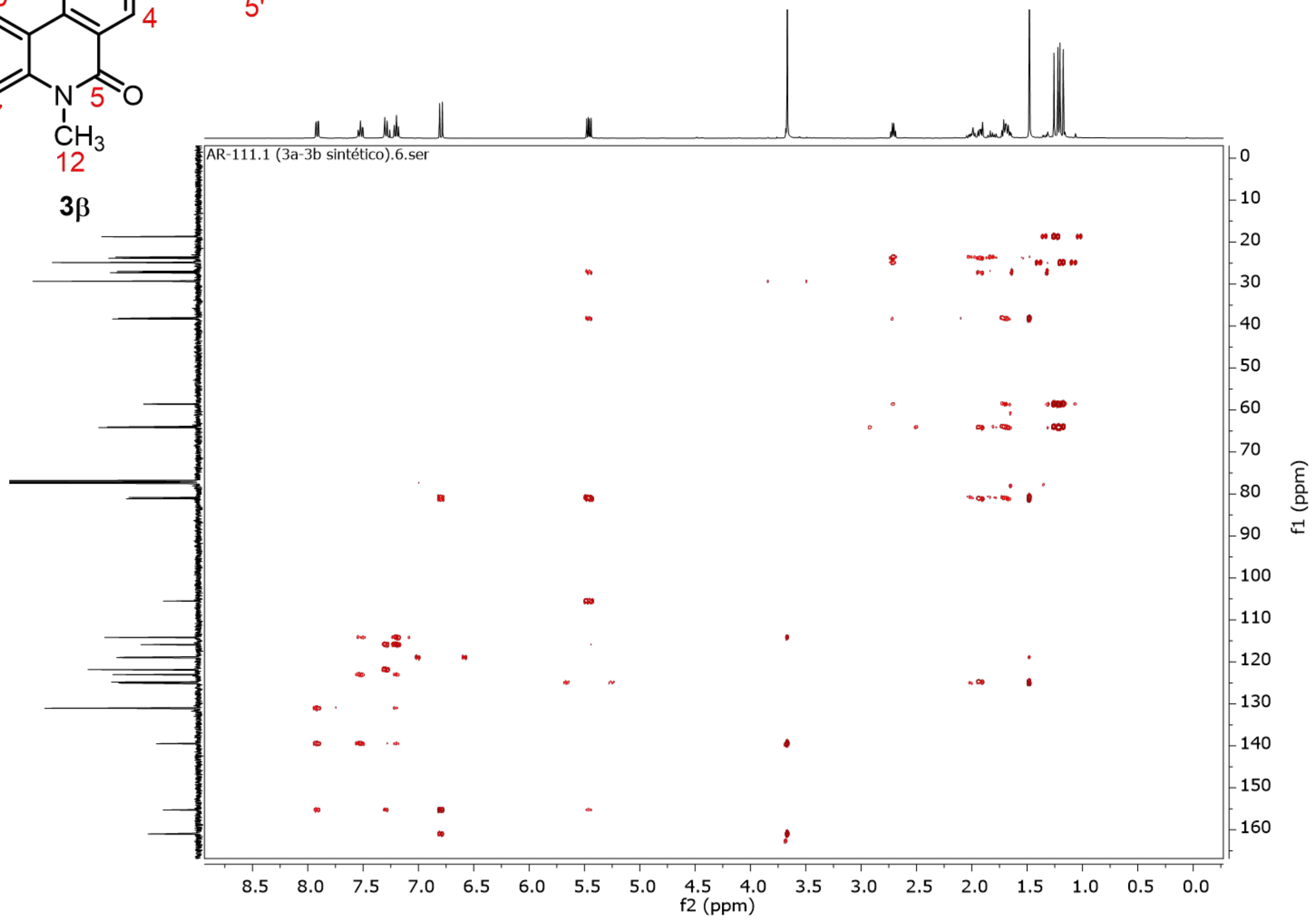

Figure S 88. HMBC spectrum of compound **3*β*** (^{1}H: 400 MHz, ^{13}C: 100 MHz, $CDCl_3$)

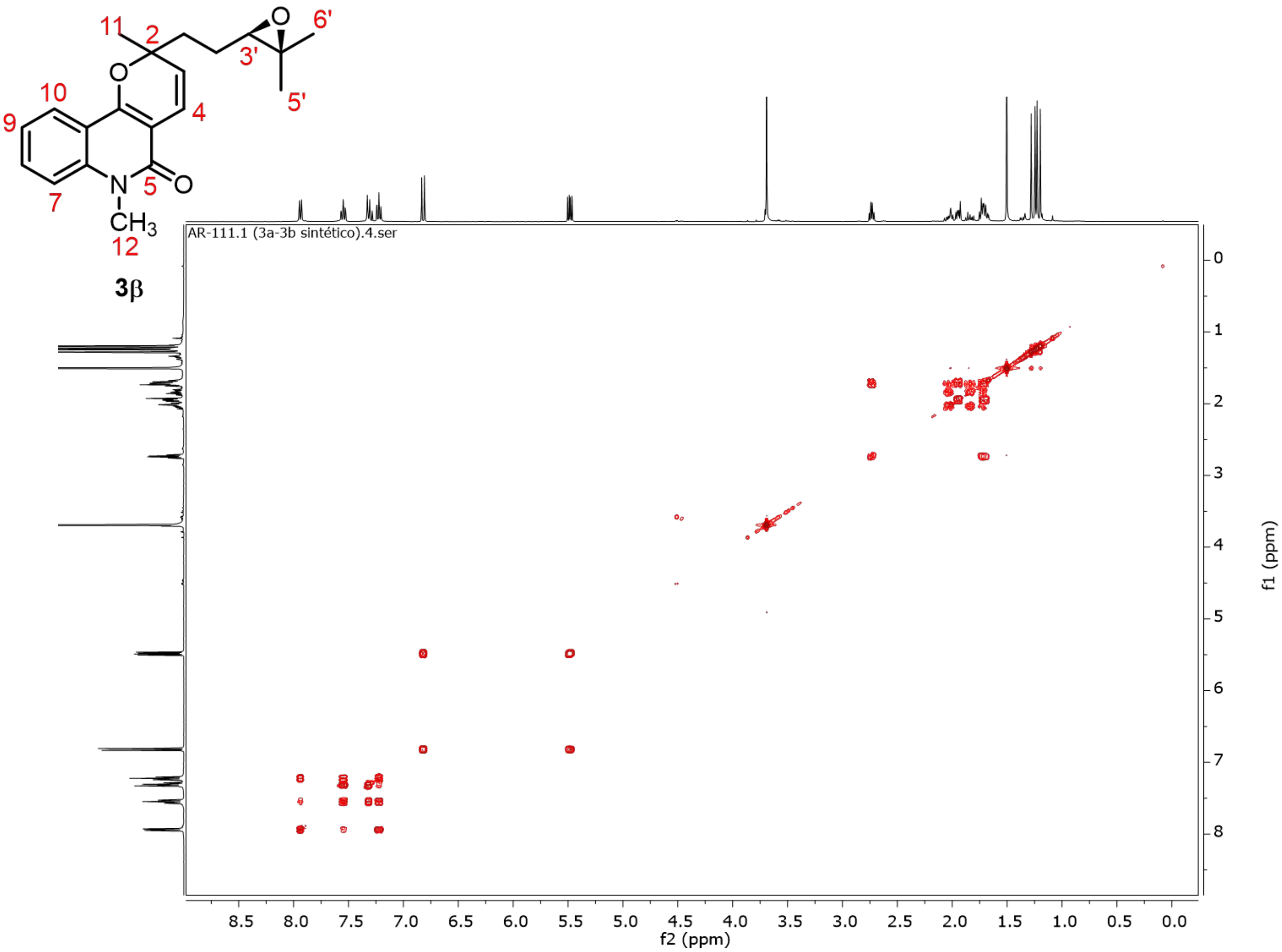

Figure S 89. COSY spectrum of compound **3*β*** (400 MHz, $CDCl_3$)

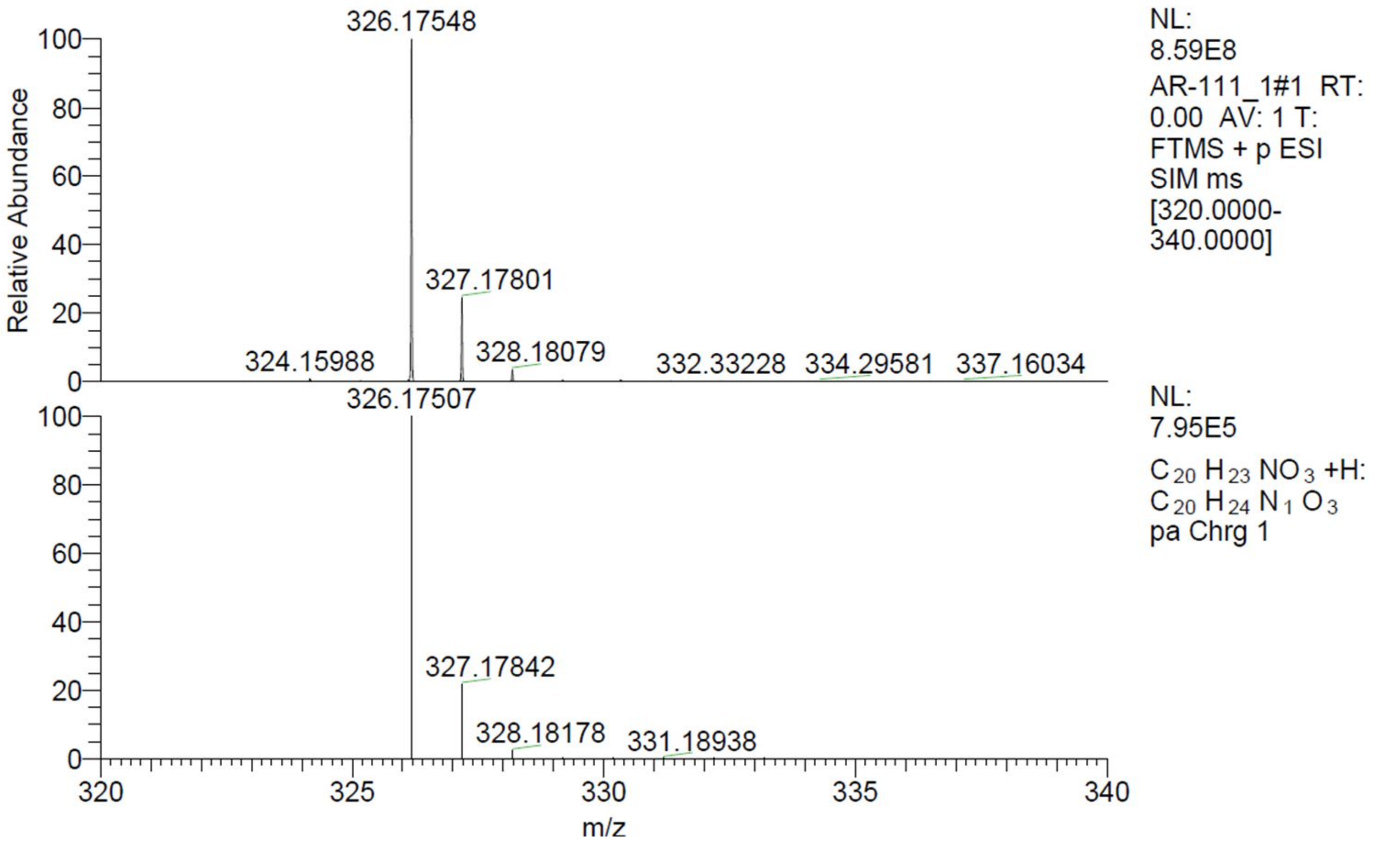

Figure S 90. (+)-HRESIMS spectrum of compound **3*β***

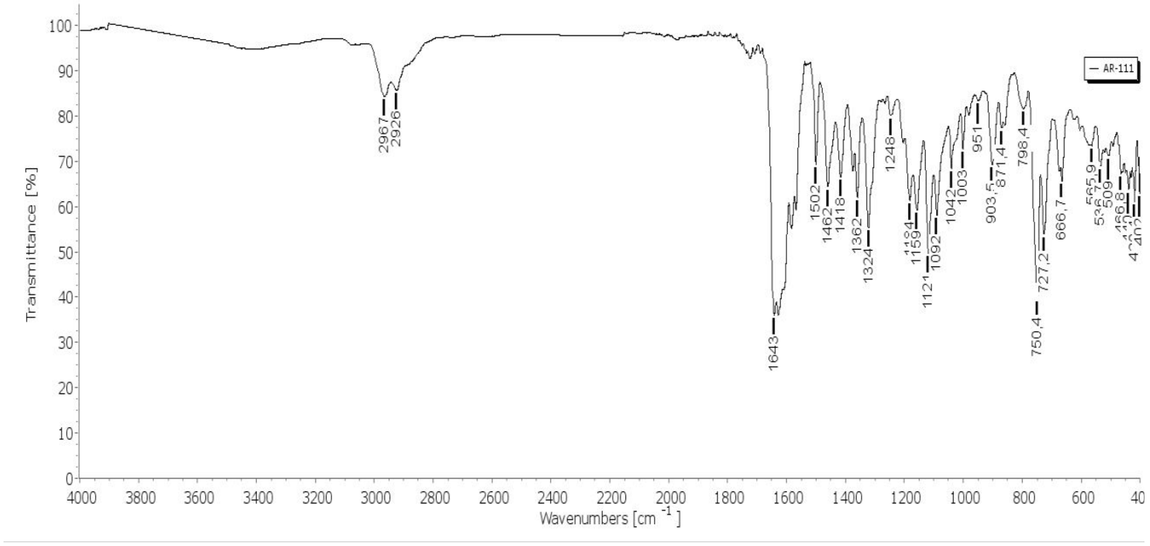

Figure S 91. IR spectrum of compound **3*β***

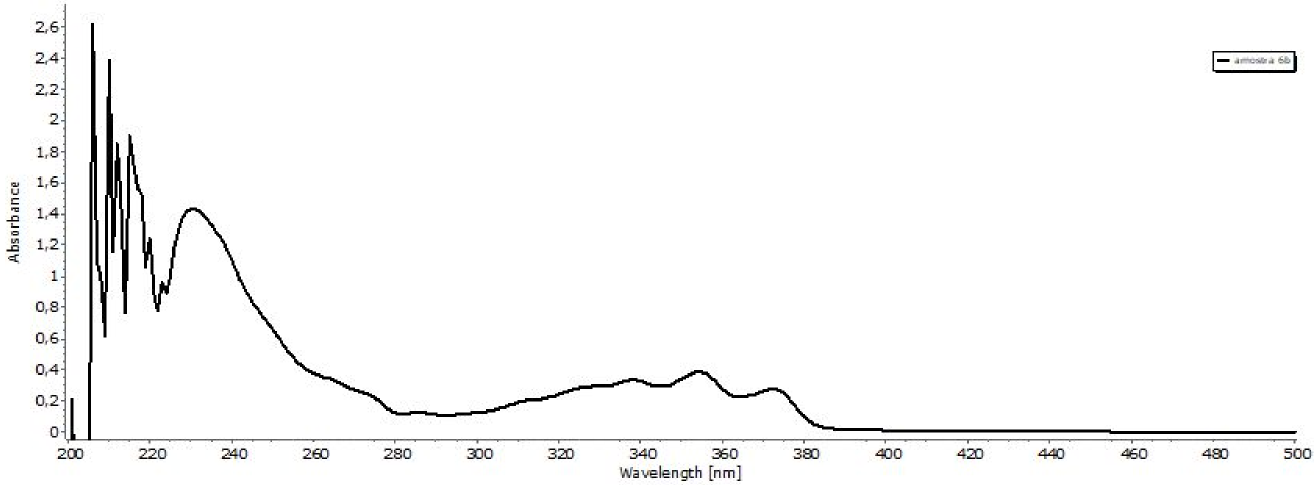

Figure S 92. UV spectrum (15 mg, CH_2Cl_2) of compound **3*β***

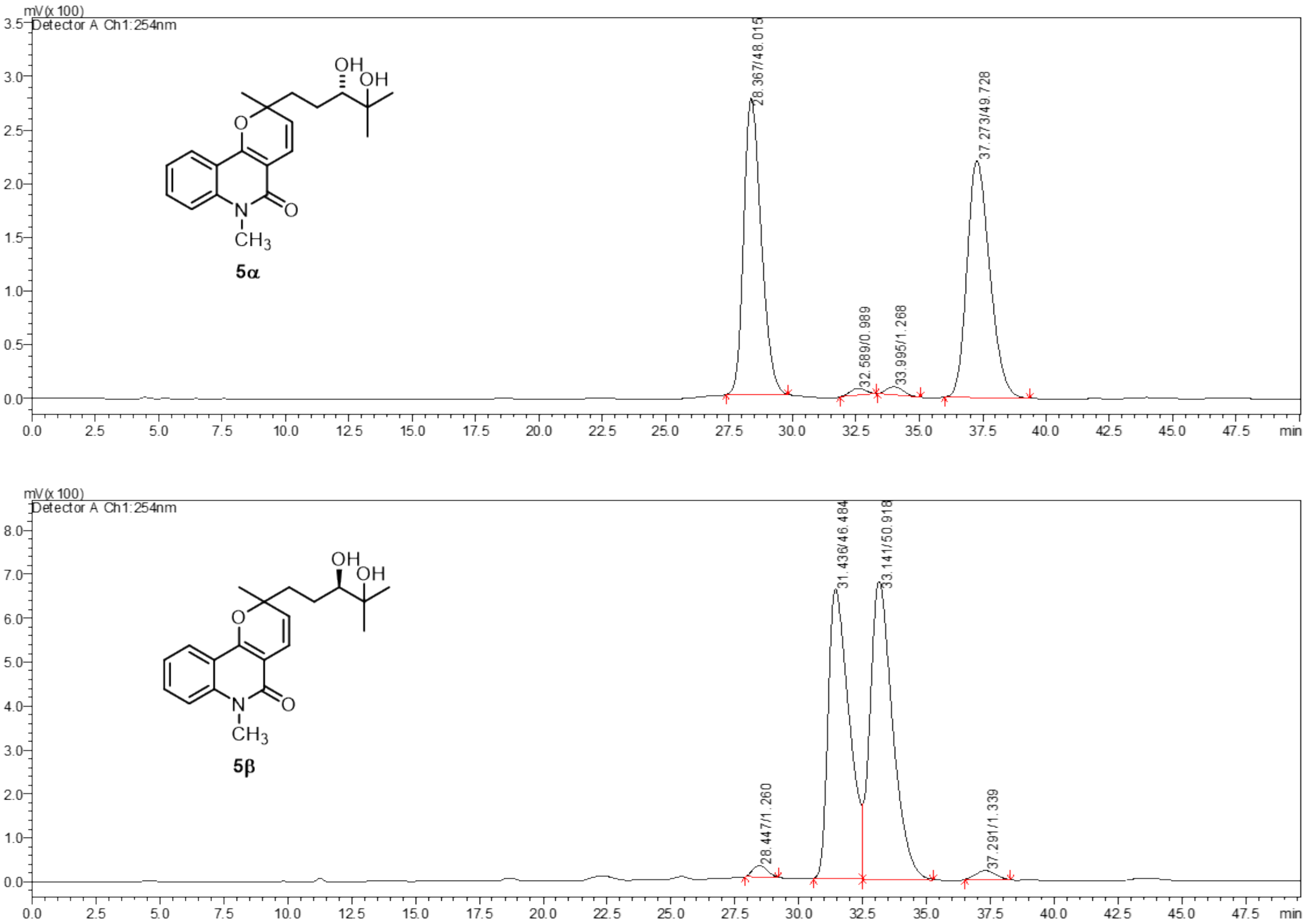

Figure S 93. Chiral phase HPLC Chromatogram of the synthetic compounds **5*α*** and **5*β***

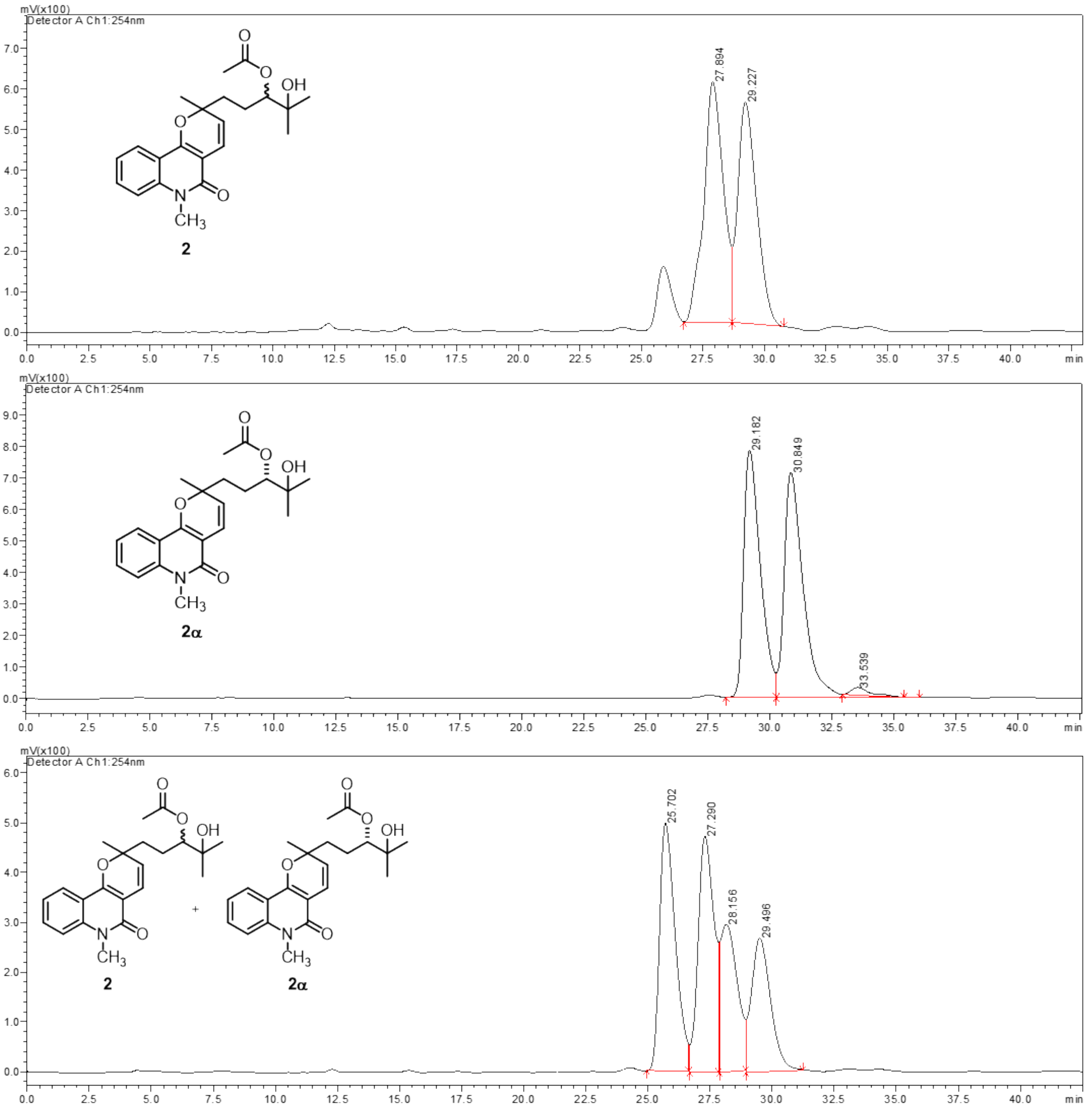

Figure S 94. Chiral phase HPLC chromatogram of natural product **2**, synthetic compound **2*α***, and their mixture (**2** + **2*α***)

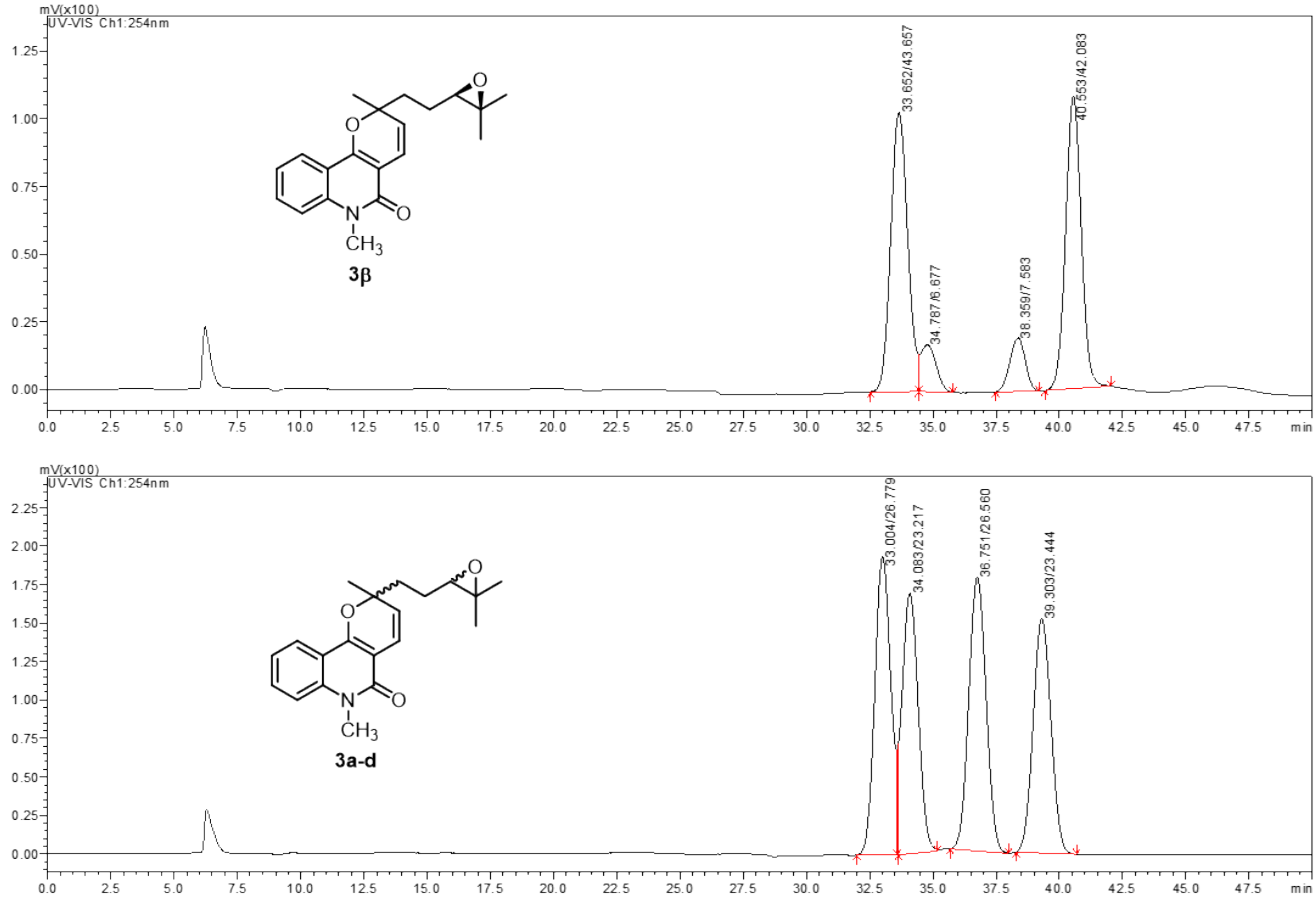

Figure S 95. Chiral phase HPLC chromatogram of synthetic compound **3*β*** and racemic synthetic compound

3a-d

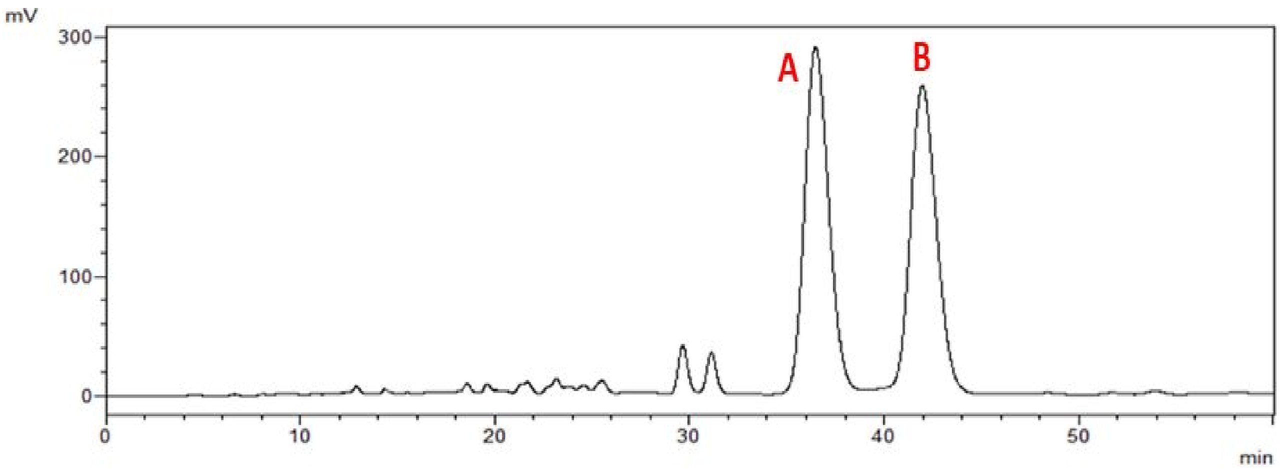

Figure S 96. Semipreparative HPLC chromatogram of natural product **2**

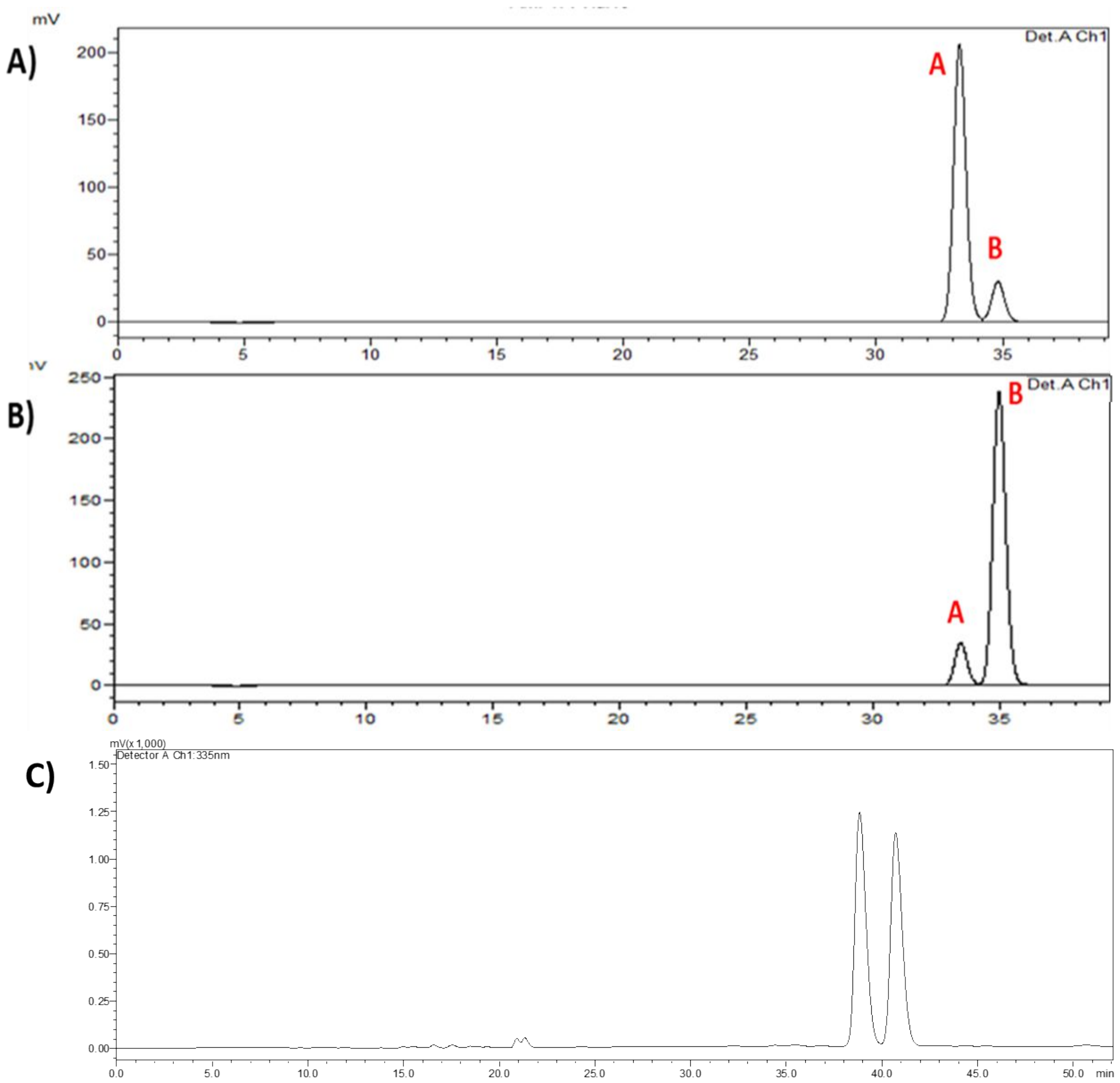

Figure S 97. Analytical HPLC chromatogram obtained from samples of epimer obtained from separation through semipreparative HPLC. A) Sample from diastereomer with the shortest retention time (here referred as A) 1 h after performing separation through semi-preparative HPLC. B) Sample from diastereomer with the longest retention time (here referred as B) 1 h after performing separation through semi-preparative

HPLC. C) Sample from diastereomer A several days after performing separation through semi-preparative HPLC (the sample was extracted with $CHCl_3$ and stored at 8 ˚C after solvent removal).

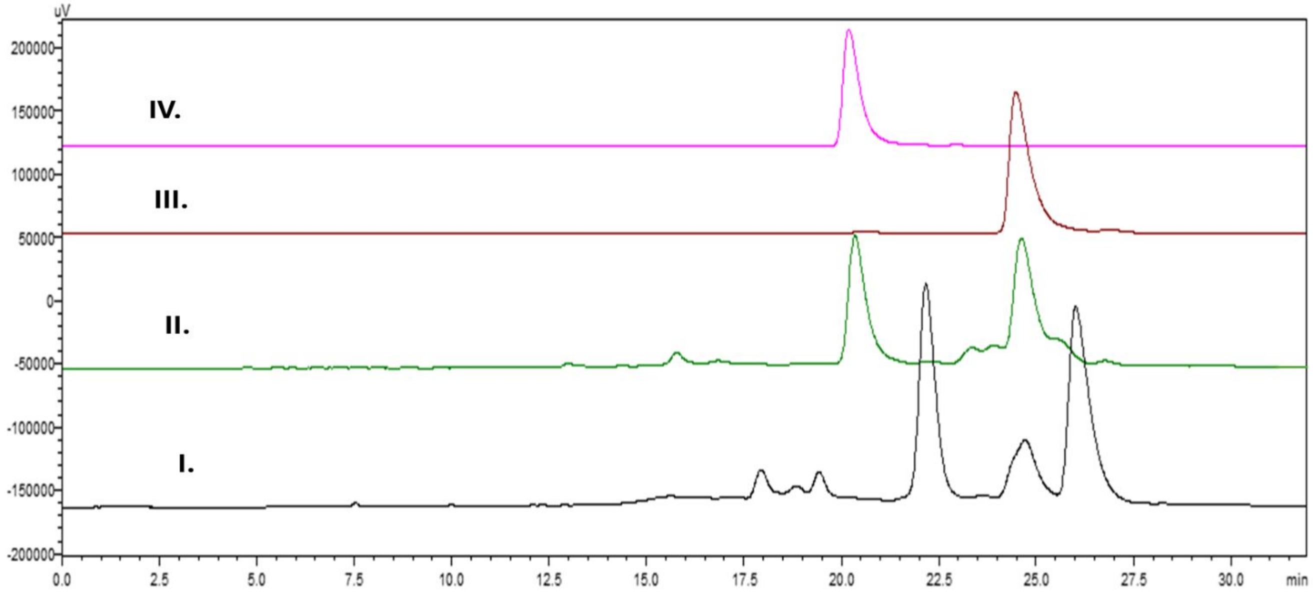

Figure S 98. Overlap of HPLC chromatograms of compound 4. I. Semipreparative column analysis. II. Analytical column analysis. III. and IV. Re-injection of the isolated diastereoisomers into the analytical column immediately after separation.

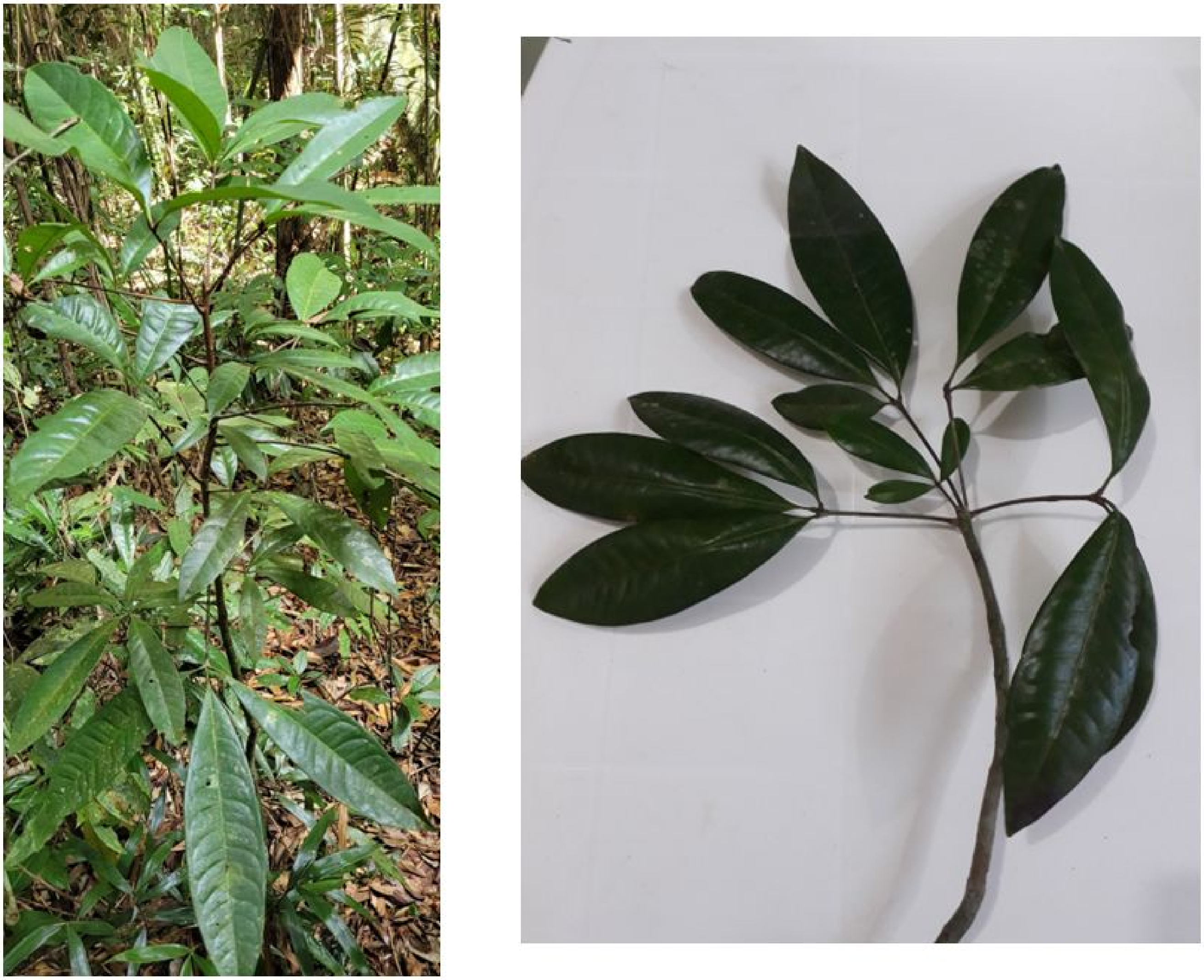

A B

Figure S 99. Pictures of *Conchocarpus mastigophorus* Kallunki (Rutaceae). **A.** *Conchocarpus mastigophorus* Kallunki specimen at the collection site. **B.** A collected branch of *Conchocarpus mastigophorus* Kallunki. All pictures are original photographs taken by the authors.
